# Nickel-catalyzed Suzuki–Miyaura cross-couplings of aldehydes

**DOI:** 10.1038/s41467-019-09766-x

**Published:** 2019-04-29

**Authors:** Lin Guo, Watchara Srimontree, Chen Zhu, Bholanath Maity, Xiangqian Liu, Luigi Cavallo, Magnus Rueping

**Affiliations:** 1 Institute of Organic Chemistry, RWTH Aachen University, Landoltweg 1, 52074 Aachen, Germany; 2 Kaust Catalysis Center (KCC), King Abdullah University of Science and Technology (KAUST), Thuwal, 23955-6900 Saudi Arabia

**Keywords:** Homogeneous catalysis, Catalyst synthesis, Synthetic chemistry methodology

## Abstract

Transition-metal-catalyzed cross-couplings have been extensively used in the pharmaceutical and agrochemical industries for the construction of diverse C–C bonds. Conventional cross-coupling reactions require reactive electrophilic coupling partners, such as organohalides or sulfonates, which are not environmentally friendly and not naturally abundant. Another disadvantage associated with these transformations is the need for an exogenous base to facilitate the key transmetalation step, and this reagent inevitably induces side reactions and limits the substrate scope. Here, we report an unconventional Suzuki-type approach to the synthesis of biaryls, through nickel-catalyzed deformylative cross coupling of aldehydes with organoboron reagents under base-free conditions. The transformation tolerates structurally diverse (hetero)aryl substituents on both coupling partners and shows high reactivity and excellent functional group tolerance. Furthermore, the protocol was carried out on gram scale and successfully applied to the functionalization of complex biologically active molecules. Mechanistic investigations support a catalytic cycle involving the oxidative addition of the nickel into the aldehyde C(acyl)–H bond with subsequent hydride transfer, transmetalation, decarbonylation and reductive elimination processes.

## Abstract (web summary)

Classical Suzuki-Miyaura couplings require wasteful electrophilic coupling partners and an exogenous base often engaging in side-reactions. Here, the authors report an alternative Suzuki-type synthesis of biaryls, involving the base-free nickel-catalyzed deformylative coupling of aldehydes with organoboron reagents.

## Introduction

**Fig. 1:**
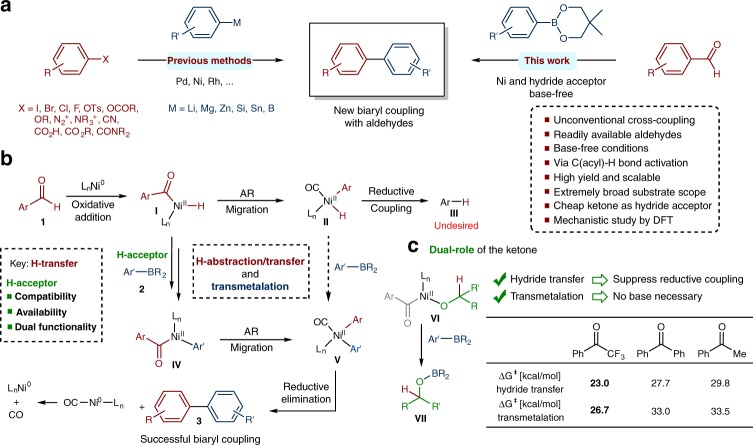
Nickel-catalyzed deformylative Suzuki–Miyaura cross coupling and related DFT studies on the role of trifluoroacetophenone. **a** Previously reported transition-metal-catalyzed construction of biaryl motifs (left) and nickel-catalyzed deformylative cross coupling for the synthesis of biaryls (right). **b** Proposed mechanism for the nickel-catalyzed arylation of aldehydes. **c** Dual-role of the ketone in the hydride-transfer and transmetalation steps

The development of efficient methods for the selective construction of C–C bonds is of great significance because carbon skeletons exist in numerous biologically active molecules, pharmaceuticals, and functional materials^1^. Since the 1970s, transition-metal-catalyzed cross-coupling reactions have attracted increasing attention, and they have become indispensable tools in the organic chemist’s arsenal^2,3^. Although the cross coupling of aryl halides and sulfonates with a variety of organometallic or main-group reagents has been successfully applied in synthetic transformations, the generation of corrosive halogen and sulfur containing waste is detrimental from synthetic, as well as environmental perspectives. With the rapid development of organometallic chemistry, impressive progress has recently been achieved in the use of C–O, C–N and carbonyl electrophiles as attractive alternative coupling partners (Fig. 1a, left)^4–8^. However, challenges still exist with these electrophiles, limiting the scope and applicability of their reactions. For instance, protocols using C–O electrophiles are often hampered by the “naphthalene problem” in which only π-extended aromatic frameworks show high reactivity^9,10^; couplings of aromatic carboxylic acids to build biaryl species via decarboxylative pathways are restricted to electron-withdrawing group-containing substrates or a strong oxidant is required^11,12^; the synthesis of biaryl species via the decarbonylative reaction of esters and amides is limited to specific substrates (phenyl esters and twisted amides) that require an additional step to be synthesized from carboxylic acids^13,14^; and the use of an exogenous base may also limit the scope of reagents and substrates^15^.

Based on the considerations mentioned above, an ideal synthetic approach to construct C–C bonds would be to use cross-coupling partners that are inexpensive, nontoxic, and readily available under conditions that are highly tolerant of diverse functional groups and are base free. Recently, the utilization of aldehydes as coupling partners has attracted growing interest in both academic and pharmaceutical laboratories^16^. Prompted by the synthetic relevance of biaryl compounds, which are ubiquitous in the skeletons of pharmaceuticals and organic materials^1^, we questioned whether it would be possible to build biaryl units through a formal deformylative pathway using aldehyde precursors^17,18^. To date and to the best of our knowledge, a general synthesis of biaryl species via the C–C cross-couplings of aldehydes with organometallic reagents is unknown.

As part of our interest in developing viable protocols for the activation of inert chemical bonds using transition-metal catalysis^19^, we herein report the nickel-catalyzed Suzuki–Miyaura cross coupling of aldehydes with organoboron compounds under base-free conditions (Fig. 1a, right). The catalytic C–C bond formation proceeds smoothly with various aromatic and heteroaromatic aldehydes, providing a useful synthetic strategy that uses aldehydes as unconventional electrophilic coupling partners in cross-coupling reactions that will inspire further exploration.

## Results

### Rational design

A description of our mechanistic concept is outlined in Fig. 1b and shows the challenges that must be addressed to develop the nickel-catalyzed biaryl coupling of aldehydes. Our initial efforts sought to identify conditions that avoid the undesired decarbonylation (**I** to **II**) of the acylnickel^(II)^ hydride species that arises from the oxidative addition of the C(acyl)-H bond of the aldehyde to L_n_Ni^(0)^^20,21^. Arylnickel^(II)^ hydride **II** preferentially undergoes very fast reductive elimination (**II** to **III**) rather than transmetalation (**II** to **V**). Thus, to avoid the undesired reductive coupling and formation of arene **III**^22^, we envisioned that a key hydride abstraction or transfer from the acylnickel^(II)^ hydride to a suitable H-acceptor would enable the transmetalation (**I** to **IV**) while suppressing the decarbonylation-reductive coupling pathway. The subsequent aryl migration (**IV** to **V**) and reductive elimination would provide the cross-coupling product and regenerate the Ni catalyst. Based on these considerations, the ideal H-acceptor needs to (i) be perfectly compatible; (ii) be readily available; and (iii) have dual functionality in that it can both suppress reductive coupling through hydride abstraction and simultaneously promote transmetalation to allow base-free conditions (Fig. 1c). Therefore, ketones were considered as possible H-acceptors because they would suit all of these requirements (they are readily available, stable, compatible, and good H-acceptors for the hydride-transfer step, and the formed alcoholate is a good activator for the transmetalation of the boronate). This hypothesis was corroborated by DFT studies (Fig. 1c), which accompanied the experimental study and revealed that the energy barriers for hydride transfer and base-free transmetalation were significantly decreased (23.0 and 26.7 kcal/mol, respectively) by the presence of trifluoroacetophenone^23^, while the same decreases were not seen with other acceptors, such as benzophenone and acetophenone.

### Optimization of the reaction conditions

**Fig. 2:**
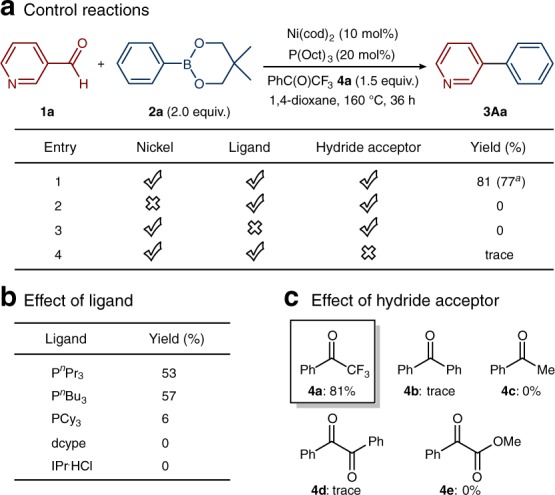
Optimization of the conditions for the Ni-catalyzed deformylative Suzuki–Miyaura cross coupling and a scaled-up reaction. **a** Optimal conditions and results of the control reactions. **b** Effect of the supporting ligand. **c**, Effect of the hydride acceptor. Yields were determined by gas chromatography (**a**, **b**, and **c**). Cod 1,5-cyclooctadiene, Oct octyl, Pr propyl, Bu butyl, Ph phenyl, Me methyl, dcype 1,2-bis(dicyclohexylphosphino)ethane, IPr•HCl 1,3-bis(2,6-diisopropylphenyl)imidazolium chloride. See Supplementary Tables 1–4 for details

Based on our mechanistic design, we began our primary investigations. Nicotinaldehyde (**1a**) and phenylboronic acid neopentylglycol ester (**2a**) were chosen as coupling partners in the presence of a nickel/ligand catalytic system with the use of a ketone as a hydride acceptor. After systematically evaluating the reaction parameters, we found that a combination of Ni(cod)_2_ and trioctylphosphine [P(Oct)_3_] afforded the biaryl product in 77% yield under base-free conditions when trifluoroacetophenone (**4a**) was employed as the hydride acceptor. Control experiments revealed that each reagent, namely, the catalyst, ligand and hydride acceptor, were all critical for the success of this reaction (Fig. 2a). Attempts under relatively lower temperature (130 °C) gave moderate yield. To date, reactions with other organoboron reagents have been less productive (see Supplementary Table 1). Regarding the influence of the ligand, replacement of trioctylphosphine with tri-*n*-propylphosphine (P^*n*^Pr_3_) or tri-*n*-butylphosphine (P^*n*^Bu_3_) under identical reaction conditions slightly decreased the yield, while the use of a bidentate phosphine ligand (dcype) or *N*-heterocyclic carbene ligand IPr•HCl gave unsatisfactory results (Fig. 2b). To facilitate the key hydride-transfer process, we evaluated several ketones (**4a–e**). However, for the reasons described, trifluoroacetophenone (**4a**) was the most effective hydride acceptor (Fig. 2c). Furthermore, commercially available and air-stable nickel precatalyst, Ni(OAc)_2_^.^4H_2_O can be applied but results in lower yields (see Supplementary Table 3).

### Substrate scope

**Fig. 3:**
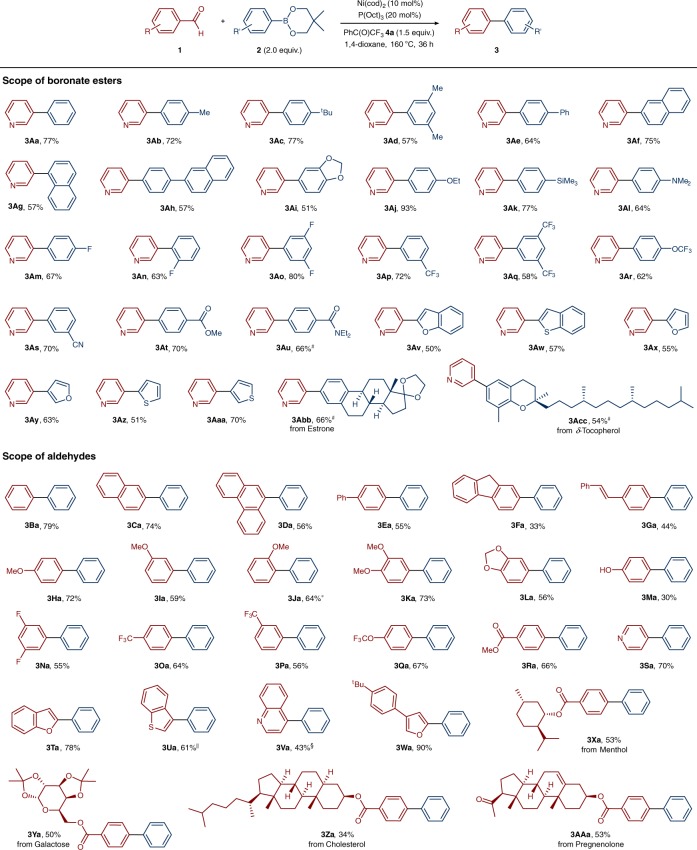
Substrate scope of the Ni-catalyzed deformylative Suzuki–Miyaura cross coupling. Reaction conditions: aldehyde **1** (0.20 mmol), boronate ester **2** (0.4 mmol, 2.0 equiv.), Ni(cod)_2_ (0.02 mmol, 10 mol%), P(Oct)_3_ (0.04 mmol, 20 mol%), and α,α,α-trifluoroacetophenone (**4a**, 0.3 mmol, 1.5 equiv.) in 1,4-dioxane (1.5 ml) at 160 °C for 36 h. Yields after isolation. #1.5 equiv. of boronate ester **2** was used. *Reaction was performed with 15 mol% Ni(cod)_2_ and 30 mol% P(Oct)_3_ for 72 h. **||**Reaction was performed with 15 mol% Ni(cod)_2_ and 30 mol% P(Oct)_3_ for 60 h. §Reaction was performed for 60 h

Encouraged by the initial results, we examined the scope of the reaction with respect to various aromatic and heteroaromatic boronic ester nucleophiles in combination with nicotinaldehyde (**1a**) as the coupling partner. As shown in Fig. 3, we found that with our catalytic system, various phenyl boronic ester substrates, bearing either electron-donating or electron-withdrawing functional groups, could be converted into the corresponding biaryl products in good yields. The use of methyl-substituted or *tert*-butyl-substituted phenylboronic esters smoothly provided the corresponding products (**3Ab**–**3Ad**), whereas the use of biphenyl boronic ester gave **3Ae** in 64% yield. Furthermore, not only naphthyl-derived substrates (**3Af**, **3Ag**) but also phenyl boronic ester derivatives possessing ethoxy (**3Aj**), silyl (**3Ak**), amine (**3Al**), fluoro (**3Am**–**3Ao**), trifluoromethyl (**3Ap**, **3Aq**), trifluoromethoxy (**3Ar**), cyano (**3As**), ester (**3At**), and amide (**3Au**) substituents are perfectly suitable for the transformation and gave the desired products in good yields. Importantly, our decarbonylative cross-coupling reaction could be readily extended to heteroaromatic boronic esters derived from (benzo)furan and (benzo)thiophene, affording the corresponding biheteroaryl motifs **3Av**–**3Aaa**. In addition, the two examples of estrone and δ-tocopherol derivatives (**3Abb** and **3Acc**) highlight the applicability of this method for the late-stage modification of complex molecules. We subsequently turned our attention to a series of aldehydes as electrophilic coupling partners to determine the scope of our method. Figure 3 also shows the excellent chemoselectivity profile of the developed method; phenyl, naphthyl, phenanthryl and fluorenyl aldehydes were suitable for this transformation (**3Ba**–**3Fa**). Although nickel catalysts have been successfully used for arylation reactions with boronic ester nucleophiles through C–OMe^11^ or C–F^24^ cleavage, we found that, in our case, these cleavage reactions did not compete with the deformylative biaryl synthesis (**3Ha**–**3Ka** and **3Na**). This chemoselectivity is a general requirement for an applicable reaction and is also important for retrosynthesis planning. In addition, the chemoselectivity of this method was nicely illustrated by the fact that functional groups such as dioxole (**3La**), trifluoromethyl (**3Oa** and **3Pa**), trifluoromethoxy (**3Qa**), and ester (**3Ra**) were well tolerated under the present conditions. It was further demonstrated that pyridine-derived, benzofuran-derived, benzothiophene-derived, quinoline-derived, and furan-derived heterocyclic aldehydes (**3Sa**–**3Wa**) did not hinder the success of the transformation. Regarding the mentioned chemoselectivity, bioactive natural product compounds such as menthol, galactose, cholesterol and pregnenolone were also suitable substrates for this reaction, effectively providing the desired products **3Xa**–**3AAa**.

### Mechanistic studies

**Fig. 4:**
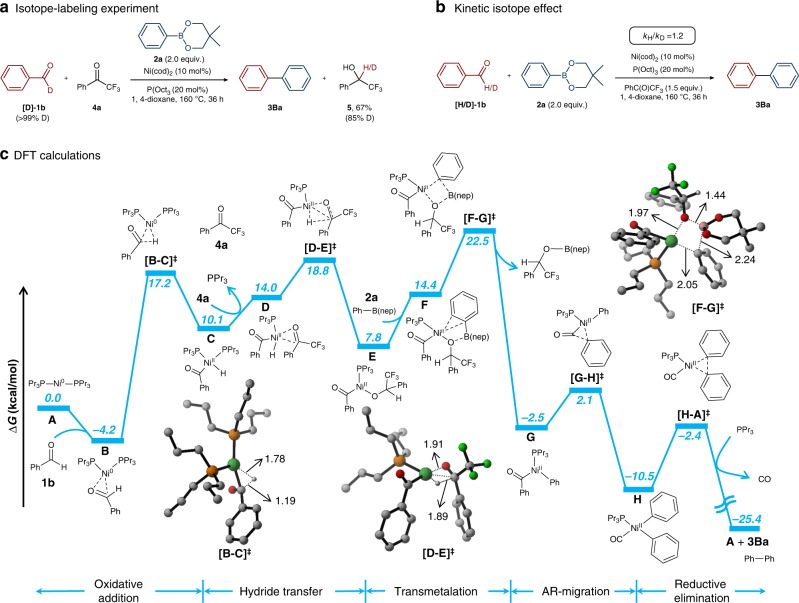
Mechanistic studies of the Ni-catalyzed deformylative Suzuki–Miyaura cross coupling. **a** Isotope-labeling experiment. **b** Kinetic isotope effect experiment. **c** Full DFT-computed energy profile for the Ni-catalyzed deformylative Suzuki–Miyaura cross coupling with benzaldehyde (**1b**) and Ph-B(nep) (**2a**) as reactants and PhC(O)CF_3_ (**4a**) as the hydride acceptor under the Ni(cod)_2_/P^*n*^Pr_3_ catalytic system. Free energies in solution (in kcal/mol) at the SMD (1,4-Dioxane)-M06/Def2-TZVPP//ωB97xD/Def2-TZVP(Ni)/Def2-SVP(non-metal) level are displayed. Selected DFT optimized geometries are listed. Bond lengths are given in Å

We conducted several experiments to elucidate the reaction mechanism. We first performed an isotope-labeling experiment with a deuterated benzaldehyde. The deuterium of [D_1_]-**1b** was shown to add to the carbonyl group of the hydrogen acceptor (trifluoroacetophenone, **4a**) to give [D_1_]−2,2,2-trifluoro-1-phenylethan-1-ol (**5**) upon hydrolysis, which provides direct evidence for the occurrence of a hydride-transfer process during the cross-coupling reaction (Fig. 4a). To determine whether this hydride-transfer step is the rate-determining step of this cross-coupling process, we carried out a KIE (kinetic isotope effect) experiment. A minor kinetic isotope effect (*k*_H_/*k*_D_ = 1.2) was found when using isotopically labeled aldehyde substrate [D_1_]-**1b**. This observation indicates the C(acyl)-H insertion and hydride-transfer processes are relatively fast (Fig. 4b).

Furthermore, we performed detailed DFT calculations (Fig. 4 and Supplementary Discussion) to rationalize and support our proposed mechanism for the nickel-catalyzed arylation of aldehydes. As a model system, we investigated the reaction of aldehyde **1b** with phenylboronic ester **2a** in the presence of Ni^0^(P^n^Pr_3_)_2_ (**A**) as the active catalyst. The reaction starts with the oxidative addition of aldehyde **1b** to the active catalyst, Ni^0^(P^n^Pr_3_)_2_ (**A**), which proceeds via adduct **B** and transition state [**B–C**]^**‡**^. The C(acyl)-H activation step has an energy barrier of 21.4 kcal/mol, while the barrier for C(aryl)-C(acyl) activation is 6.0 kcal/mol higher (Fig. S1). In the next step, resulting (acyl)Ni^II^-H species **C** binds **4a** and undergoes hydride transfer via transition state [**D–E**]^**‡**^, generating (acyl)Ni^II^-alkoxide complex **E**. Next, in the presence of aryl boronic ester **2a**, transmetalation of **E** occurs via transition state [**F–G**]^**‡**^. Upon liberation of PhCF_3_CHOB(nep), Ni^II^ intermediate **G** is formed, which undergoes a smooth CO migration to give Ni^II^ complex **H**. Finally, decarbonylation and reductive elimination affords biaryl product **3Ba** and regenerates active catalyst **A**, which coordinates to aldehyde **1b** to initiate the next catalytic cycle. Overall, the energy profile reveals that the transmetalation is the rate-limiting step, with an energy barrier of 26.7 kcal/mol. Notably, compound **4a** not only acts as an H-acceptor but also facilitates the transmetalation step to allow the cross-coupling to proceed in the absence of a base.

## Discussion

In summary, we have developed a nickel-catalyzed decarbonylative arylation reaction of aldehydes with boronic esters. In contrast to classical cross-coupling reactions, this protocol allows the use of inexpensive, nontoxic, and readily available aldehydes as unconventional coupling electrophiles. The high reactivity, broad substrate scope, and scalability of this method suggest that this protocol can be a powerful alternative to the existing methodologies for the synthesis of structurally diverse biaryls. Importantly, our developed protocol shows high chemoselectivity, and functional groups, including C–OMe, C–F, and C–CO_2_R, which can be reactive in nickel-catalyzed functional group interconversions, are preserved. Moreover, mechanistic studies based on control experiments and DFT calculations revealed that the formation of biaryl products follows a reaction pathway that includes the oxidative addition of the C(acyl)-H bond of aldehyde to a Ni species with subsequent hydrogen transfer, transmetalation, aryl migration, and reductive elimination. Based on the described strategy further C–C and C-heteroatom bond forming reactions of aldehydes are expected.

## Methods

### General procedure for the deformylative coupling

In a nitrogen-filled glovebox, a 10-mL oven-dried sealed tube containing a stirring bar was charged with the corresponding aldehyde **1** (0.20 mmol, 1.0 equiv.), aryl/heteroaryl boronic ester **2** (0.40 mmol, 2.0 equiv.) and yellow Ni(cod)_2_ (5.5 mg, 10 mol%). Subsequently, HPLC grade 1,4-dioxane (1.5 mL) was added, and then trioctylphosphine ligand (18 μL, 20 mol%), and 2,2,2-trifluoroacetophenone (42 μL, 0.30 mmol, 1.5 equiv.) were added, respectively, via microsyringe. The tube with the mixture was sealed and removed from the glovebox. After stirring at 160 °C for 36 h, the mixture was allowed to cool to room temperature, diluted with EtOAc (5 mL) and filtered through a celite plug, eluting with additional EtOAc (15 mL). The filtrate was concentrated and purified by column chromatography on silica gel to yield the title product.

### Computational details

All the geometries were optimized with the generalized gradient approximation (GGA) method with  Gaussian 09, Revision D.01,^25^ using the long-range-corrected hybrid DFT functional ωB97xD^26^. The electronic configuration of all the non-metal elements was described with the Ahlrichs split-valance polarization basis function Def2-SVP while Ni is treated with the triple-ζ valence basis set Def2-TZVP^27,28^. The geometries were optimized without any symmetry constraints. Harmonic force constants were computed at the optimized geometries to characterize the stationary points as minima or saddle points. All transition states were optimized using the default Berny algorithm implemented in the Gaussian09 code^25^. For transition state structures, IRC calculations were undertaken to confirm the transition states which were connected to the correct minima. For further validation of energetics, single-point calculations were performed on the ωB97xD/Def2-TZVP(Ni)/Def2-SVP(non-metal) optimized geometries using meta-hybrid-GGA functional M06^29^ employing a valence triple-ζ-type of basis set Def2-TZVPP^27,28^ for all atoms. The solvent effects (1,4-dioxane, ε = 2.2099) were evaluated implicitly by a self-consistent reaction field (SCRF) approach for all the intermediates and transitions states, using the SMD continuum solvation model^30^. Unless specified otherwise, the Δ*G* was used throughout the text. The Δ*G* value was obtained by augmenting the E_el_ energy terms at M06(SMD)/Def2-TZVPP with the respective free energy corrections at the ωB97xD/Def2-TZVP (Ni)/Def2-SVP (non-metal) level in gas phase. In all cases, the default integral grid (Fine Grid) was employed. The simplified trialkyl-phosphine ligand P^n^Pr_3_ was used in the DFT calculation, which gave 53% yield (see Supplementary Table 1, entry 4).

## Supplementary information

Supplematary Information

Description of Additional Supplementary Files

Supplementary Data

## Data Availability

Experimental details, characterization of compounds, copies of NMR data and details of DFT calculations are available with the submitted manuscript.

## References

[1] Hassan J, Sévignon M, Gozzi C, Schulz E, Lemaire M (2002). Aryl−aryl bond formation one century after the discovery of the Ullmann reaction. Chem. Rev..

[2] Magano J, Dunetz JR (2011). Large-scale applications of transition metal-catalyzed couplings for the synthesis of pharmaceuticals. Chem. Rev..

[3] Negishi E (2011). Magical power of transition metals: past, present, and future. Angew Chem. Int. Ed..

[4] Cornella J, Zarate C, Martin R (2014). Metal-catalyzed activation of ethers via C–O bond cleavage: a new strategy for molecular diversity. Chem. Soc. Rev..

[5] Wang Q, Su Y, Li L, Huang H (2016). Transition-metal catalysed C–N bond activation. Chem. Soc. Rev..

[6] Rodríguez N, Gooßen LJ (2011). Decarboxylative coupling reactions: a modern strategy for C–C-bond formation. Chem. Soc. Rev..

[7] Takise R, Muto K, Yamaguchi J (2017). Cross-coupling of aromatic esters and amides. Chem. Soc. Rev..

[8] Guo L, Rueping M (2018). Transition-metal-catalyzed decarbonylative coupling reactions: concepts, classifications, and applications. Chem. Eur. J..

[9] Tobisu M, Chatani N (2015). Cross-couplings using aryl ethers via C−O bond activation enabled by nickel catalysts. Acc. Chem. Res..

[10] Tobisu M, Shimasaki T, Chatani N (2008). Nickel-catalyzed cross-coupling of aryl methyl ethers with aryl boronic esters. Angew. Chem. Int. Ed..

[11] Gooßen LJ, Deng G, Levy LM (2006). Synthesis of biaryls via catalytic decarboxylative coupling. Science.

[12] Dai JJ, Liu JH, Luo DF, Liu L (2011). Pd-catalysed decarboxylative Suzuki reactions and orthogonal Cu-based O-arylation of aromatic carboxylic acids. Chem. Commun..

[13] Muto K, Yamaguchi J, Musaev DG, Itami K (2015). Decarbonylative organoboron cross-coupling of esters by nickel catalysis. Nat. Commun..

[14] Shi S, Meng G, Szostak M (2016). Synthesis of biaryls through nickel-catalyzed Suzuki-Miyaura coupling of amides by carbon–nitrogen bond cleavage. Angew. Chem. Int. Ed..

[15] Cox PA, Reid M, Leach AG, Campbell AD, King EJ, Lloyd-Jones GC (2017). Base-catalyzed aryl-B(OH)2 protodeboronation revisited: from concerted proton transfer to liberation of a transient aryl anion. J. Am. Chem. Soc..

[16] Wu XF (2015). Acylation of (hetero)arenes through C–H activation with aroyl surrogates. Chem. Eur. J..

[17] Guo X, Wang J, Li CJ (2009). An olefination via ruthenium-catalyzed decarbonylative addition of aldehydes to terminal alkynes. J. Am. Chem. Soc..

[18] Shuai Q, Yang L, Guo X, Baslé O, Li CJ (2010). Rhodium-catalyzed oxidative C–H arylation of 2-arylpyridine derivatives via decarbonylation of aromatic aldehydes. J. Am. Chem. Soc..

[19] Guo L, Rueping M (2018). Decarbonylative cross-couplings: nickel catalyzed functional group interconversion strategies for the construction of complex organic molecules. Acc. Chem. Res..

[20] Yu H, Fu Y (2012). Mechanistic origin of cross−coupling selectivity in Ni-catalysed Tishchenko reactions. Chem. Eur. J..

[21] Xiao LJ (2016). Nickel-catalyzed hydroacylation of styrenes with simple aldehydes: reaction development and mechanistic insights. J. Am. Chem. Soc..

[22] Ding K, Xu S, Alotaibi R, Paudel K, Reinheimer EW, Weatherly J (2017). Nickel-catalyzed decarbonylation of aromatic aldehydes. J. Org. Chem..

[23] Whittaker AM, Dong VM (2015). Nickel-catalyzed dehydrogenative cross-coupling: direct transformation of aldehydes into esters and amides. Angew. Chem. Int. Ed..

[24] Tobisu M, Xu T, Shimasaki T, Chatani N (2011). Nickel-catalyzed Suzuki-Miyaura reaction of aryl fluorides. J. Am. Chem. Soc..

[25] Frisch, M. J. et al. Gaussian 09 Revision D.01 (Gaussian 2013).

[26] Chai JD, Head-Gordon M (2008). Long-range corrected hybrid density functionals with damped atom–atom dispersion corrections. Phys. Chem. Chem. Phys..

[27] Weigend F, Ahlrichs R (2005). Balanced basis sets of split valence, triple zeta valence and quadruple zeta valence quality for H to Rn: Design and assessment of accuracy. Phys. Chem. Chem. Phys..

[28] Weigend F (2006). Accurate Coulomb-fitting basis sets for H to Rn. Phys. Chem. Chem. Phys..

[29] Zhao Y, Truhlar DG (2008). The M06 suite of density functionals for main group thermochemistry, thermochemical kinetics, noncovalent interactions, excited states, and transition elements: two new functionals and systematic testing of four M06-class functionals and 12 other functionals. Theor. Chem. Acc..

[30] Marenich AV, Cramer CJ, Truhlar DG (2009). Universal solvation model based on solute electron density and on a continuum model of the solvent defined by the bulk dielectric constant and atomic surface tensions. J. Phys. Chem. B.

