# Supplementary material for: Nickel-catalyzed Suzuki–Miyaura cross-couplings of aldehydes
Source: Nat Commun. 2019 Apr 29;10:1957. doi: 10.1038/s41467-019-09766-x (PMC6488620; doi:10.1038/s41467-019-09766-x)
Supplement: Supplementary file 1 — Supplematary Information [file 41467_2019_9766_MOESM1_ESM.pdf]

Supplementary Information

Nickel-Catalyzed Suzuki-Miyaura Cross-Couplings of Aldehydes

Guo, L. *et al*

Supplementary Methods

Supplementary Note 1. General Information

Unless otherwise noted, all commercially available compounds were used as provided without further purification. Solvents for chromatography were technical grade and freshly distilled prior to use. Ni(cod)$_2$, trioctylphosphine ligand and 2,2,2-trifluoroacetophenone were purchased from Alfa Aesar and Sigma-Aldrich. 1,4-Dioxane and toluene used in reactions were HPLC grade without further distillation. Analytical thin-layer chromatography (TLC) was performed on Merck silica gel aluminium plates with F-254 indicator, visualised by irradiation with UV light. Column chromatography was performed using silica gel (Macherey Nagel, particle size 0.040-0.063 mm). Solvent mixtures are understood as volume/volume. ^{1}H-NMR, ^{13}C-NMR and ^{19}F-NMR were recorded on a Varian AV400 or AV600 spectrometer in $CDCl_3$ and are reported relative to the solvents residual ^{1}H-signal ($CHCl_3$, δ(H) 7.26). Data are reported in the following order: chemical shift (δ) in ppm; multiplicities are indicated s (singlet), bs (broad singlet), d (doublet), t (triplet), q (quartet), m (multiplet); coupling constants (*J*) are in Hertz (Hz). Melting points were measured using open glass capillaries in a Buchi SMP-20 apparatus. IR spectra were recorded on a Perkin Elmer-100 spectrometer and are reported in terms of frequency of absorption (cm^{-1}). Mass spectra (EI-MS, 70 eV) were conducted on a Finnigan SSQ 7000 spectrometer. HRMS were recorded on a Thermo Scientific LTQ Orbitrap XL spectrometer.

Supplementary Tables

Supplementary Table 1. Optimization details

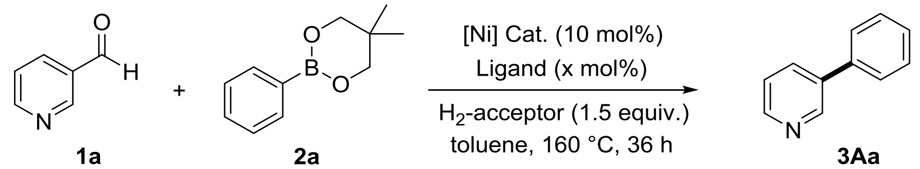

Entry	[Ni] Cat. (10 mol%)	Ligand (x mol%)	Hydride Acceptor	Yield (%)[a,b]
1[c]	$Ni(cod)_2$	P^nBu_3 (20)	**4a**	trace
2[d]	$Ni(cod)_2$	P^nBu_3 (20)	**4a**	trace
3	$Ni(cod)_2$	P^nBu_3 (20)	**4a**	57
4	$Ni(cod)_2$	P^nPr_3 (20)	**4a**	53
5	$Ni(cod)_2$	$P(Oct)_3$ (20)	**4a**	68
6	$Ni(cod)_2$	dcype (10)	**4a**	-
7	$Ni(cod)_2$	IPr·HCl (20)	**4a**	-
8	$Ni(cod)_2$	-	**4a**	-
9	$Ni(cod)_2$	$P(Oct)_3$ (20)	-	trace
10	$Ni(cod)_2$	$P(Oct)_3$ (20)	**4b**	-
11	$Ni(cod)_2$	$P(Oct)_3$ (20)	**4c**	trace
12	$Ni(cod)_2$	$P(Oct)_3$ (20)	**4d**	trace
13	$Ni(cod)_2$	$P(Oct)_3$ (20)	**4e**	-
14	-	$P(Oct)_3$ (20)	**4a**	-
15	$Ni(OAc)_2·4H_2O$	$P(Oct)_3$ (20)	**4a**	22
16	$NiBr_2$	$P(Oct)_3$ (20)	**4a**	-
**17[e]**	**$Ni(cod)_2$**	**$P(Oct)_3$ (20)**	**4a**	**81(77[f])**
18[e,g]	$Ni(cod)_2$	$P(Oct)_3$ (20)	**4a**	69

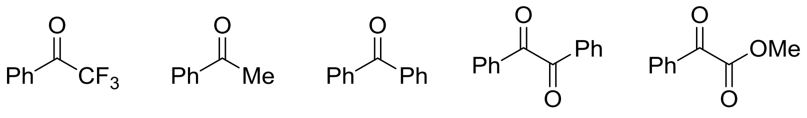

4a **4b** **4c** **4d** **4e**

[a] Reaction conditions: aldehyde **1a** (0.20 mmol), Ph-B(nep) **2a** (0.40 mmol, 2.0 equiv.), [Ni] catalyst (0.02 mmol, 10 mol%), ligand (0.02 mmol or 0.04 mmol) and hydride acceptor **4** (0.30 mmol, 1.5 equiv.) in toluene (1.5 ml) at 160 °C for 36 h. [b] Determined by GC analysis (decane as internal standard). [c] $Ph-B(OH)_2$ (**2a'**) (2.0 equiv.) as coupling nucleophile instead of **2a**. [d] Ph-B(pin) (**2a''**) (2.0 equiv.) as coupling nucleophile instead of **2a**. [e] 1,4-Dioxane as solvent. [f] Yield for isolated product. [g] 150 °C. $P(Oct)_3$ = trioctylphosphine, dcype = 1,2-bis(dicyclohexylphosphino)-ethane, IPr·HCl = 1,3-bis(2,6-diisopropyl-phenyl)imidazolium chloride, B(nep) = 5,5-dimethyl-1,3,2-dioxaborolane, B(pin) = 4,4,5,5-tetramethyl-1,3,2-dioxaborolane.

Supplementary Table 2. Control reactions

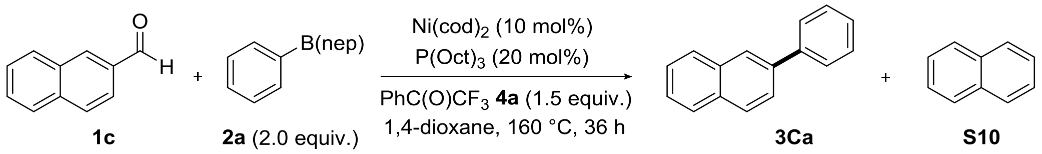

Entry	Changes	Conv. of **1c** (%)[a]	Yield of **3Ca** (%)[a]	Yield of **S10** (%)[a]
1	Without Ph-B(nep) **2a**	47%	0%	20%
2	Without $PhC(O)CF_3$ **4a**	94%	4%	35%
3	Without **2a** and **4a**	51%	0%	7%

[a] Determined by GC analysis (decane as internal standard).

Supplementary Table 3. Screening of air-stable Ni precatalyst

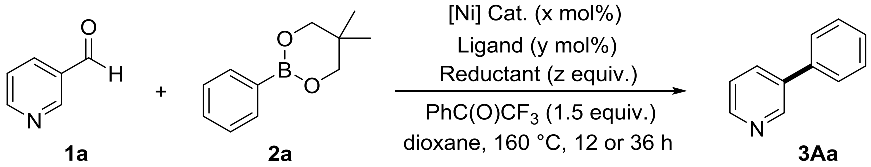

Entry	[Ni] Cat. (x mol%)	Ligand (y mol%)	Reductant (z equiv.)	Time (h)	Yield (%)[a,b]
1	$NiCl_2$ (10)	$P(Oct)_3$ (20)	-	12	-
2	$NiCl_2$ (10)	$P(Oct)_3$ (20)	Zn (2)	12	4
3	$NiCl_2$ (10)	$P(Oct)_3$ (20)	Mn (2)	12	-
4	$NiCl_2$ (10)	$P(Oct)_3$ (20)	Mg (2)	12	-
5	$NiCl_2$ (20)	$P(Oct)_3$ (40)	LiOMe (1.5)	12	-
6	$NiCl_2$ (20)	$P(Oct)_3$ (40)	KO*t*Bu (1.5)	12	-
7	$NiCl_2$ (20)	$P(Oct)_3$ (40)	NaOMe (1.5)	12	18
8	$Ni(OAc)_2 \cdot 4H_2O$ (20)	$P(Oct)_3$ (40)	NaOMe (1.5)	12	15
9	$NiBr_2$ (20)	$P(Oct)_3$ (40)	NaOMe (1.5)	12	16
10	NiI_2 (20)	$P(Oct)_3$ (40)	NaOMe (1.5)	12	-
11	$Ni(OTf)_2$ (20)	$P(Oct)_3$ (40)	NaOMe (1.5)	12	-
12	$NiCl_2$ (20)	$P(Oct)_3$ (40)	NaOMe (1.5)	36	22
13	$Ni(OAc)_2 \cdot 4H_2O$ (20)	$P(Oct)_3$ (40)	NaOMe (1.5)	36	30
14	$Ni(OAc)_2 \cdot 4H_2O$ (20)	$P(Oct)_3$ (40)	NaOEt (1.5)	36	7

[a] Reaction conditions: aldehyde **1a** (0.20 mmol), Ph-B(nep) **2a** (0.40 mmol, 2.0 equiv.), [Ni] catalyst (x mol%), ligand (y mol%), reductant (z equiv.) and hydride acceptor **4a** (0.30 mmol, 1.5 equiv.) in 1,4-dioxane (1.5 ml) at 160 °C for 12 h or 36 h. [b] Determined by GC analysis (decane as internal standard).

Supplementary Note 2. Procedure for the screening of air-stable Ni precatalyst

Procedure: A 10-mL oven-dried sealed tube containing a stirring bar was charged with Ni(II) precatalyst (x mol%) and reductant (z equiv.) under the protection of argon. Subsequently, HPLC grade 1,4-dioxane (1.5 mL) was added by syringe and trioctylphosphine ligand (y mol%) was injected by microsyringe. The mixture was stirred at room temperature for 30 min. Then, aldehyde **1a** (19 μL, 0.20 mmol, 1.0 equiv.), 2,2,2-trifluoroacetophenone **4a** (42 μL, 0.30 mmol, 1.5 equiv.) and a 1,4-dioxane solution (0.5 mL) of boronic ester **2a** (76 mg, 0.40 mmol, 2.0 equiv.) were added by microsyringe or normal syringe under argon. The tube was sealed again and stirred at 160 °C for 36 h. The mixture was allowed to cool to room temperature, diluted with EtOAc (5 mL), and merged with decane (39 μL, 0.20 mmol) which is an internal standard. The yield was checked by GC.

Supplementary Table 4. Reaction under relatively lower temperature

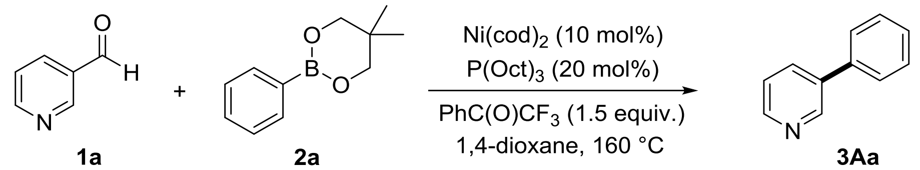

1a **2a** **3Aa**

Entry	Solvent	Temp. (°C)	Time (h)	Yield (%)[a,b]
1	1,4-dioxane	120	12	8
2	1,4-dioxane	130	12	21
3	1,4-dioxane	140	12	34
4	1,4-dioxane	130	36	32
5	1,4-dioxane	130	60	51
6	THF	130	36	5

[a] Reaction conditions: aldehyde **1a** (0.20 mmol), Ph-B(nep) **2a** (0.40 mmol, 2.0 equiv.), $Ni(cod)_2$ (10 mol%), $POct_3$ ligand (20 mol%), and hydride acceptor **4a** (0.30 mmol, 1.5 equiv.) in solvent (1.5 ml) at various temperatures for 12-60 h. [b] Determined by GC analysis (decane as internal standard).

Supplementary Figure 1. Overview of the aldehyde substrates applied in the protocol

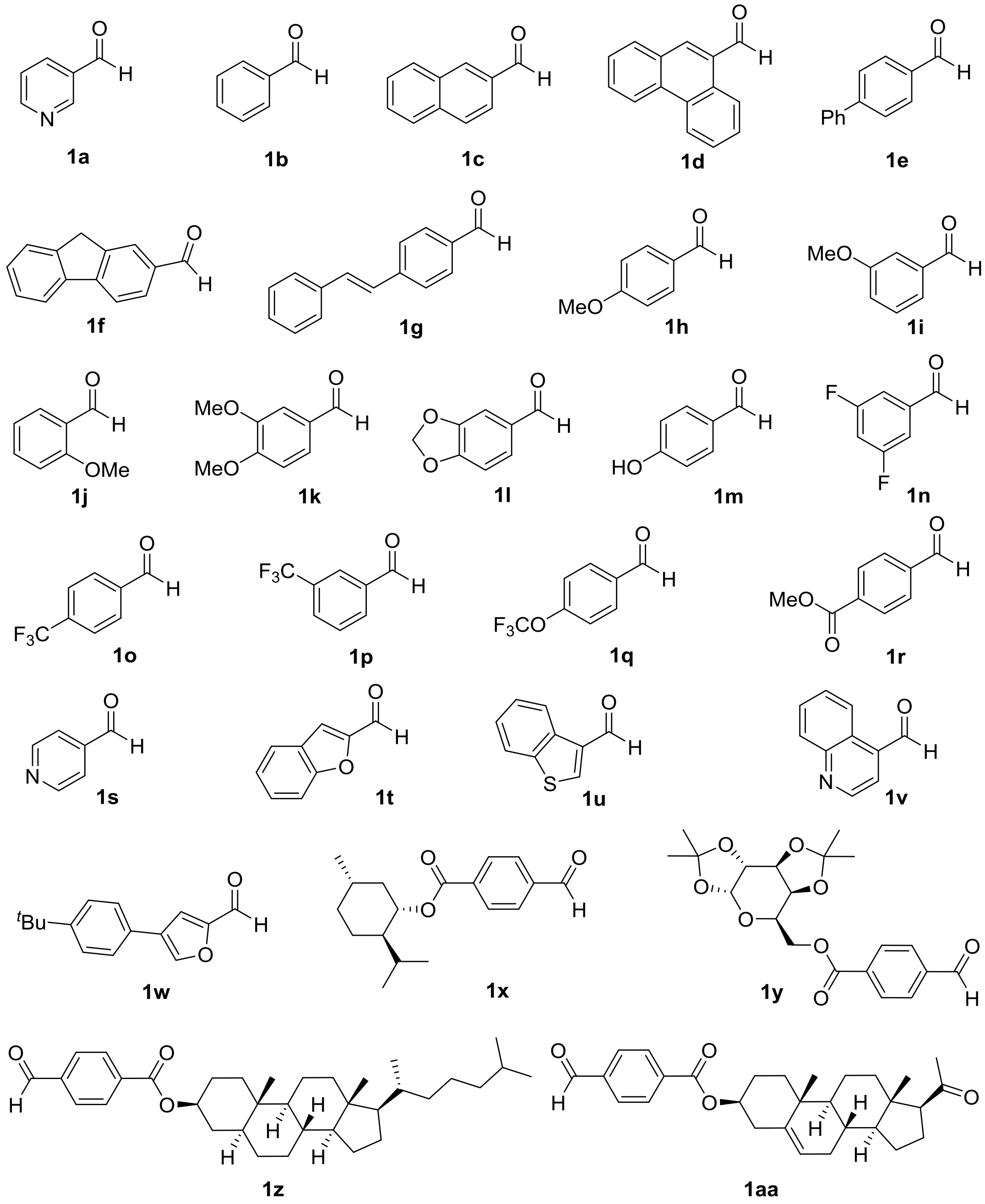

Supplementary Figure 2. Overview of the boronic ester substrates applied in the protocol

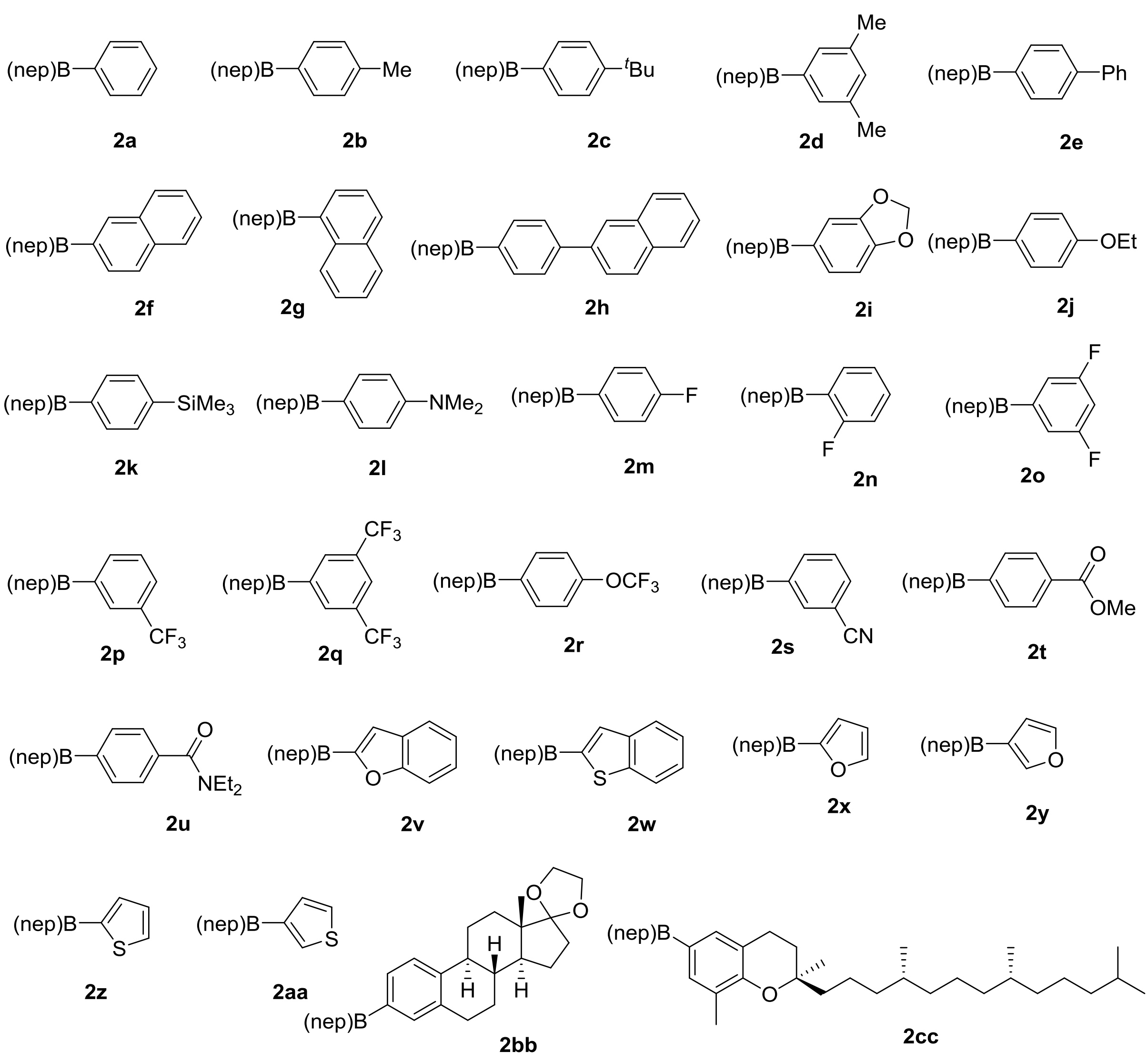

Supplementary Note 3. Synthesis and characterization of the aldehyde substrates

Substrates **1a-1v** in Supplementary Figure 1 are commercially available.

1w was prepared according to the method below.

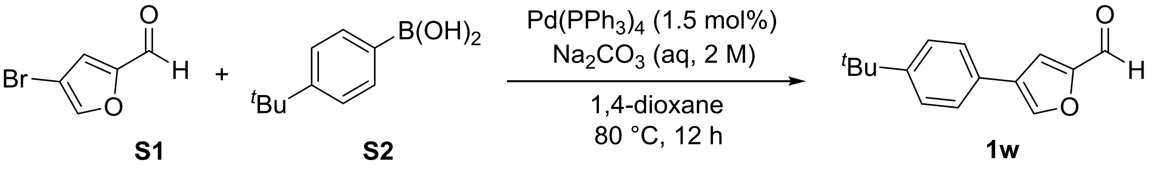

An oven-dried Schlenk flask was charged under nitrogen with 4-bromofuran-2-carbaldehyde **S1** (542 mg, 3.1 mmol), (4-(*tert*-butyl)phenyl)boronic acid **S2** (662 mg, 3.7 mmol), $Pd(PPh_3)_4$ (54 mg, 1.5 mol%), Na_2CO_3 (aq, 3 mL, 2 M) and 1,4-dioxane (15 mL). The Schlenk tube was sealed under nitrogen and placed in an oil bath preheated to 80 °C and the reaction mixture was stirred overnight at this temperature. After being cooled to room temperature, the reaction mixture was poured into water (30 mL) and extracted with ethyl acetate (3 × 20 mL). The combined organic extracts were washed with brine, dried over Na_2SO_4, and then filtered. The filtrate was concentrated in *vacuo* and the residue was purified by flash column chromatography to afford **1w** as a white solid (518 mg, 73%).

4-(4-(*Tert*-butyl)phenyl)furan-2-carbaldehyde (1w)

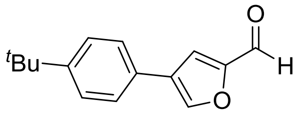

Mp: 86-87 °C; ^{1}H NMR (400 MHz, $CDCl_3$): δ 9.70 (s, 1H), 7.93 (s, 1H), 7.51 (s, 1H), 7.45 (s, 4H), 1.35 (s, 9H); ^{13}C NMR (100 MHz, $CDCl_3$): δ 178.2, 153.6, 151.4, 143.6, 129.3, 127.6, 126.1, 125.8, 34.8, 31.3; IR (ATR): 3220, 3125, 2956, 2320, 2091, 1918, 1681, 1479, 1353, 1222, 1041, 926, 758 cm^{-1}; MS (EI): m/z (%) 228.2 (M^+, 68), 213.1 (100), 185.0 (21); HRMS (ESI) for $C_{15}H_{16}O_2Na$: calcd. for $[M+Na]^+$ 251.10425, found 251.10426.

Substrates **1x-1aa** in Supplementary Figure 1 were prepared according to the method below.[1]

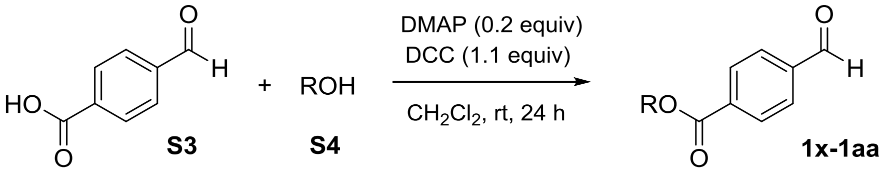

To a suspension of 4-formylbenzoic acid **S3** (0.5 g, 3.33 mmol) in dichloromethane (8 mL) were added *N*,*N*-dimethyl-4-aminopyridine (81.4 mg, 0.67 mmol), alcohol **S4** (1.13 g, 4.33 mmol) and *N*,*N*'-dicyclohexylcarbodiimide (0.756 g, 3.66 mmol). The mixture was strirred at room temperature for 24 h then diethyl ether (20 mL) was added. The precipitate was filtered through celite and the filtrate was successively washed with a 1 N aqueous solution of HCl (2 × 20 mL), saturated aqueous $NaHCO_3$ solution (2 × 20 mL) and brine (2 × 20 mL). The organic phase was then dried over anhydrous $MgSO_4$, filtered and concentrated under reduced pressure. Purification by flash chromatography (diethyl ether/petroleum ether 1:9) to yield the corresponding ester.

(1*S*,2*R*,5*S*)-2-Isopropyl-5-methylcyclohexyl 4-formylbenzoate (1x)

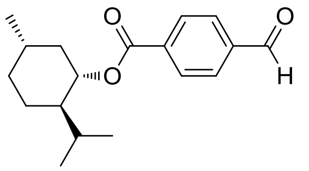

^{1}H NMR (600 MHz, $CDCl_3$) δ 10.09 (s, 1H), 8.18 (d, J = 8.3 Hz, 2H), 7.94 (d, J = 8.3 Hz, 2H), 4.95 (td, J = 10.9, 4.4 Hz, 1H), 2.14–2.09 (m, 1H), 1.96–1.89 (m, 1H), 1.75–1.71 (m, 2H), 1.58–1.53 (m, 2H), 1.15–1.09 (m, 2H), 0.93–0.90 (m, 7H), 0.78 (d, J = 7.0 Hz, 3H); ^{13}C NMR (151 MHz, $CDCl_3$) δ 191.7, 165.0, 139.0, 135.8, 130.1, 129.5, 75.6, 47.2, 40.9, 34.2, 31.4, 26.5, 23.6, 22.0, 20.7, 16.5; IR (ATR): 2945, 1707, 1577, 1457, 1377, 1270, 1199, 962, 818 cm^{-1}; MS (EI): m/z (%) 289.2 ([M + H]$^+$, 30), 138.2 (100), 105.1 (19), 81.2 (26), 55.2 (12); Data in accordance with the literature.[1]

((3a*R*,5*R*,5a*S*,8a*S*,8b*R*)-2,2,7,7-Tetramethyltetrahydro-3a*H*-bis([1,3]dioxolo)[4,5-b:4',5'-d]pyran-5-yl)methyl 4-formylbenzoate (1y)

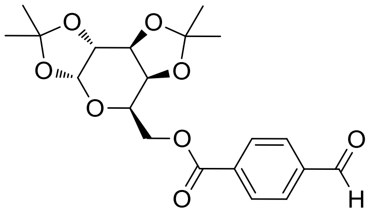

Mp: 88–90 °C; ^{1}H NMR (600 MHz, $CDCl_3$) δ 10.10 (s, 1H), 8.21 (d, J = 8.1 Hz, 2H), 7.95 (d, J = 8.2 Hz, 2H), 5.57 (d, J = 4.9 Hz, 1H), 4.66 (dd, J = 7.9, 2.4 Hz, 1H), 4.56 (dd, J = 11.6, 4.5 Hz, 1H), 4.47 (dd, J = 11.6, 7.8 Hz, 1H), 4.36 (dd, J = 4.9, 2.5 Hz, 1H), 4.33 (dd, J = 7.9, 1.3 Hz, 1H), 4.21–4.17 (m, 1H), 1.52 (s, 3H), 1.48 (s, 3H), 1.36 (s, 3H), 1.34 (s, 3H); ^{13}C NMR (151 MHz, $CDCl_3$) δ 191.7, 165.4, 139.2, 135.0, 130.3, 129.5, 109.8, 108.8, 96.3, 71.1, 70.7, 70.5, 66.1, 64.5, 26.0, 25.9, 25.0, 24.5; IR (ATR): 2987, 2917, 1713,1573, 1456, 1379, 1268, 1070, 896, 758 cm^{-1}; MS (EI): m/z (%) 393.1 ([M + H]$^+$, 41), 377.0 (100), 319.0 (24), 259.0 (10), 227.0 (28), 169.0 (27), 133.0 (62), 100.1 (33), 81.1 (38); Data in accordance with the literature.[2]

(3*S*,5*S*,8*R*,9*S*,10*S*,13*R*,14*S*,17*R*)-10,13-Dimethyl-17-((*R*)-6-methylheptan-2-yl)hexadecahydro-1*H*-cyclopenta[a]phenanthren-3-yl 4-formylbenzoate (1z)

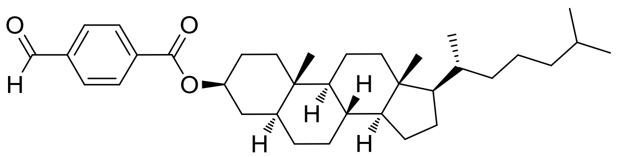

Mp: 132–133 °C; ^{1}H NMR (600 MHz, $CDCl_3$) δ 10.10 (s, 1H), 8.19 (d, J = 8.2 Hz, 2H), 7.94 (d, J = 8.2 Hz, 2H), 5.02–4.93 (m, 1H), 2.00–1.94 (m, 2H), 1.86–1.65 (m, 5H), 1.61–1.47 (m, 5H), 1.41–1.20 (m, 9H), 1.18–0.96 (m, 10H), 0.94–0.84 (m, 12H), 0.66 (s, 3H); ^{13}C NMR (151 MHz, $CDCl_3$) δ 191.7, 165.1, 138.9, 135.9, 130.1, 129.4, 75.2, 56.4, 56.2, 54.2, 44.7, 42.6, 40.0, 39.5, 36.7, 36.2, 35.8, 35.5, 35.5, 34.0, 32.0, 28.6, 28.2, 28.0, 27.5, 24.2, 23.8, 22.8, 22.6, 21.2, 18.7, 12.3, 12.1; IR (ATR): 2934, 2853, 2119, 1702, 1466, 1378, 1273, 1203,

1118, 762 cm^{-1}; MS (CI): m/z (%) 521.4 ([M + H]$^+$, 2), 520.6 (M$^+$, 1), 301.0 (11), 274.0 (100), 238.1 (12), 182.0 (44), 156.0 (42), 91.1 (15); HRMS (EI) for $C_{35}H_{52}O_3$: calcd. for [M]$^+$ 520.39110, found 520.39058.

(3*S*,8*S*,9*S*,10*R*,13*S*,14*S*,17*S*)-17-Acetyl-10,13-dimethyl-2,3,4,7,8,9,10,11,12,13,14,15,16,17-tetradecahydro-1*H*-cyclopenta[*a*]phenanthren-3-yl 4-formylbenzoate (1aa)

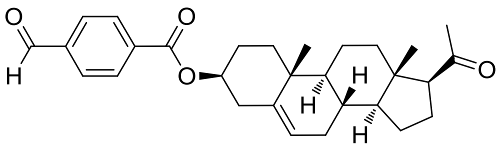

^{1}H NMR (600 MHz, $CDCl_3$) δ 10.11 (s, 1H), 8.20 (d, J = 8.2 Hz, 2H), 7.95 (d, J = 8.3 Hz, 2H), 5.44 (d, J = 4.3 Hz, 1H), 4.93–4.86 (m, 1H), 2.55 (t, J = 9.0 Hz, 1H), 2.49 (d, J = 7.7 Hz, 2H), 2.21–2.15 (m, 1H), 2.14 (s, 3H), 2.07–2.03 (m, 2H), 1.95 (dt, J = 13.4, 3.4 Hz, 1H), 1.78–1.60 (m, 6H), 1.52–1.45 (m, 3H), 1.27–1.17 (m, 4H), 1.08 (s, 3H), 0.65 (s, 3H); ^{13}C NMR (151 MHz, $CDCl_3$) δ 209.6, 191.7, 164.9, 139.4, 139.0, 135.7, 130.1, 129.5, 122.7, 75.2, 63.7, 56.8, 49.9, 44.0, 38.8, 38.1, 37.0, 36.6, 31.8, 31.7, 31.6, 27.8, 24.5, 22.8, 21.0, 19.4, 13.2; IR (ATR): 2940, 2884, 2084, 1704, 1446, 1382, 1272, 1198, 1108, 758 cm^{-1}; HRMS (EI) for $C_{29}H_{36}O_4$: calcd. for [M]$^+$ 448.26081, found 448.25919.

Supplementary Note 4. Synthesis and characterization of the boronic ester substrates

Substrates **2a-2h**, **2m-2q**, **2v-2aa** in Supplementary Figure 2 were prepared according to general method A.[3]

General Method A:

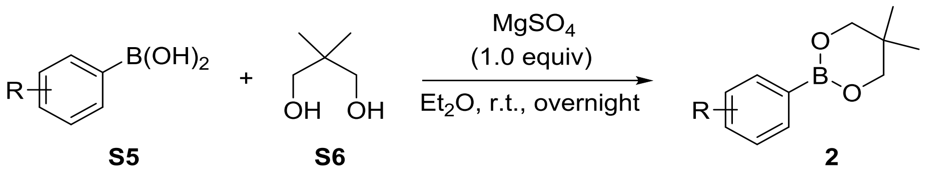

A 50 mL round-bottom flask was charged with a stir bar and $MgSO_4$ (602 mg, 5.0 mmol). The flask was further charged with the corresponding aryl boronic acid **S5** (5.0 mmol) and neopentyl glycol **S6** (521 mg, 5.0 mmol). Anhydrous Et_2O (15 mL) was then added and the resulting suspension stirred at room temperature overnight. After this time, the mixture was filtered through a celite pad and the residue washed with Et_2O (3x10 mL). The resulting mixture was concentrated in *vacuo* to afford the title aryl boronic ester as white solid or colorless oil without any further purification.

Substrates **2i-2l**, **2r-2u** in Supplementary Figure 2 were prepared according to general method B.[4]

General Method B:

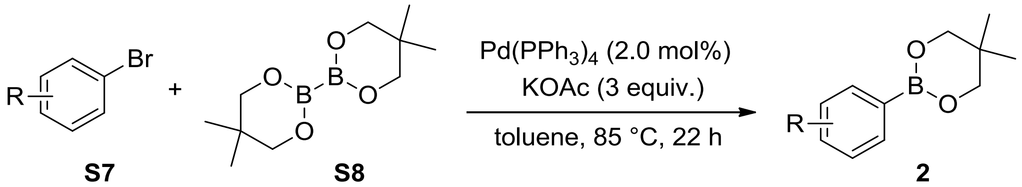

An oven-dried Schlenk flask was charged under nitrogen with aryl bromide **S7** (3.0 mmol), bis(neopentyl glycolato)diboron **S8** (814 mg, 3.6 mmol), $Pd(PPh_3)_4$ (69 mg, 2.0 mol%), potassium acetate (883 mg, 9.0 mmol) and dry toluene (around 15 mL). The Schlenk tube was sealed under nitrogen and placed in an oil bath preheated to 85 °C and the reaction mixture was stirred for 22 h at this temperature. After being cooled to room temperature, the reaction mixture was poured into water (30 mL), diluted with ethyl acetate (30 mL) and filtered through celite plugs. The mixture was extracted with ethyl acetate (3 × 20 mL) and the combined organic extracts were washed with brine, dried over Na_2SO_4, and then filtered. The filtrate was concentrated in *vacuo* and the residue was purified by flash column chromatography on silica gel to afford the corresponding aryl boronic ester **2**.

Substrates **2bb** and **2cc** in Supplementary Figure 2 were prepared according to general method C.

General Method C:

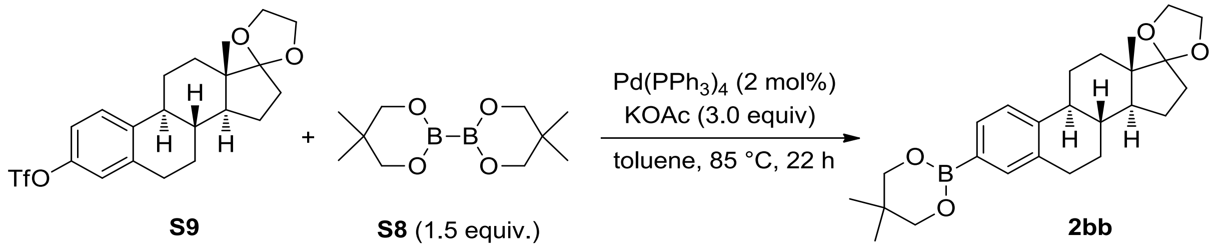

Compound **S9** was synthesized from estrone according to the reported method.[5]

An oven-dried Schlenk flask was charged under nitrogen with aryl triflate **S9** (1.03 g, 2.31 mmol), bis(neopentyl glycolato)diboron **S8** (783 mg, 3.47 mmol, 1.5 equiv.), $Pd(PPh_3)_4$ (53 mg, 2.0 mol%), potassium acetate (680 mg, 6.93 mmol, 3.0 equiv.) and dry toluene (around 20 mL). The Schlenk tube was sealed under nitrogen and placed in an oil bath preheated to 85 °C and the reaction mixture was stirred for 22 h at this temperature. After being cooled to room temperature, the reaction mixture was poured into water (30 mL), diluted with ethyl acetate (30 mL) and filtered through celite plugs. After the mixture was extracted with ethyl acetate (3 × 20 mL), the combined organic extracts were washed with brine, dried over Na_2SO_4, and then filtered. The filtrate was concentrated in *vacuo* and the residue was purified by flash column chromatography on silica gel (eluent: Hexane/EtOAc = 10:1) to afford **2bb** as light yellow solid (621 mg, 66%).

5,5-Dimethyl-2-((8*R*,9*S*,13*S*,14*S*)-13-methyl-6,7,8,9,11,12,13,14,15,16-decahydrospiro[cyclopenta[*a*]phenanthrene-17,2'-[1,3]dioxolan]-3-yl)-1,3,2-dioxaborinane (2bb)

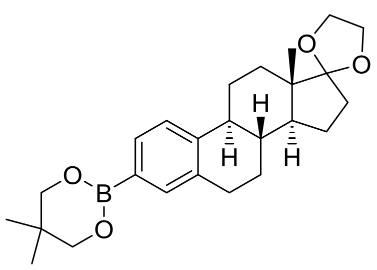

Mp: 141-142 °C; ^{1}H NMR (400 MHz, $CDCl_3$): δ 7.57 (d, J = 7.8 Hz, 1H), 7.53 (s, 1H), 7.30 (d, J = 7.8 Hz, 1H), 3.99–3.87 (m, 4H), 3.76 (s, 4H), 2.92–2.84 (m, 2H), 2.42–2.26 (m, 2H), 2.08–1.98 (m, 1H), 1.95–1.73 (m, 4H), 1.69–1.60 (m, 1H), 1.58–1.24 (m, 5H), 1.01 (s, 6H), 0.88 (s, 3H); ^{13}C NMR (100 MHz, $CDCl_3$): δ 143.2, 135.9, 134.8, 131.2, 124.7, 119.5, 115.1, 72.4, 65.3, 64.7, 49.6, 46.2, 44.5, 38.8, 34.3, 32.0, 30.9, 29.5, 27.1, 25.9, 22.5, 22.0, 14.4; IR (ATR): 3461, 2936, 2316, 2163, 1740, 1605, 1338, 1221, 692 cm^{-1}; MS (EI): m/z (%) 410.3 (M^+, 22), 348.2 (100), 99.1 (26); HRMS (ESI) for $C_{25}H_{35}BO_4K$: calculated for $[M+K]^+$ 449.22600, found 449.22638.

2-((*R*)-2,8-Dimethyl-2-((4*R*,8*R*)-4,8,12-trimethyltridecyl)chroman-6-yl)-5,5-dimethyl-1,3,2-dioxaborinane (2cc)

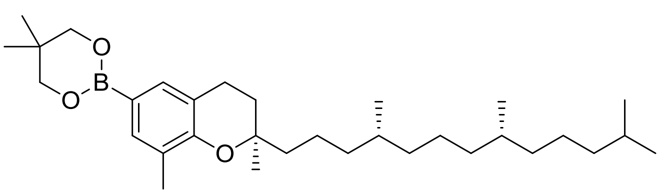

Following Method C, isolated as colorless oil. ^{1}H NMR (400 MHz, $CDCl_3$) δ 7.42 (d, J = 11.8 Hz, 2H), 3.77 (s, 4H), 2.79 (t, J = 6.6 Hz, 2H), 2.20 (s, 3H), 1.91–1.73 (m, 2H), 1.63–1.01 (m, 31H), 0.95–0.85 (m, 11H); ^{13}C NMR (101 MHz, $CDCl_3$) δ 154.7, 134.0, 133.1, 125.4, 119.6, 76.3, 72.2, 60.3, 40.2, 39.4, 37.4, 37.3, 32.8, 32.7, 31.9, 31.3, 28.0, 24.8, 24.5, 24.3, 22.7, 22.6, 22.2, 21.9, 21.0, 19.8, 19.7, 16.0, 14.2; IR (ATR): 3444, 3190, 2928, 2725, 1793, 1604, 1471, 1421, 1377, 1347, 1216, 1145, 1060, 980, 935, 872, 846, 740, 682, 611, 503 cm^{-1}; MS (EI): m/z (%) 498.3 (M^+, 100), 497.3 (31), 273.0 (27), 233.0 (59); HRMS (ESI) for $C_{32}H_{55}BO_3Na$: calculated for $[M+Na]^+$ 521.41365, found 521.41296.

Supplementary Note 5. General procedure for the deformylative coupling and spectroscopic data of the products

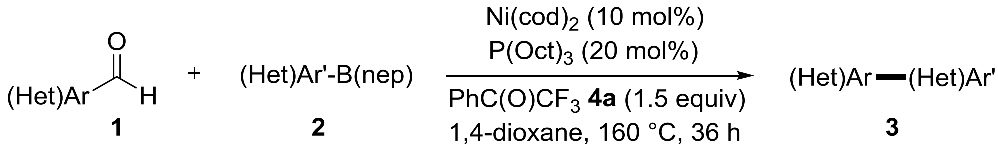

In a nitrogen-filled glovebox, a 10-mL oven-dried sealed tube containing a stirring bar was charged with the corresponding aldehyde **1** (0.20 mmol, 1.0 equiv.), aryl/heteroaryl boronic ester **2** (0.40 mmol, 2.0 equiv.) and yellow Ni(cod)$_2$ (5.5 mg, 10 mol%). Subsequently, HPLC grade 1,4-dioxane (1.5 mL) was added, and then trioctylphosphine ligand (18 μL, 20 mol%) and 2,2,2-trifluoroacetophenone (42 μL, 0.30 mmol, 1.5 equiv.) were added respectively via microsyringe. The tube with the mixture was sealed and removed from the glovebox. After stirring at 160 °C for 36 h, the mixture was allowed to cool to room temperature, diluted with EtOAc (5 mL) and filtered through a celite plug, eluting with additional EtOAc (15 mL). The filtrate was concentrated and purified by column chromatography on silica gel to yield the title product.

3-Phenylpyridine (3Aa)

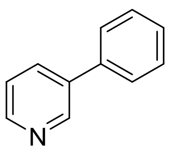

Following the general procedure, the title product was isolated as colorless oil after flash chromatography on silica gel. ^{1}H NMR (600 MHz, $CDCl_3$): δ 8.86 (d, J = 2.4 Hz, 1H), 8.59 (dd, J = 4.8, 1.7 Hz, 1H), 7.87 (dt, J = 7.9, 2.0 Hz, 1H), 7.61–7.55 (m, 2H), 7.51–7.45 (m, 2H), 7.43–7.39 (m, 1H), 7.38–7.33 (m, 1H); ^{13}C NMR (150 MHz, $CDCl_3$): δ 148.5, 148.3, 137.9, 136.7, 134.4, 129.1, 128.2, 127.2, 123.6; IR (ATR): 3031, 2340, 2112, 1992, 1581, 1472, 1450, 1406, 1336, 1187, 1024, 1004, 913, 813, 754, 703 cm^{-1}; MS (EI): m/z (%) 155.0 (M^+, 100), 154.0 (44), 127.0 (17), 102.0 (15); Data in accordance with the literature.[6]

3-(*p*-Tolyl)pyridine (3Ab)

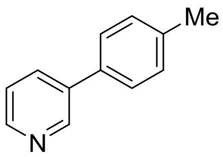

Following the general procedure, the title product was isolated as white solid after flash chromatography on silica gel. ^{1}H NMR (600 MHz, $CDCl_3$): δ 8.84 (s, 1H), 8.57 (d, J = 4.0 Hz, 1H), 7.86 (d, J = 7.9 Hz, 1H), 7.48 (d, J = 8.1 Hz, 2H), 7.35 (dd, J = 7.9, 4.8 Hz, 1H), 7.29 (d, J = 7.9 Hz, 2H), 2.41 (s, 3H); ^{13}C NMR (150 MHz, $CDCl_3$): δ 148.13, 148.11, 138.1, 136.7, 134.9, 134.3, 129.9, 127.0, 123.6, 21.2; IR (ATR): 3029, 2961, 2919, 2862, 2094, 1744, 1582, 1471, 1425, 1389, 1262, 1132, 1101, 1021, 795, 708 cm^{-1}; MS (EI): m/z (%) 169.0 (M^+, 100), 168.0 (62), 115.0 (16); Data in accordance with the literature.[6]

3-(4-(*Tert*-butyl)phenyl)pyridine (3Ac)

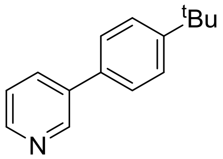

Following the general procedure, the title product was isolated as white solid after flash chromatography on silica gel. ^{1}H NMR (600 MHz, $CDCl_3$): δ 8.86 (s, 1H), 8.60–8.54 (m, 1H), 7.87 (dt, J = 7.8, 1.8 Hz, 1H), 7.56–7.48 (m, 4H), 7.35 (dd, J = 7.8, 4.8 Hz, 1H), 1.37 (s, 9H); ^{13}C NMR (150 MHz, $CDCl_3$): δ 151.3, 148.24, 148.19, 136.6, 134.9, 134.3, 126.9, 126.1, 123.6, 34.7, 31.4; IR (ATR): 3400, 3034, 2957, 2328, 2098, 1913, 1742, 1582, 1471, 1386, 1267, 1185, 1112, 1013, 803, 706 cm^{-1}; MS (EI): m/z (%) 211.0 (M^+, 57), 196.0 (100), 167.9 (18); Data in accordance with the literature.[6]

3-(3,5-Dimethylphenyl)pyridine (3Ad)

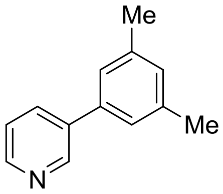

Following the general procedure, the title product was isolated as colorless oil after flash chromatography on silica gel. ^{1}H NMR (400 MHz, $CDCl_3$) δ 8.83 (s, 1H), 8.57 (s, 1H), 7.86 (d, J = 7.8 Hz, 1H), 7.34 (dd, J = 7.4, 5.0 Hz, 1H), 7.19 (s, 2H), 7.05 (s, 1H), 2.40 (s, 6H); ^{13}C NMR (101 MHz, $CDCl_3$) δ 148.3, 148.2, 138.6, 137.8, 136.9, 134.4, 129.7, 125.0, 123.4, 21.4; IR (ATR): 3435, 3027, 2920, 2858, 1904, 1734, 1604, 1465, 1394, 1329, 1186, 1104, 1026, 852, 806, 709, 621 cm^{-1}; MS (EI): m/z (%) 183.1 (M^+, 100), 182.0 (26), 168.0 (39), 167.0 (24); Data in accordance with the literature.[6]

3-([1,1'-Biphenyl]-4-yl)pyridine (3Ae)

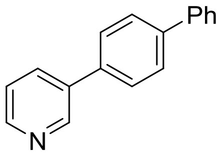

Following the general procedure, the title product was isolated as white solid after flash chromatography on silica gel. ^{1}H NMR (600 MHz, $CDCl_3$): δ 8.92 (s, 1H), 8.62 (d, J = 4.0 Hz, 1H), 7.92 (dt, J = 7.9, 1.9 Hz, 1H), 7.72 (d, J = 8.3 Hz, 2H), 7.69–7.63 (m, 4H), 7.48 (t, J = 7.7 Hz, 2H), 7.41–7.36 (m, 2H); ^{13}C NMR (150 MHz, $CDCl_3$): δ 148.6, 148.3, 141.0, 140.4, 136.7, 136.2, 134.2, 128.9, 127.8, 127.6, 127.5, 127.1, 123.6; IR (ATR): 3054, 2329, 2095, 1696, 1470, 1394, 1314, 1260, 1180, 1138, 999, 842, 805, 760, 691 cm^{-1}; MS (EI): m/z (%) 231.2 (M^+, 100), 220.1 (12), 192.1 (12); Data in accordance with the literature.[6]

3-(Naphthalen-2-yl)pyridine (3Af)

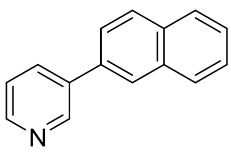

Following the general procedure, the title product was isolated as white solid after flash chromatography on silica gel. ^{1}H NMR (400 MHz, $CDCl_3$): δ 8.99 (s, 1H), 8.63 (d, J = 4.0 Hz, 1H), 8.04 (s, 1H), 8.02–7.85 (m, 4H), 7.71 (dd, J = 8.5, 1.8 Hz, 1H), 7.57–7.49 (m, 2H), 7.40 (dd, J = 7.8, 4.8 Hz, 1H); ^{13}C NMR (100 MHz, $CDCl_3$): 148.6, 148.5, 136.6, 135.1, 134.6, 133.6, 132.9, 128.9, 128.3, 127.8, 126.7, 126.5, 126.2, 125.1, 123.7; IR (ATR): 3049, 2089, 1656, 1583, 1480, 1411, 1283, 1187, 1127, 1020, 956, 865, 799, 757, 704 cm^{-1}; MS (EI): m/z (%) 205.1 (M^+, 100), 204.1 (38), 155.1 (29), 127.0 (16); Data in accordance with the literature.[6]

3-(Naphthalen-1-yl)pyridine (3Ag)

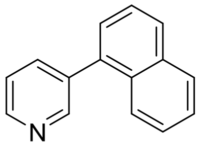

Following the general procedure, the title product was isolated as white solid after flash chromatography on silica gel. ^{1}H NMR (400 MHz, $CDCl_3$): δ 8.99 (s, 1H), 8.64 (s, 1H), 8.05 (d, J = 1.4 Hz, 1H), 8.03–7.86 (m, 4H), 7.71 (dd, J = 8.5, 1.8 Hz, 1H), 7.57–7.49 (m, 2H), 7.41 (dd, J = 7.8, 4.8 Hz, 1H); ^{13}C NMR (100 MHz, $CDCl_3$): δ 148.7, 148.6, 136.7, 135.2, 134.6, 133.7, 133.0, 129.0, 128.3, 127.8, 126.7, 126.5, 126.2, 125.1, 123.7; IR (ATR): 3046, 2089, 1926, 1657, 1576, 1478, 1411, 1283, 1187, 1128, 1019, 865, 799, 757, 704 cm^{-1}; MS (EI): m/z (%) 205.2 (M^+, 100), 204.2 (39); Data in accordance with the literature.[6]

3-(4-(Naphthalen-2-yl)phenyl)pyridine (3Ah)

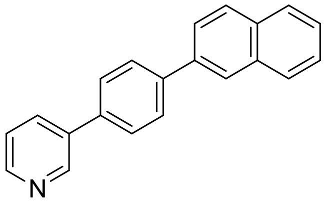

Following the general procedure, the title product was isolated as colorless oil after flash chromatography on silica gel. ^{1}H NMR (600 MHz, $CDCl_3$) δ 8.95 (s, 1H), 8.64 (s, 1H), 8.10 (s, 1H), 7.94 (dd, J = 15.1, 8.2 Hz, 3H), 7.89 (d, J = 7.7 Hz, 1H), 7.85 (d, J = 8.2 Hz, 2H), 7.80 (d, J = 8.5 Hz, 1H), 7.72 (d, J = 8.2 Hz, 2H), 7.56–7.49 (m, 2H), 7.41 (brs, 1H); ^{13}C NMR (151 MHz, $CDCl_3$) δ 148.3, 148.1, 140.9, 137.6, 136.7, 136.3, 134.4, 133.6, 132.7, 128.6, 128.2, 128.1, 127.7, 127.6, 126.4, 126.1, 125.8, 125.3, 123.7; IR (ATR): 3049, 2925, 2311, 2116, 1920, 1580, 1472, 1397, 1128, 1018, 996, 950, 897, 798, 750, 702 cm^{-1}; MS (EI): m/z (%) 281.1 (M^+, 100), 278.1 (10), 252.0 (7), 202.1 (8), 140.4 (8); HRMS (EI) for $C_{21}H_{16}N$: calcd. for $[M+H]^+$ 282.12773, found 282.12732.

3-(Benzo[*d*][1,3]dioxol-5-yl)pyridine (3Ai)

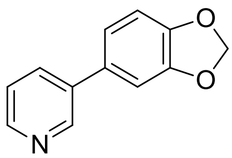

Following the general procedure, the title product was isolated as white solid after flash chromatography on silica gel. ^{1}H NMR (400 MHz, $CDCl_3$) δ 8.78 (s, 1H), 8.55 (s, 1H), 7.81 (d, J = 7.9 Hz, 1H), 7.34 (dd, J = 7.7, 4.9 Hz, 1H), 7.11–7.00 (m, 2H), 6.91 (d, J = 8.5 Hz, 1H), 6.01 (s, 2H); ^{13}C NMR (101 MHz, $CDCl_3$) δ 148.4, 147.8, 147.7, 136.5, 134.3, 131.8, 123.6, 120.8, 108.9, 107.5, 101.3; IR (ATR): 3412, 2923, 1712, 1606, 1510, 1477, 1421, 1363, 1258, 1167, 1113, 1036, 930, 869, 804, 708, 621, 562, 530 cm^{-1}; MS (EI): m/z (%) 199.0 (M^+, 100), 198.0 (59), 140.0 (23), 114.0 (36), 88.1 (21), 63.1 (27); Data in accordance with the literature.[7]

3-(4-Ethoxyphenyl)pyridine (3Aj)

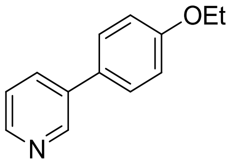

Following the general procedure, the title product was isolated as white solid after flash chromatography on silica gel. ^1H NMR (400 MHz, $CDCl_3$) δ 8.81 (s, 1H), 8.53 (s, 1H), 7.81 (d, J = 7.9 Hz, 1H), 7.54 – 7.43 (m, 2H), 7.31 (dd, J = 7.6, 4.8 Hz, 1H), 7.03 – 6.89 (m, 2H), 4.06 (q, J = 7.0 Hz, 2H), 1.42 (t, J = 7.0 Hz, 3H); ^{13}C NMR (101 MHz, $CDCl_3$) δ 159.1, 147.8, 147.7, 136.3, 133.8, 130.0, 128.2, 123.5, 115.1, 63.5, 14.8; IR (ATR): 3037, 2978, 2927, 2531, 1894, 1604, 1514, 1471, 1286, 1243, 1178, 1115, 1042, 920, 802, 708 cm^{-1}; MS (EI): m/z (%) 199.1 (M^+, 100), 171.0 (100), 142.0 (17), 115.1 (18); Data in accordance with the literature.[8]

3-(4-(Trimethylsilyl)phenyl)pyridine (3Ak)

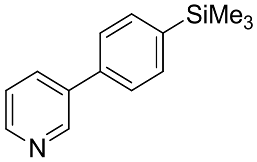

Following the general procedure, the title product was isolated as colorless oil after flash chromatography on silica gel. ^1H NMR (400 MHz, $CDCl_3$) δ 8.85 (s, 1H), 8.58 (d, J = 4.1 Hz, 1H), 7.86 (d, J = 7.9 Hz, 1H), 7.63 (d, J = 8.1 Hz, 2H), 7.56 (d, J = 8.0 Hz, 2H), 7.34 (dd, J = 7.8, 4.8 Hz, 1H), 0.30 (s, 9H); ^{13}C NMR (101 MHz, $CDCl_3$) δ 148.4, 148.3, 140.4, 138.1, 136.6, 134.3, 134.1, 126.4, 123.5, -1.17; IR (ATR): $\tilde{\nu}$ 3466, 3023, 2956, 1745, 1663, 1593, 1474, 1417, 1381, 1315, 1251, 1184, 1106, 1024, 1000, 845, 799, 758, 711, 556 cm^{-1}; MS (EI): m/z (%) 227.1 (M^+, 60), 212.1 (100), 182.0 (11), 106.0 (13); HRMS (EI) for $C_{14}H_{18}NSi$: calcd. for $[M+H]^+$ 228.12030, found 228.11975.

***N,N*-dimethyl-4-(pyridin-3-yl)aniline (3Al)**

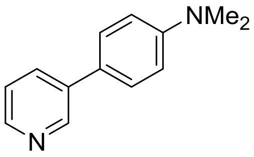

Following the general procedure, the title product was isolated as white solid after flash chromatography on silica gel. ^1H NMR (400 MHz, $CDCl_3$): δ 8.83 (s, 1H), 8.49 (d, J = 4.5 Hz, 1H), 7.82 (dt, J = 8.2, 1.8 Hz, 1H), 7.49 (d, J = 8.8 Hz, 2H), 7.29 (dd, J = 7.8, 4.8 Hz, 1H), 6.82 (d, J = 8.8 Hz, 2H), 3.00 (s, 6H); ^{13}C NMR (100 MHz, $CDCl_3$): δ 150.4, 147.7, 147.2, 136.6, 133.2, 127.7, 125.4, 123.5, 112.8, 40.5; IR (ATR): 2905, 2811, 2314, 2108, 1884, 1602, 1525, 1474, 1438, 1350, 1225, 941, 796, 706 cm^{-1}; MS (EI): m/z (%) 198.2 (M^+, 100), 197.1 (68); Data in accordance with the literature.[9]

3-(4-Fluorophenyl)pyridine (3Am)

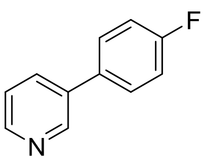

Following the general procedure, the title product was isolated as white solid after flash chromatography on silica gel. ^{1}H NMR (600 MHz, $CDCl_3$): δ 8.80 (s, 1H), 8.58 (d, J = 4.4 Hz, 1H), 7.82 (dt, J = 7.9, 1.9 Hz, 1H), 7.56–7.50 (m, 2H), 7.35 (dd, J = 7.9, 4.8 Hz, 1H), 7.19–7.14 (m, 2H); ^{13}C NMR (150 MHz, $CDCl_3$): δ 163.0 (d, J_{C-F} = 247.5 Hz), 148.5, 148.1, 135.8, 134.3, 134.0 (d, J_{C-F} = 3.2 Hz), 128.9 (d, J_{C-F} = 8.5 Hz), 123.6, 116.1 (d, J_{C-F} = 22.1 Hz); ^{19}F NMR (564 MHz, $CDCl_3$): δ - 114.2; IR (ATR): 3031, 2341, 2092, 1892, 1743, 1595, 1476, 1388, 1223, 1003, 812, 712 cm^{-1}; MS (EI): m/z (%) 173.9 (M^+, 13), 172.9 (100), 171.9 (34), 145.9 (8), 120.0 (8); Data in accordance with the literature.[6]

3-(2-Fluorophenyl)pyridine (3An)

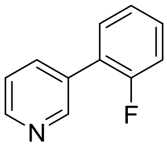

Following the general procedure, the title product was isolated as white solid after flash chromatography on silica gel. ^{1}H NMR (400 MHz, $CDCl_3$): δ 8.78 (s, 1H), 8.59 (s, 1H), 7.85 (d, J = 7.9 Hz, 1H), 7.45–7.30 (m, 3H), 7.26–7.12 (m, 2H); ^{13}C NMR (100 MHz, $CDCl_3$): δ 159.9 (d, J_{C-F} = 248.6 Hz), 149.7, 148.8, 136.3 (d, J_{C-F} = 3.2 Hz), 131.7, 130.5 (d, J_{C-F} = 3.2 Hz), 130.0 (d, J_{C-F} = 8.3 Hz), 125.7 (d, J_{C-F} = 13.5 Hz), 124.7 (d, J_{C-F} = 3.7 Hz), 123.3, 116.3 (d, J_{C-F} = 22.2 Hz); ^{19}F NMR (376 MHz, $CDCl_3$): δ - 118.0; IR (ATR): 3034, 2322, 2093, 1895, 1743, 1453, 1216, 1107, 1007, 742 cm^{-1}; MS (EI): m/z (%) 173.9 (M^+, 17), 172.9 (100), 171.9 (32), 145.9 (7), 119.9 (9); Data in accordance with the literature.[10]

3-(3,5-Difluorophenyl)pyridine (3Ao)

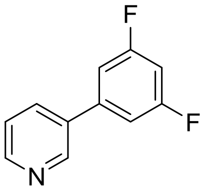

Following the general procedure, the title product was isolated as white solid after flash chromatography on silica gel. Mp: 47-49 °C; ^{1}H NMR (600 MHz, $CDCl_3$): δ 8.84 (s, 1H), 8.67 (s, 1H), 7.84 (d, J = 7.9 Hz, 1H), 7.43–7.37 (m, 1H), 7.13–7.07 (m, 2H), 6.85 (tt, J = 8.8, 2.3 Hz, 1H); ^{13}C NMR (150 MHz, $CDCl_3$): δ 163.6 (d, J_{C-F} = 249.3 Hz), 163.5 (d, J_{C-F} = 249.2 Hz), 149.7, 148.1, 141.2 (t, J_{C-F} = 9.6 Hz), 134.6, 134.4, 123.9, 110.19 (d, J_{C-F} = 20.4 Hz), 110.15 (d, J_{C-F} = 20.3 Hz), 103.5 (t, J_{C-F} = 25.3 Hz); ^{19}F NMR (564 MHz, $CDCl_3$): δ - 108.8; IR (ATR): 3087, 3044, 2095, 1730, 1620, 1596, 1451, 1399, 1340, 1216, 1111, 984, 861, 830, 803, 706, 681 cm^{-1}; MS (EI): m/z (%) 191.1 (M^+, 100), 190.1 (36), 164.0 (12), 138.0 (13); HRMS (EI) for $C_{11}H_8F_2N$: calcd. for $[M+H]^+$ 192.06193, found 192.06195.

3-(3-(Trifluoromethyl)phenyl)pyridine (3Ap)

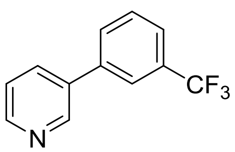

Following the general procedure, the title product was isolated as white solid after flash chromatography on silica gel. ^{1}H NMR (400 MHz, $CDCl_3$): δ 8.86 (s, 1H), 8.65 (d, J = 3.3 Hz, 1H), 7.91–7.85 (m, 1H), 7.81 (s, 1H), 7.76 (d, J = 7.7 Hz, 1H), 7.66 (d, J = 7.7 Hz, 1H), 7.60 (t, J = 7.7 Hz, 1H), 7.40 (dd, J = 7.9, 4.8 Hz, 1H); ^{13}C NMR (100 MHz, $CDCl_3$): δ 149.3, 148.3, 138.8, 135.4, 134.5, 131.6 (q, J_{C-F} = 32.5 Hz), 130.5, 129.7, 124.9 (q, J_{C-F} = 3.6 Hz), 124.03 (q, J_{C-F} = 272.4 Hz), 124.02 (q, J_{C-F} = 3.6 Hz), 123.8; ^{19}F NMR (564 MHz, $CDCl_3$): δ - 62.7; IR (ATR): 3044, 2928, 2109, 1793, 1668, 1586, 1438, 1403, 1333, 1267, 1165, 1123, 1075, 1023, 901, 795, 706 cm^{-1}; MS (EI): m/z (%) 223.1 (M^+, 100), 105.1 (54); Data in accordance with the literature.[6]

3-(3,5-Bis(trifluoromethyl)phenyl)pyridine (3Aq)

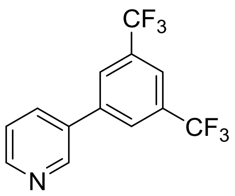

Following the general procedure, the title product was isolated as white solid after flash chromatography on silica gel. ^{1}H NMR (600 MHz, $CDCl_3$): δ 8.89 (s, 1H), 8.72 (s, 1H), 8.01 (s, 2H), 7.95–7.91 (m, 2H), 7.46 (dd, J = 7.8, 4.8 Hz, 1H); ^{13}C NMR (150 MHz, $CDCl_3$): δ 150.1, 148.2, 140.1, 134.7, 134.1, 132.7 (q, J_{C-F} = 33.3 Hz), 127.4 (q, J_{C-F} = 3.6 Hz), 124.0, 123.2 (q, J_{C-F} = 272.9 Hz), 121.9 (q, J_{C-F} = 3.8 Hz); ^{19}F NMR (564 MHz, $CDCl_3$): δ - 62.9; IR (ATR): 3044, 2931, 2111, 1745, 1590, 1458, 1375, 1276, 1116, 1030, 894, 804, 706, 681 cm^{-1}; MS (EI): m/z (%) 291.1 (M^+, 13), 261.1 (13), 109.1 (32), 106.0 (100), 78.1 (41), 51.2 (39); Data in accordance with the literature.[11]

3-(4-(Trifluoromethoxy)phenyl)pyridine (3Ar)

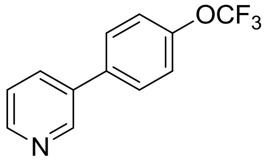

Following the general procedure, the title product was isolated as colorless oil after flash chromatography on silica gel. ^{1}H NMR (400 MHz, $CDCl_3$) δ 8.82 (d, J = 1.8 Hz, 1H), 8.62 (d, J = 4.7 Hz, 1H), 7.87–7.82 (m, 1H), 7.63–7.55 (m, 2H), 7.38 (dd, J = 7.9, 4.8 Hz, 1H), 7.33 (d, J = 8.3 Hz, 2H); ^{13}C NMR (101 MHz, $CDCl_3$) δ 149.2, 148.8, 148.2, 136.5, 135.3, 134.3, 128.5, 123.6, 121.5, 120.4 (q, J_{C-F} = 257.5 Hz); ^{19}F NMR (376 MHz, $CDCl_3$) δ -57.87 (s); IR (ATR): 3433, 3044, 2928, 2474, 1746, 1668, 1590, 1513, 1476, 1429, 1385, 1259, 1164, 1023, 1004, 853, 803, 710, 556 cm^{-1}; MS (EI): m/z (%) 239.3 (M^+, 1), 142.1 (15), 114.0 (52), 105.0 (61), 77.1 (100), 72.2 (98); Data in accordance with the literature.[12]

3-(Pyridin-3-yl)benzonitrile (3As)

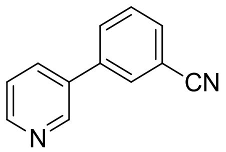

Following the general procedure, the title product was isolated as white solid after flash chromatography on silica gel. ^1H NMR (400 MHz, $CDCl_3$) δ 8.84 (s, 1H), 8.67 (s, 1H), 7.90–7.78 (m, 3H), 7.70 (d, J = 7.7 Hz, 1H), 7.60 (t, J = 7.8 Hz, 1H), 7.42 (dd, J = 7.6, 4.5 Hz, 1H); ^{13}C NMR (101 MHz, $CDCl_3$) δ 149.5, 148.0, 139.1, 134.5, 134.4, 131.5, 131.4, 130.7, 130.0, 123.8, 118.4, 113.4; IR (ATR): 3364, 2958, 2872, 2231, 1706, 1667, 1589, 1473, 1429, 1393, 1316, 1215, 1186, 1048, 898, 796, 705, 625 cm^{-1}; MS (EI): m/z (%) 180.1 (M^+, 100), 179.0 (42), 153.0 (12), 127.0 (11); Data in accordance with the literature.[13]

Methyl 4-(pyridin-3-yl)benzoate (3At)

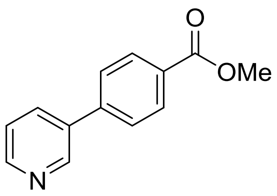

Following the general procedure, the title product was isolated as white solid after flash chromatography on silica gel. ^1H NMR (400 MHz, $CDCl_3$): δ 8.87 (s, 1H), 8.62 (d, J = 3.1 Hz, 1H), 8.15–8.09 (m, 2H), 7.89 (dt, J = 7.9, 1.9 Hz, 1H), 7.66–7.60 (m, 2H), 7.37 (dd, J = 7.9, 4.8 Hz, 1H), 3.93 (s, 3H); ^{13}C NMR (100 MHz, $CDCl_3$): δ 166.7, 149.3, 148.4, 142.3, 135.6, 134.5, 130.4, 129.8, 127.1, 123.7, 52.3; IR (ATR): 2940, 2320, 2102, 1862, 1715, 1602, 1429, 1270, 1194, 1104, 1002, 869, 810, 763, 703 cm^{-1}; MS (EI): m/z (%) 213.1 (M^+, 89), 182.1 (100), 154.1 (29), 127.1 (17); Data in accordance with the literature.[11]

***N,N*-diethyl-4-(pyridin-3-yl)benzamide (3Au)**

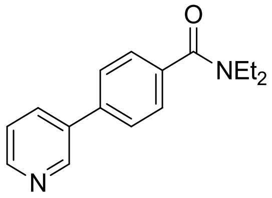

Following the general procedure, the title product was isolated as colorless oil after flash chromatography on silica gel. ^1H NMR (400 MHz, $CDCl_3$) δ 8.83 (s, 1H), 8.59 (s, 1H), 7.84 (dd, J = 12.5, 7.2 Hz, 1H), 7.59 (d, J = 8.3 Hz, 2H), 7.48 (t, J = 9.3 Hz, 2H), 7.36 (dd, J = 7.8, 4.8 Hz, 1H), 3.43 (t, J = 51.4 Hz, 4H), 1.37–1.04 (m, 6H); ^{13}C NMR (101 MHz, $CDCl_3$) δ 170.7, 148.7, 148.1, 138.5, 136.9, 135.9, 134.4, 127.1, 127.1, 123.6, 43.3, 39.3, 14.2, 12.9; IR (ATR): 3423, 3071, 1721, 1598, 1452, 1335, 1184, 1148, 940, 750, 716, 677, 602, 526 cm^{-1}; MS (EI): m/z (%) 254.1 (M^+, 40), 253.1 (59), 182.0 (100), 154.0 (24), 127.0 (21); HRMS (EI) for $C_{16}H_{19}ON_2$: calcd. for $[M+H]^+$ 255.14919, found 255.14848.

3-(Benzofuran-2-yl)pyridine (3Av)

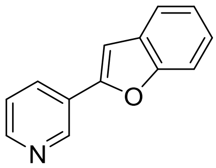

Following the general procedure, the title product was isolated as white solid after flash chromatography on silica gel. ^{1}H NMR (400 MHz, $CDCl_3$): δ 9.10 (s, 1H), 8.56 (d, J = 3.9 Hz, 1H), 8.10 (dt, J = 8.1, 1.8 Hz, 1H), 7.59 (d, J = 7.6 Hz, 1H), 7.52 (d, J = 8.1 Hz, 1H), 7.39–7.21 (m, 3H), 7.09 (s, 1H); ^{13}C NMR (100 MHz, $CDCl_3$): δ 155.1, 152.9, 149.3, 146.4, 131.9, 128.8, 126.7, 125.0, 123.7, 123.3, 121.3, 111.4, 102.8; IR (ATR): 3009, 2155, 1906, 1740, 1579, 1368, 1216, 1011, 916, 733 cm^{-1}; MS (EI): m/z (%) 195.9 (M^+, 17), 194.8 (100), 165.9 (15), 138.9 (14); Data in accordance with the literature.[14]

3-(Benzo[*b*]thiophen-2-yl)pyridine (3Aw)

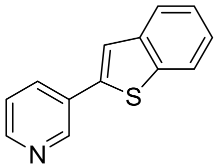

Following the general procedure, the title product was isolated as white solid after flash chromatography on silica gel. ^{1}H NMR (400 MHz, $CDCl_3$): δ 8.99 (s, 1H), 8.58 (s, 1H), 7.95 (d, J = 7.9 Hz, 1H), 7.87–7.83 (m, 1H), 7.80 (dd, J = 7.1, 2.0 Hz, 1H), 7.59 (s, 1H), 7.41–7.31 (m, 3H); ^{13}C NMR (100 MHz, $CDCl_3$): δ 149.1, 147.4, 140.4, 140.3, 139.8, 133.6, 130.4, 124.93, 124.85, 123.9, 123.7, 122.4, 120.8; IR (ATR): 3007, 2178, 1740, 1580, 1371, 1215, 1011, 915, 741 cm^{-1}; MS (EI): m/z (%) 211.9 (M^+, 22), 210.8 (100), 209.9 (29); Data in accordance with the literature.[14]

3-(Furan-2-yl)pyridine (3Ax)

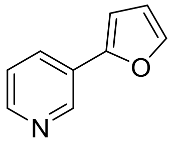

Following the general procedure, the title product was isolated as colorless oil after flash chromatography on silica gel. 1H NMR (400 MHz, $CDCl_3$) δ 8.90 (s, 1H), 8.46 (d, J = 4.0 Hz, 1H), 7.89 (dt, J = 8.0, 1.9 Hz, 1H), 7.48 (d, J = 1.2 Hz, 1H), 7.34–7.18 (m, 1H), 6.71 (d, J = 3.1 Hz, 1H), 6.47 (dd, J = 3.4, 1.8 Hz, 1H); ^{13}C NMR (101 MHz, $CDCl_3$) δ 151.0, 148.2, 145.4, 143.0, 130.7, 126.8, 123.4, 111.8, 106.3; IR (ATR): 3438, 3116, 2925, 2856, 1902, 1739, 1588, 1500, 1464, 1420, 1376, 1335, 1265, 1183, 1159, 1129, 1016, 905, 801, 740, 706, 593 cm^{-1}; MS (EI): m/z (%) 145.2 (M^+, 32), 121.2 (60), 106.1 (100), 78.3 (27), 51.2 (30); Data in accordance with the literature.[15]

3-(Furan-3-yl)pyridine (3Ay)

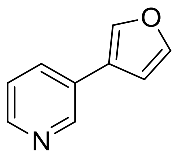

Following the general procedure, the title product was isolated as colorless oil after flash chromatography on silica gel. ^{1}H NMR (400 MHz, $CDCl_3$) δ 8.75 (s, 1H), 8.48 (s, 1H), 7.78–7.69 (m, 2H), 7.49 (t, J = 1.5 Hz, 1H), 7.36–7.20 (m, 1H), 6.74–6.64 (m, 1H); ^{13}C NMR (101 MHz, $CDCl_3$) δ 148.1, 147.1, 144.1, 138.9, 133.0, 128.4, 123.6, 123.2, 108.4; IR (ATR): 3441, 3132, 3028, 2926, 2855, 1732, 1652, 1584, 1509, 1472, 1418, 1382, 1327, 1161, 1063, 1023, 923, 874, 786, 736, 707, 598 cm^{-1}; MS (EI): m/z (%) 145.0 (M^+, 19), 105.0 (100), 77.2 (84), 51.2 (34); Data in accordance with the literature.[16]

3-(Thiophen-2-yl)pyridine (3Az)

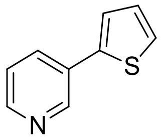

Following the general procedure, the title product was isolated as colorless oil after flash chromatography on silica gel. ^{1}H NMR (400 MHz, $CDCl_3$) δ 8.86 (s, 1H), 8.51 (d, J = 3.8 Hz, 1H), 7.84 (d, J = 7.9 Hz, 1H), 7.53–7.47 (m, 1H), 7.42 (dd, J = 4.9, 3.0 Hz, 1H), 7.37 (dd, J = 5.0, 1.0 Hz, 1H), 7.30 (dd, J = 7.8, 4.9 Hz, 1H); ^{13}C NMR (101 MHz, $CDCl_3$) δ 148.2, 147.6, 138.8, 133.5, 131.5, 127.0, 125.9, 123.6, 121.4; IR (ATR): 3076, 3031, 2925, 2856, 2328, 2084, 1905, 1649, 1575, 1515, 1473, 1420, 1281, 1218, 1183, 1125, 1020, 953, 892, 860, 783, 700 cm^{-1}; MS (EI): m/z (%) 161.0 (M^+, 100), 133.9 (10), 117.0 (30), 89.1 (10), 63.1 (9); Data in accordance with the literature.[14]

3-(Thiophen-3-yl)pyridine (3Aaa)

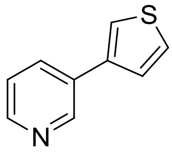

Following the general procedure, the title product was isolated as colorless oil after flash chromatography on silica gel. ^{1}H NMR (400 MHz, $CDCl_3$) δ 8.88 (s, 1H), 8.51 (d, J = 4.1 Hz, 1H), 7.90–7.82 (m, 1H), 7.40–7.33 (m, 2H), 7.30 (dd, J = 7.8, 4.9 Hz, 1H), 7.12 (dd, J = 4.8, 3.9 Hz, 1H); ^{13}C NMR (101 MHz, $CDCl_3$) δ 148.4, 146.9, 140.3, 133.0, 130.4, 128.3, 126.0, 124.2, 123.6; IR (ATR): 3417, 2959, 2930, 1741, 1638, 1591, 1477, 1421, 1351, 1283, 1183, 1131, 1108, 1026, 957, 850, 803, 704, 633 cm^{-1}; MS (EI): m/z (%) 161.0 (M^+, 10), 106.0 (100), 78.1 (40), 51.0 (38); Data in accordance with the literature.[14]

3-((8*R*,9*S*,13*S*,14*S*)-13-Methyl-6,7,8,9,11,12,13,14,15,16-decahydrospiro[cyclopenta[*a*]phenanthrene-17,2'-[1,3]dioxolan]-3-yl)pyridine (3Abb)

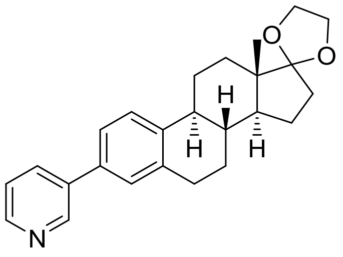

According to the typical procedure for decarbonylative cross-coupling reaction, the title product was isolated as colorless oil after flash chromatography on silica gel. ^{1}H NMR (600 MHz, $CDCl_3$): δ 8.84 (s, 1H), 8.56 (s, 1H), 7.85 (d, J = 7.8 Hz, 1H), 7.41 (d, J = 8.1 Hz, 1H), 7.38–7.28 (m, 3H), 4.00–3.87 (m, 4H), 2.97–2.92 (m, 2H), 2.42–2.30 (m, 2H), 2.08–2.01 (m, 1H), 1.99–1.92 (m, 1H), 1.90–1.76 (m, 3H), 1.71–1.64 (m, 1H), 1.61–1.33 (m, 5H), 0.90 (s, 3H); ^{13}C NMR (150 MHz, $CDCl_3$): δ 148.2, 148.1, 140.7, 137.7, 136.7, 135.1, 134.2, 127.7, 126.2, 124.4, 123.6, 119.4, 65.3, 64.6, 49.5, 46.2, 44.1, 38.9, 34.3, 30.8, 29.7, 27.0, 26.0, 22.4, 14.4; IR (ATR): 2918, 2248, 1891, 1742, 1583, 1458, 1307, 1051, 911, 726 cm^{-1}; MS (EI): m/z (%) 375.4 (M^+, 30), 314.3 (28), 313.3 (100), 99.1 (74); HRMS (ESI) for $C_{25}H_{30}NO_2$: calcd. for $[M+H]^+$ 376.22711, found 376.22726.

3-((*R*)-2,8-dimethyl-2-((4*R*,8*R*)-4,8,12-trimethyltridecyl)chroman-6-yl)pyridine (3Acc)

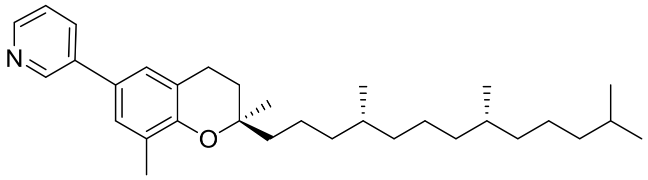

Following the general procedure, the title product was isolated as colorless oil after flash chromatography on silica gel. 1H NMR (400 MHz, $CDCl_3$) δ 8.80 (s, 1H), 8.50 (s, 1H), 7.82 (d, J = 7.9 Hz, 1H), 7.31 (dd, J = 7.7, 4.9 Hz, 1H), 7.19 (s, 1H), 7.14 (s, 1H), 2.87–2.78 (m, 2H), 2.24 (s, 3H), 1.90–1.76 (m, 2H), 1.63–1.04 (m, 25H), 0.89–0.81 (m, 11H); ^{13}C NMR (101 MHz, $CDCl_3$) δ 152.6, 147.7, 147.2, 136.8, 133.9, 128.2, 127.1, 127.0, 125.6, 123.4, 121.1, 40.2, 39.3, 37.4, 37.3, 32.8, 32.7, 31.1, 28.0, 24.8, 24.4, 24.3, 22.7, 22.6, 22.4, 21.1, 19.7, 19.6, 16.2; IR (ATR): 3440, 3028, 2927, 1745, 1593, 1465, 1377, 1348, 1261, 1185, 1136, 1103, 1023, 938, 876, 805, 737, 708, 613, 526 cm^{-1}; MS (EI): m/z (%) 463.4 (M^+, 100), 238.1 (36), 199.1 (70), 198.0 (81); HRMS (ESI) for $C_{32}H_{50}NO$: calcd. for $[M+H]^+$ 464.38869, found 464.38751.

1,1'-Biphenyl (3Ba)

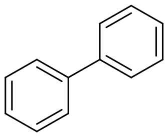

Following the general procedure, the title product was isolated as white solid after flash chromatography on silica gel. ^{1}H NMR (600 MHz, $CDCl_3$) δ 7.61 (d, J = 7.3 Hz, 4H), 7.45 (t, J = 7.7 Hz, 4H), 7.36 (t, J = 7.4 Hz, 2H); ^{13}C NMR (151 MHz, $CDCl_3$) δ 141.2,

128.7, 127.2, 127.2; IR (ATR): 3033, 1660, 1476, 1428, 727, 693 cm^{-1}; MS (EI): m/z (%) 154.4 (M^+, 3), 124.0 (19), 95.0 (29), 73.2 (35); Data in accordance with the literature.[17]

2-Phenylnaphthalene (3Ca)

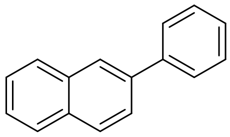

Following the general procedure, the title product was isolated as white solid after flash chromatography on silica gel. ^1H NMR (600 MHz, $CDCl_3$): δ 8.06 (s, 1H), 7.96–7.86 (m, 3H), 7.79–7.72 (m, 3H), 7.55–7.48 (m, 4H), 7.40 (t, J = 7.4 Hz, 1H); ^{13}C NMR (150 MHz, $CDCl_3$): δ 141.2, 138.6, 133.7, 132.7, 128.9, 128.5, 128.3, 127.7, 127.5, 127.4, 126.4, 126.0, 125.9, 125.7; IR (ATR): 3055, 2112, 1943, 1595, 1490, 1453, 1360, 1274, 1207, 1161, 1128, 1075, 1017, 949, 892, 857, 820, 753, 686 cm^{-1}; MS (EI): m/z (%) 204.0 (M^+, 100), 202.0 (31); Data in accordance with the literature.[18]

9-Phenylphenanthrene (3Da)

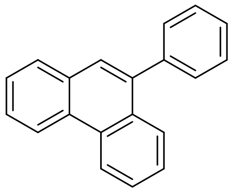

Following the general procedure, the title product was isolated as white solid after flash chromatography on silica gel. ^1H NMR (600 MHz, $CDCl_3$): δ 8.80 (d, J = 8.3 Hz, 1H), 8.74 (d, J = 8.3 Hz, 1H), 7.93 (dd, J = 16.0, 8.0 Hz, 2H), 7.73–7.66 (m, 3H), 7.63 (t, J = 7.4 Hz, 1H), 7.59–7.50 (m, 5H), 7.50–7.45 (m, 1H); ^{13}C NMR (150 MHz, $CDCl_3$): δ 140.9, 138.8, 131.6, 131.2, 130.7, 130.1, 130.0, 128.7, 128.4, 127.6, 127.4, 127.0, 126.9, 126.7, 126.6, 126.5, 123.0, 122.6; IR (ATR): 3071, 3023, 2327, 2106, 1799, 1592, 1487, 1444, 1258, 1125, 1071, 886, 857, 764, 720, 696 cm^{-1}; MS (EI): m/z (%) 254.1 (M^+, 100), 253.1 (43), 126.2 (7); Data in accordance with the literature.[18]

1,1':4',1''-Terphenyl (3Ea)

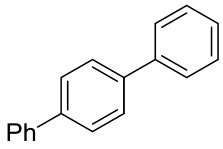

Following the general procedure, the title product was isolated as white solid after flash chromatography on silica gel. ^1H NMR (600 MHz, $CDCl_3$): δ 7.70 (s, 4H), 7.69–7.65 (m, 4H), 7.51–7.46 (m, 4H), 7.41–7.36 (m, 2H); ^{13}C NMR (150 MHz, $CDCl_3$): δ 140.8, 140.2, 128.9, 127.6, 127.4, 127.1; IR (ATR): 3033, 2327, 2105, 1940, 1577, 1468, 1400, 998, 836, 738, 682 cm^{-1}; MS (EI): m/z (%) 230.9 (M^+, 20), 229.9 (100), 227.9 (11); Data in accordance with the literature.[18]

2-Phenyl-9*H*-fluorene (3Fa)

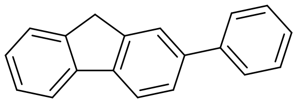

Following the general procedure, the title product was isolated as white solid after flash chromatography on silica gel. ^1H NMR (600 MHz, $CDCl_3$) δ 7.86 (d, J = 7.9 Hz, 1H), 7.83 (d, J = 7.6 Hz, 1H), 7.79 (s, 1H), 7.68 (d, J = 7.4 Hz, 2H), 7.64 (d, J = 7.8 Hz, 1H), 7.58 (d, J = 7.4 Hz, 1H), 7.48 (t, J = 7.7 Hz, 2H), 7.41 (t, J = 7.4 Hz, 1H), 7.37 (t, J = 7.4 Hz, 1H), 7.33 (t, J = 7.3 Hz, 1H), 3.98 (s, 2H); ^{13}C NMR (151 MHz, $CDCl_3$) δ 143.9, 143.5, 141.5, 141.4, 140.9, 139.9, 128.8, 127.2, 127.1, 126.8, 126.7, 126.0, 125.1, 123.8, 120.1, 120.0, 37.0; IR (ATR): 3034, 2320, 2084, 1993, 1595, 1450, 1412, 832, 746 cm^{-1}; Data in accordance with the literature.[19]

(*E*)-4-Styryl-1,1'-biphenyl (3Ga)

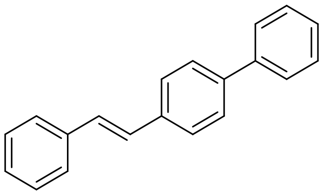

Following the general procedure, the title product was isolated as white solid after flash chromatography on silica gel. ^1H NMR (600 MHz, $CDCl_3$) δ 7.65–7.59 (m, 6H), 7.55 (d, J = 7.2 Hz, 2H), 7.46 (t, J = 7.7 Hz, 2H), 7.40–7.34 (m, 3H), 7.28 (t, J = 7.4 Hz, 1H), 7.17 (s, 2H); ^{13}C NMR (151 MHz, $CDCl_3$) δ 140.7, 140.3, 137.3, 136.4, 128.8, 128.7, 128.7, 128.2, 127.7, 127.4, 127.3, 126.9, 126.8, 126.5; IR (ATR): 3026, 2925, 2095, 1595, 1482, 1447, 1408, 966, 824, 758 cm^{-1}; MS (EI): m/z (%) 257.2 ($[M + H]^+$, 20), 256.2 (M^+, 94), 178.0 (83), 152.1 (25), 102.0 (15); Data in accordance with the literature.[20]

4-Methoxy-1,1'-biphenyl (3Ha)

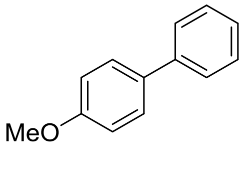

Following the general procedure, the title product was isolated as white solid after flash chromatography. ^1H NMR (400 MHz, $CDCl_3$): δ 7.60–7.52 (m, 4H), 7.43 (t, J = 7.6 Hz, 2H), 7.35–7.29 (m, 1H), 7.02–6.97 (m, 2H), 3.87 (s, 3H); ^{13}C NMR (100 MHz, $CDCl_3$): δ 159.2, 140.9, 133.9, 128.8, 128.2, 126.8, 126.7, 114.3, 55.4; IR (ATR): $\tilde{\nu}$ = 3053, 3000, 2958, 2175, 2059, 1598, 1472, 1244, 1187, 1033, 831, 757, 684 cm^{-1}; MS (EI): m/z (%) 184.9 (M^+, 24), 183.8 (100), 168.8 (57), 140.8 (50), 114.9 (30); Data in accordance with the literature.[18]

3-Methoxy-1,1'-biphenyl (3Ia)

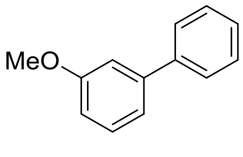

Following the general procedure, the title product was isolated as colorless oil after flash chromatography on silica gel. ^1H NMR (400 MHz, $CDCl_3$): δ 7.64–

7.58 (m, 2H), 7.49–7.42 (m, 2H), 7.40–7.34 (m, 2H), 7.21 (d, J = 7.7 Hz, 1H), 7.15 (s, 1H), 6.92 (dd, J = 8.2, 1.8 Hz, 1H), 3.88 (s, 3H); ^{13}C NMR (100 MHz, $CDCl_3$): δ 160.0, 142.9, 141.2, 129.8, 128.8, 127.5, 127.3, 119.8, 113.0, 112.8, 55.9; IR (ATR): 3040, 2944, 2835, 2325, 2095, 1893, 1753, 1592, 1473, 1420, 1297, 1213, 1172, 1038, 860, 754, 697 cm^{-1}; MS (EI): m/z (%) 184.4 (M$^+$, 100), 183.4 (75), 153.9 (66), 140.8 (92), 114.9 (86); Data in accordance with the literature.[21]

2-Methoxy-1,1'-biphenyl (3Ja)

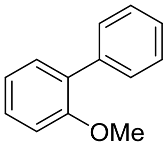

Following the general procedure, the title product was isolated as white solid after flash chromatography on silica gel. ^{1}H NMR (600 MHz, $CDCl_3$): δ 7.57–7.53 (m, 2H), 7.43 (t, J = 7.7 Hz, 2H), 7.36–7.32 (m, 3H), 7.05 (td, J = 7.5, 0.9 Hz, 1H), 7.01 (d, J = 8.5 Hz, 1H), 3.83 (s, 3H); ^{13}C NMR (150 MHz, $CDCl_3$): δ 156.5, 138.6, 131.0, 130.8, 129.6, 128.7, 128.1, 127.0, 120.9, 111.3, 55.6; IR (ATR): 3030, 2947, 2338, 1743, 1588, 1453, 1231, 1024, 728 cm^{-1}; MS (EI): m/z (%) 184.1 (M$^+$, 100), 169.1 (29), 141.1 (18), 115.1 (15); Data in accordance with the literature.[18]

3,4-Dimethoxy-1,1'-biphenyl (3Ka)

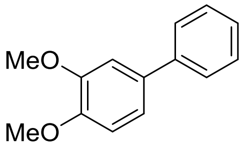

Following the general procedure, the title product was isolated as colorless oil after flash chromatography on silica gel. ^{1}H NMR (400 MHz, $CDCl_3$): δ 7.57 (d, J = 7.3 Hz, 2H), 7.43 (t, J = 7.6 Hz, 2H), 7.32 (t, J = 7.3 Hz, 1H), 7.19–7.10 (m, 2H), 6.96 (d, J = 8.3 Hz, 1H), 3.96 (s, 3H), 3.93 (s, 3H); ^{13}C NMR (100 MHz, $CDCl_3$): δ 149.2, 148.7, 141.1, 134.3, 128.8, 126.93, 126.90, 119.5, 111.6, 110.6, 56.1, 56.0; IR (ATR): 3002, 2960, 2935, 2835, 2080, 1700, 1597, 1517, 1484, 1448, 1405, 1246, 1215, 1172, 1140, 1023, 854, 813, 759, 700 cm^{-1}; MS (EI): m/z (%) 214.4 (M$^+$, 100), 199.1 (65), 171.1 (44), 153.1 (38); Data in accordance with the literature.[22]

5-Phenylbenzo[*d*][1,3]dioxole (3La)

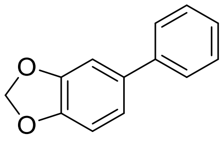

Following the general procedure, the title product was isolated as colorless oil after flash chromatography on silica gel. ^{1}H NMR (400 MHz, $CDCl_3$): δ 7.55–7.50 (m, 2H), 7.44–7.38 (m, 2H), 7.35–7.29 (m, 1H), 7.10–7.05 (m, 2H), 6.89 (d, J = 7.8 Hz, 1H), 6.00 (s, 2H); ^{13}C NMR (100 MHz, $CDCl_3$): δ 148.2, 147.1, 141.0, 135.7, 128.8, 127.00, 126.97,

120.7, 108.6, 107.8, 101.2; IR (ATR): 3028, 2889, 2322, 2104, 1858, 1604, 1475, 1431, 1225, 1036, 934, 756, 696 cm^{-1}; MS (EI): m/z (%) 198.9 (M^+, 14), 197.9 (100), 196.9 (46), 138.9 (60); Data in accordance with the literature.[21]

[1,1'-Biphenyl]-4-ol (3Ma)

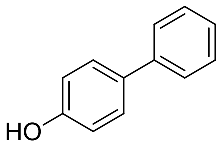

Following the general procedure, the title product was isolated as colorless oil after flash chromatography on silica gel. ^{1}H NMR (600 MHz, $CDCl_3$) δ 7.57–7.53 (m, 2H), 7.50–7.47 (m, 2H), 7.42 (t, J = 7.8 Hz, 2H), 7.31 (t, J = 7.4 Hz, 1H), 6.93–6.89 (m, 2H), 4.82 (s, 1H); ^{13}C NMR (151 MHz, $CDCl_3$) δ 155.0, 140.7, 134.0, 128.7, 128.4, 126.7, 115.6; IR (ATR): 3410, 3038, 2923, 1601, 1521, 1483, 1461, 1251, 830, 755 cm^{-1}; Data in accordance with the literature.[23]

3,5-Difluoro-1,1'-biphenyl (3Na)

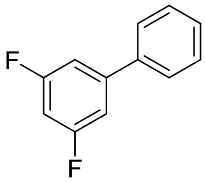

Following the general procedure, the title product was isolated as colorless oil after flash chromatography on silica gel. ^{1}H NMR (600 MHz, $CDCl_3$): δ 7.56 (d, J = 7.4 Hz, 2H), 7.46 (t, J = 7.6 Hz, 2H), 7.41 (t, J = 7.4 Hz, 1H), 7.15–7.08 (m, 2H), 6.79 (tt, J = 8.9, 2.2 Hz, 1H); ^{13}C NMR (150 MHz, $CDCl_3$): δ 163.43 (d, $J_{C\text{-}F}$ = 247.9 Hz), 163.35 (d, $J_{C\text{-}F}$ = 247.9 Hz), 144.6 (t, $J_{C\text{-}F}$ = 9.5 Hz), 139.0, 129.1, 128.5, 127.1, 110.03 (d, $J_{C\text{-}F}$ = 20.3 Hz), 110.00 (d, $J_{C\text{-}F}$ = 20.3 Hz), 102.6 (t, $J_{C\text{-}F}$ = 25.5 Hz); ^{19}F NMR (564 MHz, $CDCl_3$): δ - 109.9; IR (ATR): 3087, 3063, 2324, 2110, 1990, 1725, 1599, 1459, 1420, 1337, 1244, 1202, 1116, 1076, 986, 914, 859, 760, 693 cm^{-1}; MS (EI): m/z (%) 190.1 (M^+, 100), 154.0 (19); Data in accordance with the literature.[24]

4-(Trifluoromethyl)-1,1'-biphenyl (3Oa)

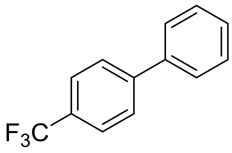

Following the general procedure, the title product was isolated as white solid after flash chromatography on silica gel. ^{1}H NMR (600 MHz, $CDCl_3$): δ 7.71 (s, 4H), 7.62 (d, J = 7.4 Hz, 2H), 7.49 (t, J = 7.6 Hz, 2H), 7.43 (t, J = 7.8 Hz, 1H); ^{13}C NMR (150 MHz, $CDCl_3$): δ 144.8, 139.8, 129.40 (d, $J_{C\text{-}F}$ = 32.5 Hz), 129.1, 128.3, 127.5, 127.4, 125.78 (q, $J_{C\text{-}F}$ = 3.7 Hz), 124.40 (q, $J_{C\text{-}F}$ = 271.9 Hz); ^{19}F NMR (564 MHz, $CDCl_3$): δ - 62.4; IR (ATR): 3038, 2933, 2318, 2101, 1610, 1403, 1321, 1114, 1004, 840, 763, 689 cm^{-1}; MS (EI): m/z (%) 222.9 (M^+, 13),

221.9 (100), 151.9 (14); Data in accordance with the literature.[18]

3-(Trifluoromethyl)-1,1'-biphenyl (3Pa)

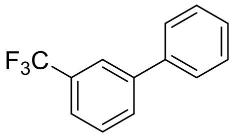

Following the general procedure, the title product was isolated as white solid after flash chromatography on silica gel. ^{1}H NMR (400 MHz, $CDCl_3$): δ 7.86 (s, 1H), 7.78 (d, J = 7.6 Hz, 1H), 7.65–7.54 (m, 4H), 7.49 (t, J = 7.4 Hz, 2H), 7.45–7.38 (m, 1H); ^{13}C NMR (100 MHz, $CDCl_3$): δ 142.1, 139.9, 131.3 (q, J = 32.5 Hz), 130.5, 129.3, 129.1, 128.1, 127.3, 124.3 (q, J = 272.6 Hz), 124.0 (qd, J = 3.9, 2.1 Hz); ^{19}F NMR (376 MHz, $CDCl_3$): δ - 62.6; IR (ATR): 3038, 2330, 2112, 1995, 1595, 1483, 1455, 1423, 1333, 1260, 1165, 1126, 1076, 1046, 898, 804, 758, 699, 658 cm^{-1}; MS (EI): m/z (%) 222.2 (M^+, 100), 201.1 (14), 152.1 (22); Data in accordance with the literature.[25]

4-(Trifluoromethoxy)-1,1'-biphenyl (3Qa)

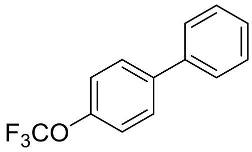

Following the general procedure, the title product was isolated as white solid after flash chromatography on silica gel. ^{1}H NMR (600 MHz, $CDCl_3$): δ 7.63–7.59 (m, 2H), 7.58–7.55 (m, 2H), 7.46 (t, J = 7.7 Hz, 2H), 7.41–7.36 (m, 1H), 7.30 (d, J = 7.9 Hz, 2H); ^{13}C NMR (150 MHz, $CDCl_3$): δ 148.8, 140.1, 139.9, 129.0, 128.5, 127.8, 127.2, 121.3, 120.63 (d, *J* = 257.1 Hz); ^{19}F NMR (564 MHz, $CDCl_3$): δ - 57.8; IR (ATR): 2947, 2345, 1740, 1486, 1165, 845, 692 cm^{-1}; MS (EI): m/z (%) 238.2 (M^+, 100), 169.1 (28), 141.1 (30), 115.1 (19); Data in accordance with the literature.[26]

Methyl [1,1'-biphenyl]-4-carboxylate (3Ra)

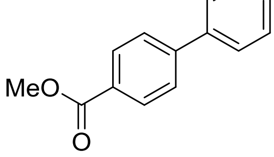

Following the general procedure, the title product was isolated as white solid after flash chromatography on silica gel. ^{1}H NMR (400 MHz, $CDCl_3$): δ 8.12 (d, J = 8.6 Hz, 2H), 7.69–7.60 (m, 4H), 7.47 (t, J = 7.3 Hz, 2H), 7.40 (t, J = 7.3 Hz, 1H), 3.95 (s, 3H); ^{13}C NMR (100 MHz, $CDCl_3$): δ 167.1, 145.7, 140.1, 130.2, 128.98, 128.96, 128.2, 127.3, 127.1, 52.2; IR (ATR): 3414, 2938, 2343, 2092, 1907, 1693, 1421, 1246, 1111, 720 cm^{-1}; MS (EI): m/z (%) 212.2 (M^+, 100), 181.1 (87), 152.1 (42); Data in accordance with the literature.[22]

4-Phenylpyridine (3Sa)

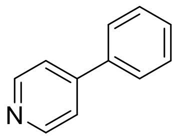

Following the general procedure, the title product was isolated as white solid after flash chromatography on silica gel. ^{1}H NMR (400 MHz, $CDCl_3$): δ 8.66 (d, J = 4.4 Hz, 2H), 7.66–7.61 (m, 2H), 7.53–7.42 (m, 5H); ^{13}C NMR (100 MHz, $CDCl_3$): δ 150.3, 148.5, 138.2, 129.18, 129.15, 127.1, 121.7; IR (ATR): 3038, 2329, 2092, 1900, 1668, 1589, 1408, 1281, 1167, 941, 830, 749, 691 cm^{-1}; MS (EI): m/z (%) 155.9 (M^+, 49), 154.9 (100), 153.9 (58), 127.0 (15); Data in accordance with the literature.[27]

2-Phenylbenzofuran (3Ta)

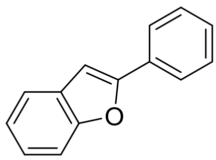

Following the general procedure, the title product was isolated as white solid after flash chromatography on silica gel. ^{1}H NMR (600 MHz, $CDCl_3$): δ 7.93–7.87 (m, 2H), 7.61 (d, J = 7.7 Hz, 1H), 7.58–7.54 (m, 1H), 7.50–7.45 (m, 2H), 7.41–7.36 (m, 1H), 7.34–7.29 (m, 1H), 7.29–7.24 (m, 1H), 7.05 (s, 1H); ^{13}C NMR (150 MHz, $CDCl_3$): δ 156.0, 154.9, 130.5, 129.3, 128.9, 128.6, 125.0, 124.3, 123.0, 121.0, 111.3, 101.4; IR (ATR): 3046, 2324, 2091, 1900, 1603, 1561, 1444, 1256, 1168, 1017, 918, 805, 742, 687 cm^{-1}; MS (EI): m/z (%) 193.9 (M^+, 100), 164.9 (75), 138.9 (19); Data in accordance with the literature.[28]

3-Phenylbenzo[*b*]thiophene (3Ua)

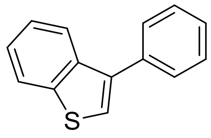

Following the general procedure, the title product was isolated as white solid after flash chromatography on silica gel. ^{1}H NMR (600 MHz, $CDCl_3$): δ 7.95–7.90 (m, 2H), 7.62–7.58 (m, 2H), 7.49 (t, J = 7.6 Hz, 2H), 7.43–7.37 (m, 4H); ^{13}C NMR (150 MHz, $CDCl_3$): δ 140.8, 138.2, 138.0, 136.1, 128.8, 127.6, 125.0, 124.4, 123.5, 123.0; IR (ATR): 3060, 3031, 2107, 1716, 1675, 1476, 1441, 1236, 1148, 1085, 975, 905, 846, 781, 730, 694 cm^{-1}; MS (EI): m/z (%) 210.1 (M^+, 100), 165.0 (47); Data in accordance with the literature.[18]

4-Phenylquinoline (3Va)

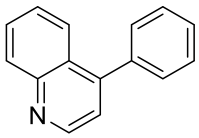

Following the general procedure, the title product was isolated as colorless oil after flash chromatography on silica gel. ^{1}H NMR (400 MHz, $CDCl_3$): δ 8.95 (d, J = 4.1 Hz, 1H), 8.19 (d, J = 8.4 Hz, 1H), 7.96–7.90 (m, 1H), 7.76–7.70 (m, 1H), 7.56–7.47 (m, 6H), 7.34 (d, J = 4.4 Hz, 1H); ^{13}C NMR (100 MHz, $CDCl_3$): δ 150.1, 148.8, 148.6, 138.1, 129.9,

129.6, 129.4, 128.7, 128.5, 126.8, 126.7, 126.0, 121.4; IR (ATR): 3036, 2677, 2341, 2096, 1738, 1574, 1488, 1384, 850, 764 cm^{-1}; MS (EI): m/z (%) 205.1 (M^+, 100), 204.1 (86), 176.1 (24); Data in accordance with the literature.[29]

4-(4-(*Tert*-butyl)phenyl)-2-phenylfuran (3Wa)

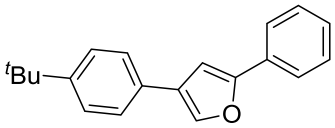

Following the general procedure, the title product was isolated as white solid after flash chromatography on silica gel. Mp: 46-48 °C; ^{1}H NMR (600 MHz, $CDCl_3$): δ 7.75–7.71 (m, 3H), 7.49 (d, J = 8.4 Hz, 2H), 7.46–7.39 (m, 4H), 7.29 (t, J = 7.4 Hz, 1H), 6.96 (s, 1H), 1.36 (s, 9H); ^{13}C NMR (150 MHz, $CDCl_3$): δ 154.8, 150.3, 137.8, 130.8, 129.6, 128.8, 128.3, 127.6, 125.8, 125.6, 123.9, 104.2, 34.7, 31.4; IR (ATR): 2945, 2333, 1751, 1455, 1305, 1121, 810, 695 cm^{-1}; MS (EI): m/z (%) 276.2 (M^+, 99), 262.1 (23), 261.1 (100), 116.5 (11); HRMS (ESI) for $C_{20}H_{21}O$: calcd. for $[M+H]^+$ 277.15869, found 277.15869.

(1*S*,2*R*,5*S*)-2-Isopropyl-5-methylcyclohexyl [1,1'-biphenyl]-4-carboxylate (3Xa)

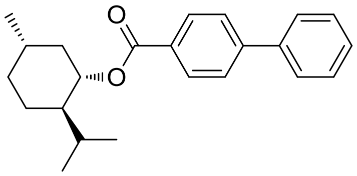

Following the general procedure, the title product was isolated as colorless oil after flash chromatography on silica gel. ^{1}H NMR (600 MHz, $CDCl_3$) δ 8.11 (d, J = 8.3 Hz, 2H), 7.66 (d, J = 8.3 Hz, 2H), 7.62 (d, J = 7.3 Hz, 2H), 7.47 (t, J = 7.6 Hz, 2H), 7.40 (t, J = 7.4 Hz, 1H), 4.96 (td, J = 10.9, 4.4 Hz, 1H), 2.15 (d, J = 12.0 Hz, 1H), 2.02–1.95 (m, 1H), 1.76–1.72 (m, 2H), 1.61–1.56 (m, 2H), 1.17–1.10 (m, 2H), 0.93 (dd, J = 6.6, 5.1 Hz, 7H), 0.81 (d, J = 6.9 Hz, 3H); ^{13}C NMR (151 MHz, $CDCl_3$) δ 166.0, 145.5, 140.1, 130.1, 129.6, 128.9, 128.1, 127.3, 127.0, 74.8, 47.3, 41.0, 34.3, 31.5, 26.5, 23.6, 22.1, 20.8, 16.5; IR (ATR): 3054, 2956, 1712, 1604, 1479, 1448, 1313, 1112, 701 cm^{-1}; MS (EI): m/z (%) 337.2 ($[M + H]^+$, 6), 336.1 (M^+, 9), 198.0 (41), 181.0 (73), 95.1 (63).

((3a*R*,5*R*,5a*S*,8a*S*,8b*R*)-2,2,7,7-Tetramethyltetrahydro-3a*H*-bis([1,3]dioxolo)[4,5-b:4',5'-d]pyran-5-yl)methyl [1,1'-biphenyl]-4-carboxylate (3Ya)

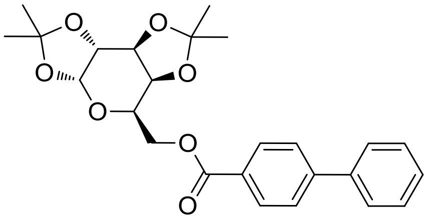

Following the general procedure, the title product was isolated as colorless viscous oil after flash chromatography on silica gel. ^{1}H NMR (600 MHz, $CDCl_3$) δ 8.12 (d, J = 8.3 Hz, 2H), 7.66 (d, J = 8.3 Hz, 2H), 7.62 (d, J = 7.6 Hz, 2H), 7.47 (t, J = 7.6 Hz, 2H),

7.40 (t, J = 7.4 Hz, 1H), 5.58 (d, J = 5.0 Hz, 1H), 4.67 (dd, J = 7.9, 2.3 Hz, 1H), 4.56 (dd, J = 11.5, 4.8 Hz, 1H), 4.45 (dd, J = 11.4, 7.6 Hz, 1H), 4.37–4.32 (m, 2H), 4.23–4.18 (m, 1H), 1.51 (d, J = 25.2 Hz, 6H), 1.35 (d, J = 15.3 Hz, 6H); ^{13}C NMR (151 MHz, $CDCl_3$) δ 166.3, 145.7, 140.0, 130.2, 128.9, 128.8, 128.1, 127.3, 127.0, 109.7, 108.8, 96.3, 71.1, 70.7, 70.5, 66.2, 63.9, 26.0, 26.0, 25.0, 24.5; IR (ATR): 3062, 2986, 1722, 1607, 1452, 1379, 1275, 1105, 1006, 896, 710 cm^{-1}; MS (EI): m/z (%) 441.1 ([M + H]$^+$, 2), 440.1 (2), 227.1 (11), 181.0 (100), 113.1 (15), 81.1 (30); HRMS (ESI) for $C_{25}H_{28}O_7Na$: calcd. for [M+Na]$^+$ 463.17272, found 463.17184.

(3*S*,5*S*,8*R*,9*S*,10*S*,13*R*,14*S*,17*R*)-10,13-dimethyl-17-((*R*)-6-methylheptan-2-yl)hexadecahydro-1*H*-cyclopenta[*a*]phenanthren-3-yl [1,1'-biphenyl]-4-carboxylate (3Za)

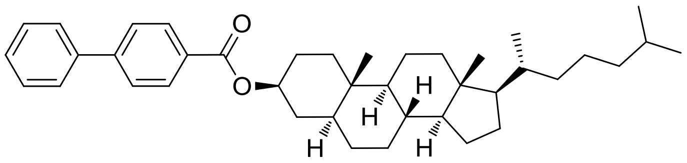

Following the general procedure, the title product was isolated as white solid after flash chromatography on silica gel. Mp: 70-72 °C; ^{1}H NMR (600 MHz, $CDCl_3$) δ 8.10 (d, J = 8.3 Hz, 2H), 7.63 (dd, J = 15.6, 7.8 Hz, 4H), 7.47 (t, J = 7.6 Hz, 2H), 7.39 (t, J = 7.4 Hz, 1H), 5.00–4.93 (m, 1H), 2.01–1.94 (m, 2H), 1.82–1.65 (m, 5H), 1.55–1.49 (m, 5H), 1.32–1.25 (m, 9H), 1.15–0.97 (m, 10H), 0.91–0.86 (m, 12H), 0.66 (s, 3H); ^{13}C NMR (151 MHz, $CDCl_3$) δ 166.0, 145.4, 140.1, 130.0, 129.7, 128.9, 128.1, 127.3, 126.9, 74.4, 56.4, 56.2, 54.2, 44.7, 42.6, 40.0, 39.5, 36.8, 36.2, 35.8, 35.5, 35.5, 34.2, 32.0, 28.6, 28.3, 28.0, 27.6, 24.2, 23.8, 22.8, 22.6, 21.2, 18.7, 12.3, 12.1; IR (ATR): 2934, 2869, 1709, 1601, 1476, 1443, 1376, 1316, 1282, 1130, 1005, 699 cm^{-1}; MS (EI): m/z (%) 568.9 (M$^+$, 5), 519.3 (5), 469.9 (6), 305.6 (6), 279.1 (27), 167.0 (52), 112.1 (10); HRMS (EI) for $C_{40}H_{56}O_2$: calcd. for [M]$^+$ 568.42748, found 568.42866.

(3*S*,8*S*,9*S*,10*R*,13*S*,14*S*,17*S*)-17-Acetyl-10,13-dimethyl-2,3,4,7,8,9,10,11,12,13,14,15,16,17-tetradecahydro-1H-cyclopenta[a]phenanthren-3-yl [1,1'-biphenyl]-4-carboxylate (3AAa)

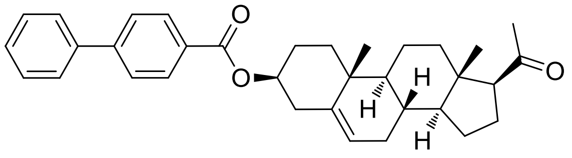

Following the general procedure, the title product was isolated as white solid after flash chromatography on silica gel. ^{1}H NMR (600 MHz, $CDCl_3$) δ 8.11 (d, J = 8.3 Hz, 2H), 7.66 (d, J = 8.3 Hz, 2H), 7.62 (d, J = 7.3 Hz, 2H), 7.47 (t, J = 7.6 Hz, 2H), 7.39 (t, J = 7.4 Hz, 1H), 5.43 (d, J = 4.9 Hz, 1H), 4.92–4.86 (m, 1H), 2.55 (t, J = 9.0 Hz, 1H), 2.49 (d, J = 7.6 Hz, 2H), 2.20–2.16 (m, 1H), 2.13 (s, 3H), 2.07–2.03 (m, 2H), 1.97–1.91 (m, 1H), 1.77–1.61

(m, 6H), 1.53–1.47 (m, 3H), 1.27–1.18 (m, 4H), 1.08 (s, 3H), 0.65 (s, 3H); ^{13}C NMR (151 MHz, $CDCl_3$) δ 209.6, 165.9, 145.4, 140.1, 139.7, 130.1, 129.5, 128.9, 128.1, 127.3, 127.0, 122.5, 74.4, 63.7, 56.8, 49.9, 44.0, 38.8, 38.2, 37.0, 36.7, 31.8, 31.8, 31.6, 27.9, 24.5, 22.8, 21.1, 19.4, 13.2; IR (ATR): 2941, 2898, 1705, 1607, 1469, 1447, 1354, 1274, 1113, 1001, 855, 744 cm^{-1}; HRMS (EI) for $C_{34}H_{40}O_3$: calcd. for $[M]^+$ 496.29720, found 496.29591.

Supplementary Note 6. Procedure for the large-scale experiment

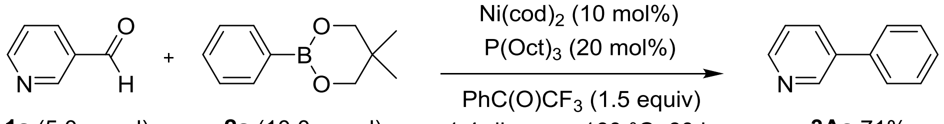

1a (5.0 mmol) **2a** (10.0 mmol) **3Aa** 71%

In a nitrogen-filled glovebox, a 100-mL oven-dried sealed tube containing a stirring bar was charged with nicotinaldehyde **1a** (470 μL, 5.0 mmol, 1.0 equiv), 5,5-dimethyl-2-phenyl-1,3,2-dioxaborinane **2a** (1.90 g, 10.0 mmol, 2.0 equiv) and yellow $Ni(cod)_2$ (137.5 mg, 10 mol%). Subsequently, 1,4-dioxane (25 mL) and 2,2,2-trifluoroacetophenone (1.05 mL, 7.5 mmol, 1.5 equiv) was added via syringe, and then trioctylphosphine ligand (450 μL, 20 mol%) was added via microsyringe. The tube was sealed and removed from the glovebox. After stirring at 160 °C for 60 h, the mixture was allowed to cool to room temperature, diluted with EtOAc (30 mL) and filtered through celite plugs, eluting with additional EtOAc (30 mL). The filtrate was concentrated and purified by column chromatography on silica gel (eluent: Hexane/EA = 10:1, then 4:1) to yield the title product **3Aa** as yellow oil (546.8 mg, 71%).

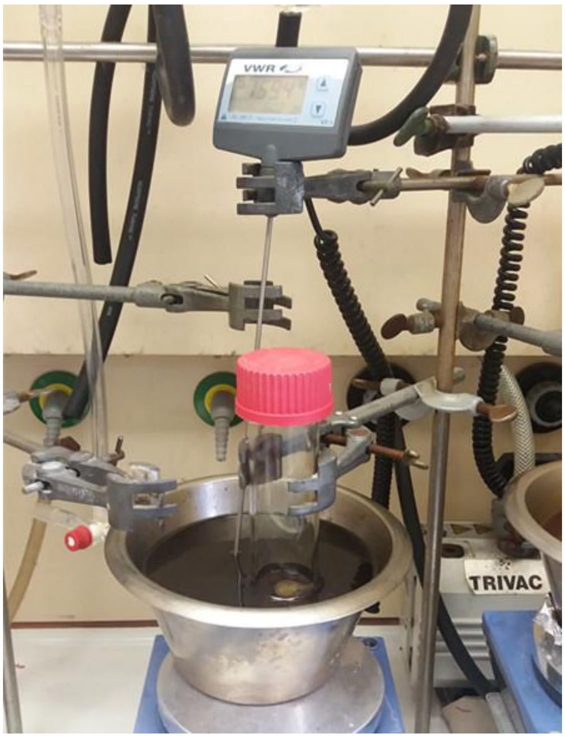

Supplementary Figure 3. Set-up for the large scale reaction.

Supplementary Discussion

Supplementary Note 7. Procedure for the isotope-labeling experiment

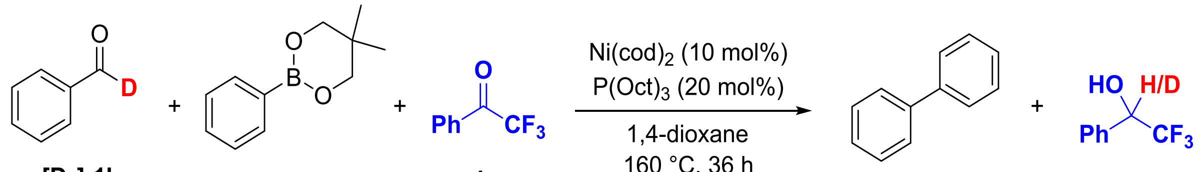

[D₁]-1b (> 99% D) + **2a** (2.0 equiv.) + **4a** (1.5 equiv.) → **3Ba**, 80% + **5**, 67% (85% D)

Deuterated aldehyde **[D_1]-1b** (benzaldehyde-α-[D_1], > 99% D) was synthesized according to a literature method.[20] In a nitrogen-filled glovebox, a 10-mL oven-dried sealed tube containing a stirring bar was charged with phenyl boronic ester **2a** (152 mg, 0.80 mmol, 2.0 equiv.) and yellow Ni(cod)$_2$ (11 mg, 10 mol%). Subsequently, 1,4-dioxane (2.0 mL) was added, and then deuterated aldehyde **[D_1]-1b** (41 μL, 0.40 mmol, 1.0 equiv.), trioctylphosphine ligand (36 μL, 20 mol%) and 2,2,2-trifluoroacetophenone (**4a**, 84 μL, 0.60 mmol, 1.5 equiv.) were added respectively via microsyringe. The tube with the mixture was sealed and removed from the glovebox. After stirring at 160 °C for 36 h, the mixture was allowed to cool to room temperature, diluted with EtOAc (15 mL) and filtered through a celite plug, eluting with water (20 mL) and additional EtOAc (10 mL). The aqueous layer was extracted with ethyl acetate (3 × 15 mL) and the combined organic extracts were washed with brine, dried over Na_2SO_4, and then filtered. The filtrate was concentrated in *vacuo* and the residue was purified by column chromatography on silica gel to yield the title product **3Ba** (80% yield, eluent: hexane) and **5** (67% yield, eluent: hexane/ethyl acetate = 10:1). Compound **3Ba**:[18] ^{1}H NMR (400 MHz, $CDCl_3$): δ 7.67–7.62 (m, 4H), 7.52–7.46 (m, 4H), 7.42–7.36 (m, 2H). Compound **5** (85% D):[31] ^{1}H NMR (400 MHz, $CDCl_3$): δ 7.50–7.43 (m, 2H), 7.43–7.37 (m, 3H), 5.03–4.96 (m, 0.15H), 2.73 (d, J = 22.5 Hz, 1H).

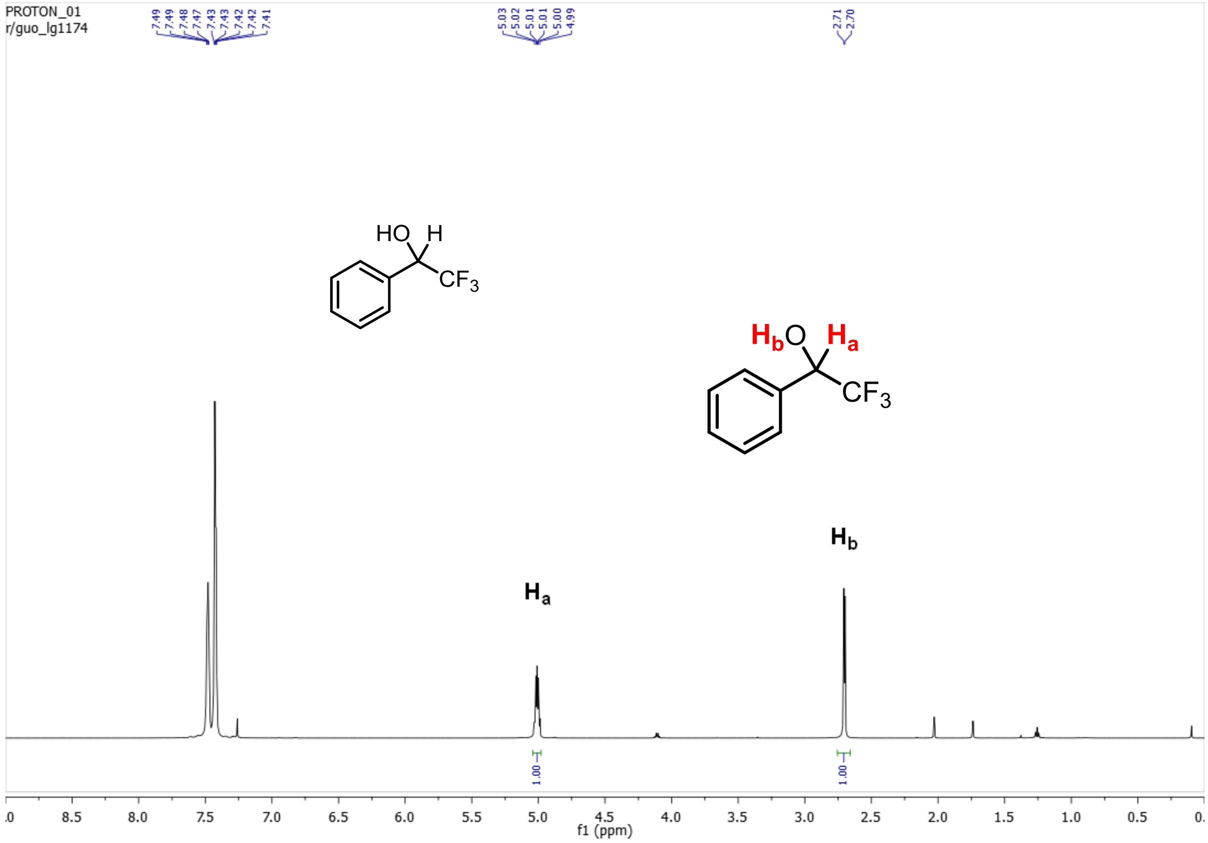

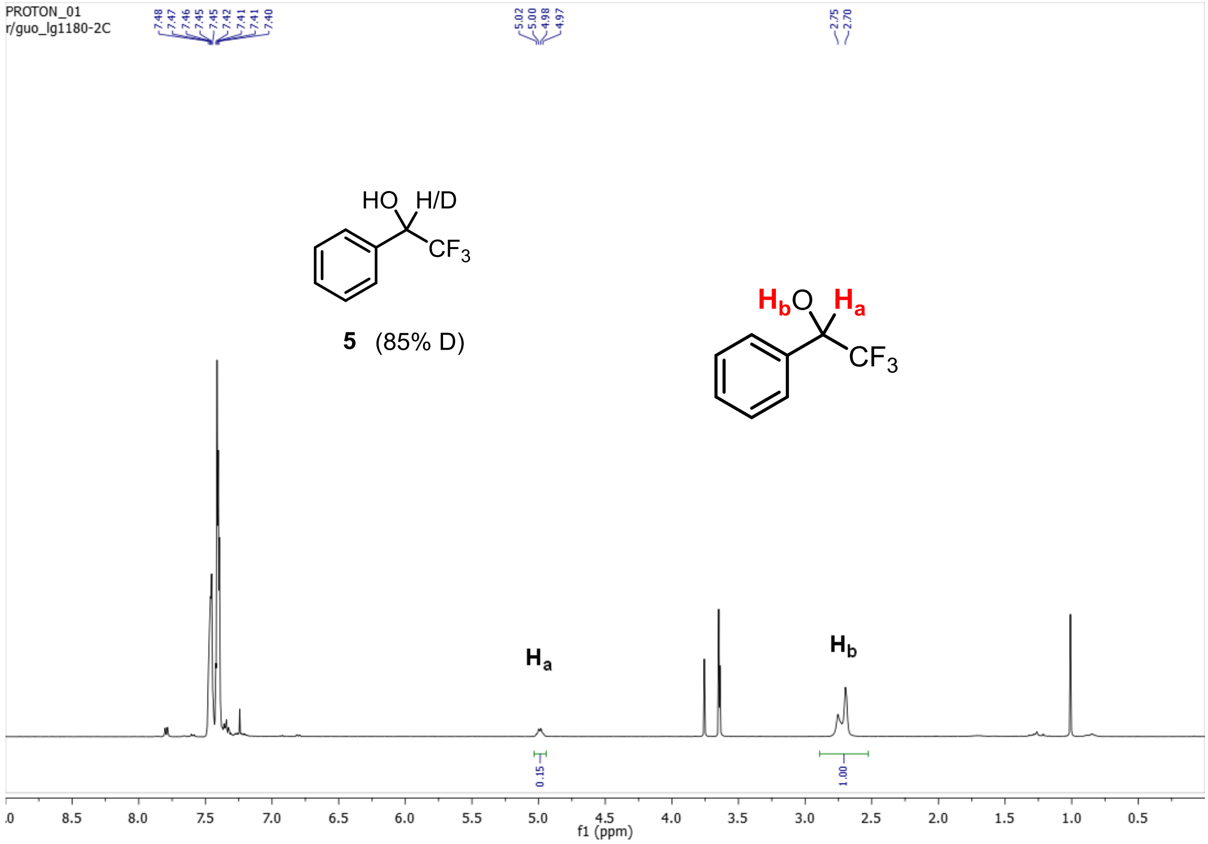

Supplementary Figure 4. ^{1}H NMR in $CDCl_3$ for compounds **5** and **5-D** (85% D).

Supplementary Note 8. Procedure for the kinetic isotope effect

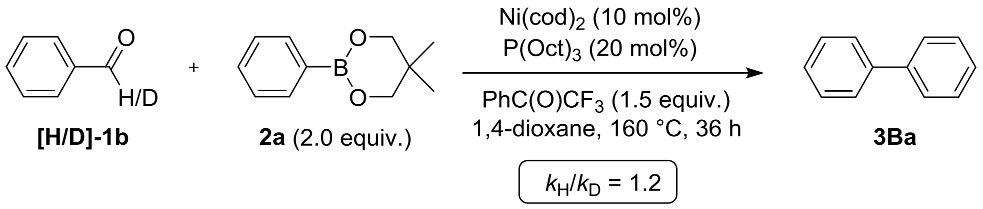

Two parallel reactions of **2a** with **1b** and **[D$_1$]-1b** respectively were performed to determine the corresponding KIE value. In a nitrogen-filled glovebox, a 10-mL oven-dried sealed tube containing a stirring bar was charged with phenyl boronic ester **2a** (76 mg, 0.40 mmol, 2.0 equiv.) and yellow Ni(cod)$_2$ (5.5 mg, 10 mol%). Subsequently, 1,4-dioxane (1.5 mL) was added, and then distilled benzaldehyde **1b** (20 μL, 0.20 mmol) or deuterated benzaldehyde **[D$_1$]-1b** (20 μL, 0.20 mmol), trioctylphosphine ligand (18 μL, 20 mol%), 2,2,2-trifluoroacetophenone (42 μL, 0.30 mmol, 1.5 equiv.), and decane (39 μL, as internal standard) were added respectively via microsyringe. The tube with the mixture was sealed and the mixture was stirred at 160 °C. At different reaction time, a small amount of reaction solution was taken by microsyringe and analyzed by GC to provide the following conversion:

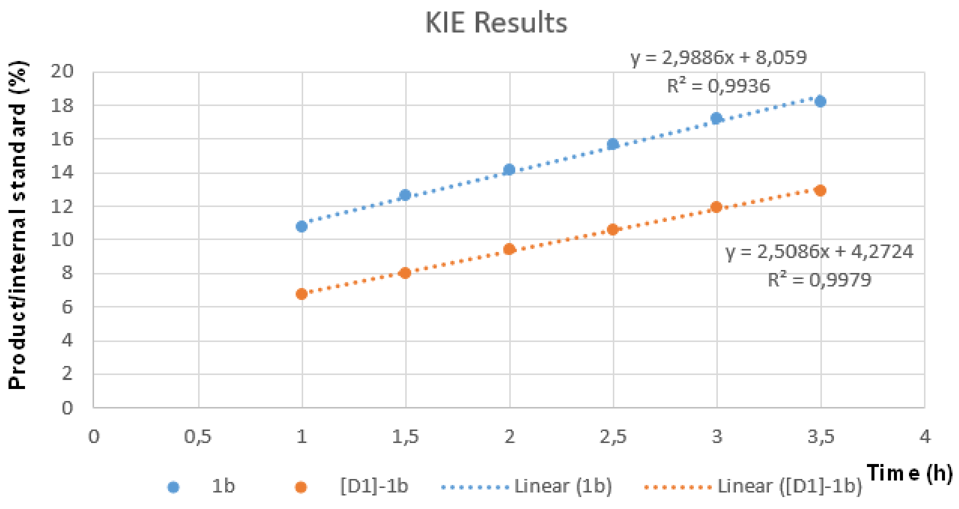

Supplementary Figure 5. Kinetic isotope effect results.

Supplementary Table 5.

Time (h)	0.5	1.0	1.5	2.0	2.5	3.0	3.5
**1b**	8.1	10.8	12.6	14.2	15.7	17.2	18.2
**[D$_1$]-1b**	4.6	6.7	8.0	9.4	10.6	11.9	12.9

Supplementary Note 9. Computational methods

All the geometries were optimized with the generalized gradient approximation (GGA) method with Gaussian 09, Revision D.01,[32] using meta-hybrid-GGA DFT functional ωB97xD.[33] The electronic configuration of all the non-metal elements was described with the Ahlrichs split-valance polarization basis function Def2-SVP while Ni is treated with the triple-ζ valence basis set Def2-TZVP.[34,35] The geometries were optimized without any symmetry constraints. Harmonic force constants were computed at the optimized geometries to characterize the stationary points as minima or saddle points. All transition states were optimized using the default Berny algorithm implemented in the Gaussian09 code.[32] For transition state structures, IRC calculations were undertaken to confirm the transition states were connected to the correct minima. For further validation of energetics, single-point calculations were performed on the ωB97xD/Def2-TZVP(Ni)/Def2-SVP(non-metal) optimized geometries using meta-hybrid-GGA functional M06[36] employing a valence triple-ζ-type of basis set Def2-TZVPP[34,35] for all atoms. The solvent effects (1,4-dioxane, ε = 2.2099) were evaluated implicitly by a self-consistent reaction field (SCRF) approach for all the intermediates and transitions states, using the SMD continuum solvation model.[37] Unless specified otherwise, the ΔG was used throughout the text. The ΔG value was obtained by augmenting the E_{el} energy terms at M06(SMD)/Def2-TZVPP with the respective free energy corrections at the ωB97xD/Def2-TZVP (Ni)/Def2-SVP (non-metal) level in gas phase. In all cases, the default integral grid (Fine Grid) was employed. The simplified trialkyl-phosphine ligand P^nPr_3 was used in the DFT calculation, which gave 53% yield (see Supplementary Table 1, entry 4). Ball and stick models are made using CYLView visualization programs.[38]

Oxidative addition pathways. Based on our previous research,[39] we proposed that the oxidative addition of the aryl-aldehyde **1b** to Ni^0-intermediate **A** has two activation modes, namely C(acyl)-H activation or C(aryl)-C(acyl) activation. Two different oxidative addition pathways were calculated. The results indicated the C(acyl)-H activation pathway was both kinetically (-6.0 kcal/mol) and thermodynamically (-9.3 kcal/mol) favored (Supplementary Figure 6).

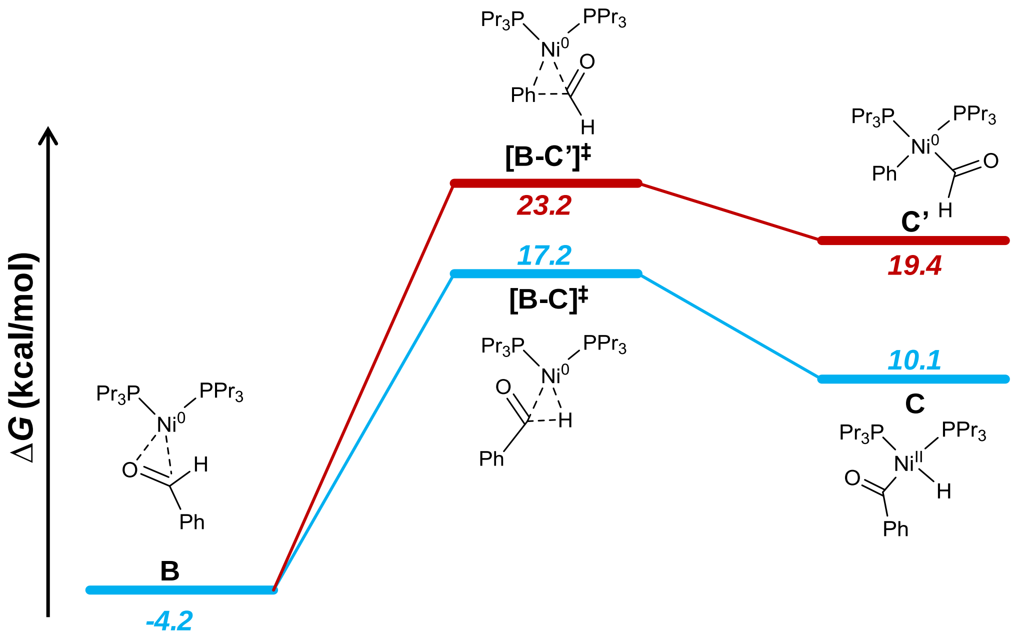

Supplementary Figure 6. Oxidative addition pathways.

Competition reactions between hydride transfer and decarbonylation

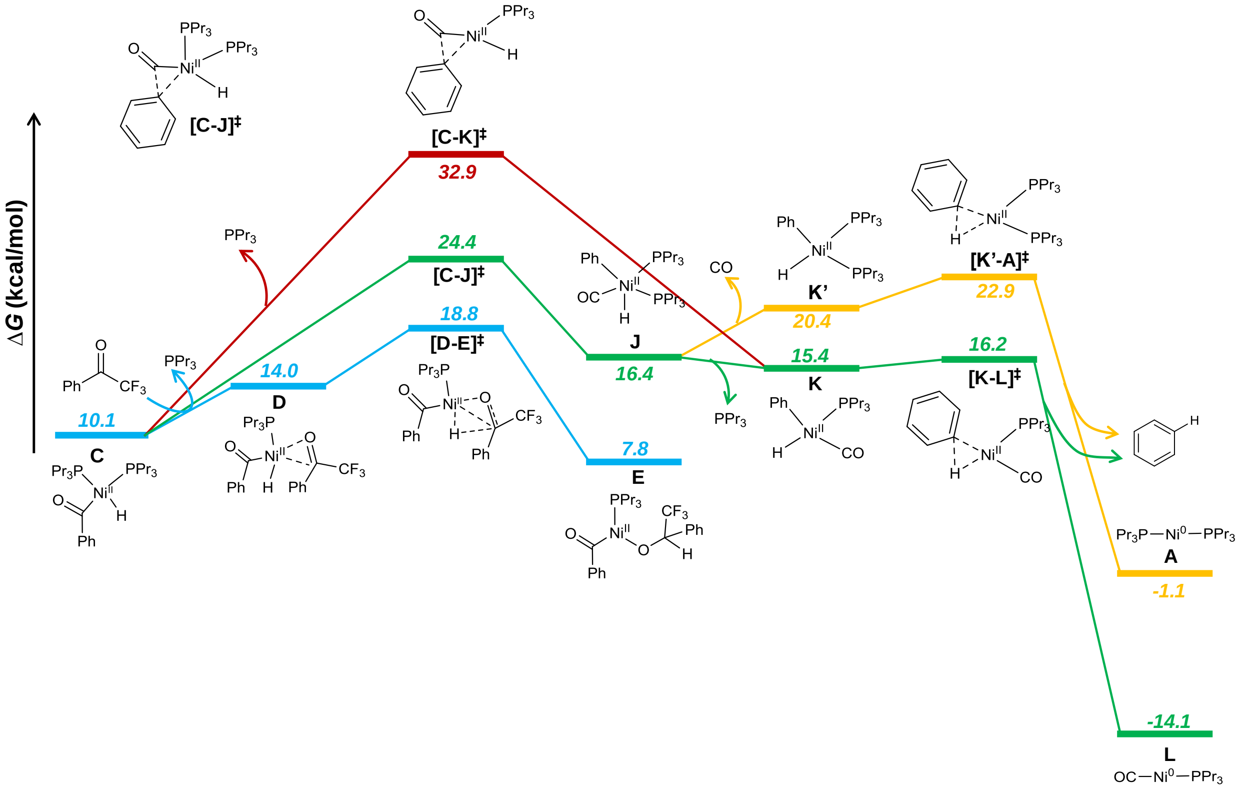

Supplementary Figure 7. Competition reactions between hydride transfer and decarbonylation followed by reductive elimination. Blue line describes the hydride migration step and green line describes decarbonylation followed by reductive elimination of C(aryl)-H bond. Alternative pathway (red line) is highly unfavorable compared to the green line by 8.5 kcal/mol. On the other hand alternative reductive elimination route (orange line) is also unfavorable by 6.7 kcal/mol compared to the green line.

Competition reactions between transmetalation and decarbonylation

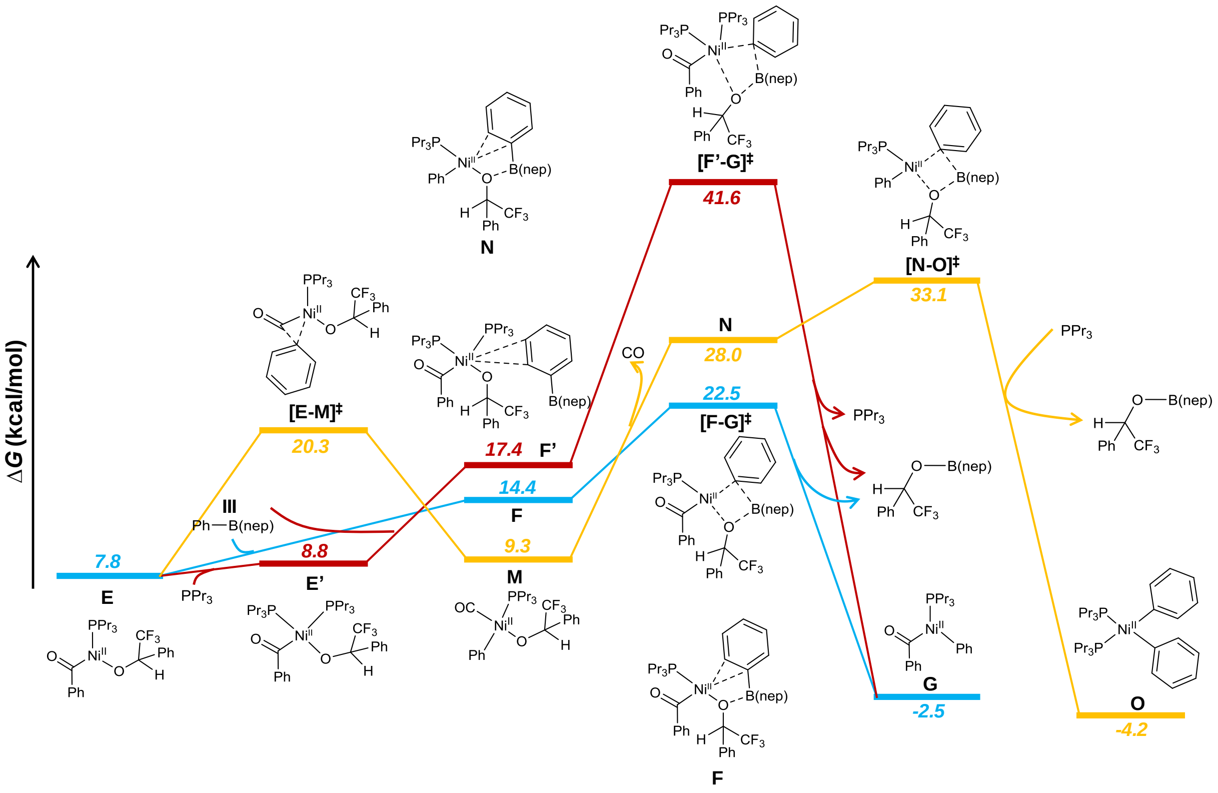

Supplementary Figure 8. Competition reactions between transmetalation and decarbonylation. Blue path is transmetalation in the presence of one phosphine, while in alternative red path two phosphines are involved which is highly unfavorable by 19.1 kcal/mol. Orange pathway describes the decarbonylation prior to the transmetalation, which is also less favorable compared to the blue line.

Reductive elimination pathways

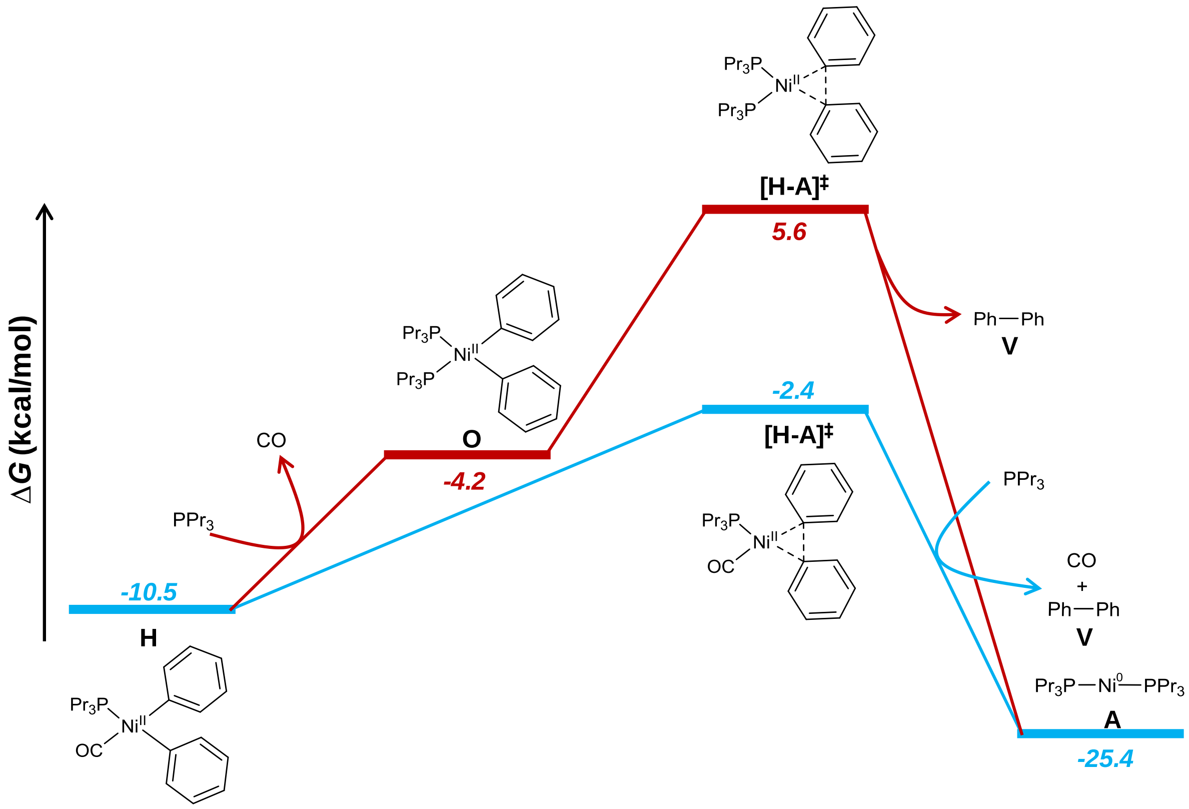

Supplementary Figure 9. Reductive elimination pathways. These two pathways are differed by the ligand combination. Two phosphines are involved in the red pathway, while one phosphine is replaced by CO in the blue pathway. However, the blue line is favored compared to the red line by 8.0 kcal/mol.

Energy barrier of hydride transfer and transmetalation process for different ketones/aldehydes

Hydride Transfer **Transmetalation**

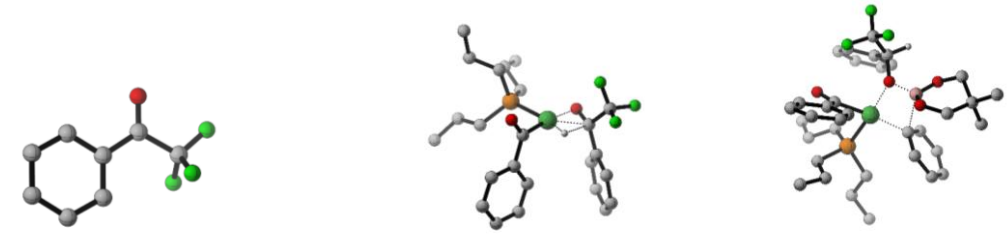

II **[D-E]‡ $\Delta G^{‡}$ = 23.0 kcal/mol** **[F-G]‡ $\Delta G^{‡}$ = 26.7 kcal/mol**

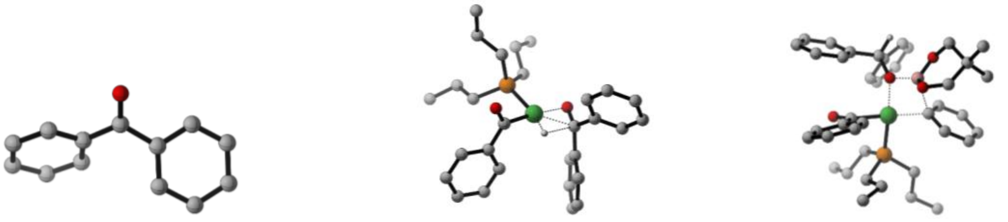

II_b **$[D\text{-}E]_b^{‡}$ $\Delta G^{‡}$ = 27.7 kcal/mol** **$[F\text{-}G]_b^{‡}$ $\Delta G^{‡}$ = 33.0 kcal/mol**

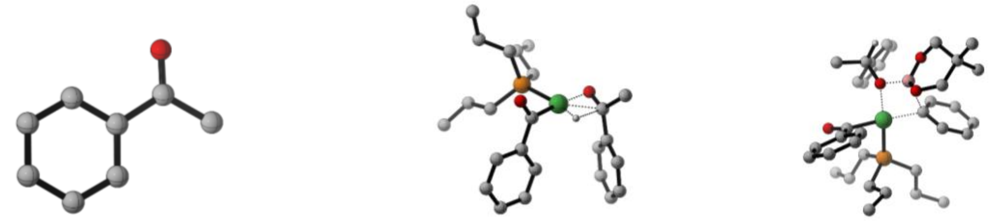

II_c **$[D\text{-}E]_c^{‡}$ $\Delta G^{‡}$ = 29.8 kcal/mol** **$[F\text{-}G]_c^{‡}$ $\Delta G^{‡}$ = 33.5 kcal/mol**

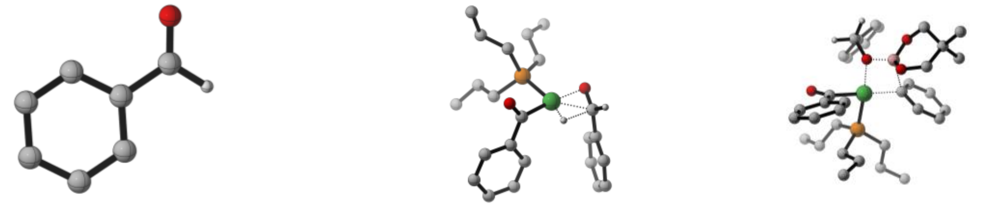

1b **$[D\text{-}E]_d^{‡}$ $\Delta G^{‡}$ = 25.2 kcal/mol** **$[F\text{-}G]_d^{‡}$ $\Delta G^{‡}$ = 30.2 kcal/mol**

Supplementary Figure 10. Energy barrier of hydride transfer and transmetalation process for different ketones/aldehydes. Benzophenone, acetophenone and benzaldehyde have significantly higher barrier for both process compared to α,α,α-trifluoroacetophenone.

Boronic acid as nucleophile

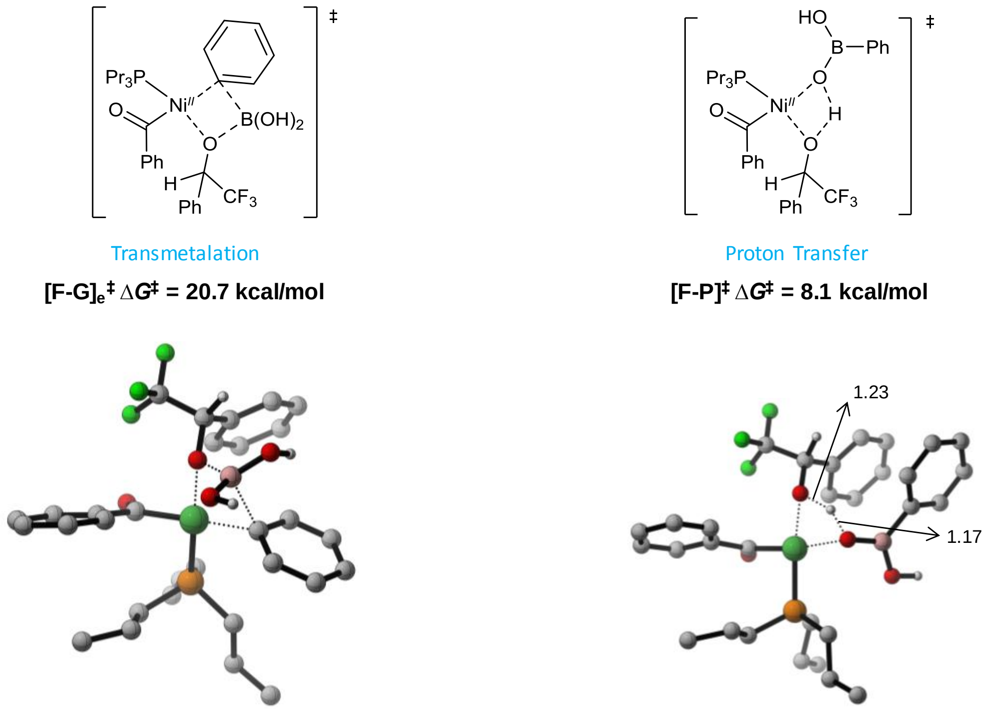

Supplementary Figure 11. Energy barrier of transmetalation and proton transfer process for boronic acid as nucleophile. The boronic acid is more reactive as nucleophile compared to boronic esters. However, the proton transfer from boronic acid to O-CH(CF_3)(Ph) is significantly easier (only 8.1 kcal/mol barrier), which blocked the transmetalation of aryl-group to Ni^{II}-complex (see SI III, entry 1, only trace amount of product was formed using boronic acid as nucleophile).

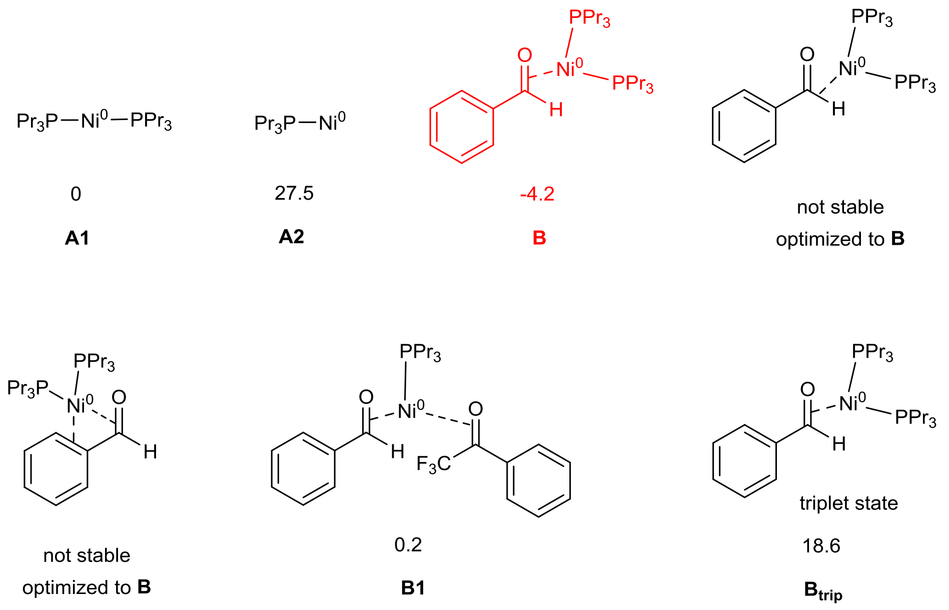

Supplementary Figure 12. DFT-computed Gibbs free energy (in kcal/mol) for conformers and isomers of key intermediates or transition states.

Copies of ^{1}H, ^{13}C and ^{19}F NMR Spectra

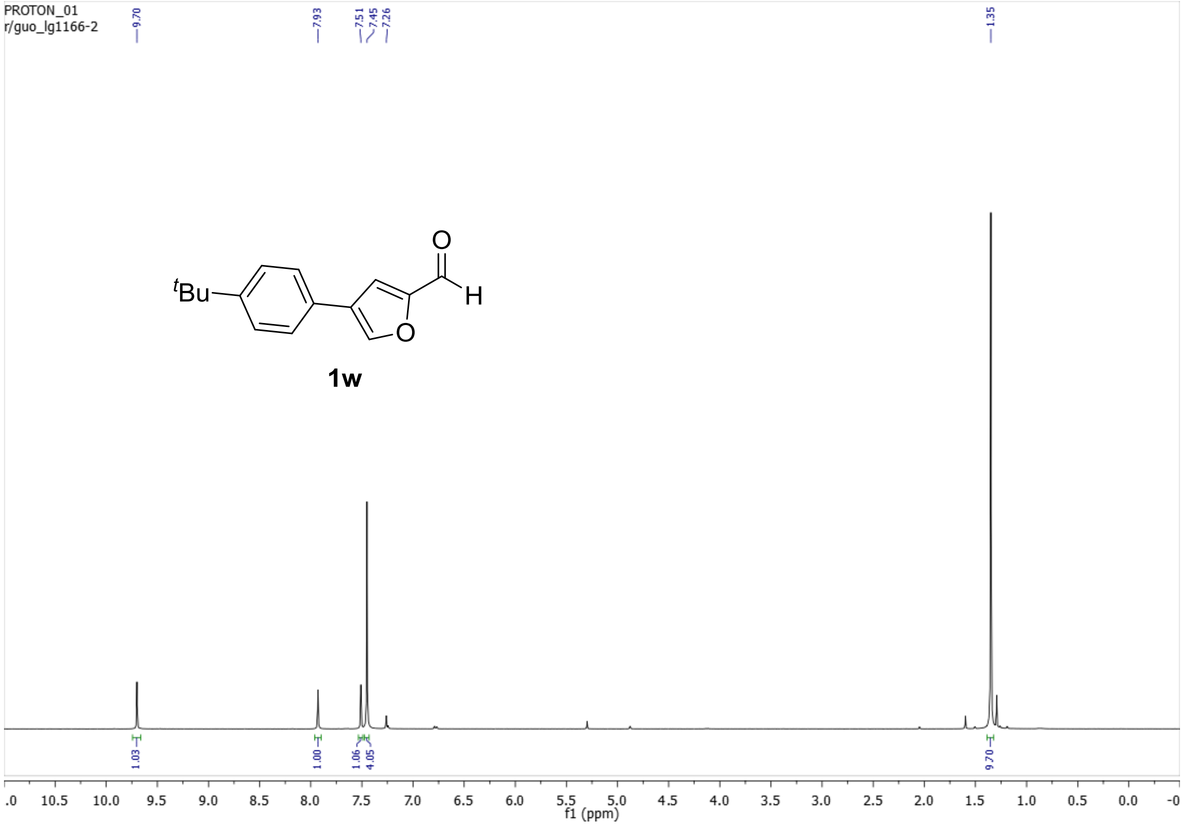

Supplementary Figure 13. ^{1}H NMR spectrum in $CDCl_3$ of compound **1w**.

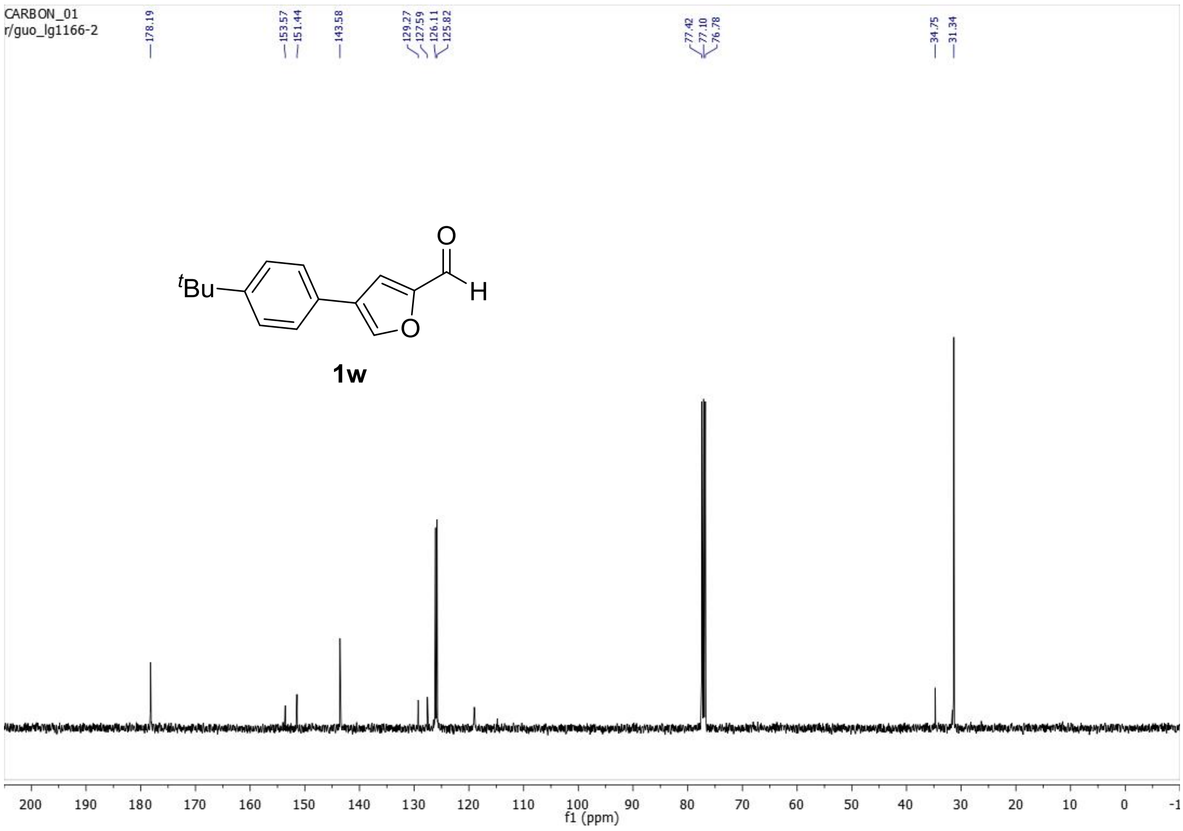

Supplementary Figure 14. ^{13}C NMR spectrum in $CDCl_3$ of compound **1w**.

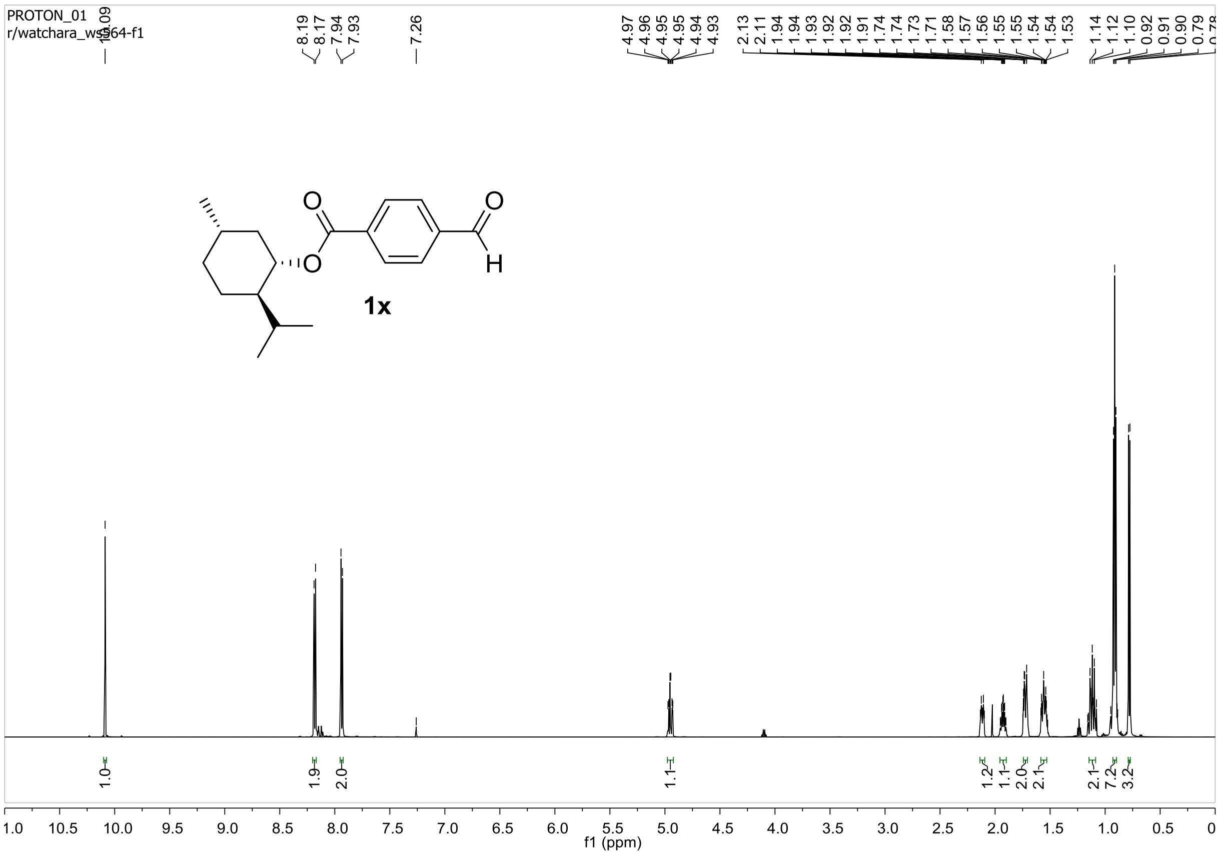

Supplementary Figure 15. ^{1}H NMR spectrum in $CDCl_3$ of compound **1x**.

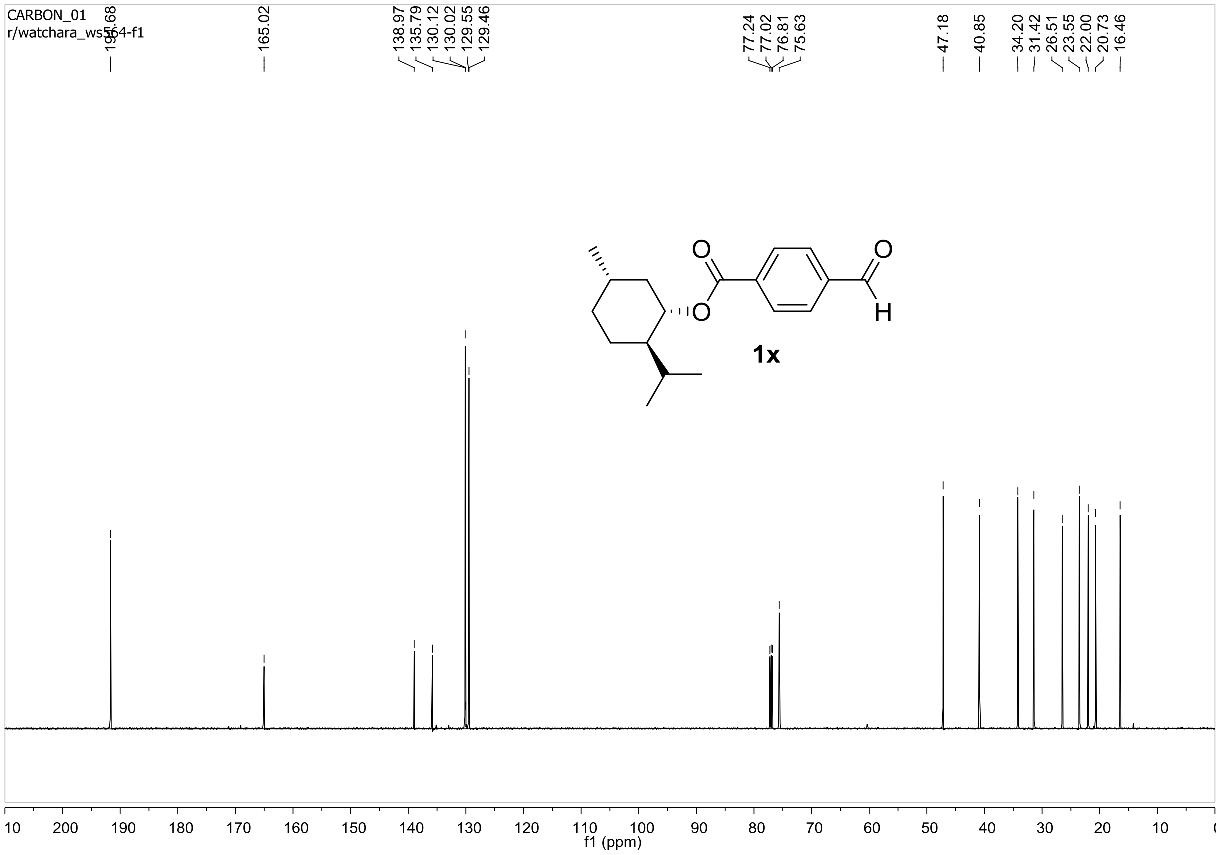

Supplementary Figure 16. ^{13}C NMR spectrum in $CDCl_3$ of compound **1x**.

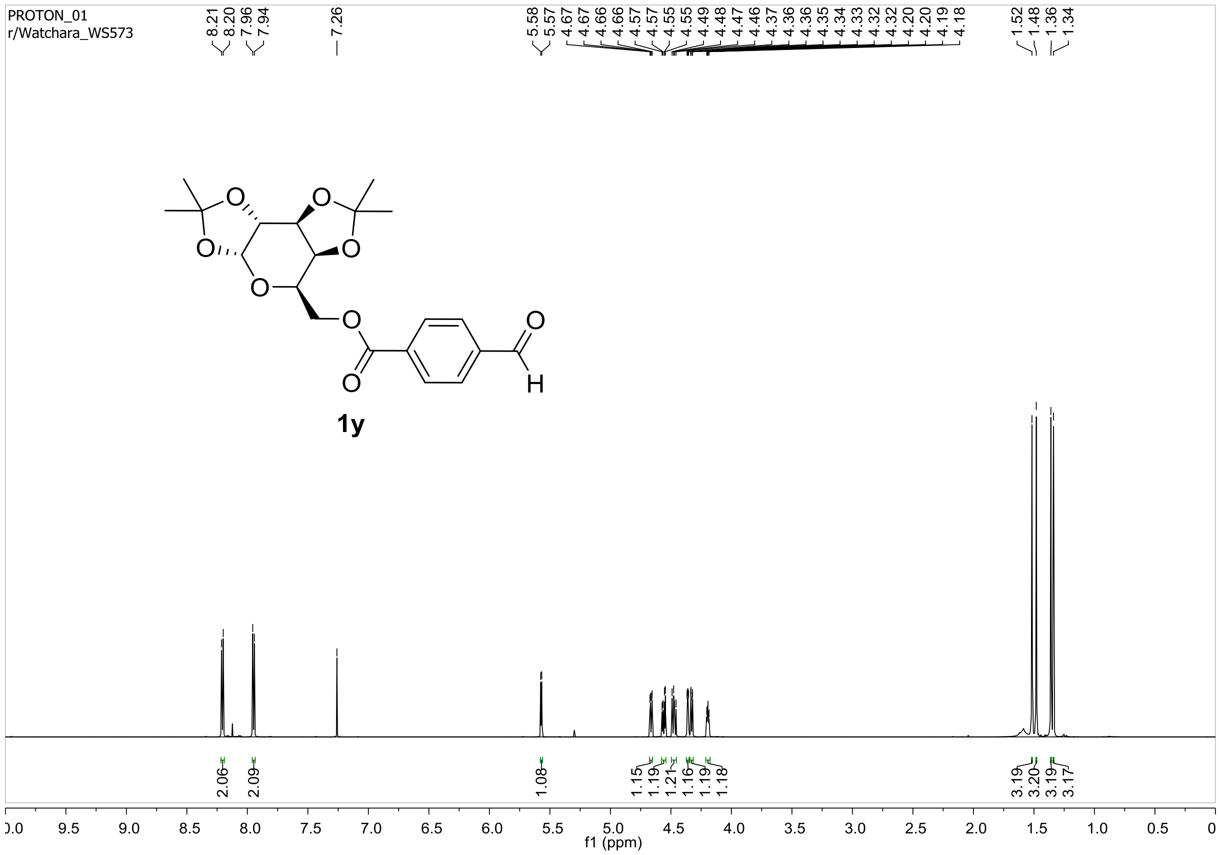

Supplementary Figure 17. ^{1}H NMR spectrum in $CDCl_3$ of compound **1y**.

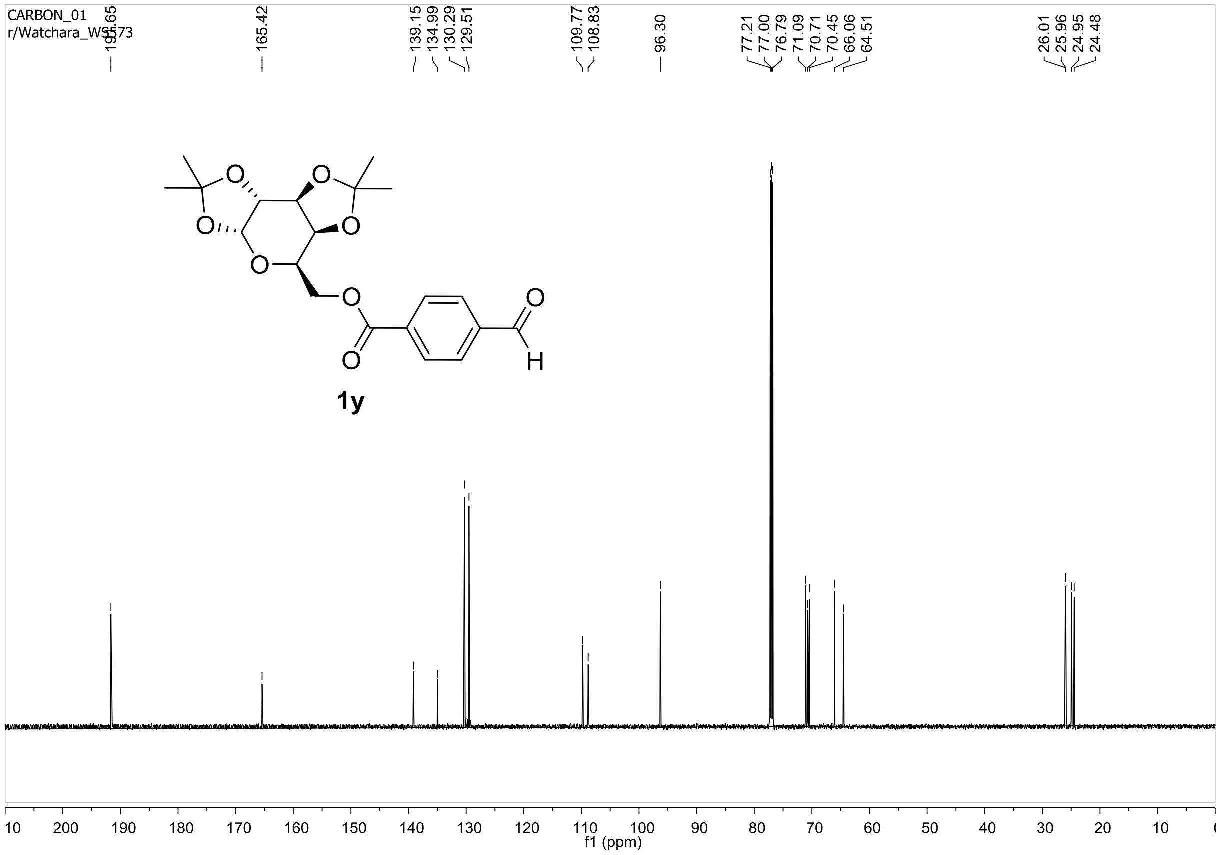

Supplementary Figure 18. ^{13}C NMR spectrum in $CDCl_3$ of compound **1y**.

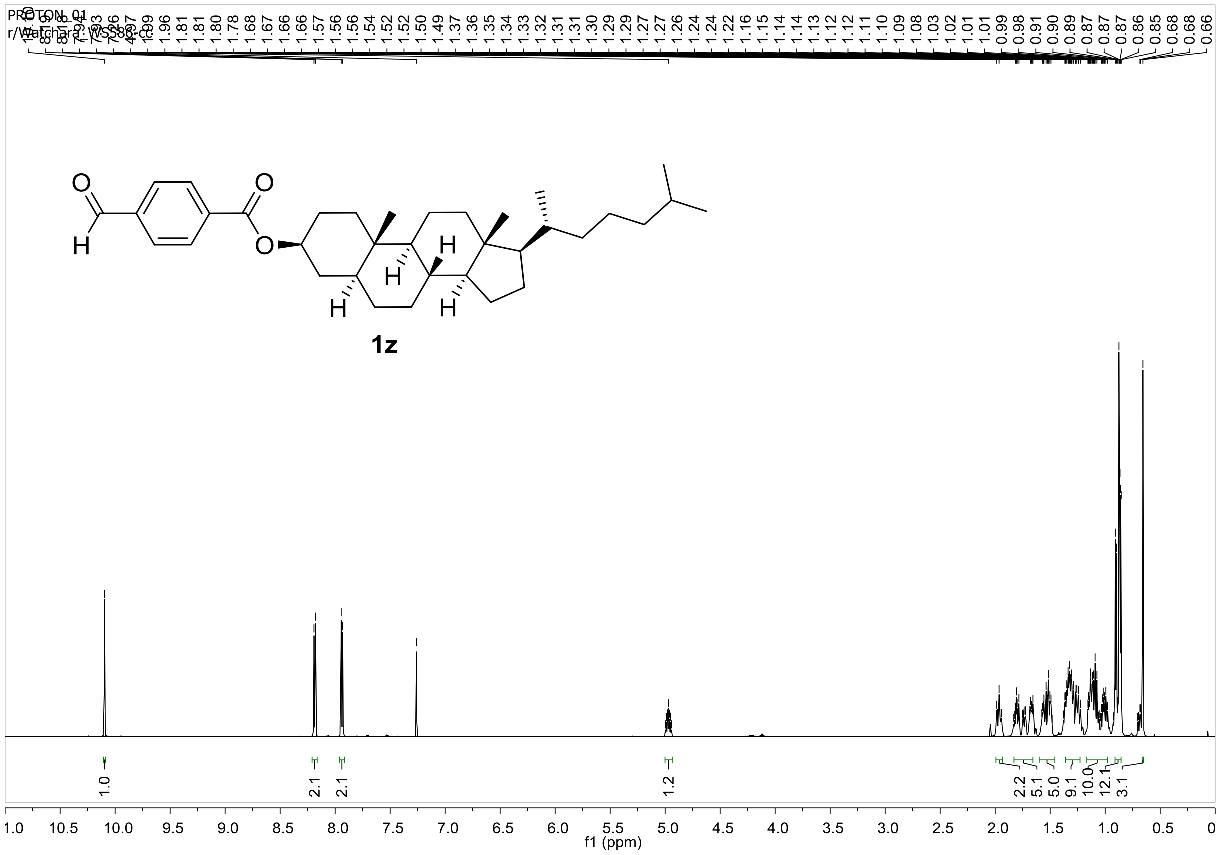

Supplementary Figure 19. ^{1}H NMR spectrum in $CDCl_3$ of compound **1z**.

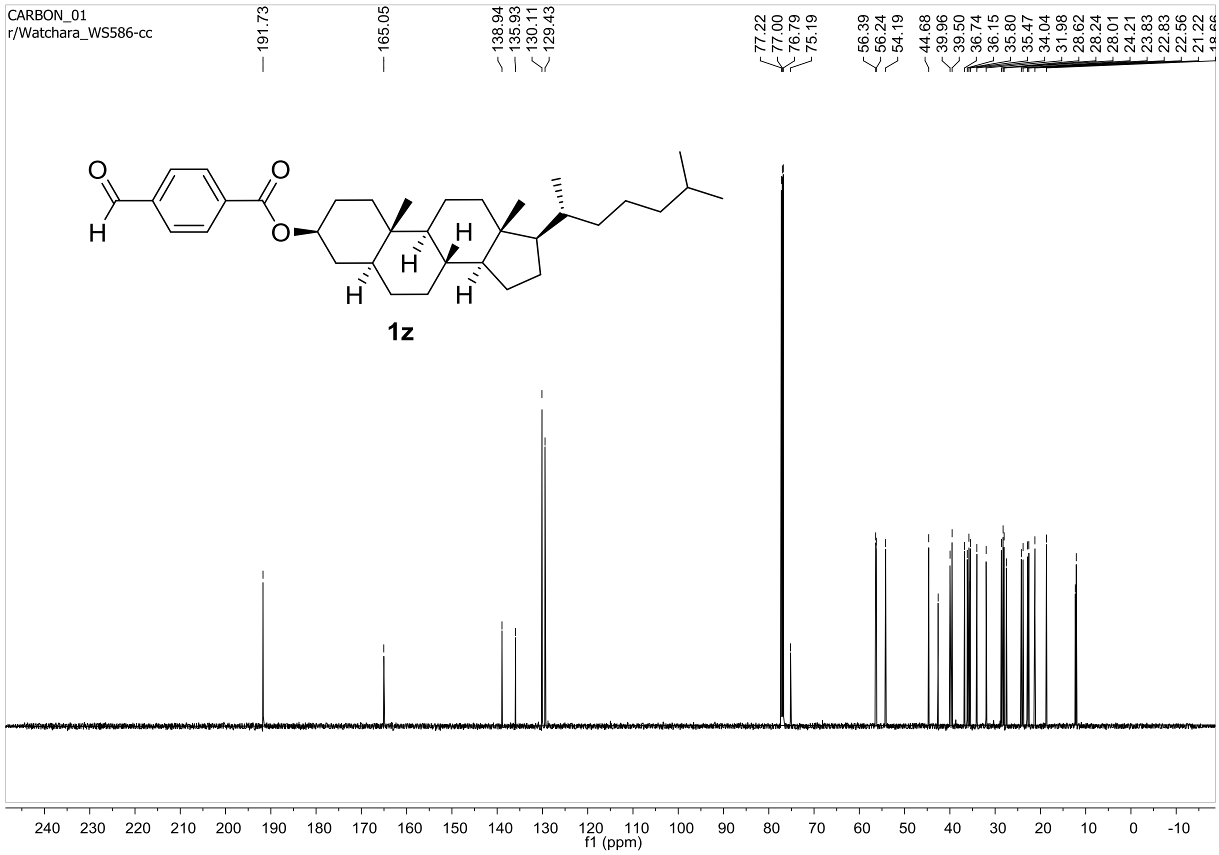

Supplementary Figure 20. ^{13}C NMR spectrum in $CDCl_3$ of compound **1z**.

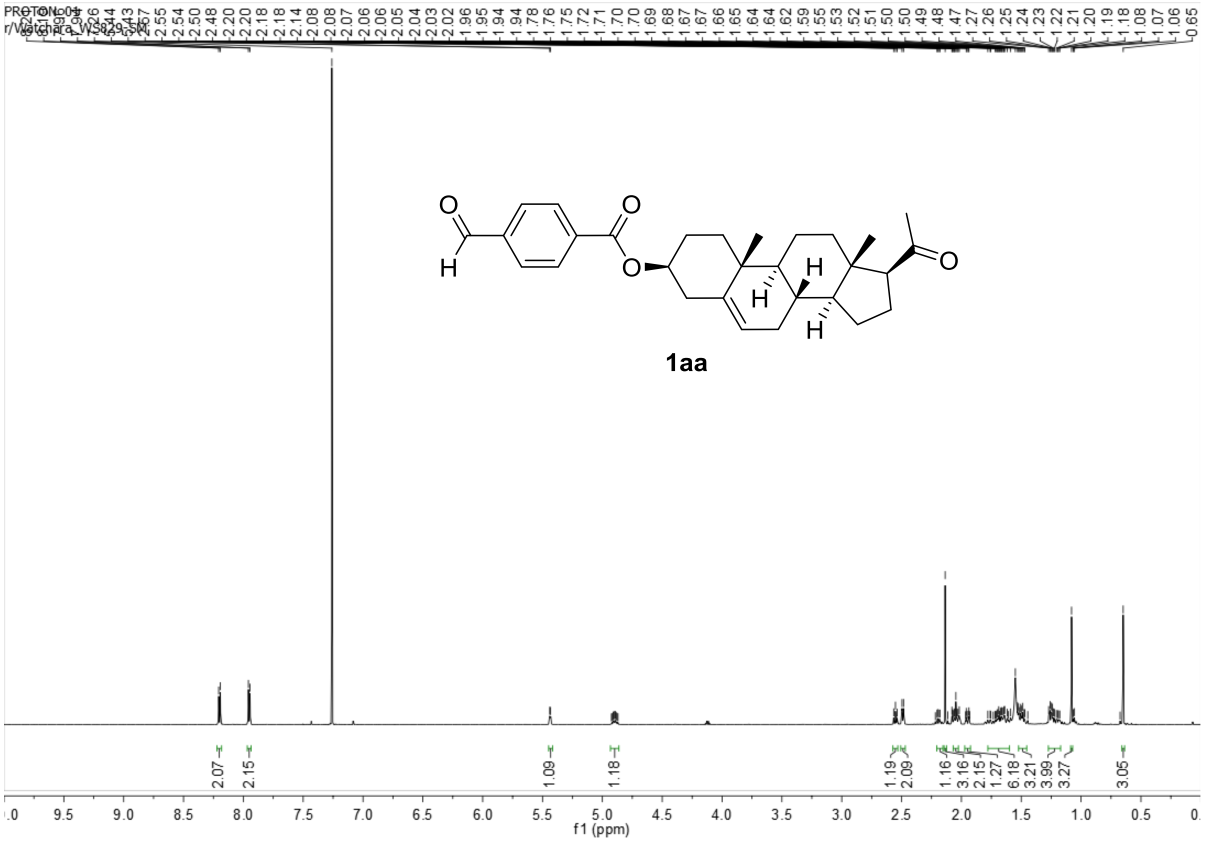

Supplementary Figure 21. ^{1}H NMR spectrum in $CDCl_3$ of compound **1aa**.

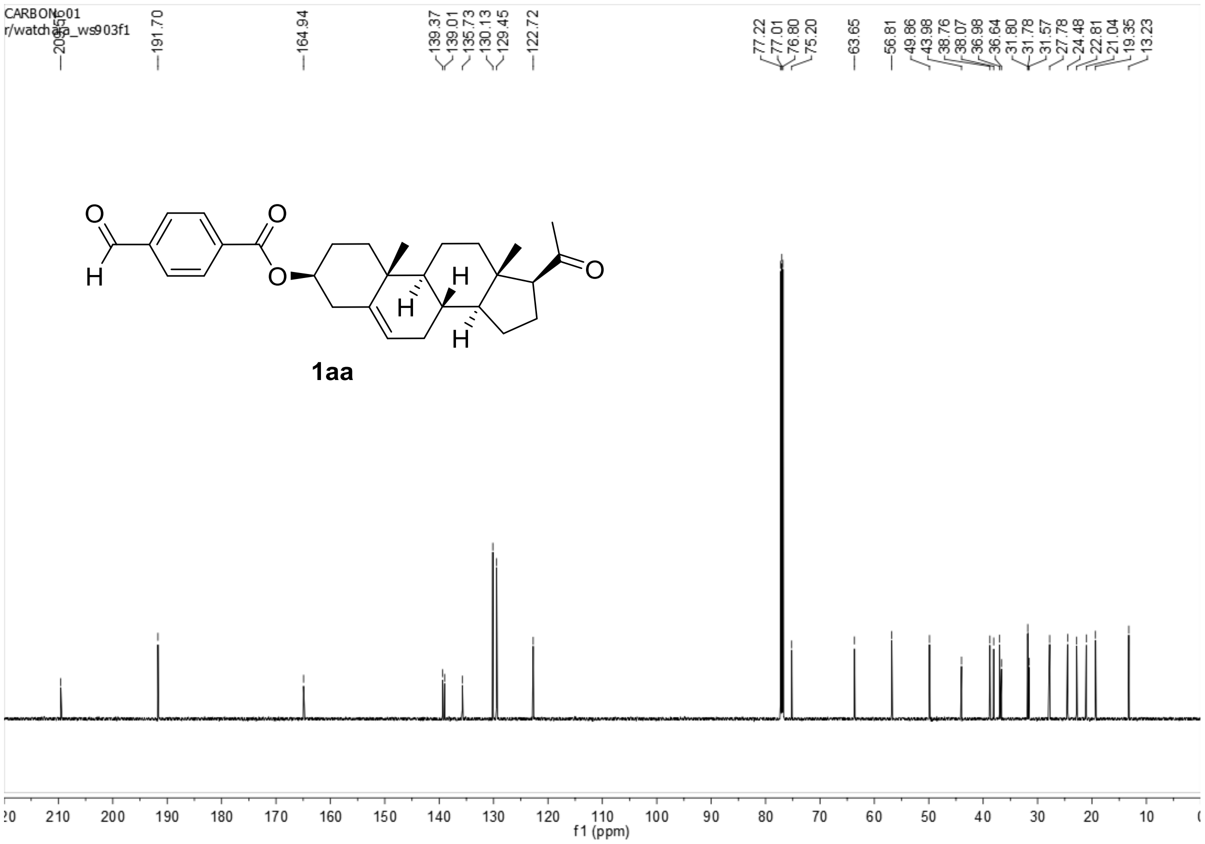

Supplementary Figure 22. ^{13}C NMR spectrum in $CDCl_3$ of compound **1aa**.

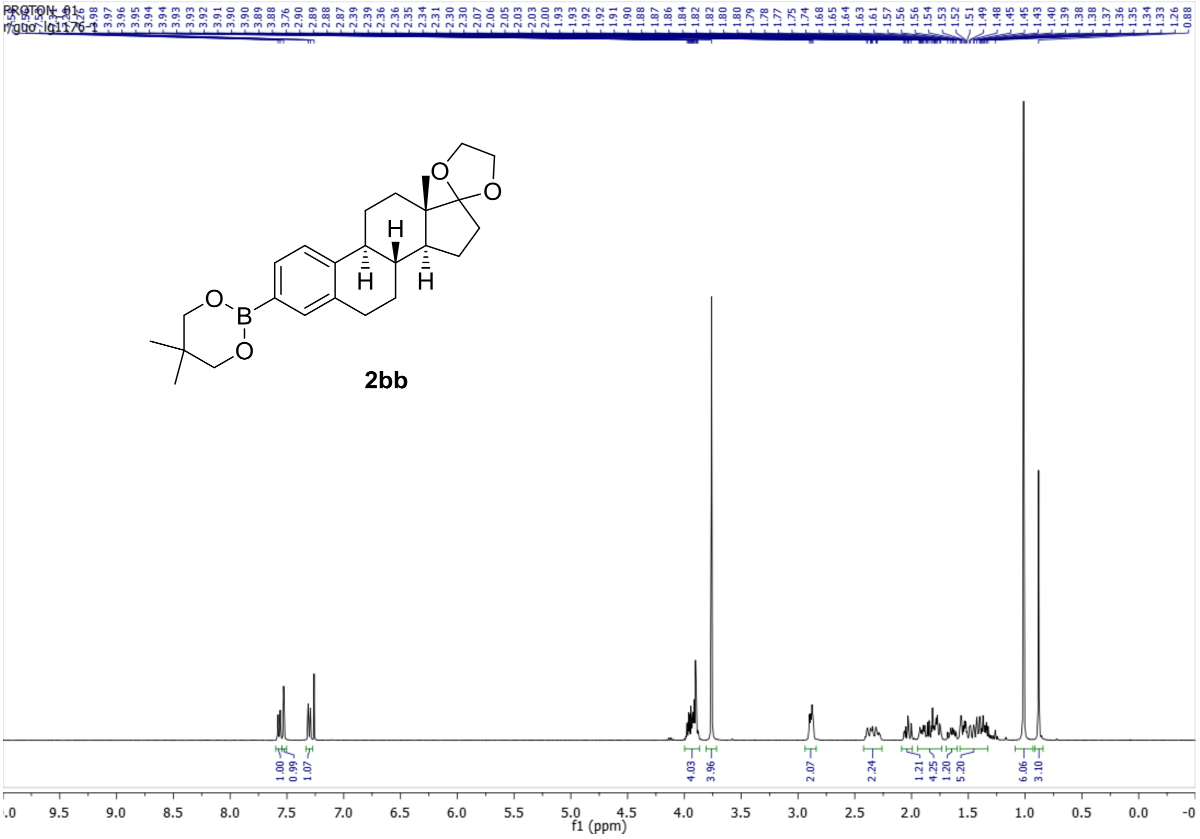

Supplementary Figure 23. ^{1}H NMR spectrum in $CDCl_3$ of compound **2bb**.

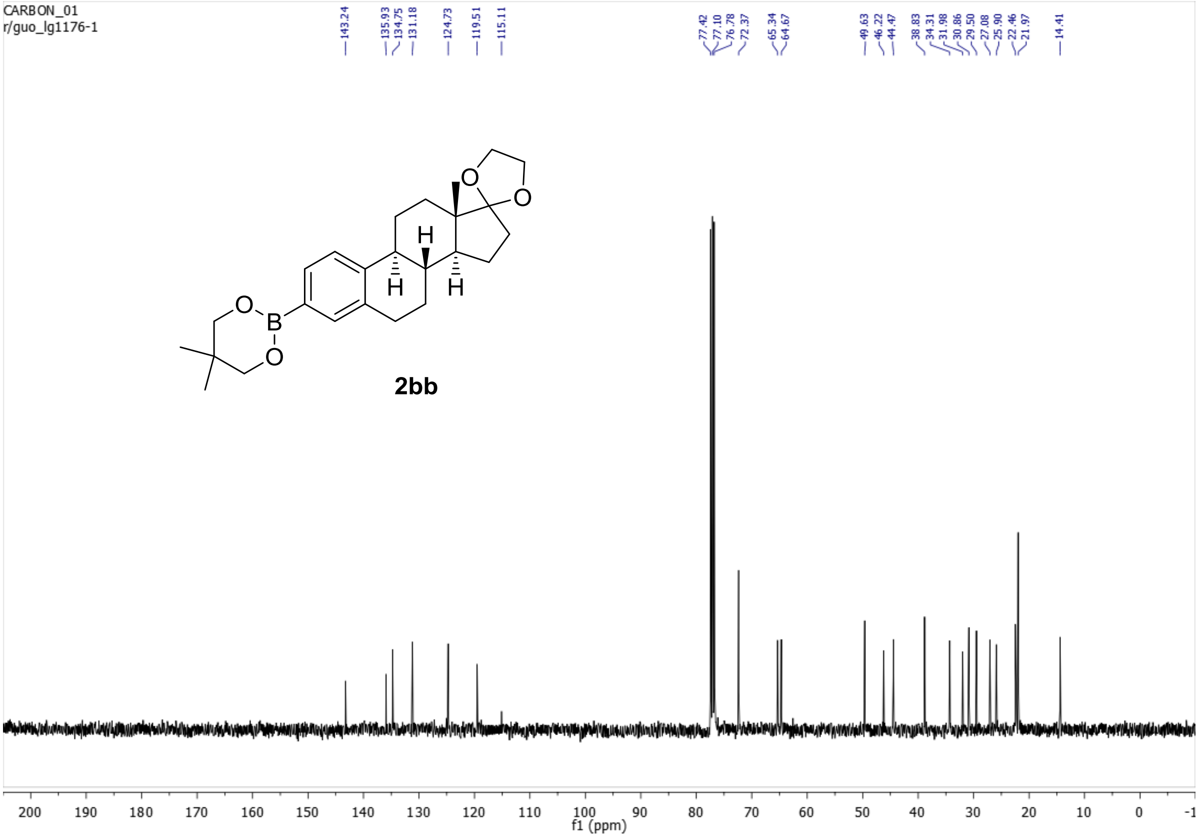

Supplementary Figure 24. ^{13}C NMR spectrum in $CDCl_3$ of compound **2bb**.

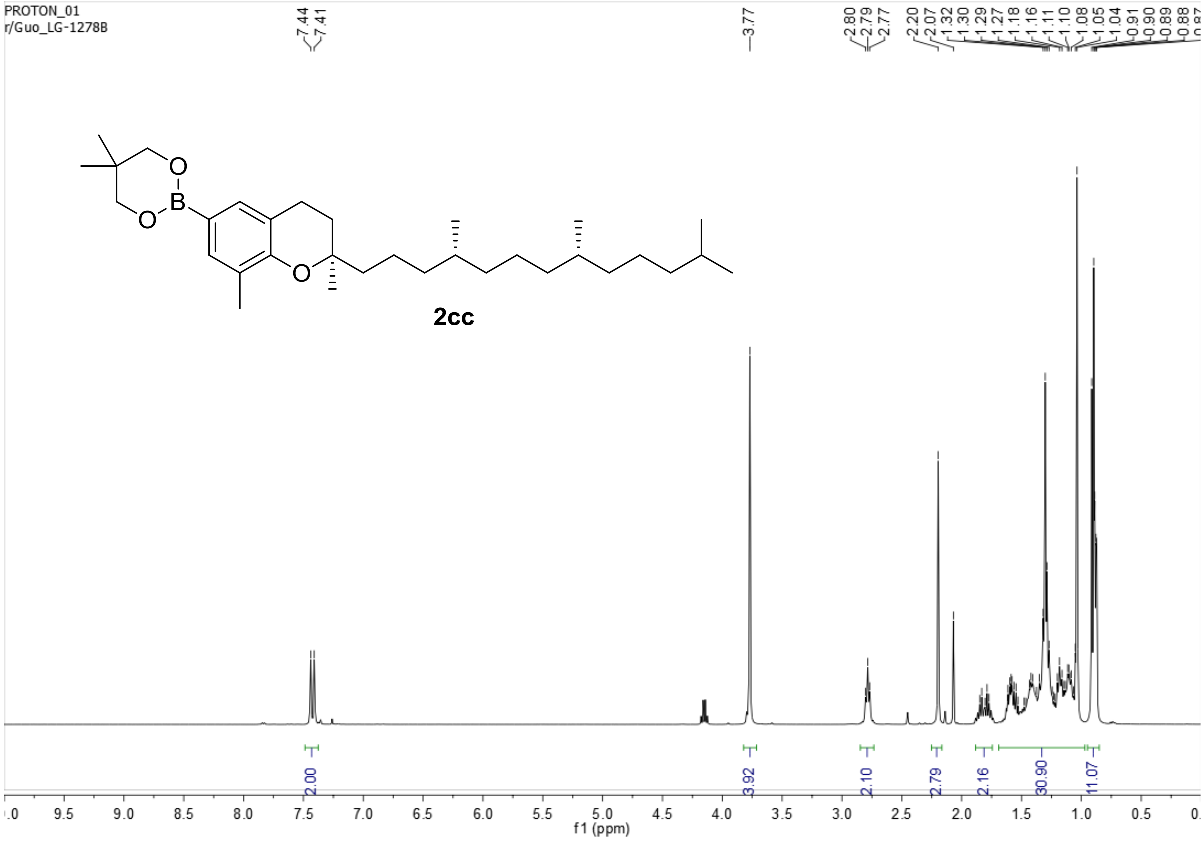

Supplementary Figure 25. ^{1}H NMR spectrum in $CDCl_3$ of compound **2cc**.

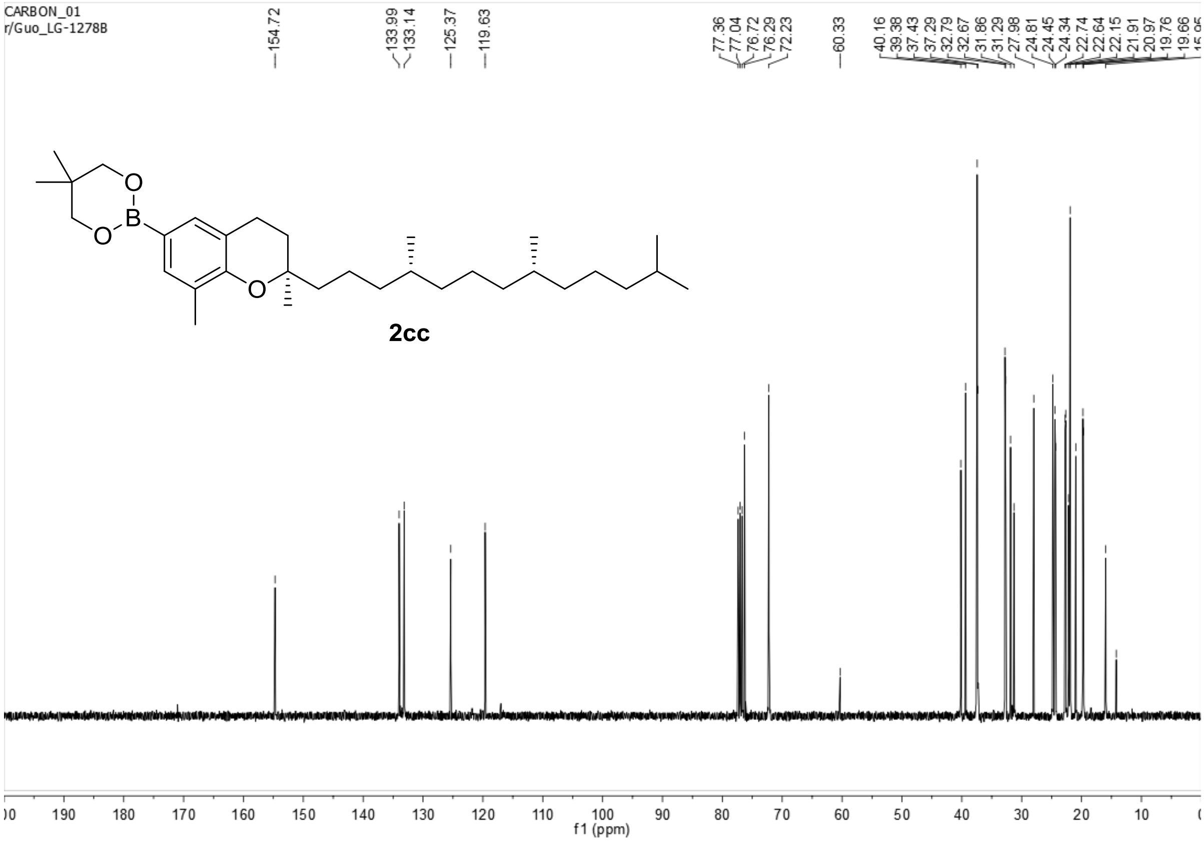

Supplementary Figure 26. ^{13}C NMR spectrum in $CDCl_3$ of compound **2cc**.

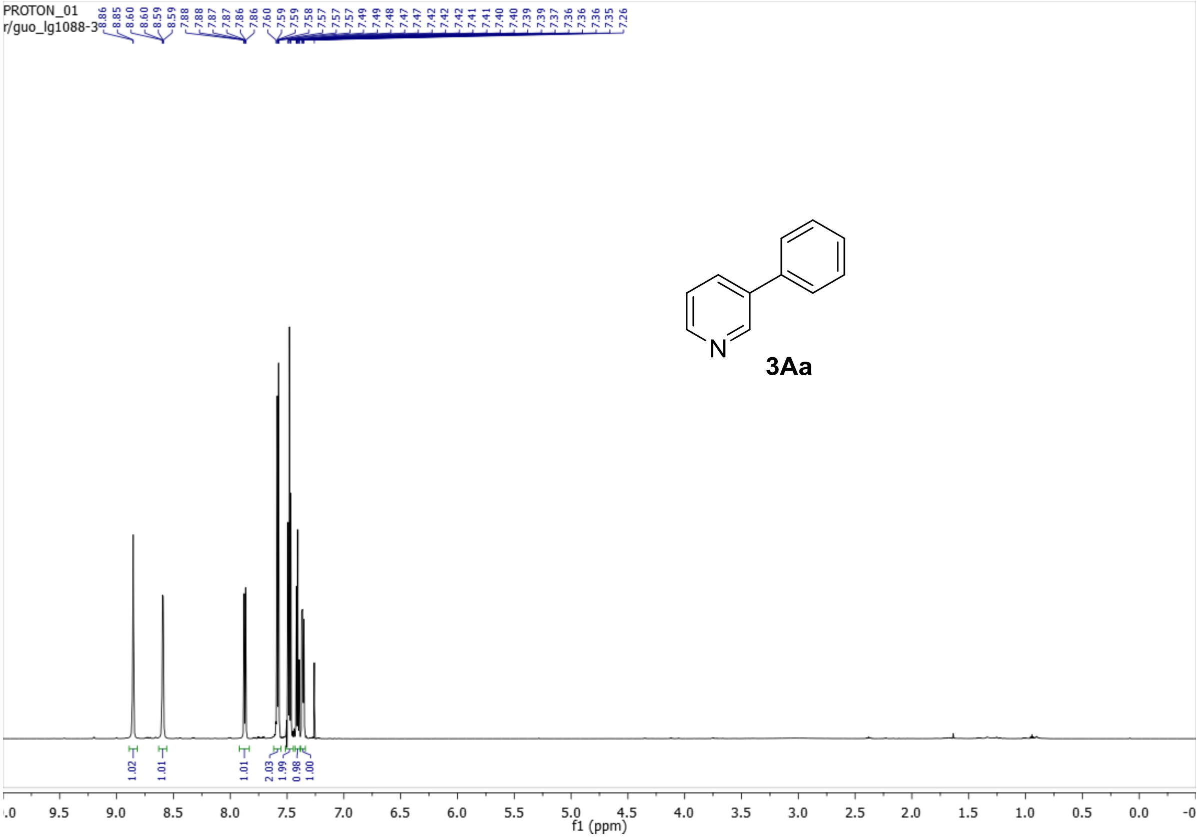

Supplementary Figure 27. ^{1}H NMR spectrum in $CDCl_3$ of compound **3Aa**.

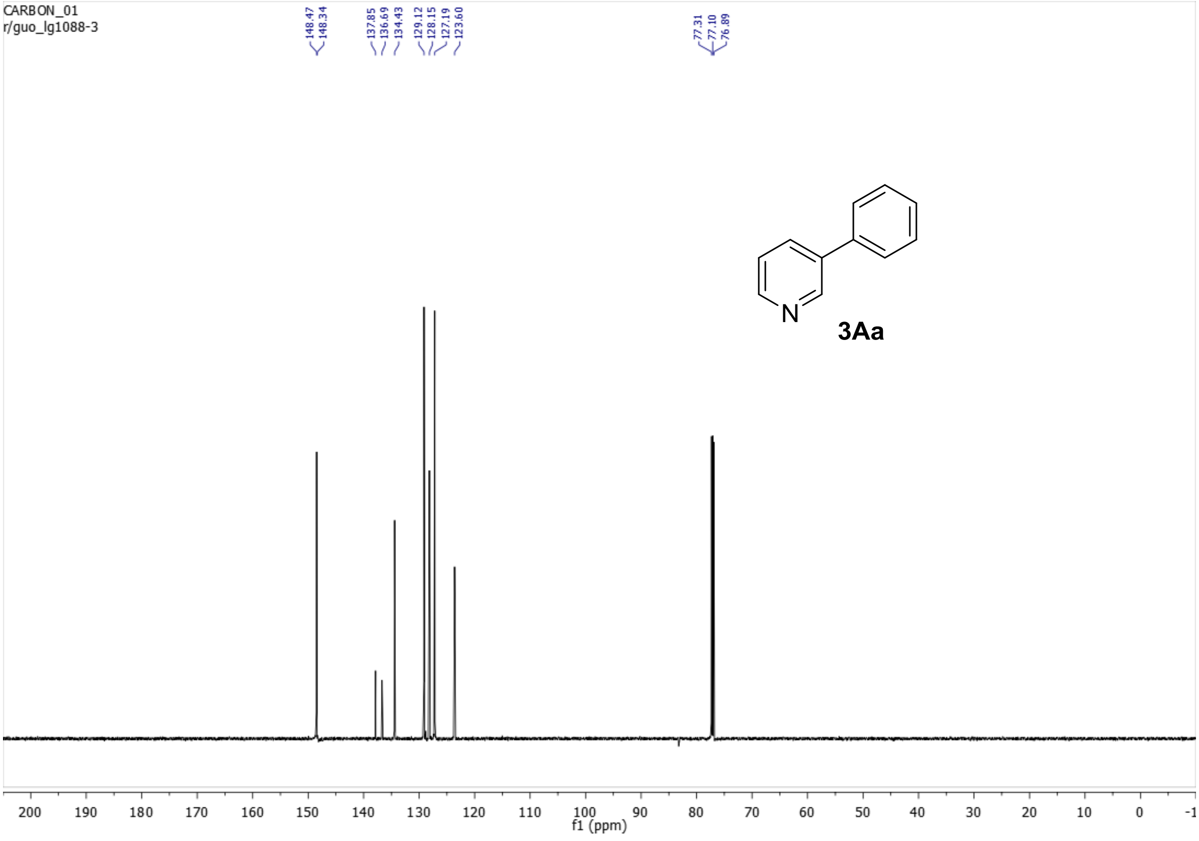

Supplementary Figure 28. ^{13}C NMR spectrum in $CDCl_3$ of compound **3Aa**.

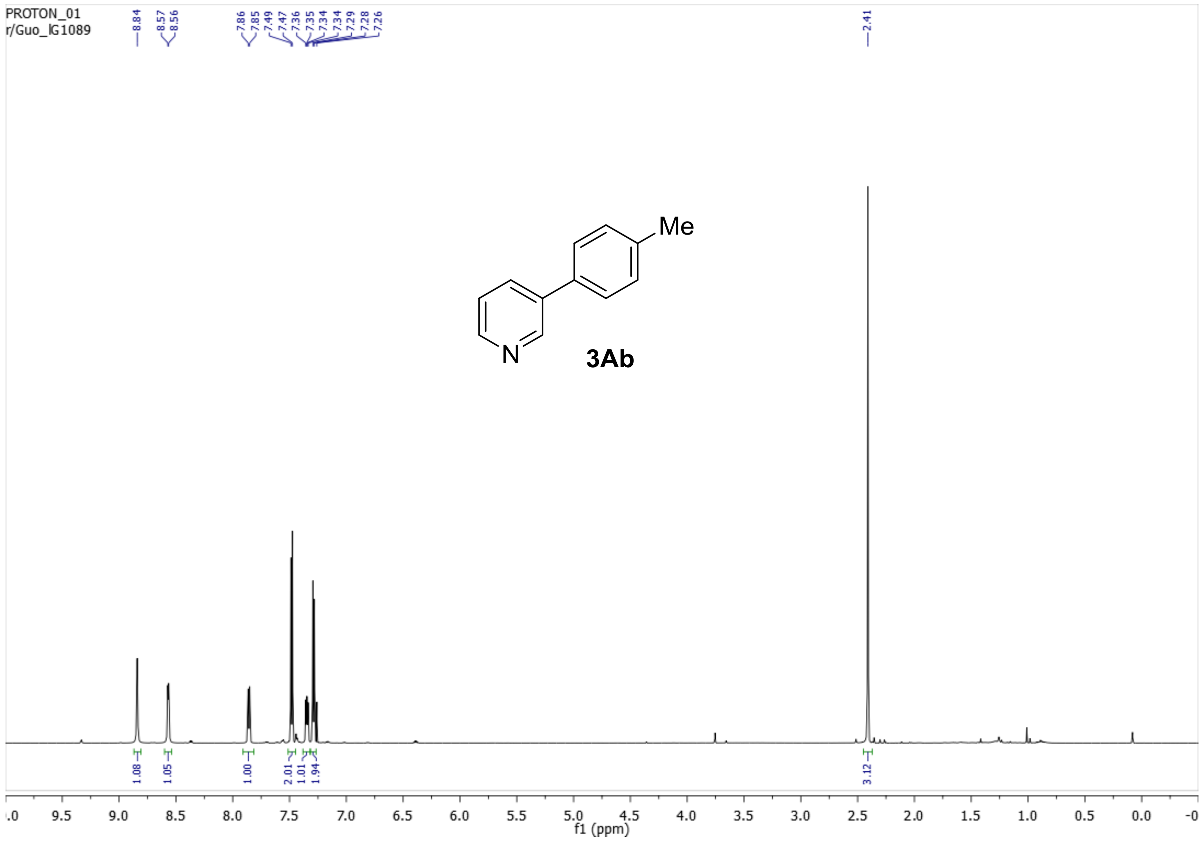

Supplementary Figure 29. ^{1}H NMR spectrum in $CDCl_3$ of compound **3Ab**.

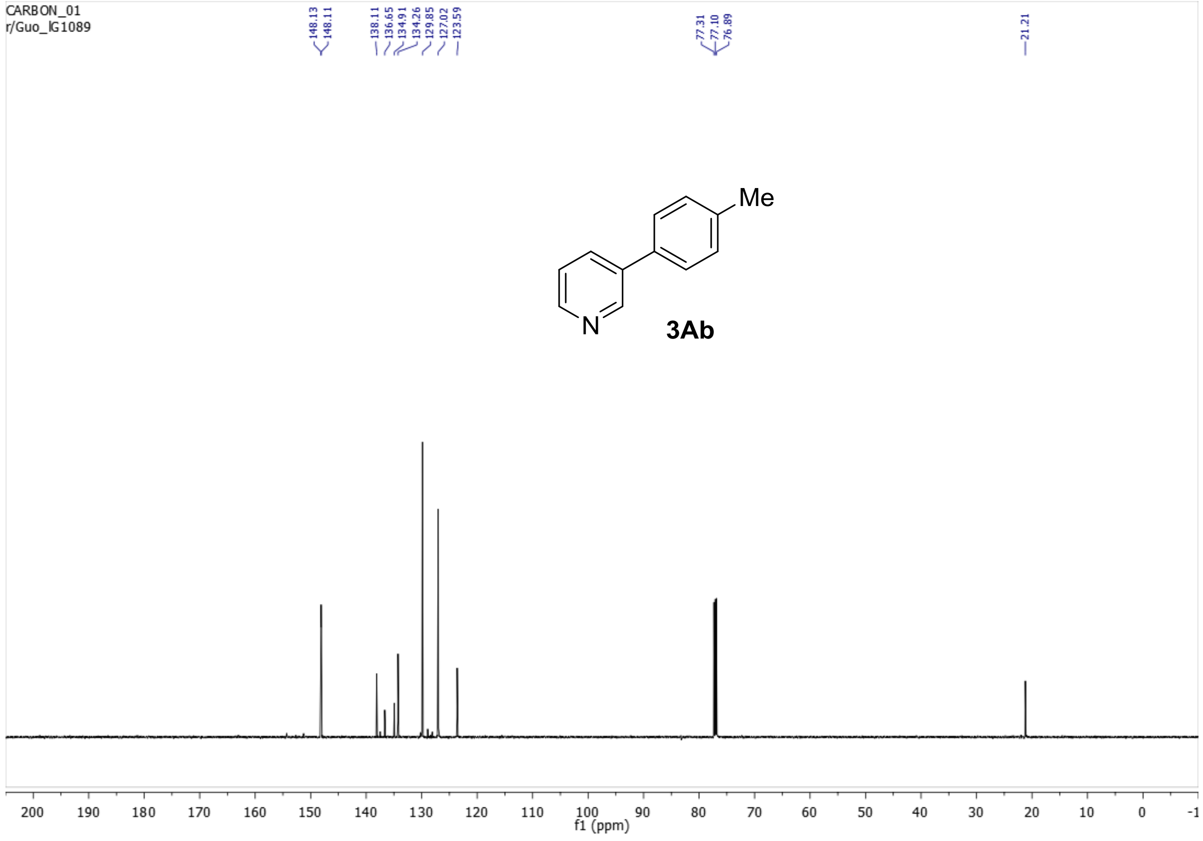

Supplementary Figure 30. ^{13}C NMR spectrum in $CDCl_3$ of compound **3Ab**.

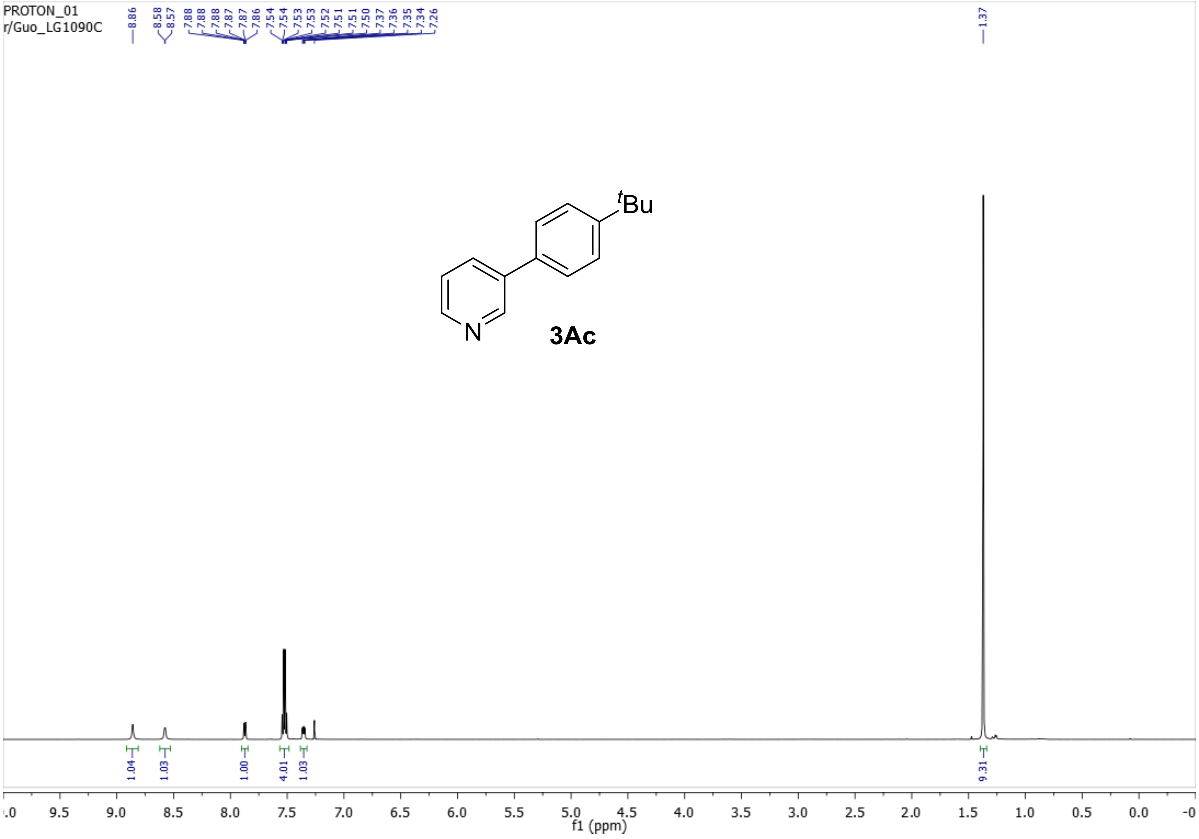

Supplementary Figure 31. ^{1}H NMR spectrum in $CDCl_3$ of compound **3Ac**.

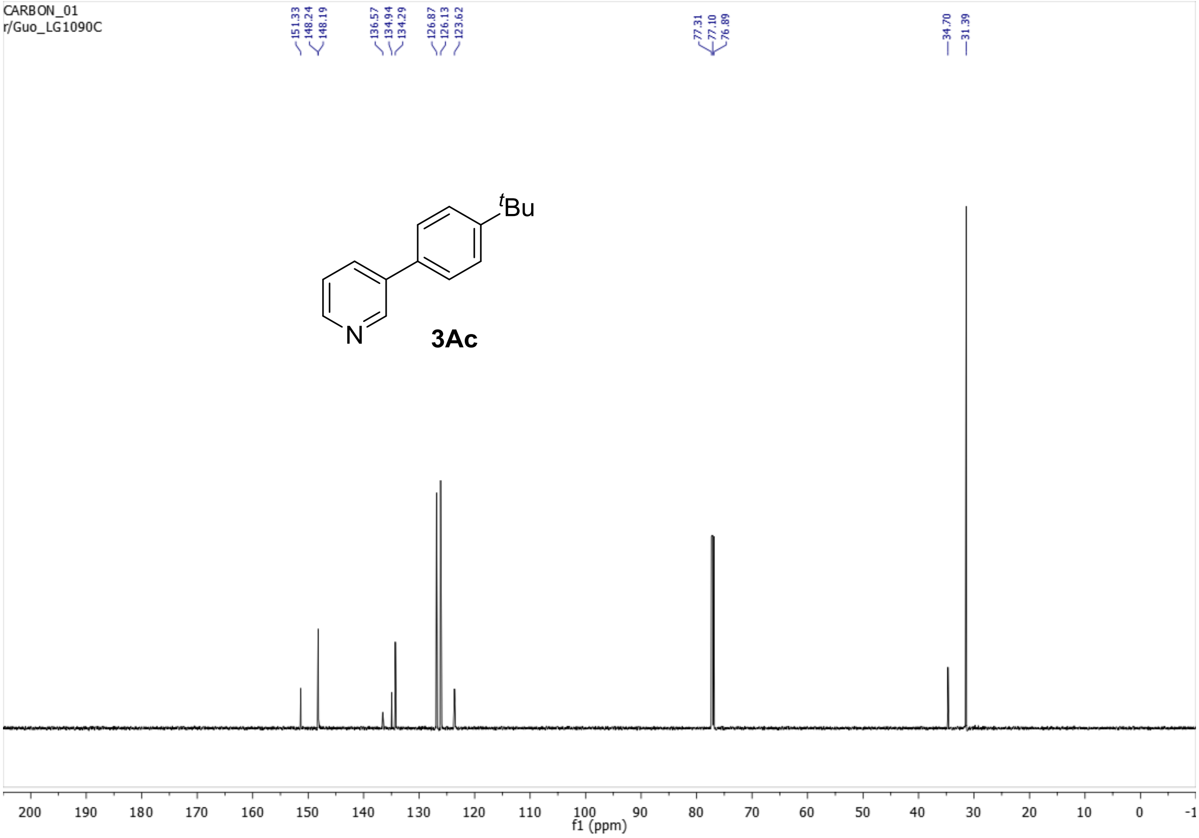

Supplementary Figure 32. ^{13}C NMR spectrum in $CDCl_3$ of compound **3Ac**.

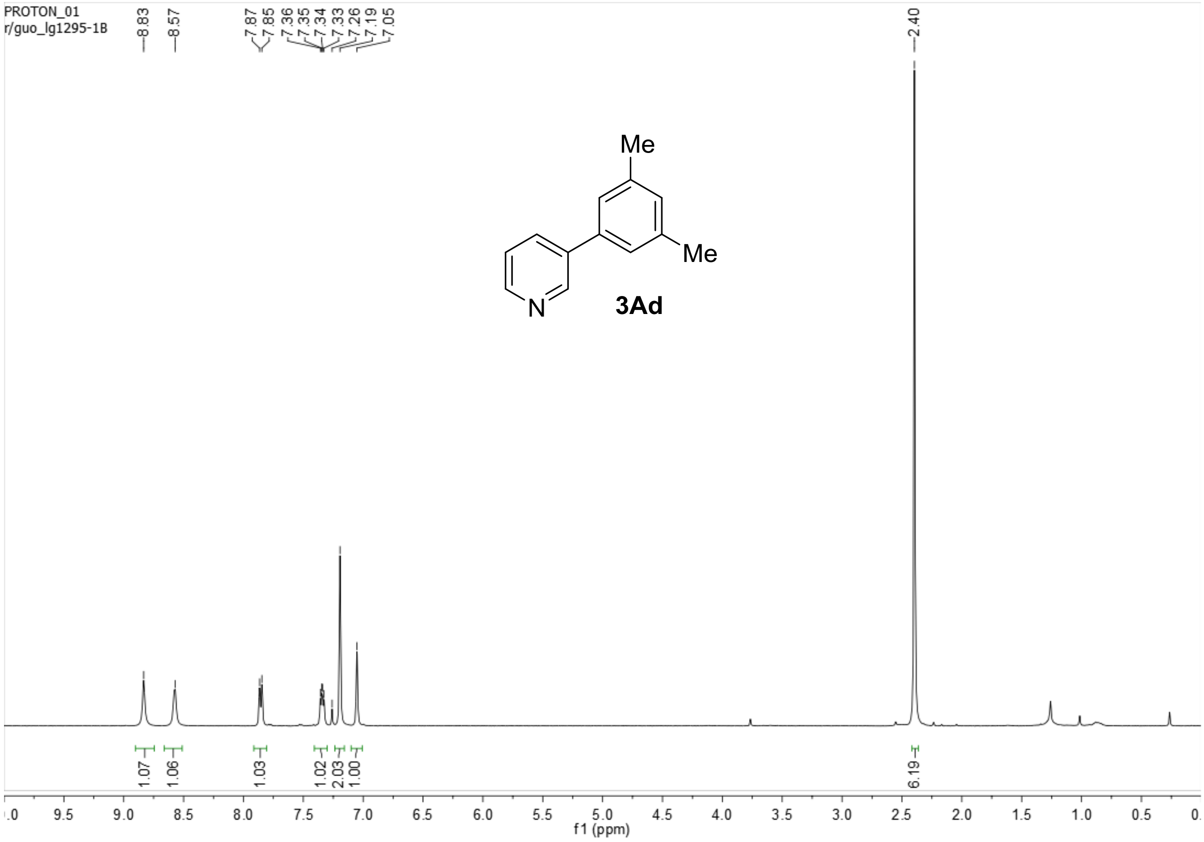

Supplementary Figure 33. ^{1}H NMR spectrum in $CDCl_3$ of compound **3Ad**.

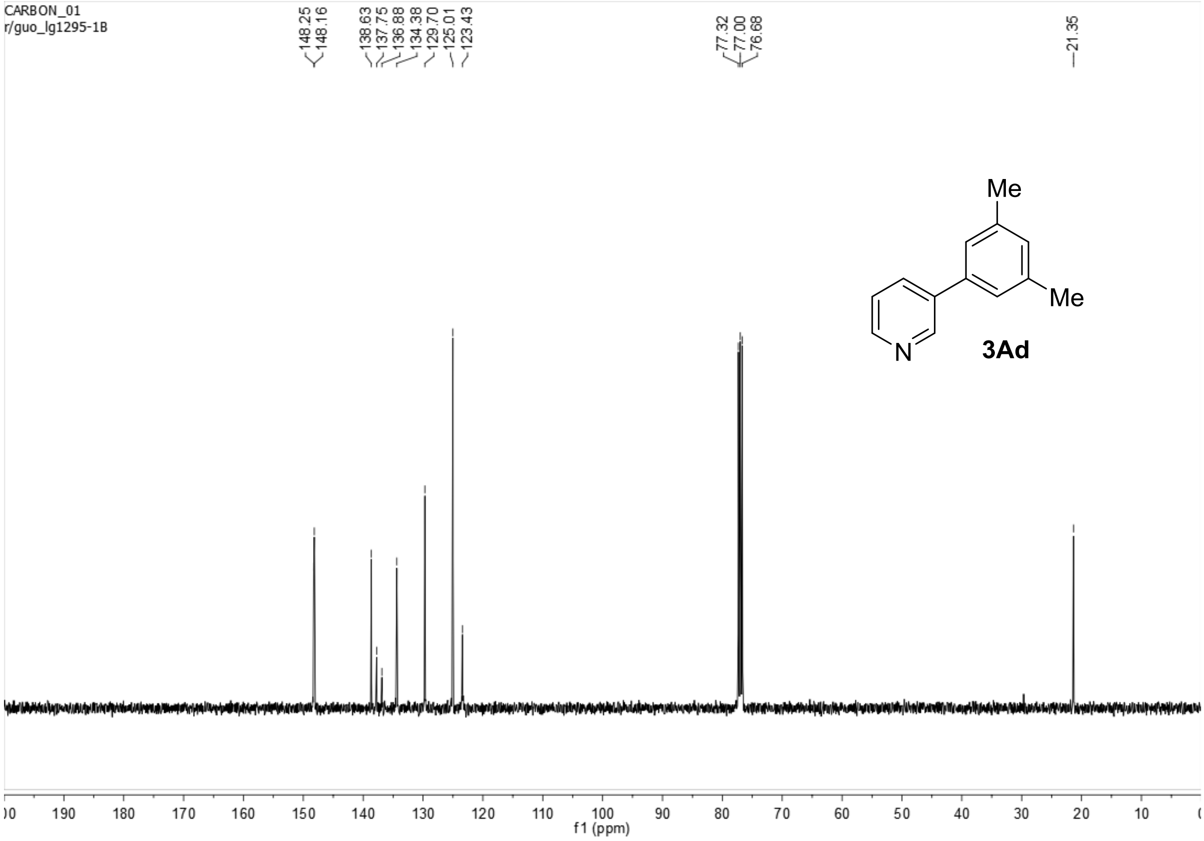

Supplementary Figure 34. ^{13}C NMR spectrum in $CDCl_3$ of compound **3Ad**.

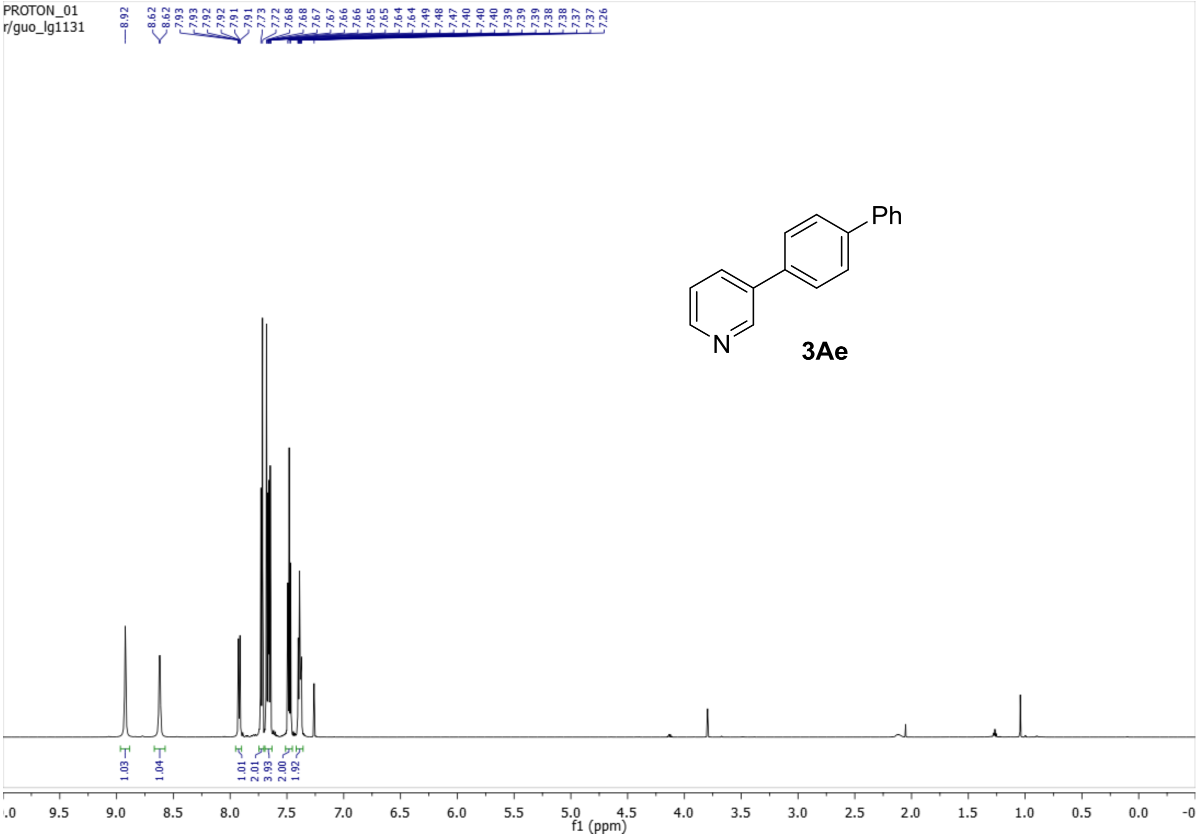

Supplementary Figure 35. ^{1}H NMR spectrum in $CDCl_3$ of compound **3Ae**.

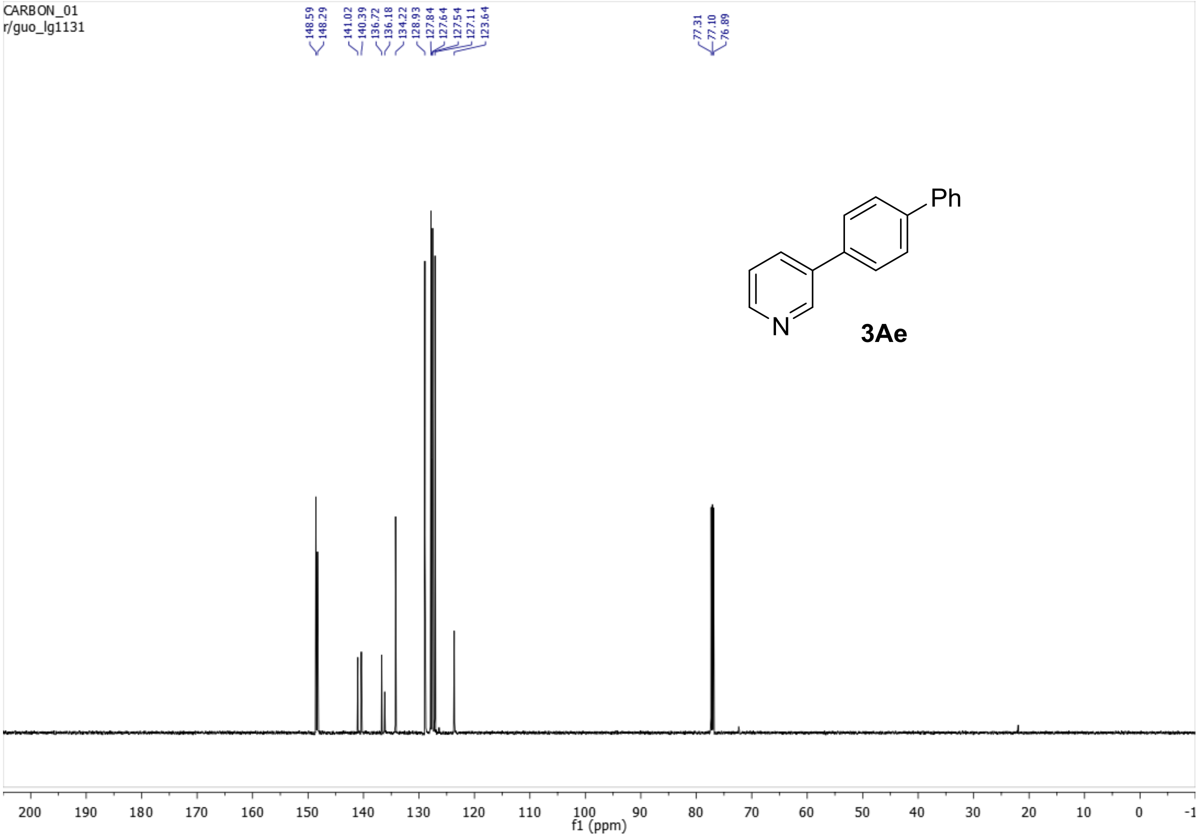

Supplementary Figure 36. ^{13}C NMR spectrum in $CDCl_3$ of compound **3Ae**.

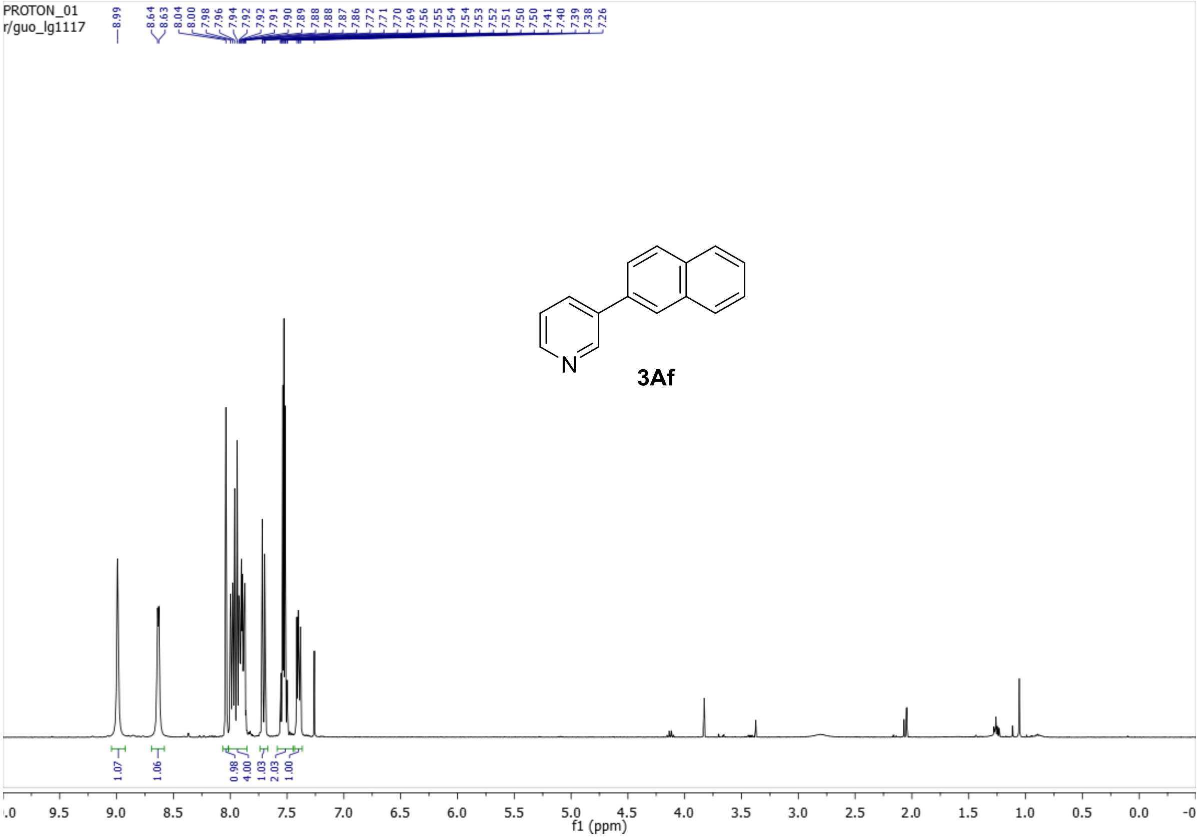

Supplementary Figure 37. ^{1}H NMR spectrum in $CDCl_3$ of compound **3Af**.

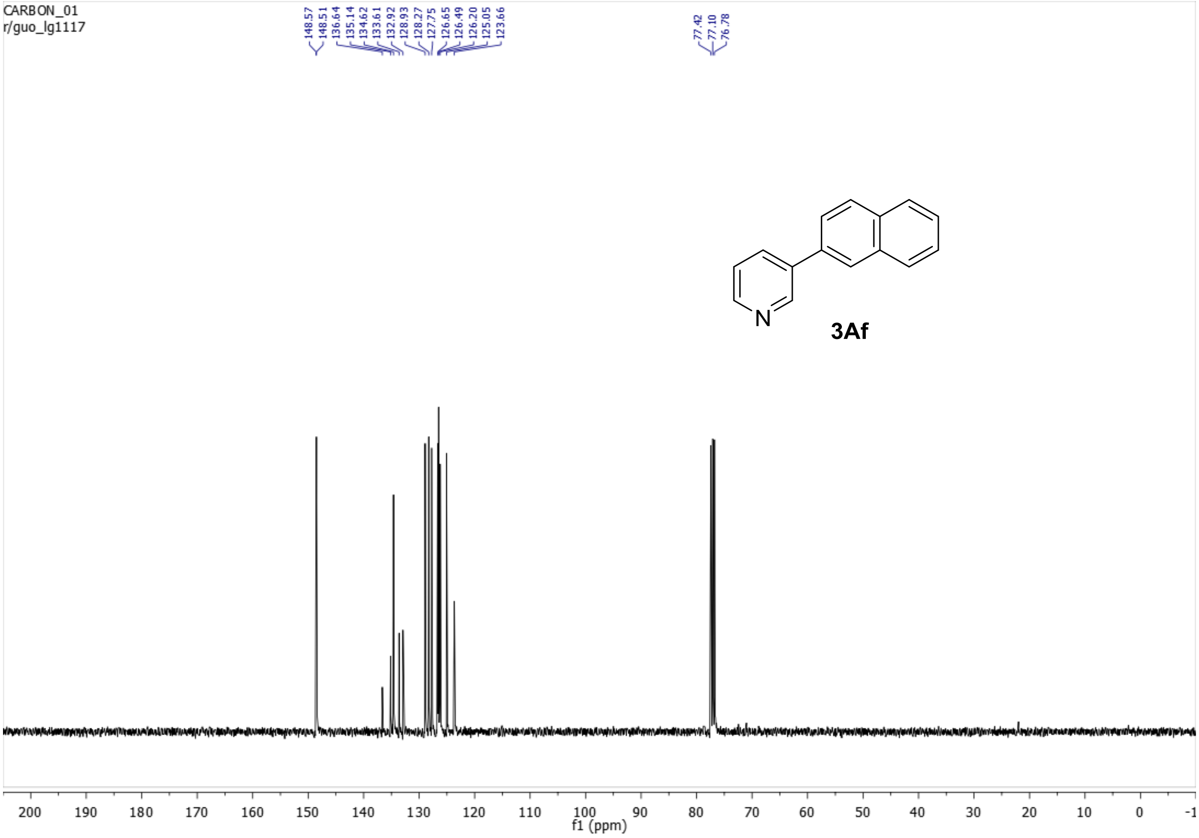

Supplementary Figure 38. ^{13}C NMR spectrum in $CDCl_3$ of compound **3Af**.

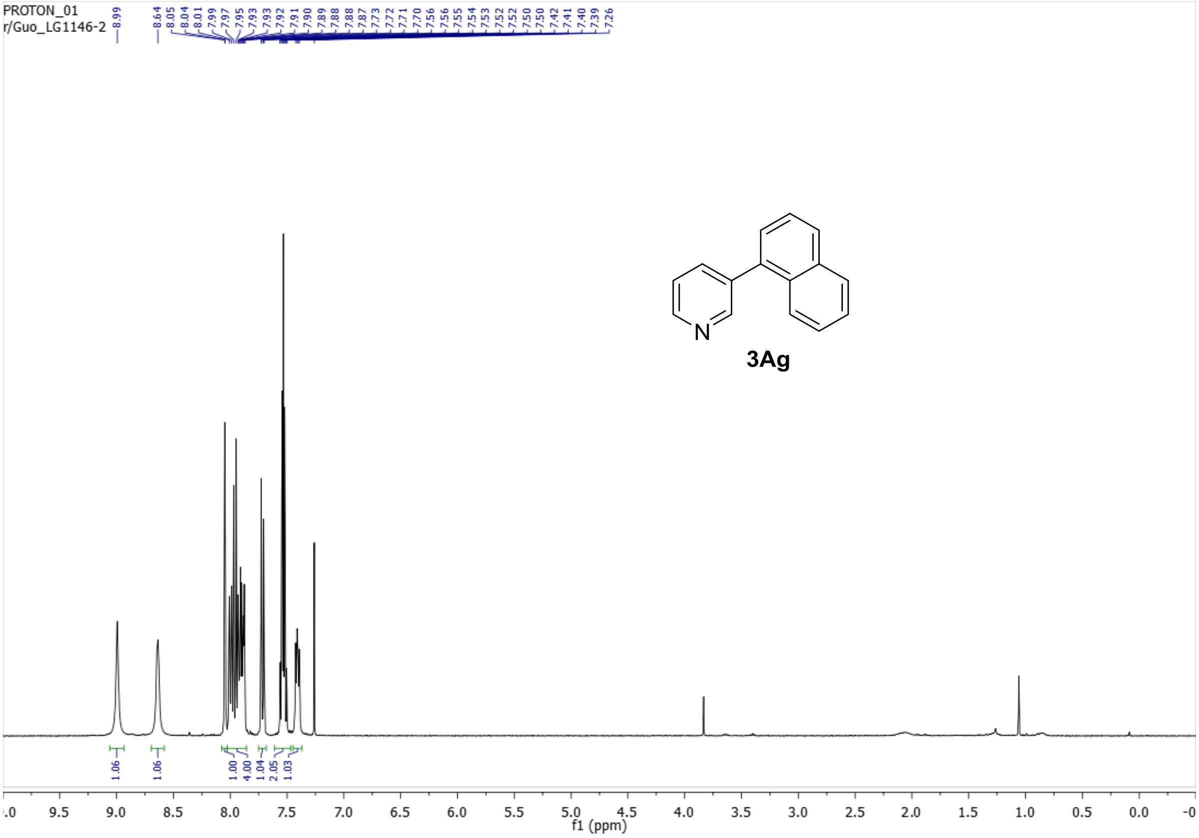

Supplementary Figure 39. ^{1}H NMR spectrum in $CDCl_3$ of compound **3Ag**.

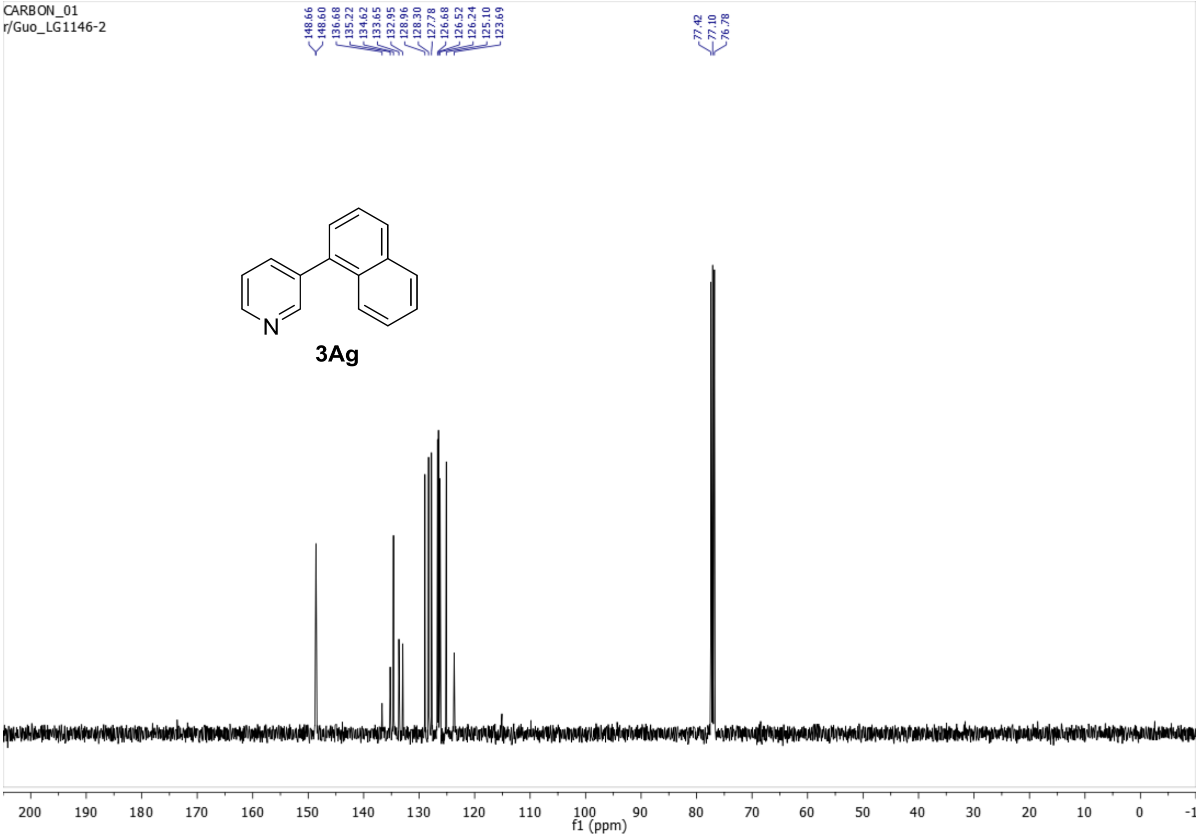

Supplementary Figure 40. ^{13}C NMR spectrum in $CDCl_3$ of compound **3Ag**.

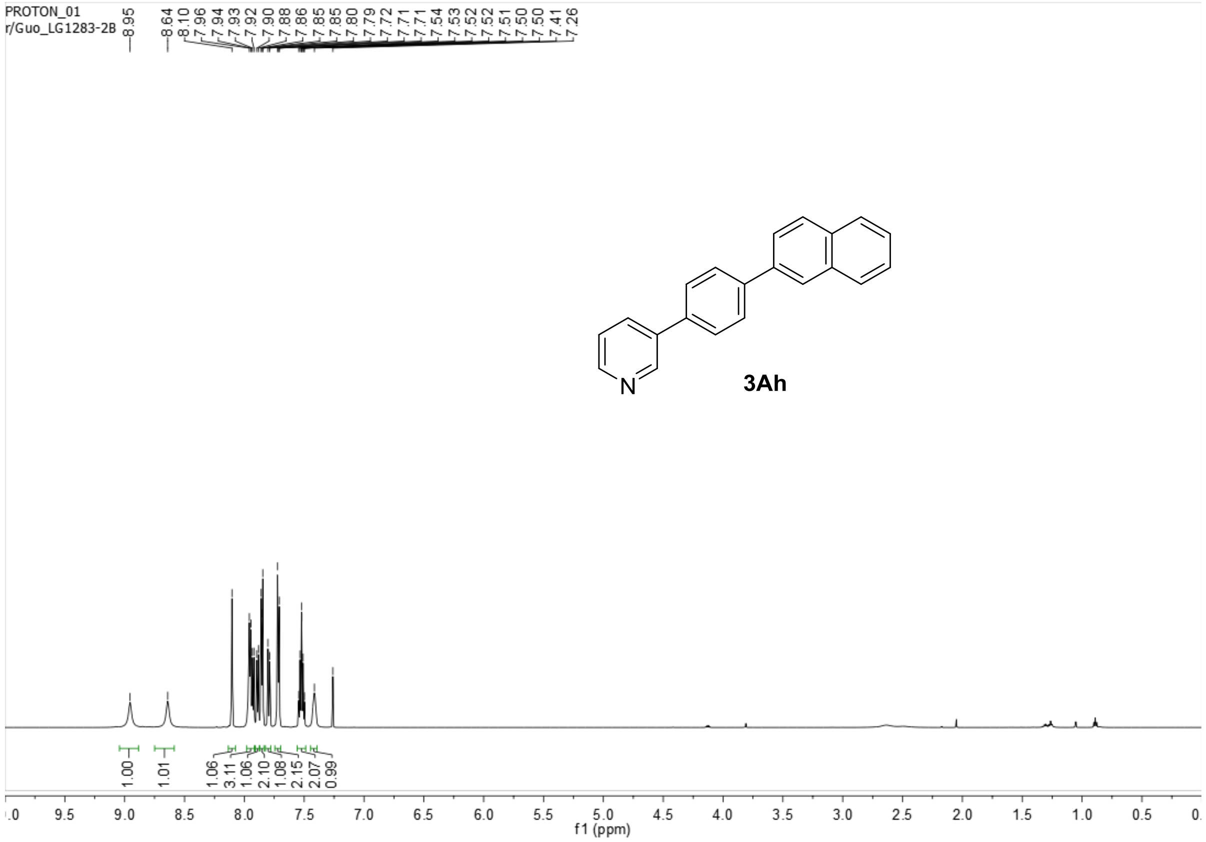

Supplementary Figure 41. ^{1}H NMR spectrum in $CDCl_3$ of compound **3Ah**.

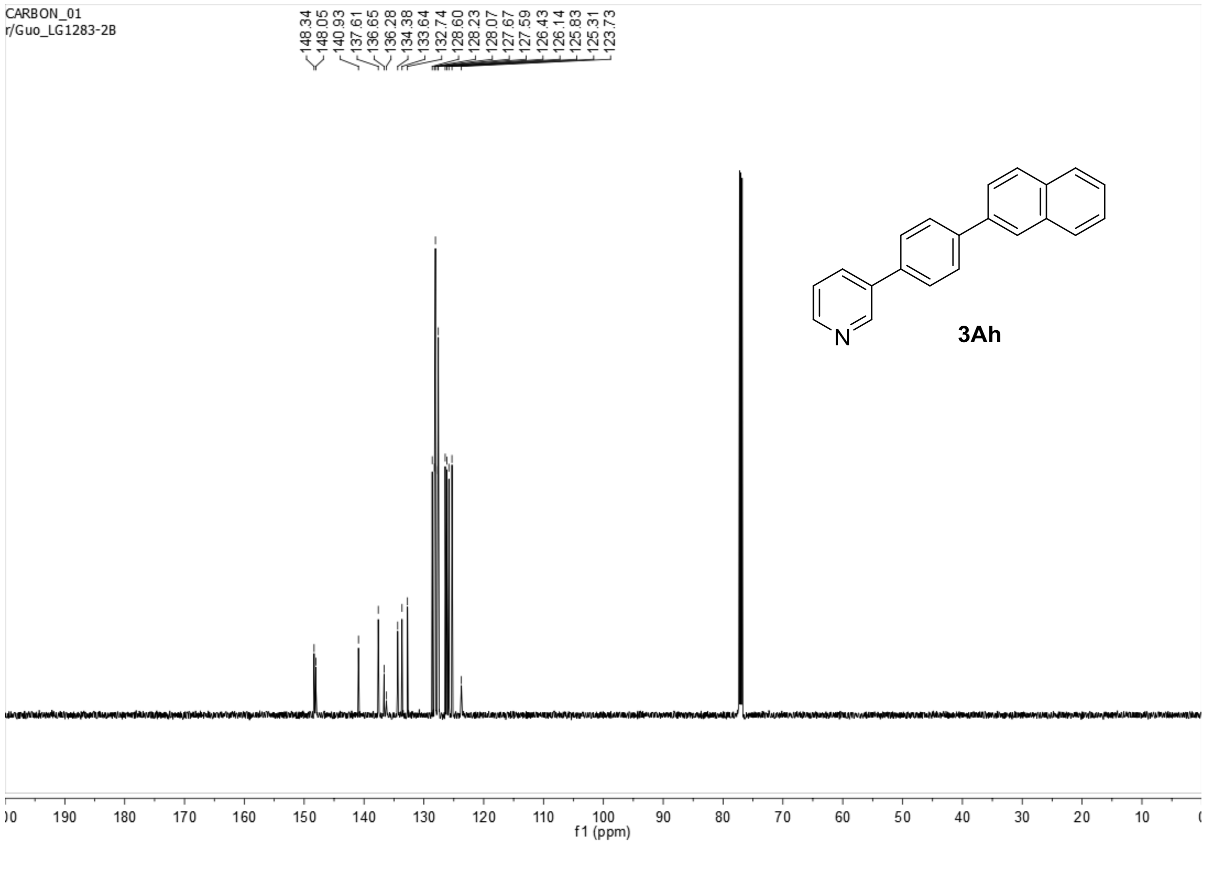

Supplementary Figure 42. ^{13}C NMR spectrum in $CDCl_3$ of compound **3Ah**.

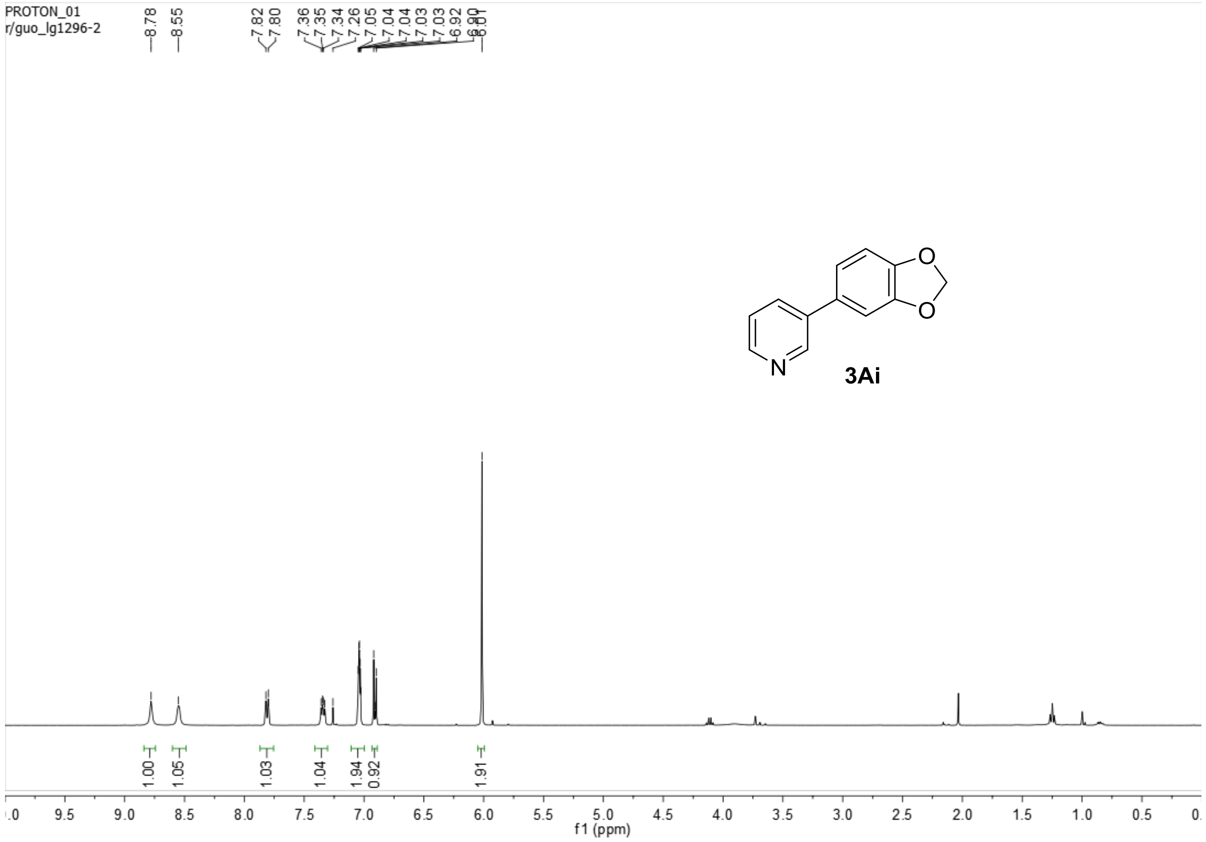

Supplementary Figure 43. ^{1}H NMR spectrum in $CDCl_3$ of compound **3Ai**.

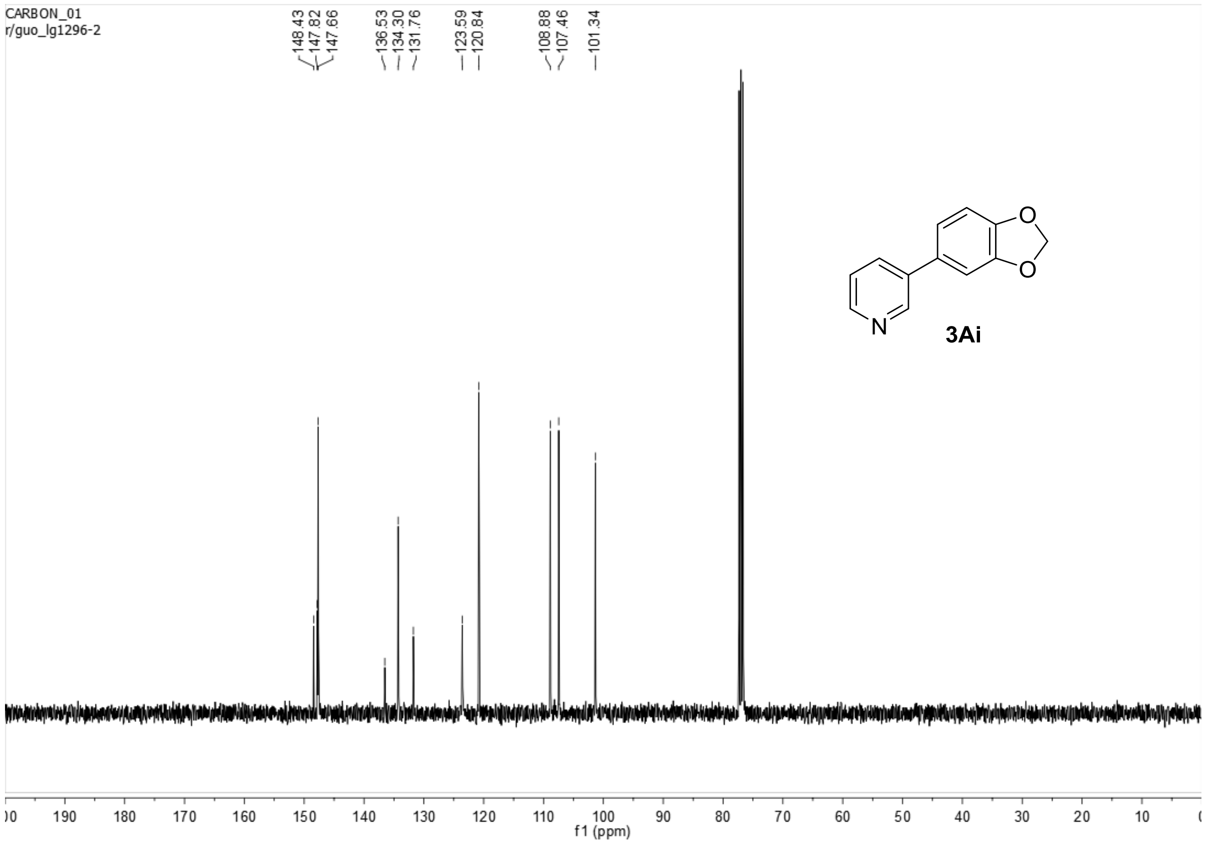

Supplementary Figure 44. ^{13}C NMR spectrum in $CDCl_3$ of compound **3Ai**.

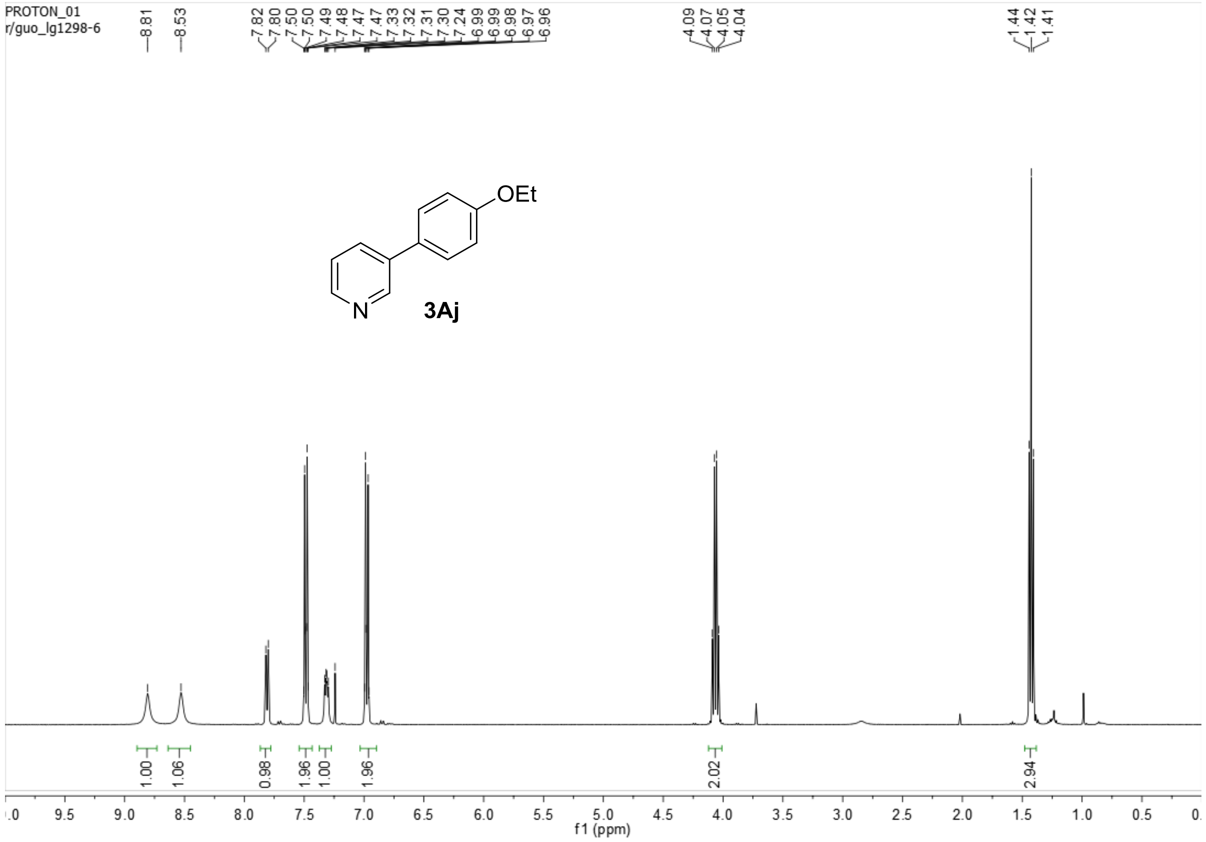

Supplementary Figure 45. ^{1}H NMR spectrum in $CDCl_3$ of compound **3Aj**.

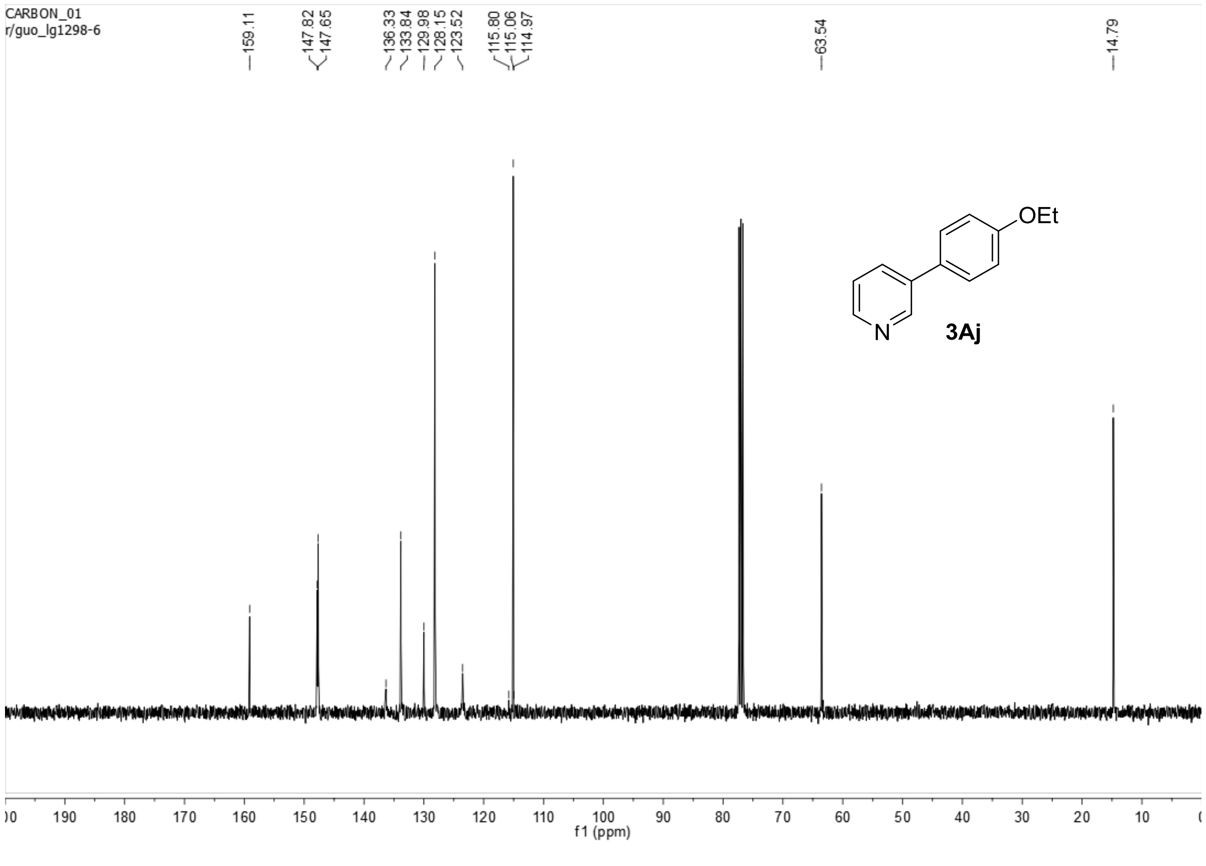

Supplementary Figure 46. ^{13}C NMR spectrum in $CDCl_3$ of compound **3Aj**.

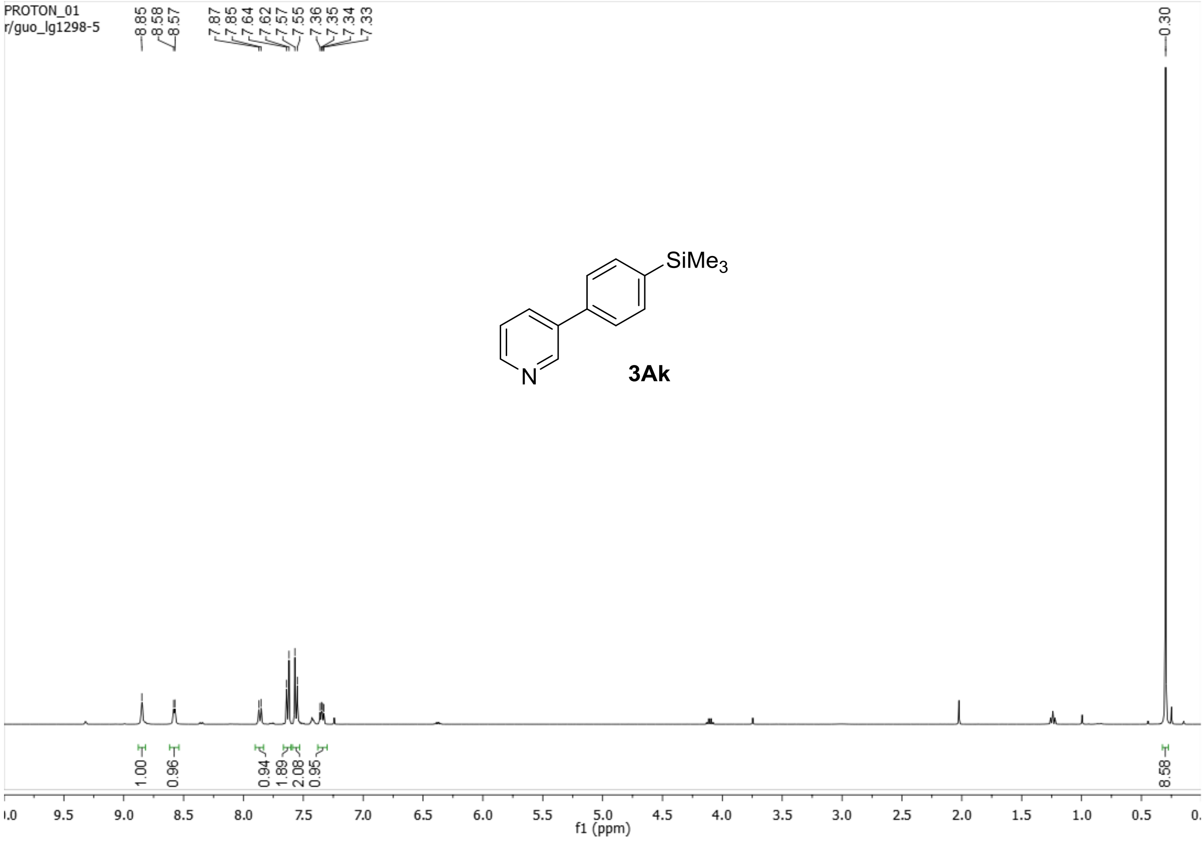

Supplementary Figure 47. ^{1}H NMR spectrum in $CDCl_3$ of compound **3Ak**.

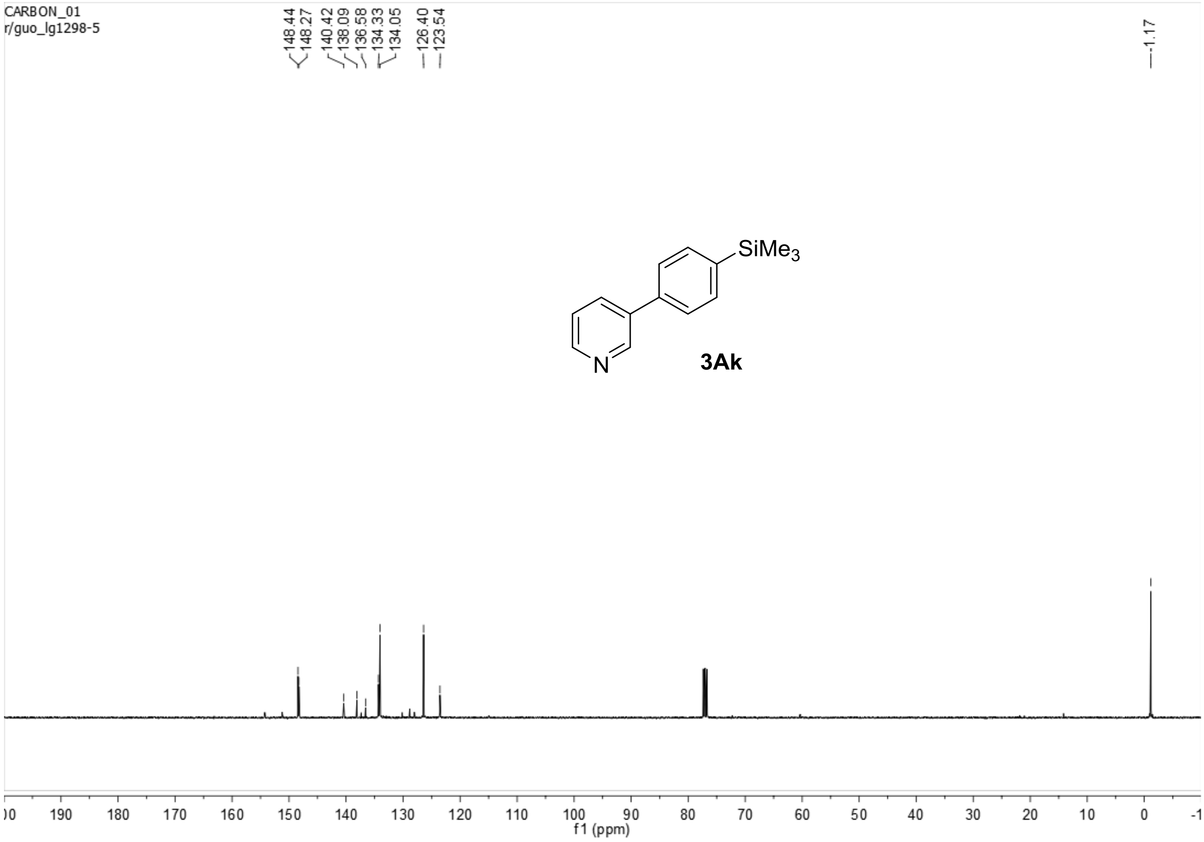

Supplementary Figure 48. ^{13}C NMR spectrum in $CDCl_3$ of compound **3Ak**.

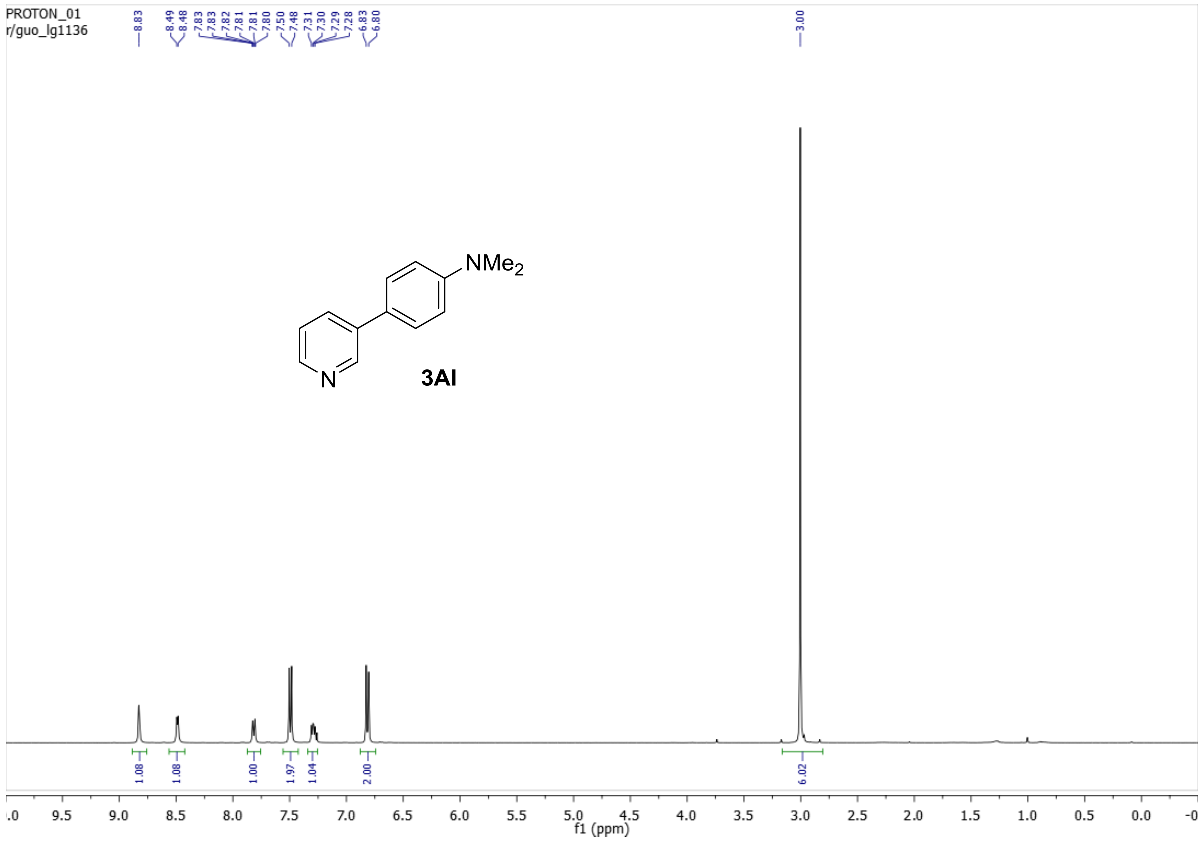

Supplementary Figure 49. ^{1}H NMR spectrum in $CDCl_3$ of compound **3Al**.

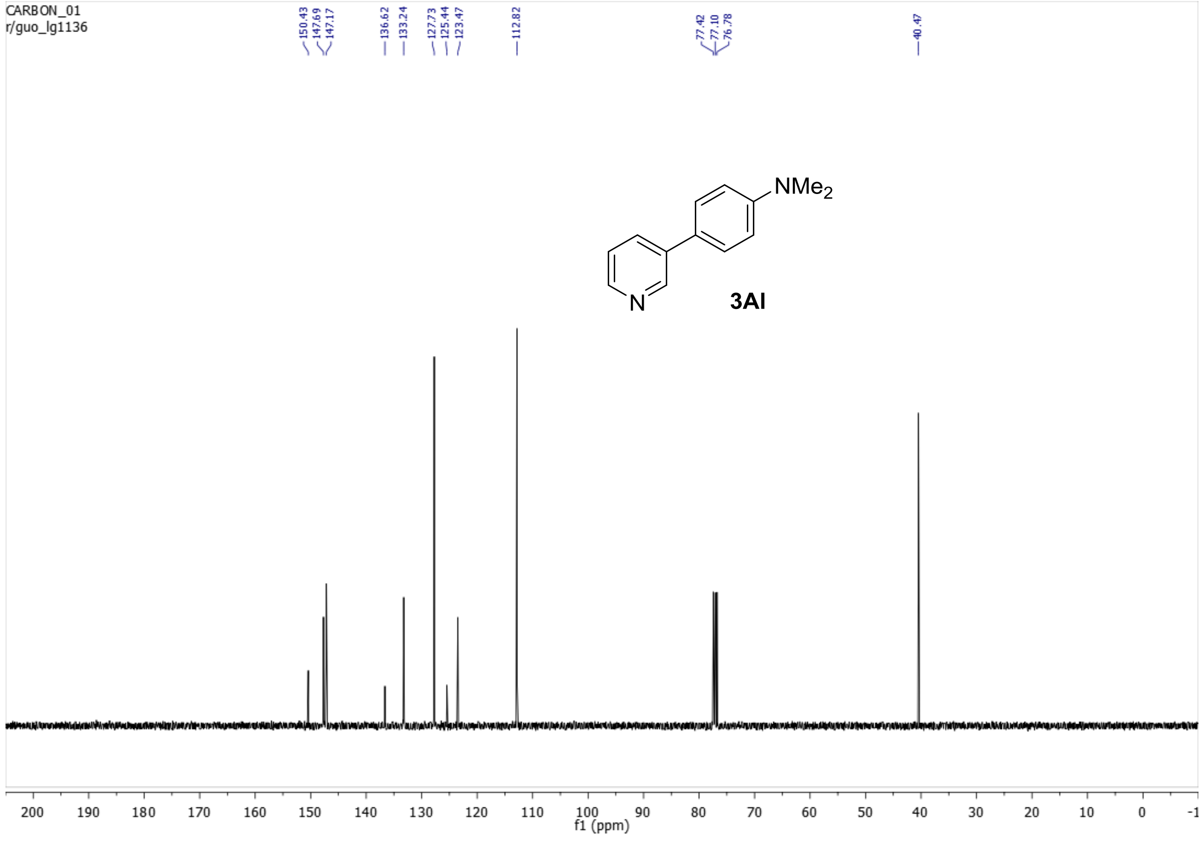

Supplementary Figure 50. ^{13}C NMR spectrum in $CDCl_3$ of compound **3Al**.

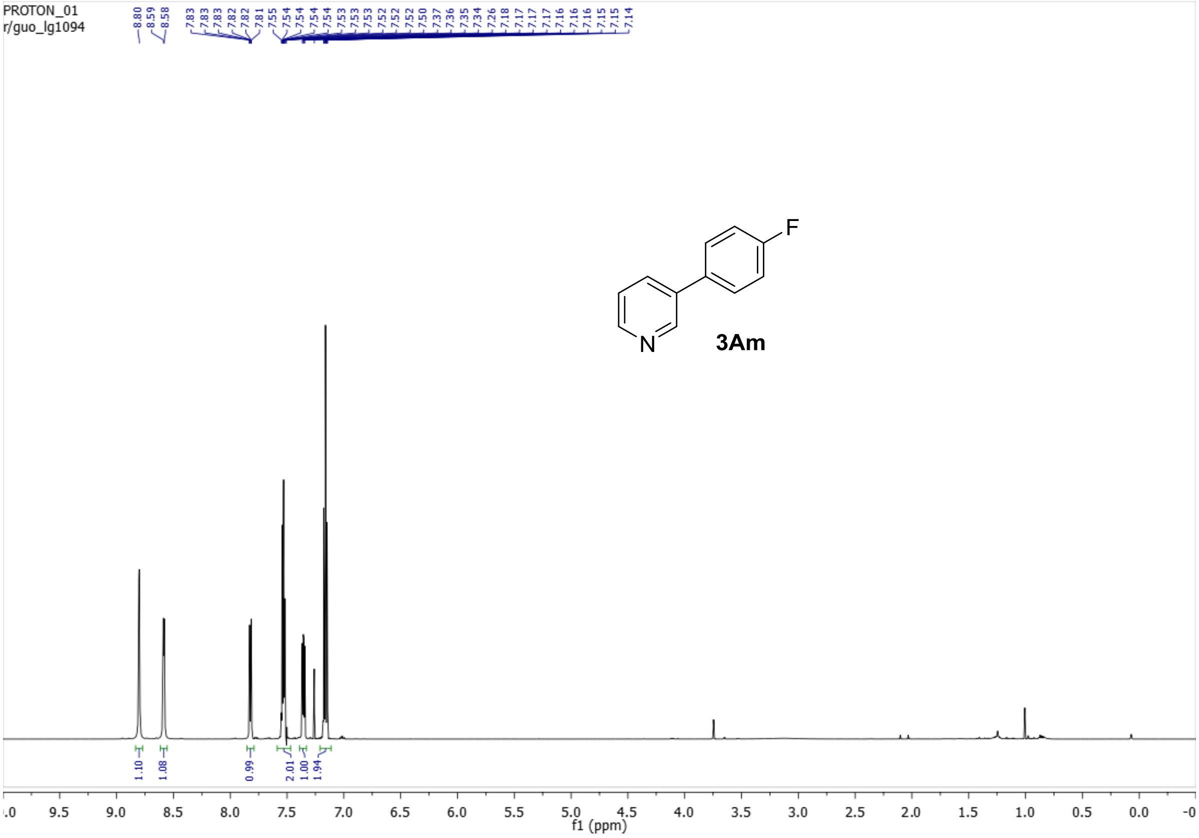

Supplementary Figure 51. ^{1}H NMR spectrum in $CDCl_3$ of compound **3Am**.

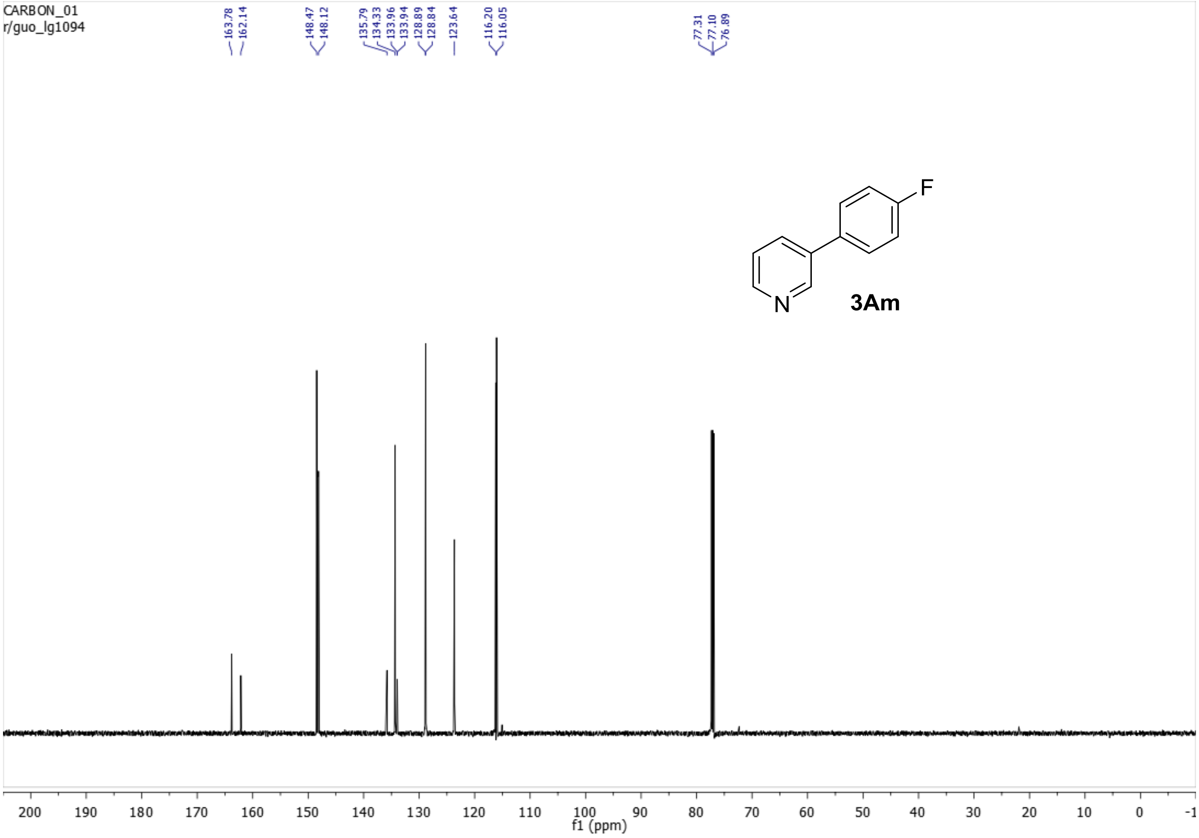

Supplementary Figure 52. ^{13}C NMR spectrum in $CDCl_3$ of compound **3Am**.

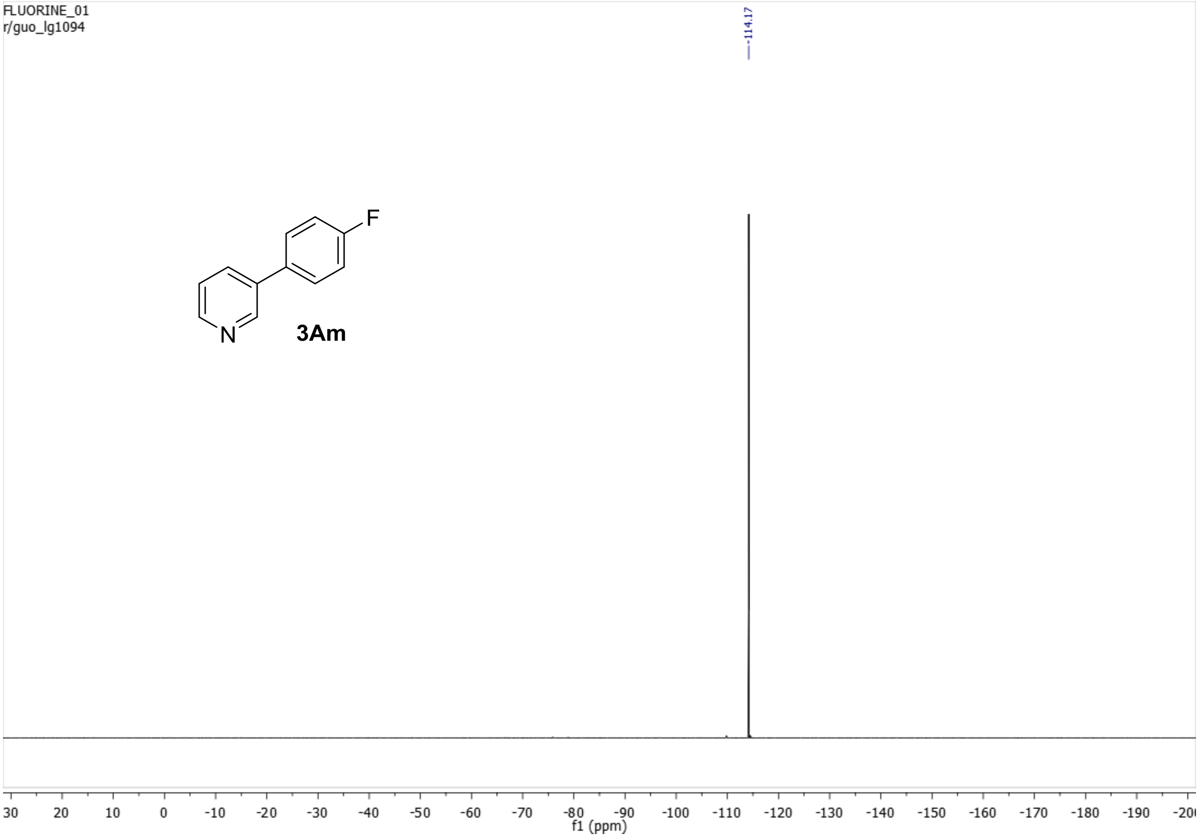

Supplementary Figure 53. ^{19}F NMR spectrum in $CDCl_3$ of compound **3Am**.

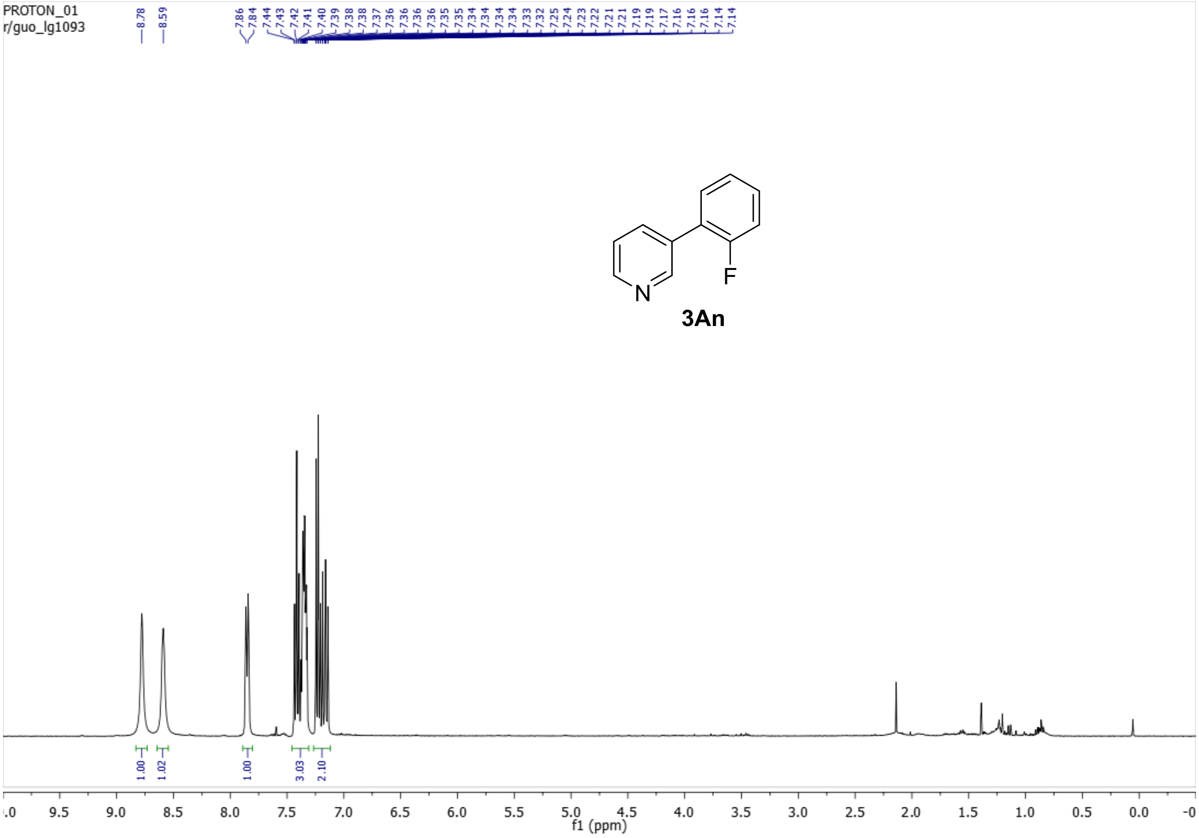

Supplementary Figure 54. ^{1}H NMR spectrum in $CDCl_3$ of compound **3An**.

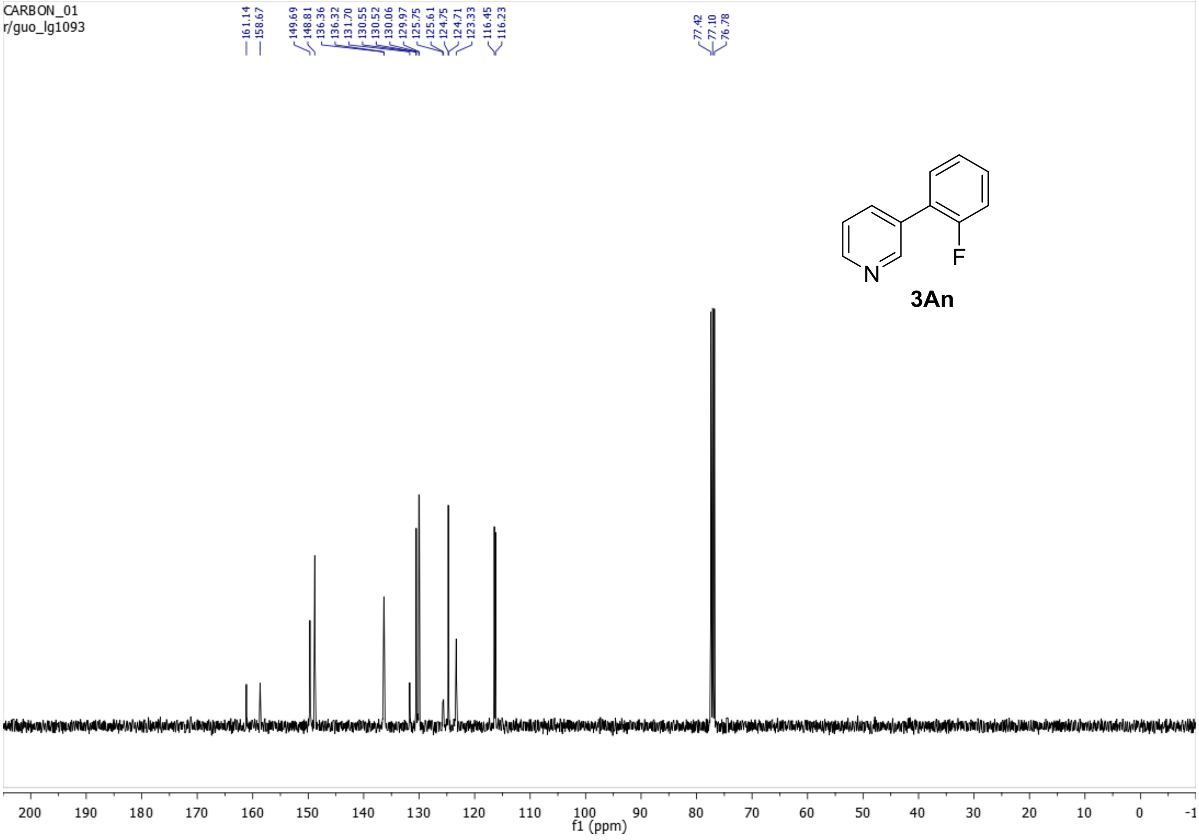

Supplementary Figure 55. ^{13}C NMR spectrum in $CDCl_3$ of compound **3An**.

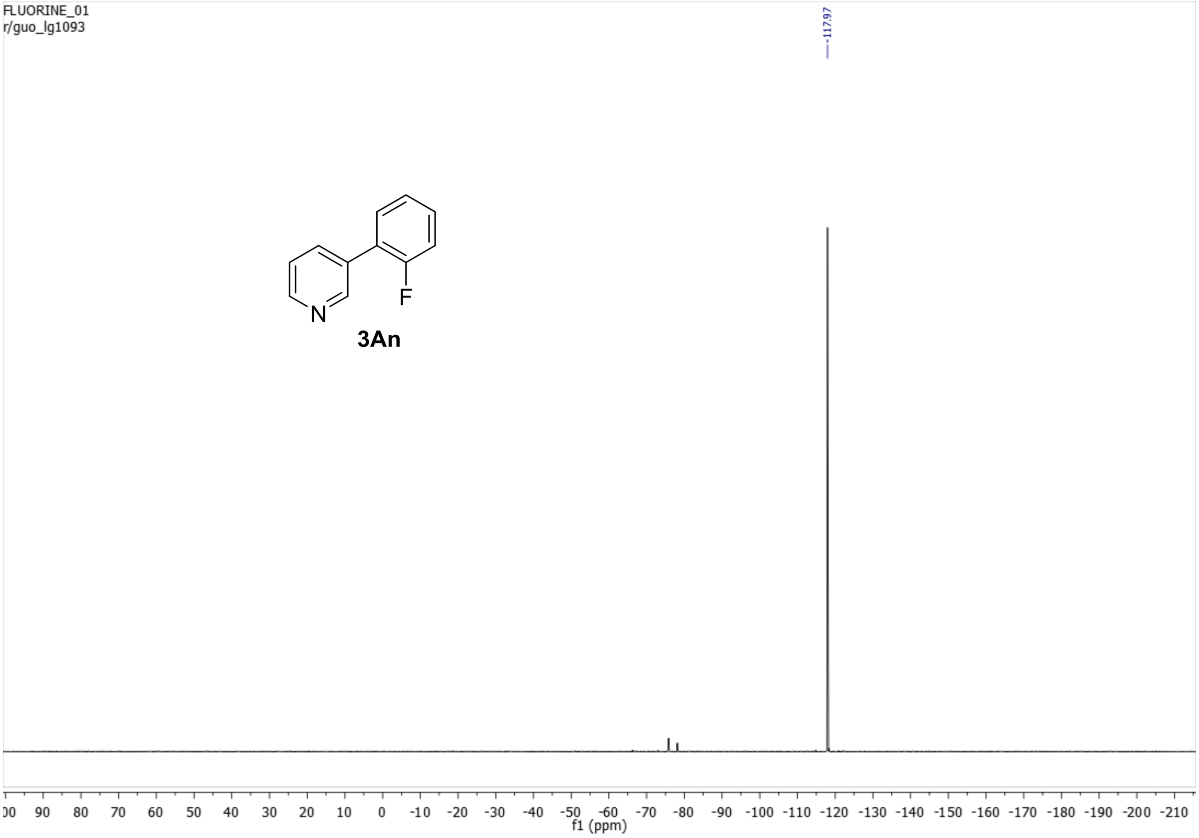

Supplementary Figure 56. ^{19}F NMR spectrum in $CDCl_3$ of compound **3An**.

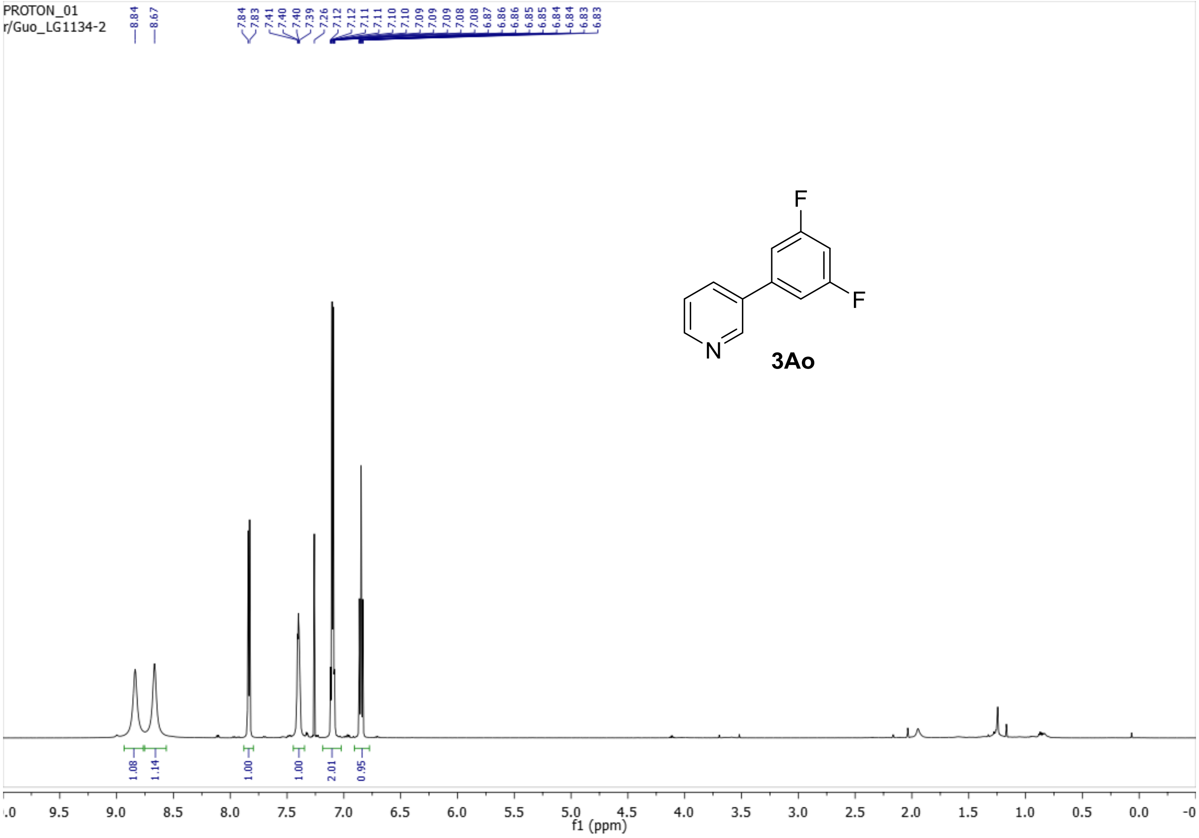

Supplementary Figure 57. ^{1}H NMR spectrum in $CDCl_3$ of compound **3Ao**.

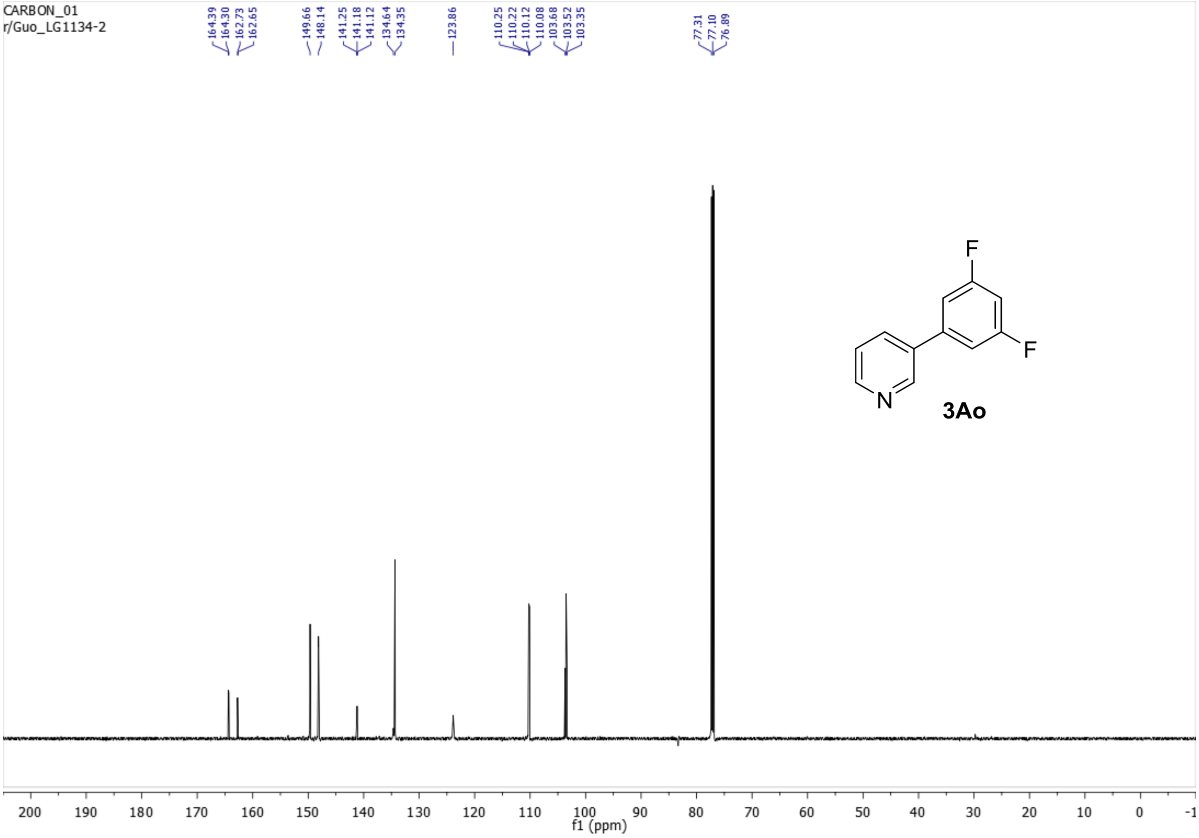

Supplementary Figure 58. ^{13}C NMR spectrum in $CDCl_3$ of compound **3Ao**.

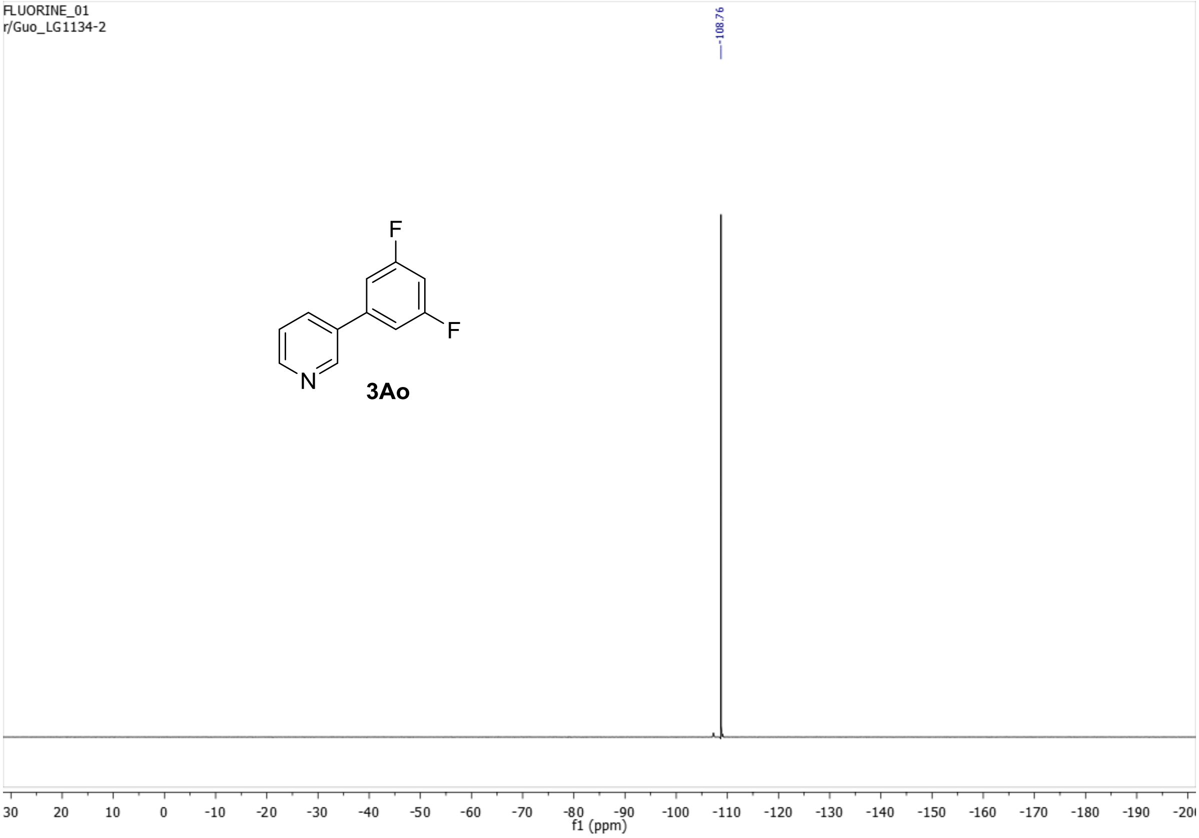

Supplementary Figure 59. ^{19}F NMR spectrum in $CDCl_3$ of compound **3Ao**.

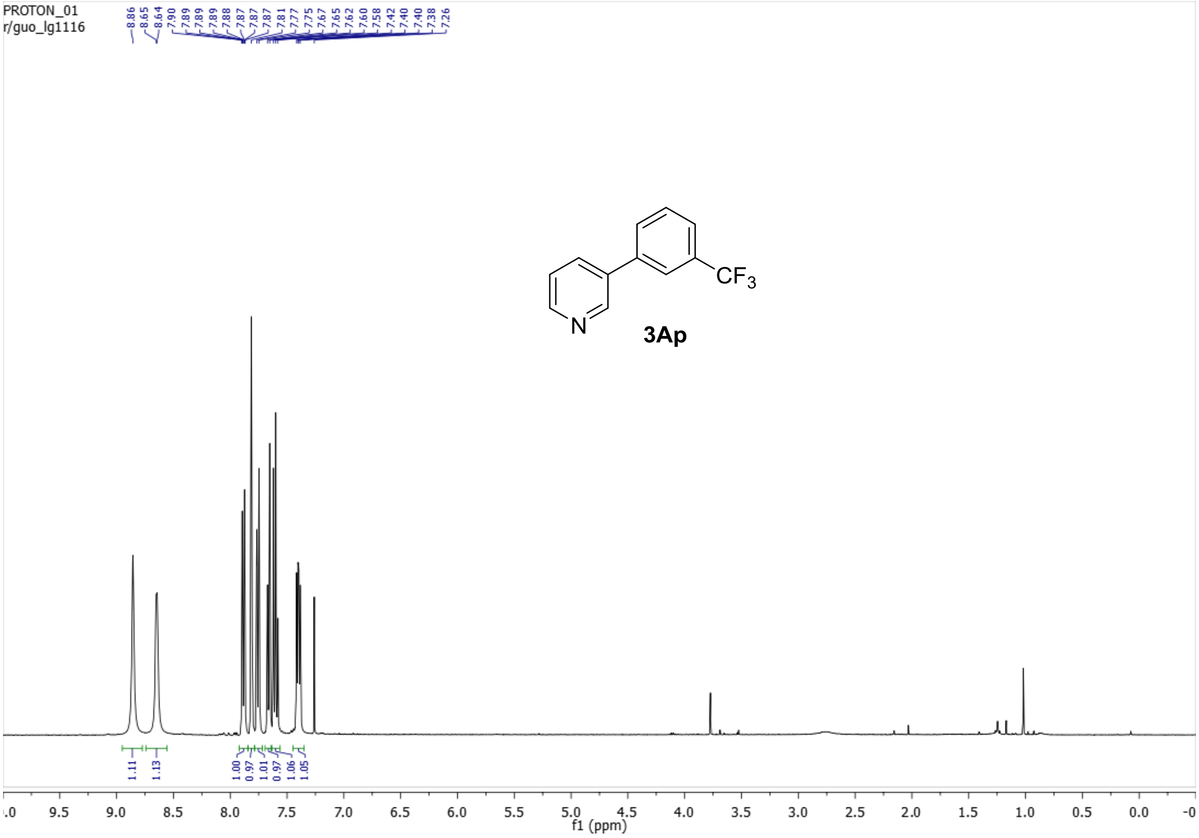

Supplementary Figure 60. ^{1}H NMR spectrum in $CDCl_3$ of compound **3Ap**.

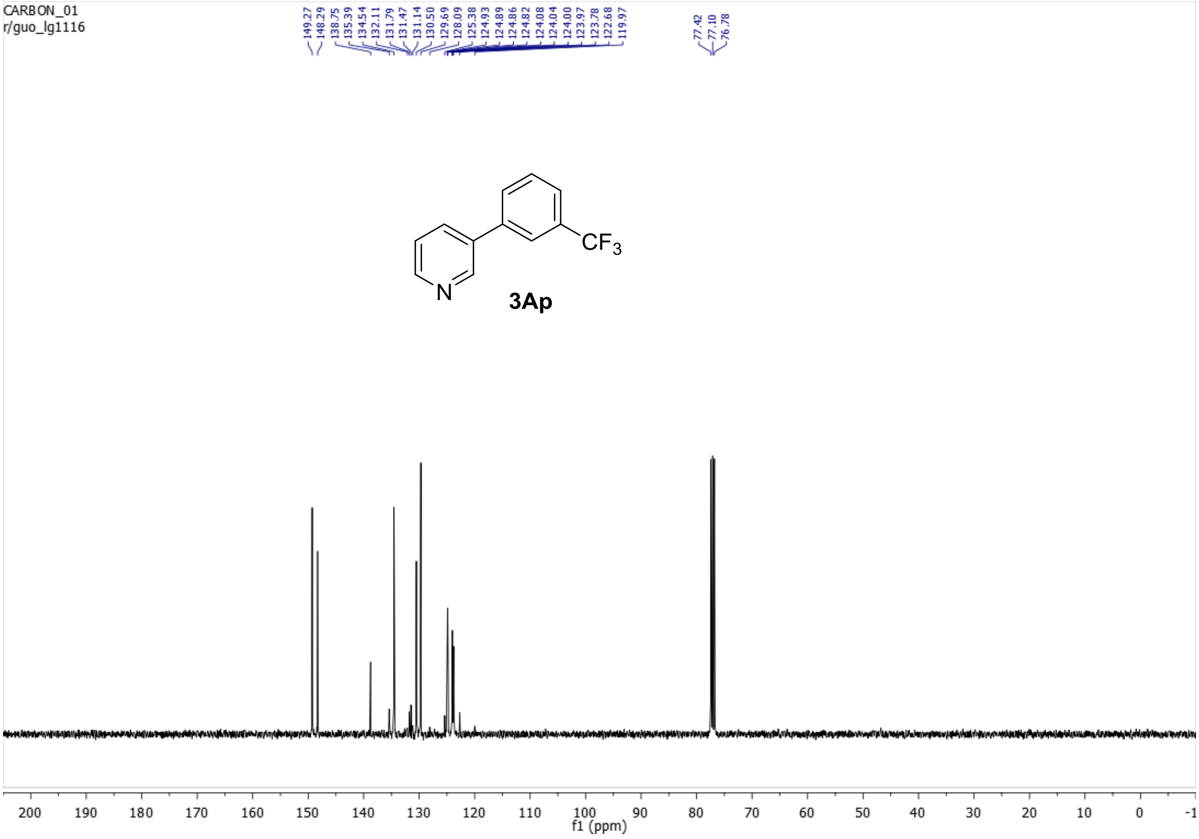

Supplementary Figure 61. ^{13}C NMR spectrum in $CDCl_3$ of compound **3Ap**.

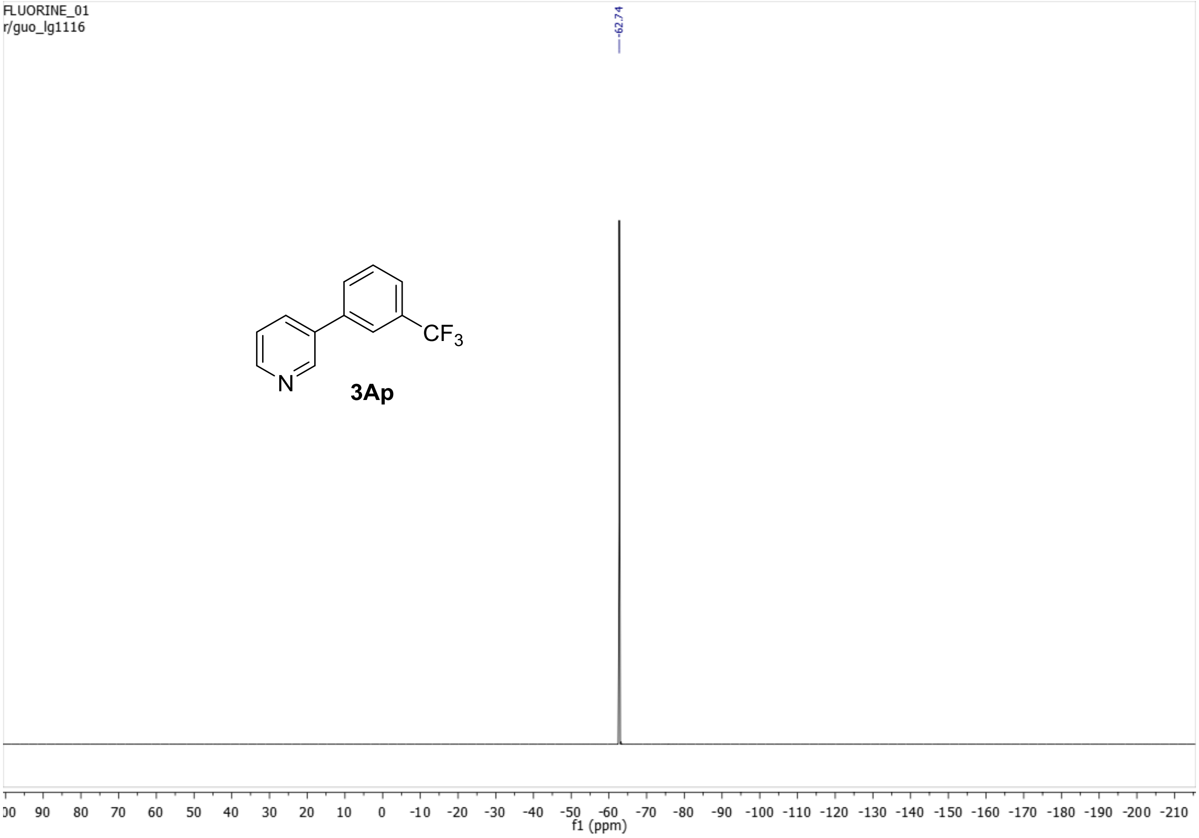

Supplementary Figure 62. ^{19}F NMR spectrum in $CDCl_3$ of compound **3Ap**.

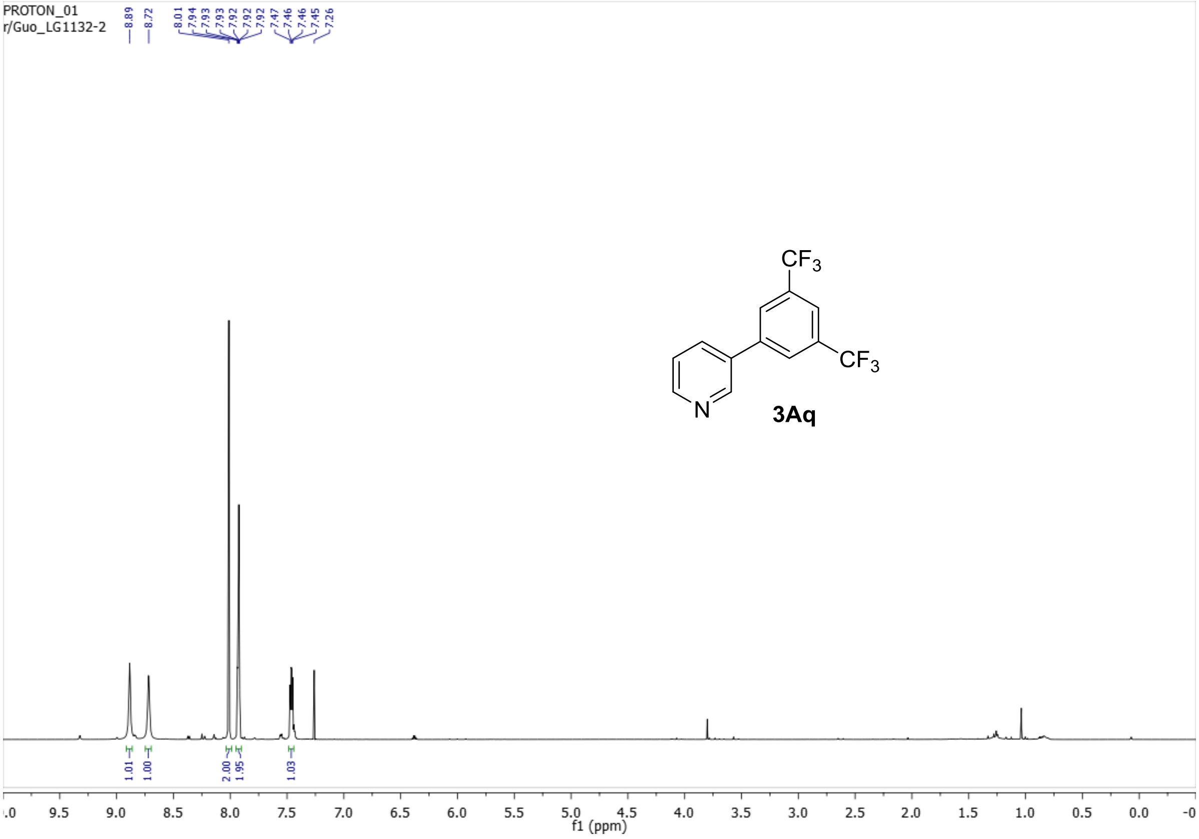

Supplementary Figure 63 ^{1}H NMR spectrum in $CDCl_3$ of compound **3Aq**.

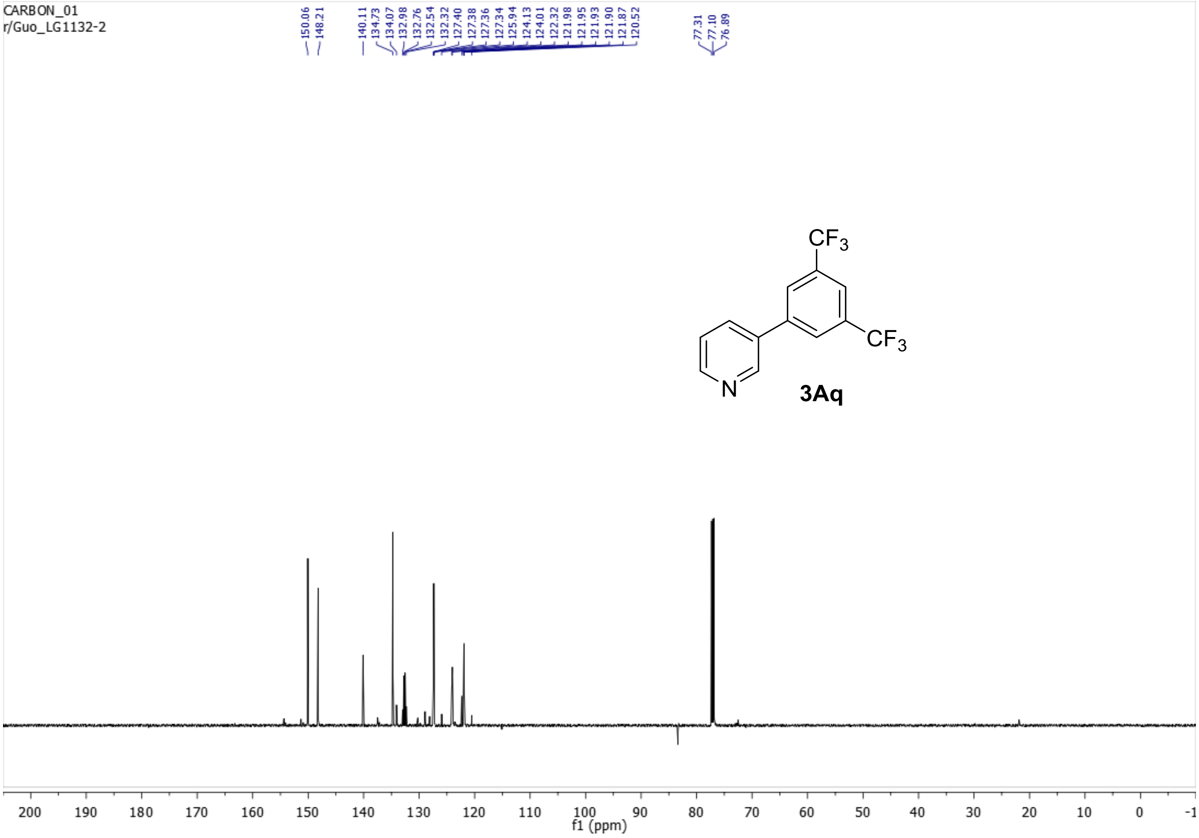

Supplementary Figure 64. ^{13}C NMR spectrum in $CDCl_3$ of compound **3Aq**.

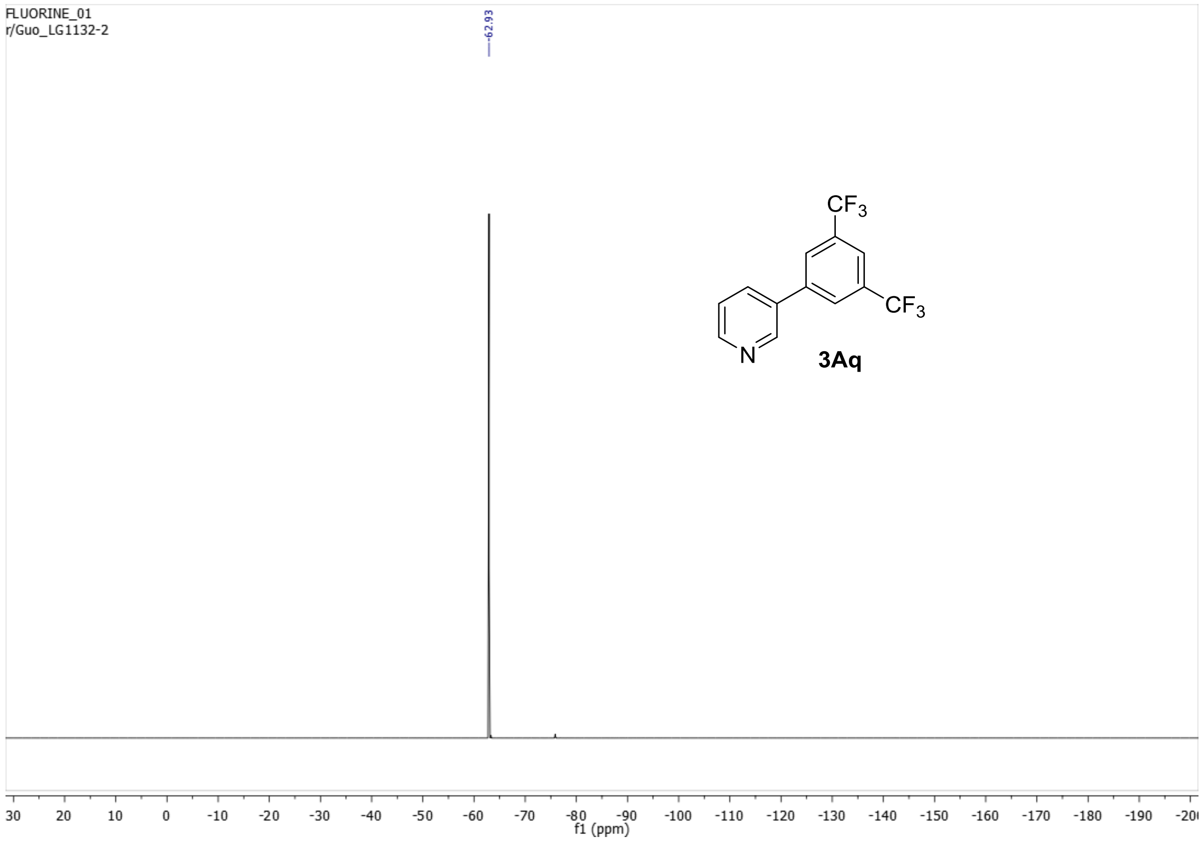

Supplementary Figure 65. ^{19}F NMR spectrum in $CDCl_3$ of compound **3Aq**.

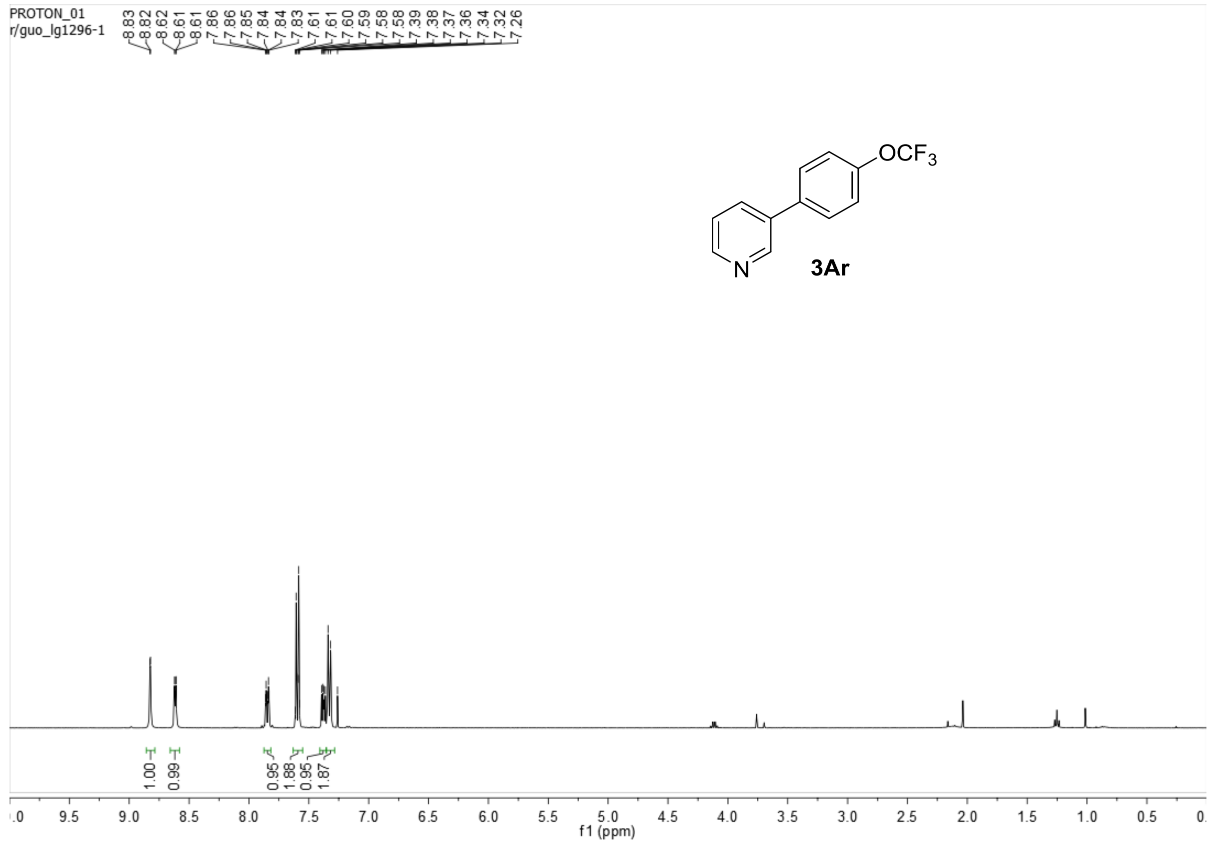

Supplementary Figure 66. ^{1}H NMR spectrum in $CDCl_3$ of compound **3Ar**.

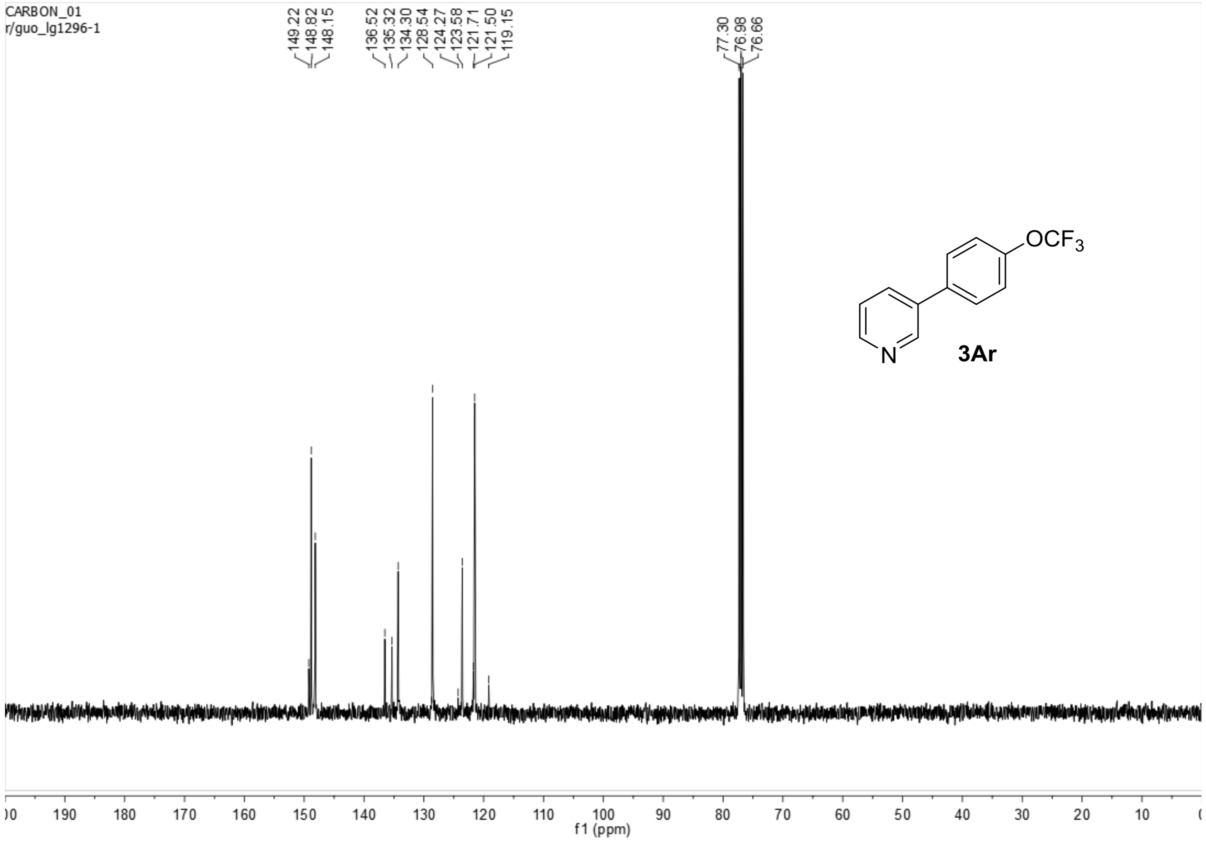

Supplementary Figure 67. ^{13}C NMR spectrum in $CDCl_3$ of compound **3Ar**.

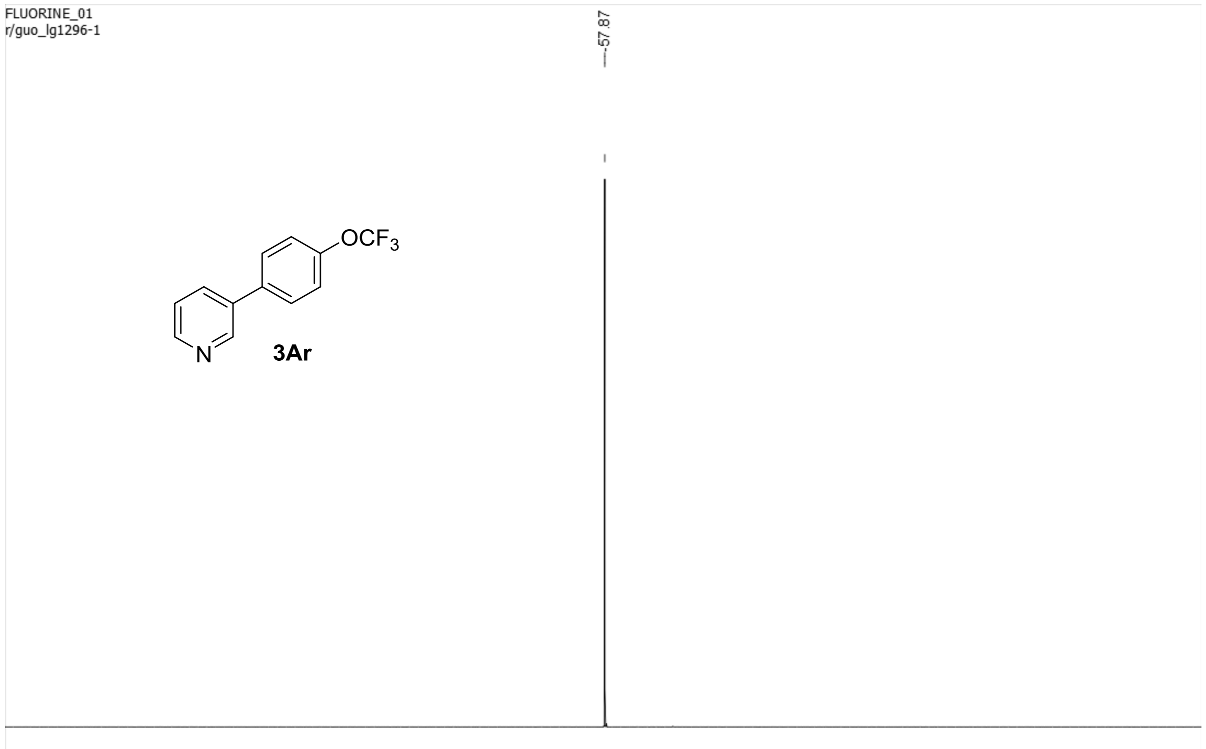

Supplementary Figure 68. ^{19}F NMR spectrum in $CDCl_3$ of compound **3Ar**.

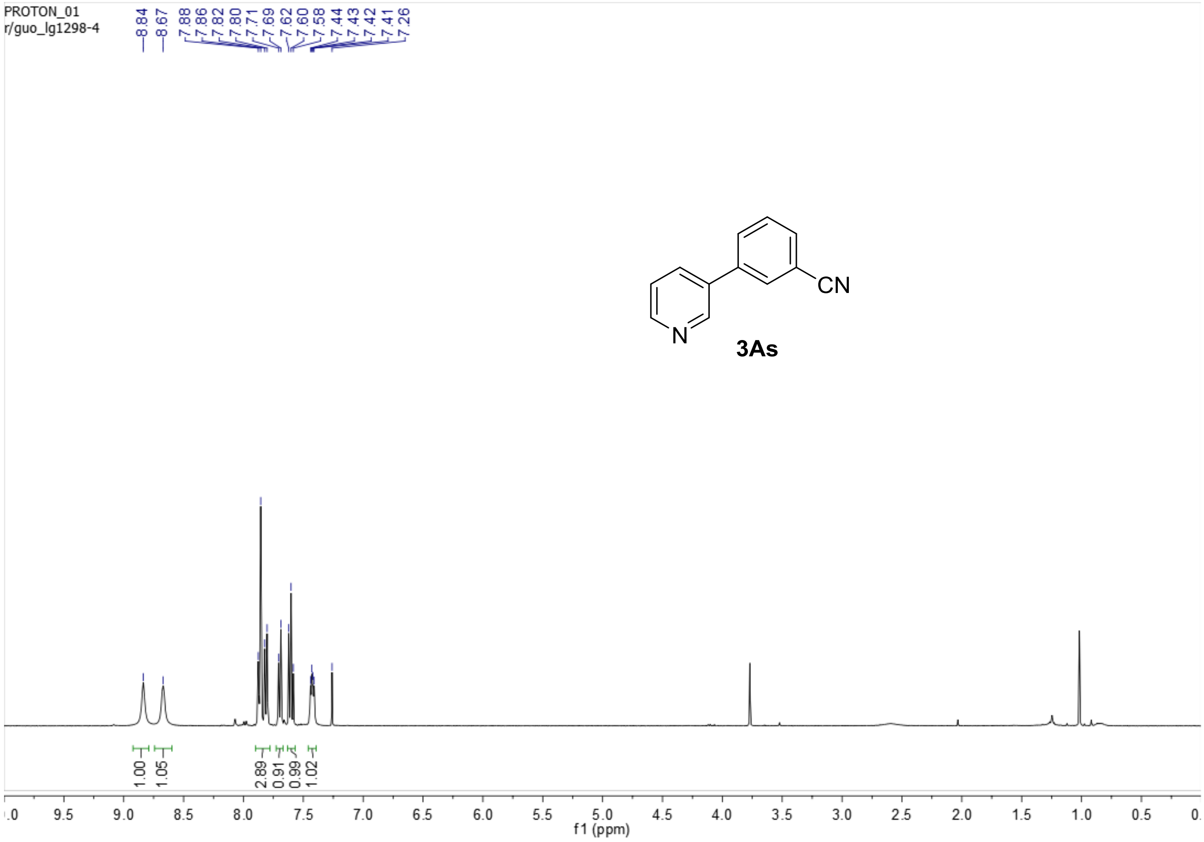

Supplementary Figure 69. ^{1}H NMR spectrum in $CDCl_3$ of compound **3As**.

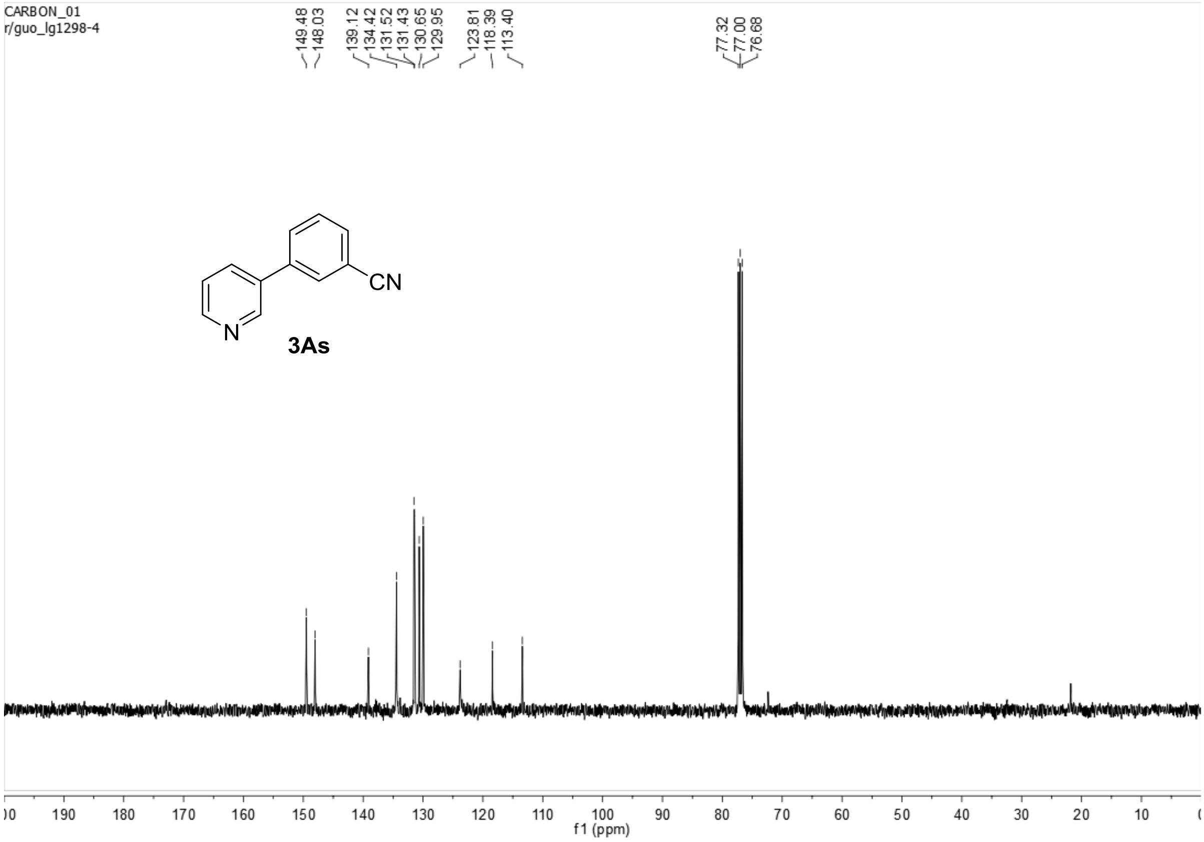

Supplementary Figure 70. ^{13}C NMR spectrum in $CDCl_3$ of compound **3As**.

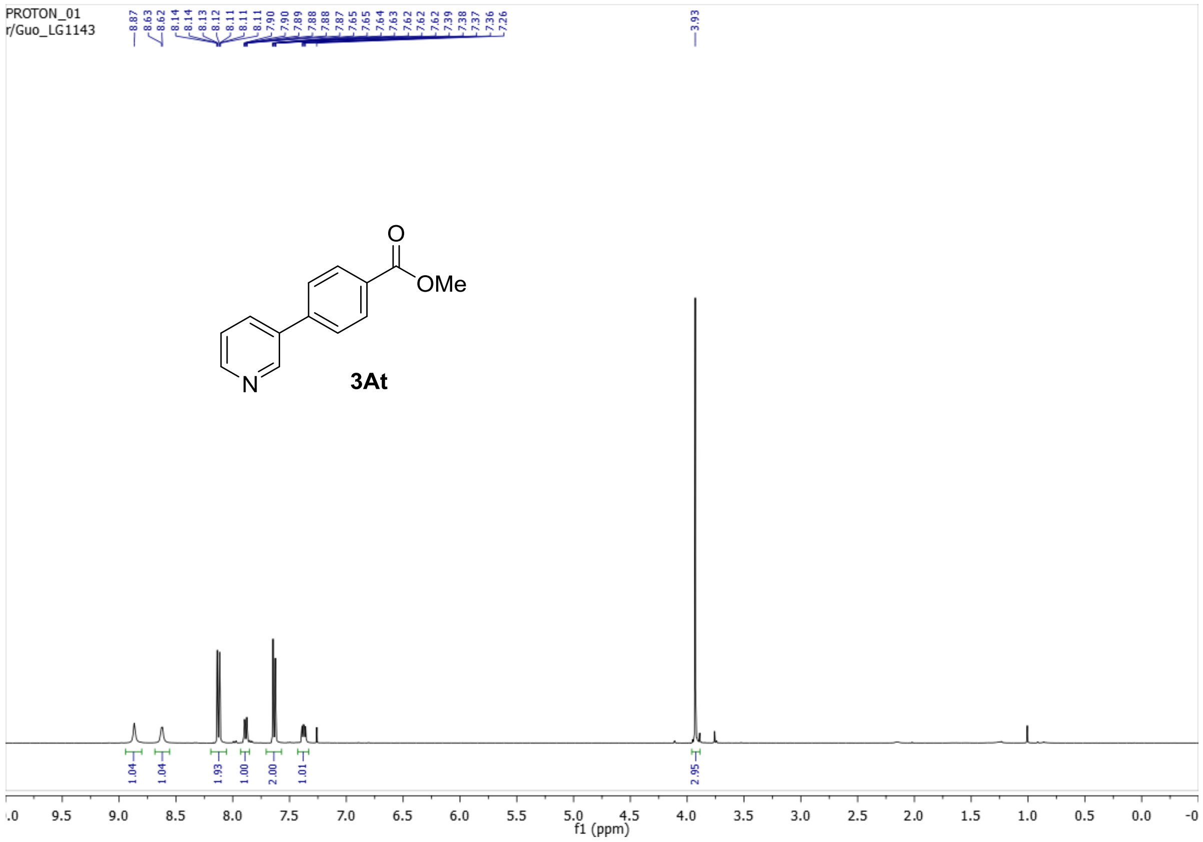

Supplementary Figure 71. ^{1}H NMR spectrum in $CDCl_3$ of compound **3At**.

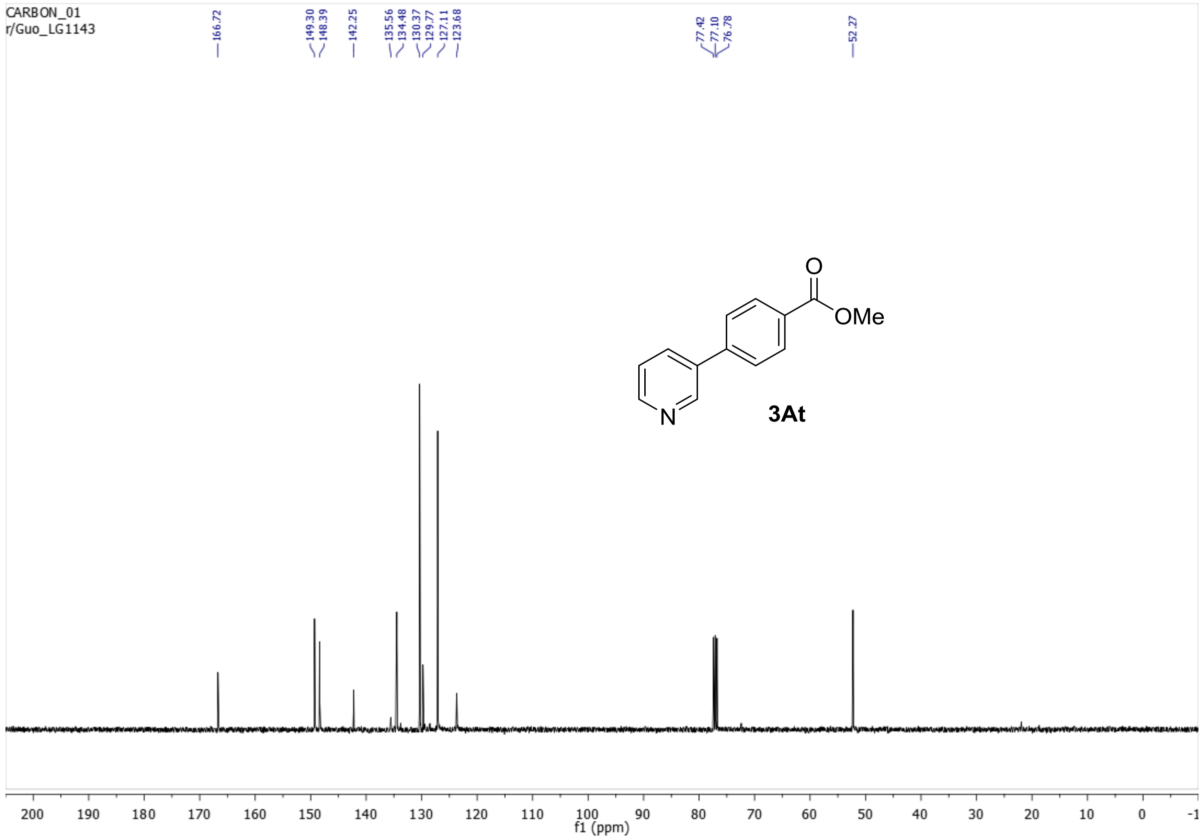

Supplementary Figure 72. ^{13}C NMR spectrum in $CDCl_3$ of compound **3At**.

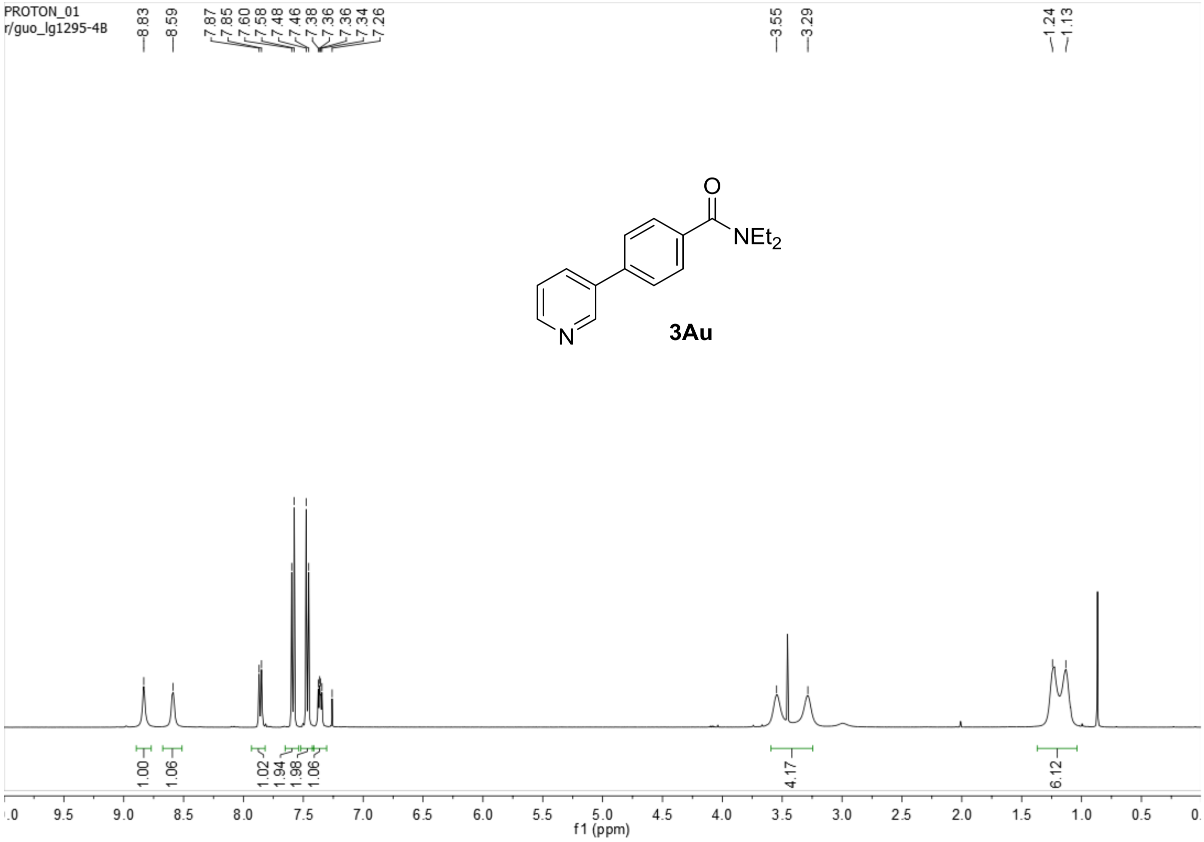

Supplementary Figure 73. ^{1}H NMR spectrum in $CDCl_3$ of compound **3Au**.

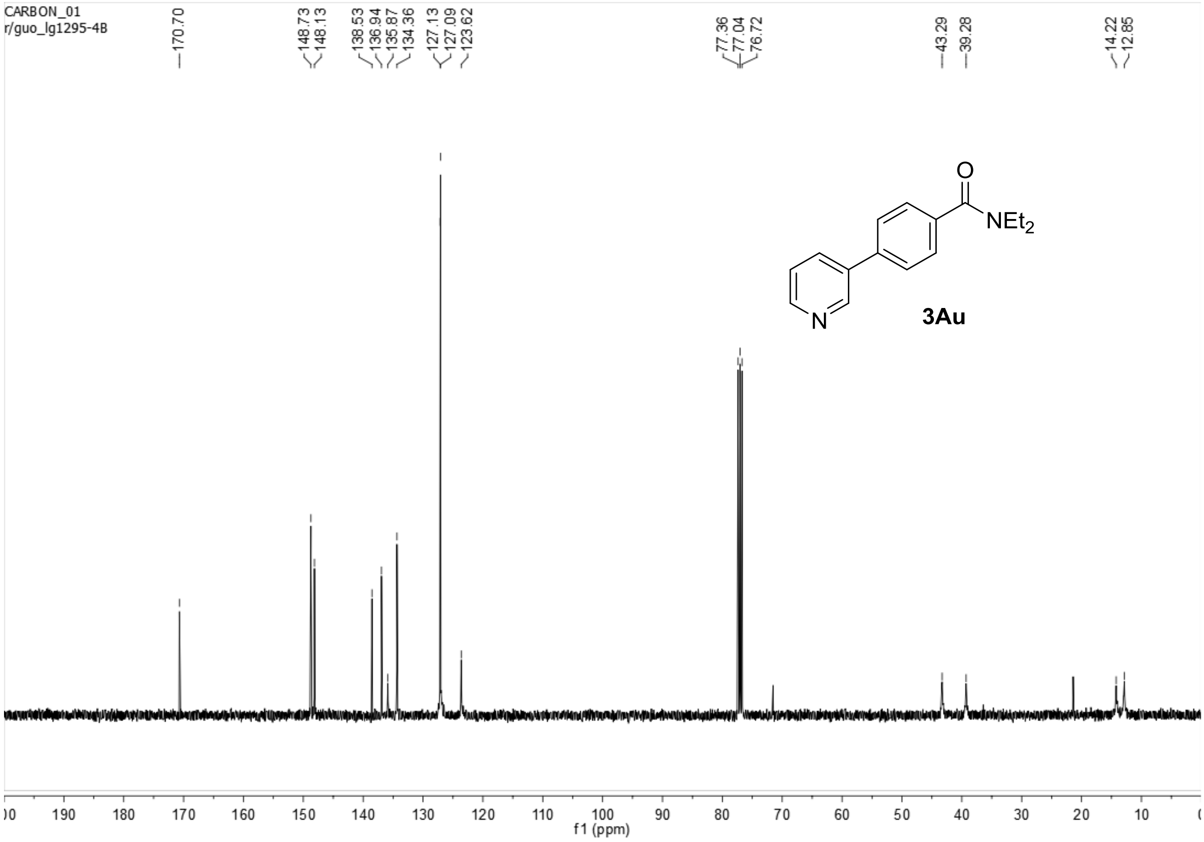

Supplementary Figure 74. ^{13}C NMR spectrum in $CDCl_3$ of compound **3Au**.

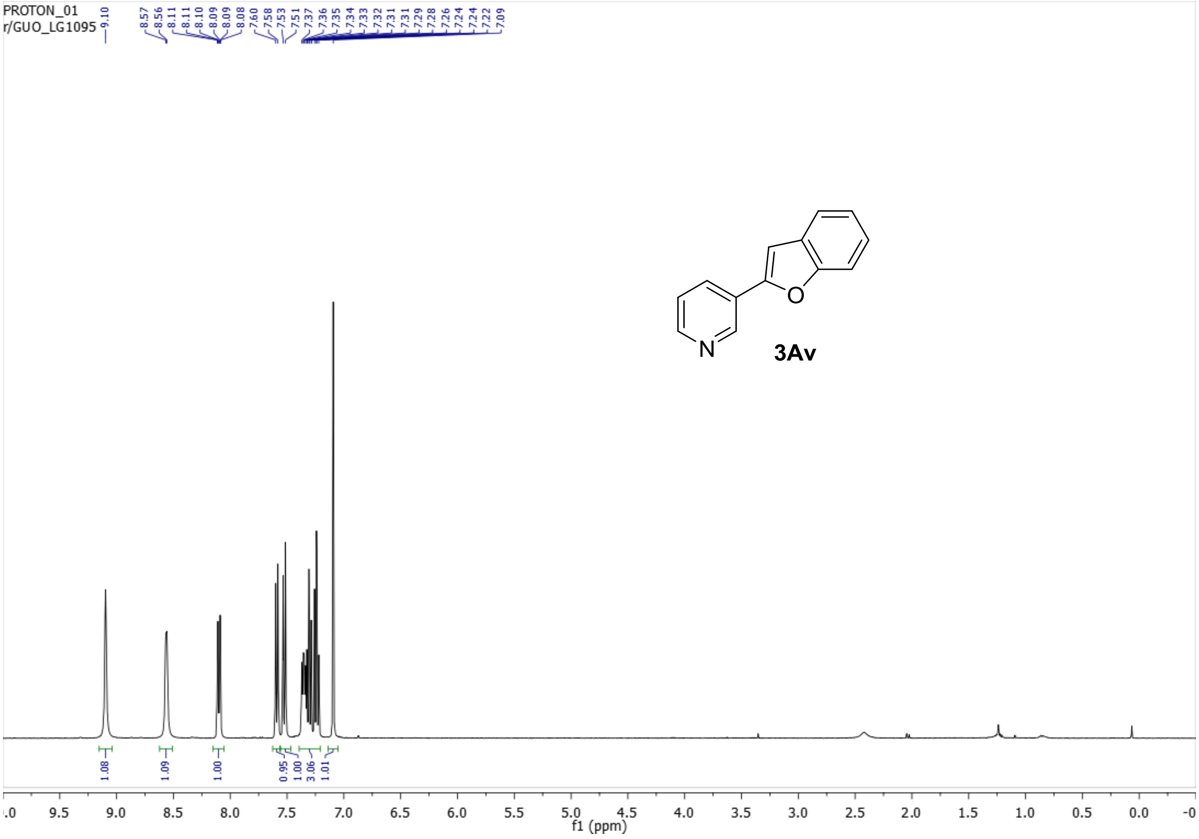

Supplementary Figure 75. ^{1}H NMR spectrum in $CDCl_3$ of compound **3Av**.

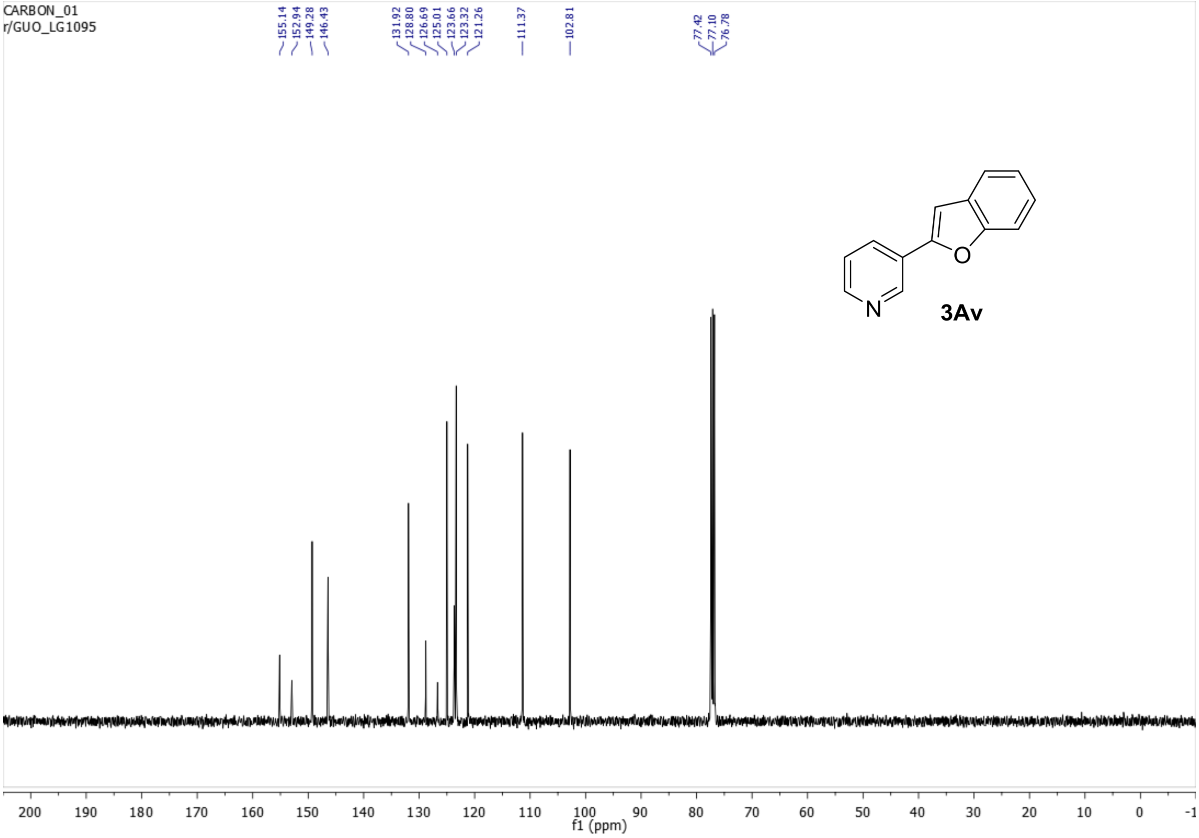

Supplementary Figure 76. ^{13}C NMR spectrum in $CDCl_3$ of compound **3Av**.

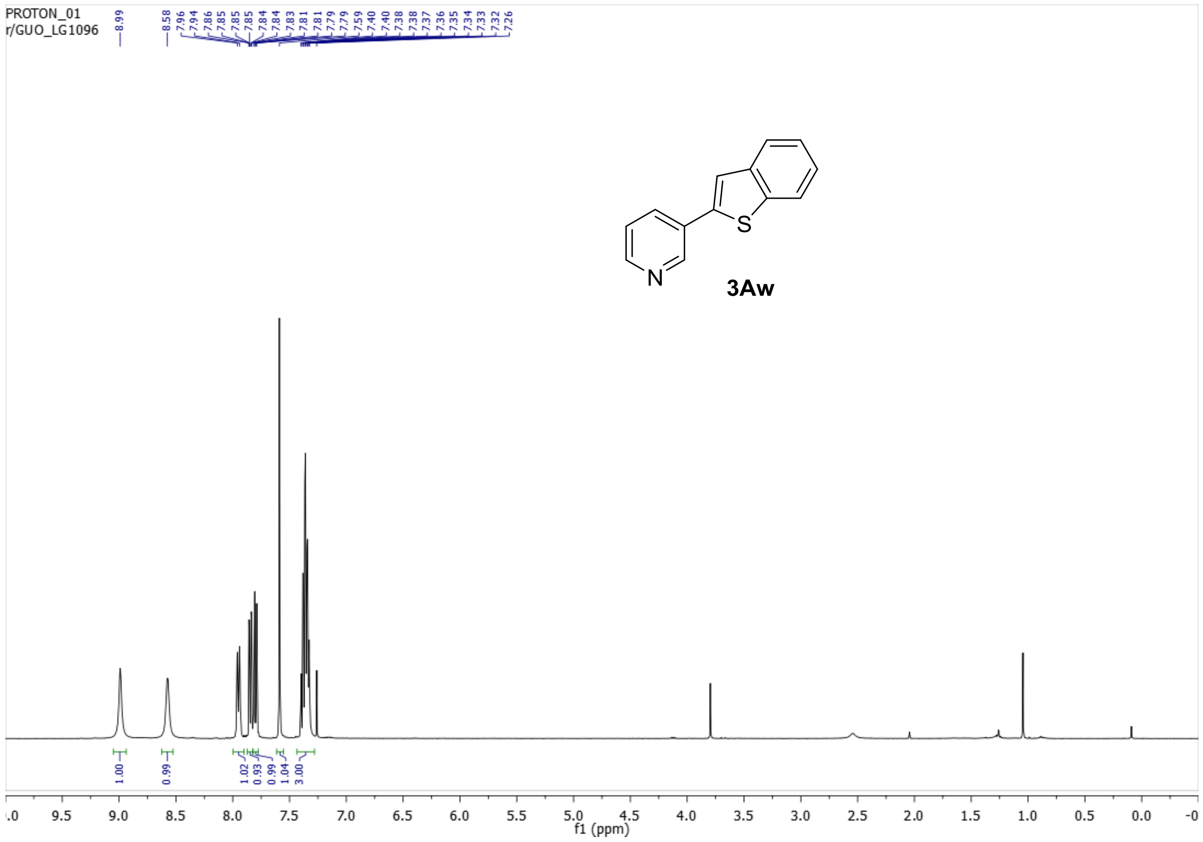

Supplementary Figure 77. ^{1}H NMR spectrum in $CDCl_3$ of compound **3Aw**.

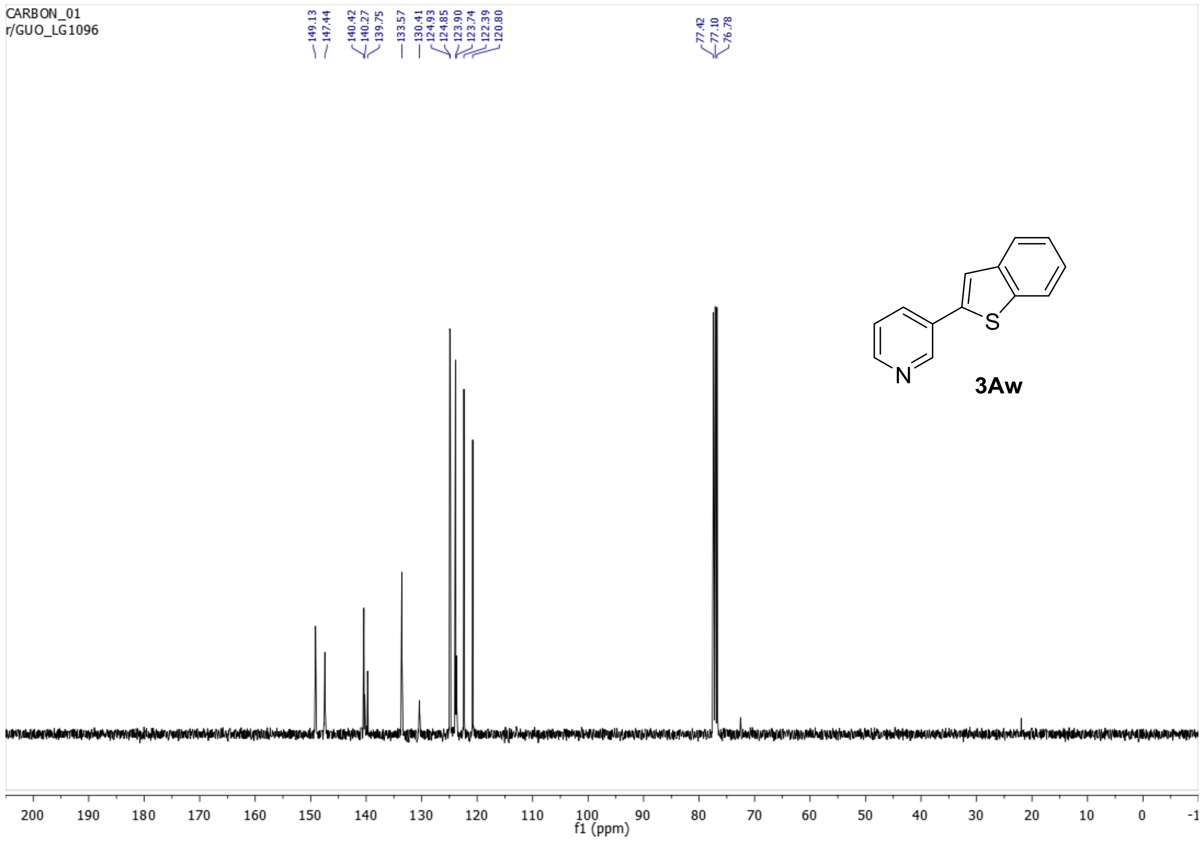

Supplementary Figure 78. ^{13}C NMR spectrum in $CDCl_3$ of compound **3Aw**.

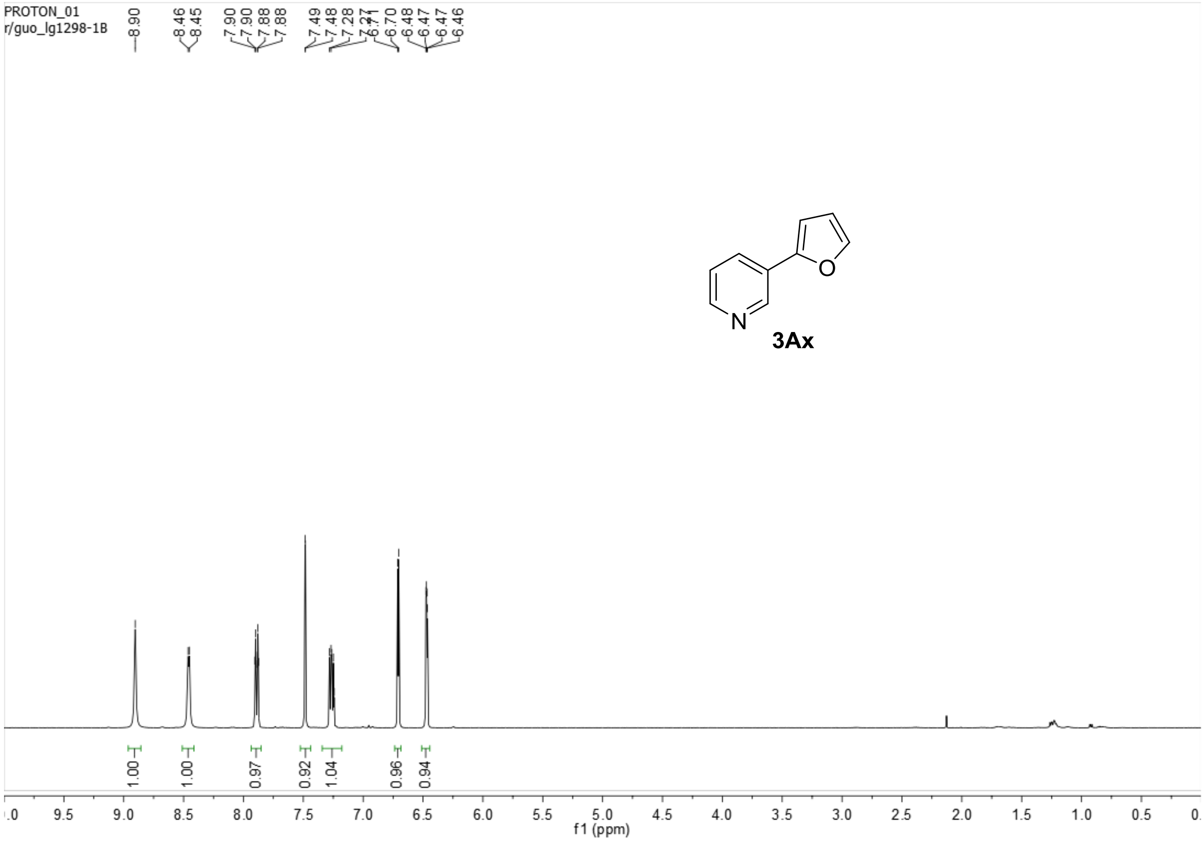

Supplementary Figure 79. ^{1}H NMR spectrum in $CDCl_3$ of compound **3Ax**.

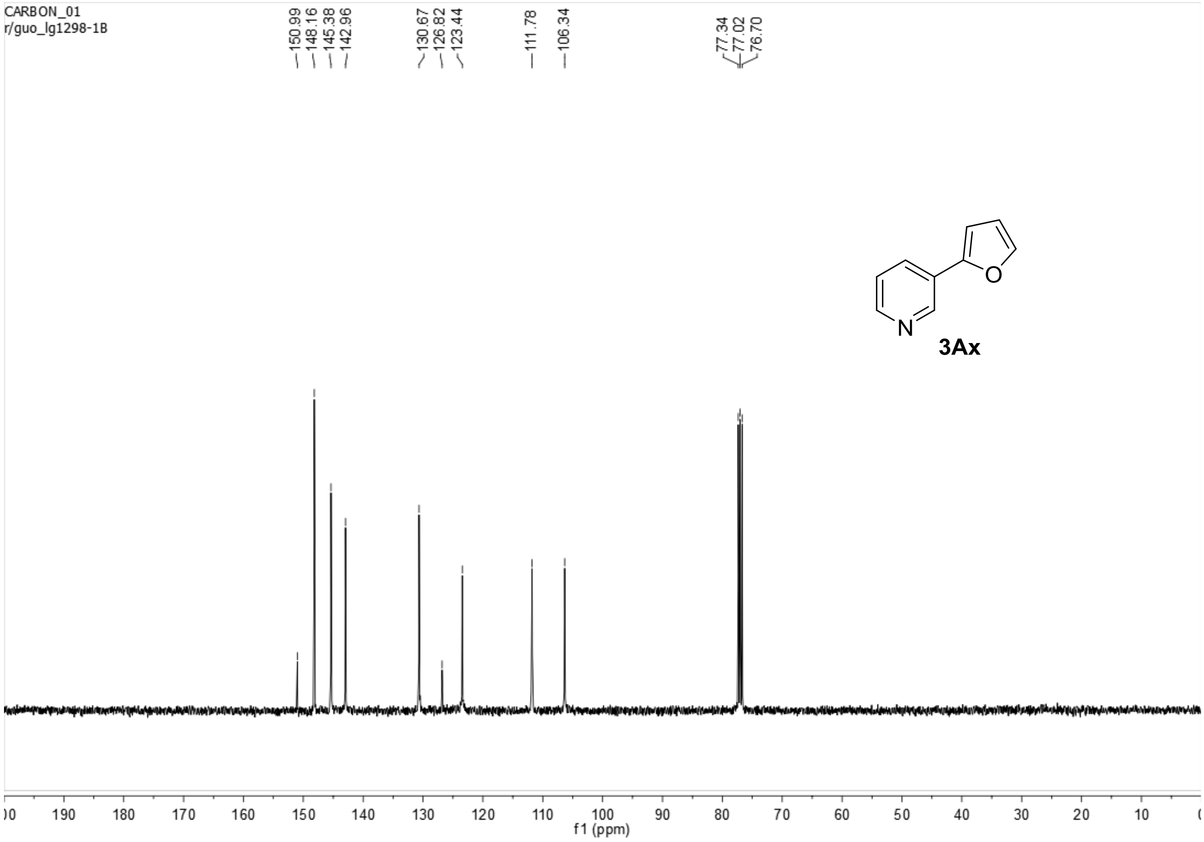

Supplementary Figure 80. ^{13}C NMR spectrum in $CDCl_3$ of compound **3Ax**.

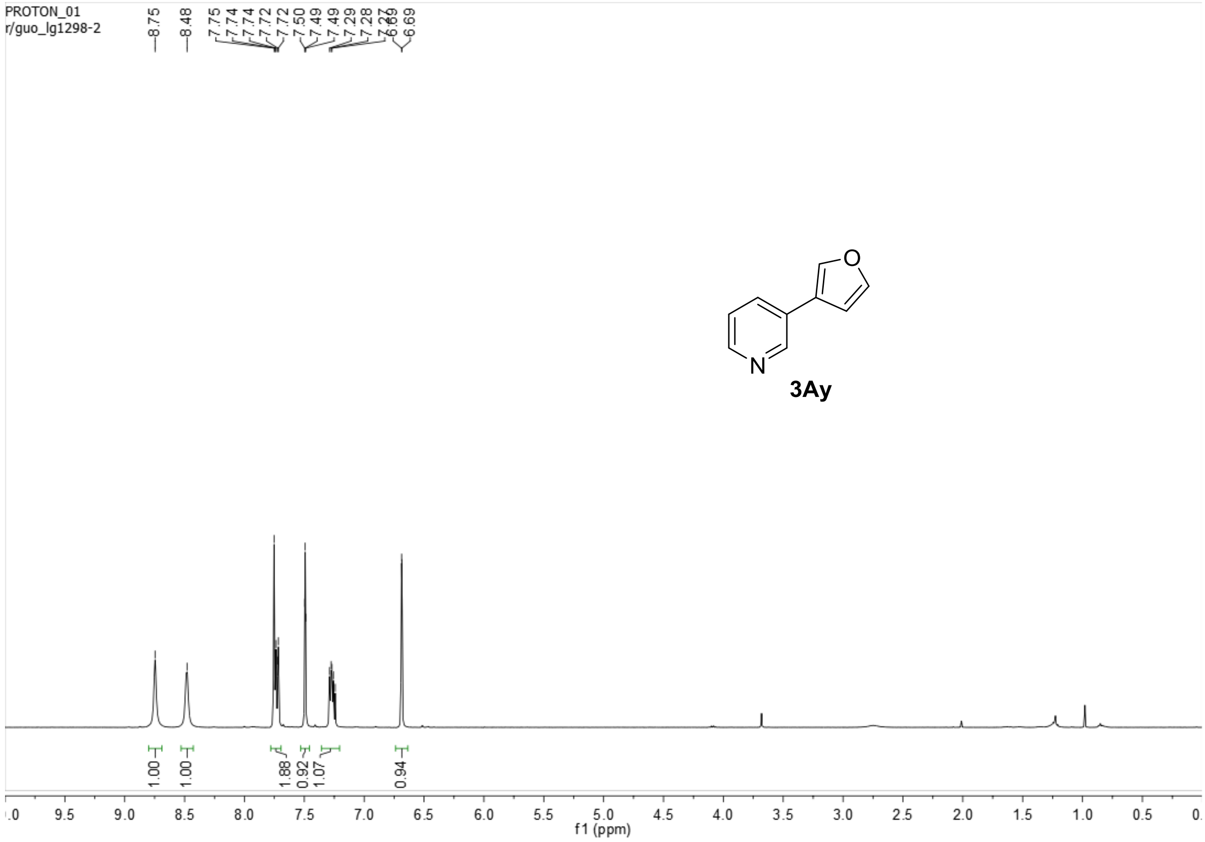

Supplementary Figure 81. ^{1}H NMR spectrum in $CDCl_3$ of compound **3Ay**.

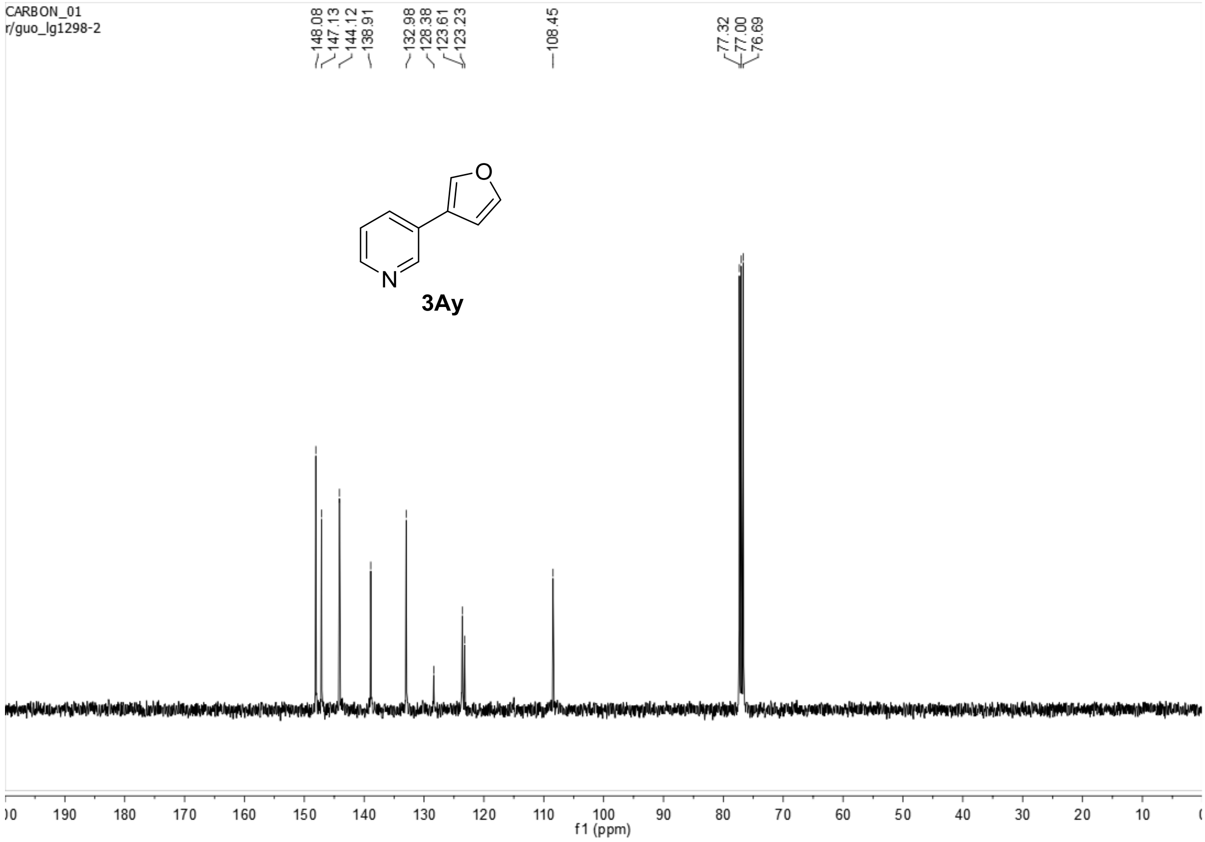

Supplementary Figure 82. ^{13}C NMR spectrum in $CDCl_3$ of compound **3Ay**.

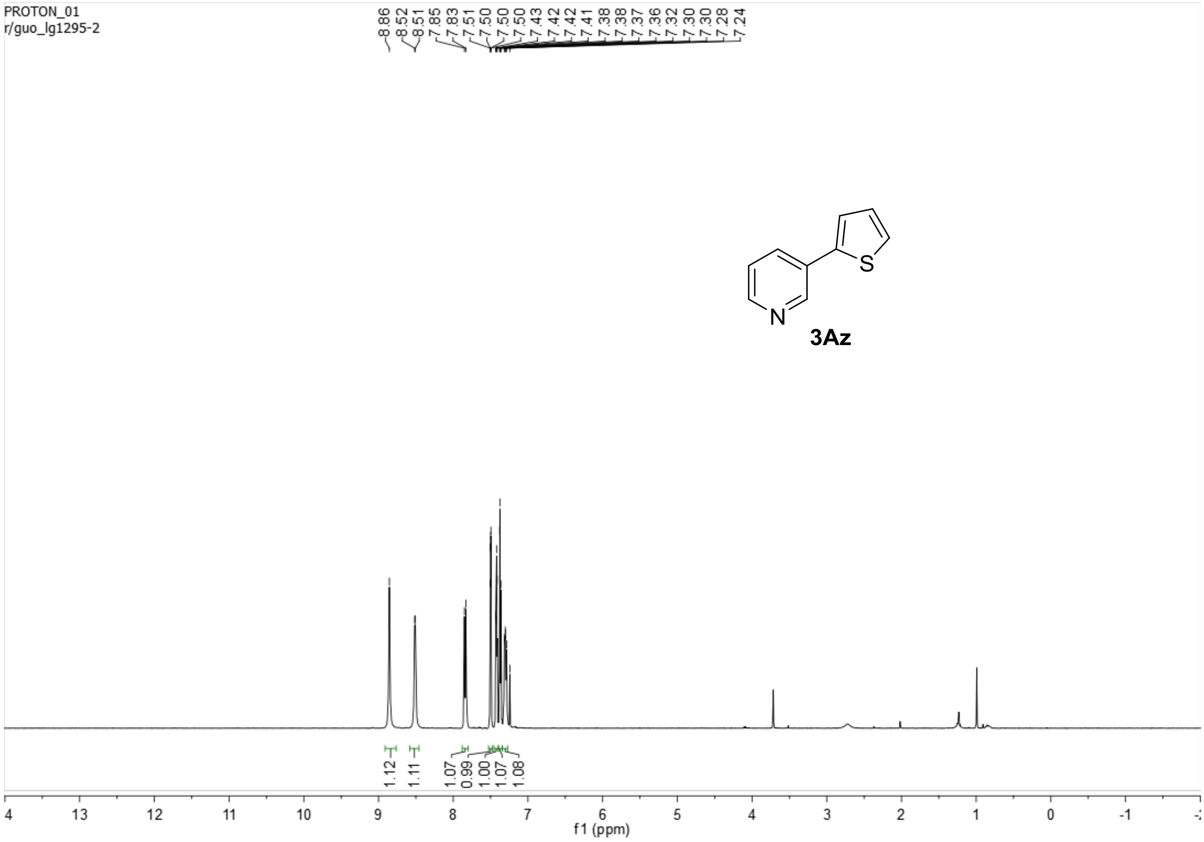

Supplementary Figure 83. ^{1}H NMR spectrum in $CDCl_3$ of compound **3Az**.

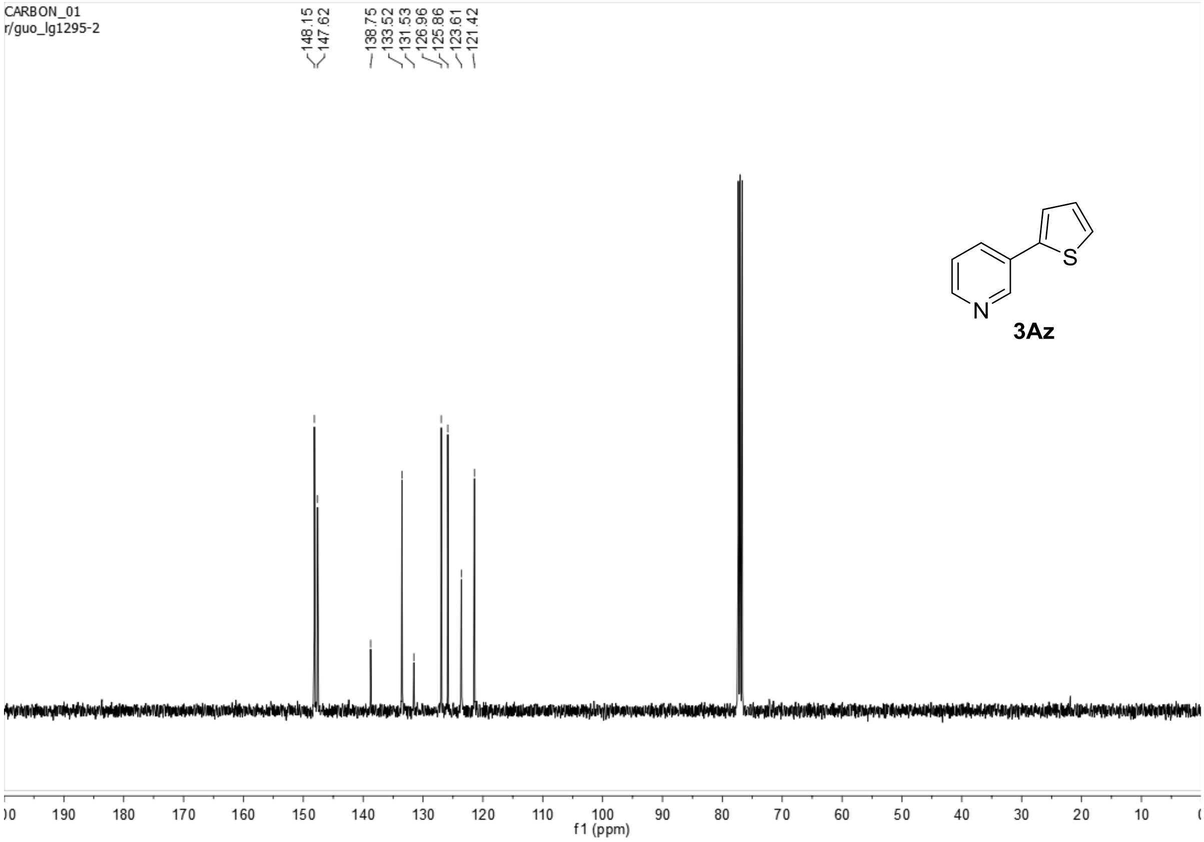

Supplementary Figure 84. ^{13}C NMR spectrum in $CDCl_3$ of compound **3Az**.

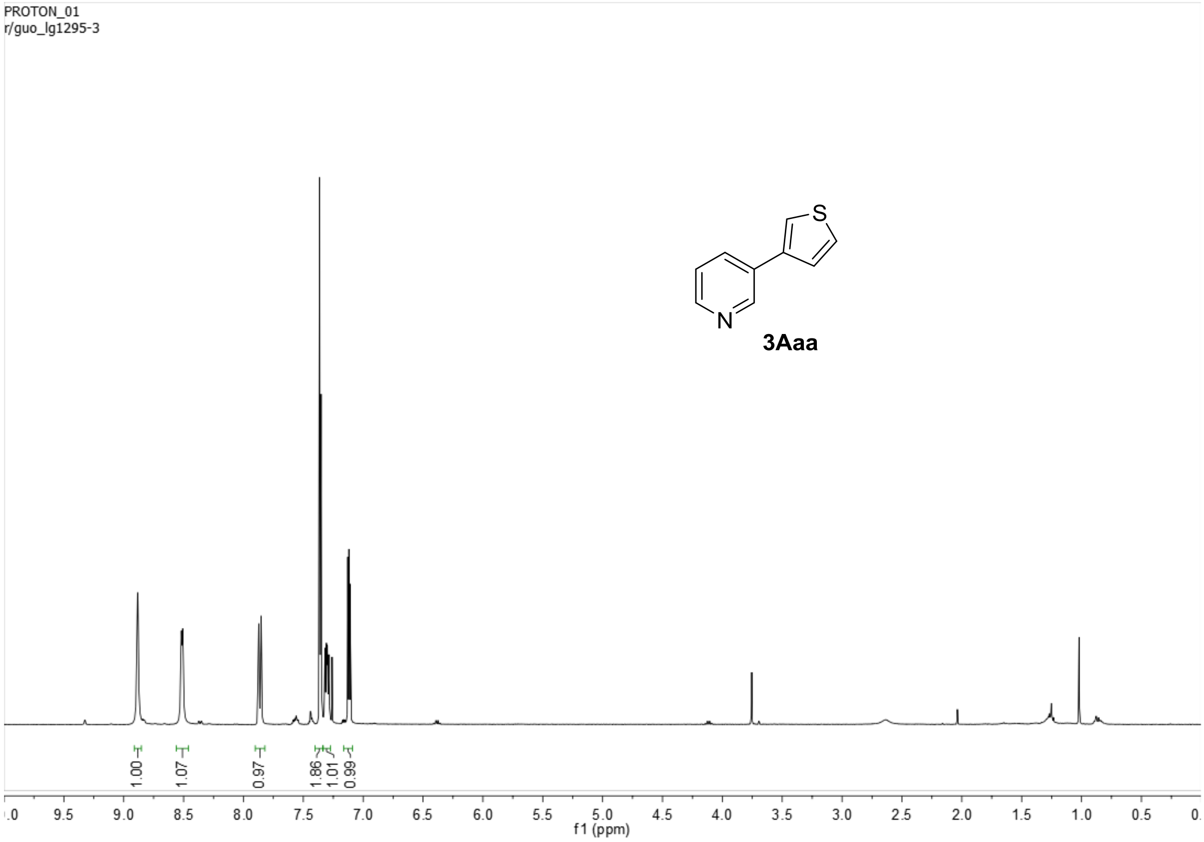

Supplementary Figure 85. ^{1}H NMR spectrum in $CDCl_3$ of compound **3Aaa**.

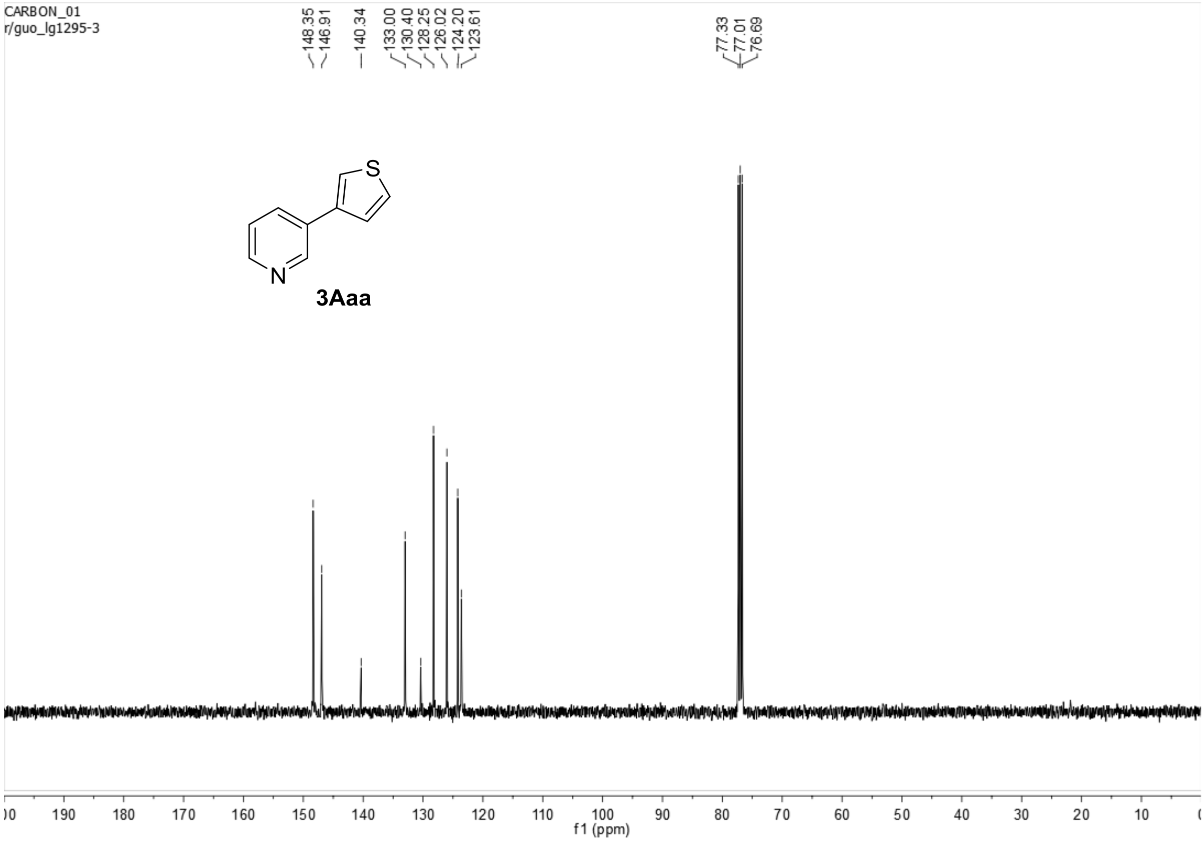

Supplementary Figure 86. ^{13}C NMR spectrum in $CDCl_3$ of compound **3Aaa**.

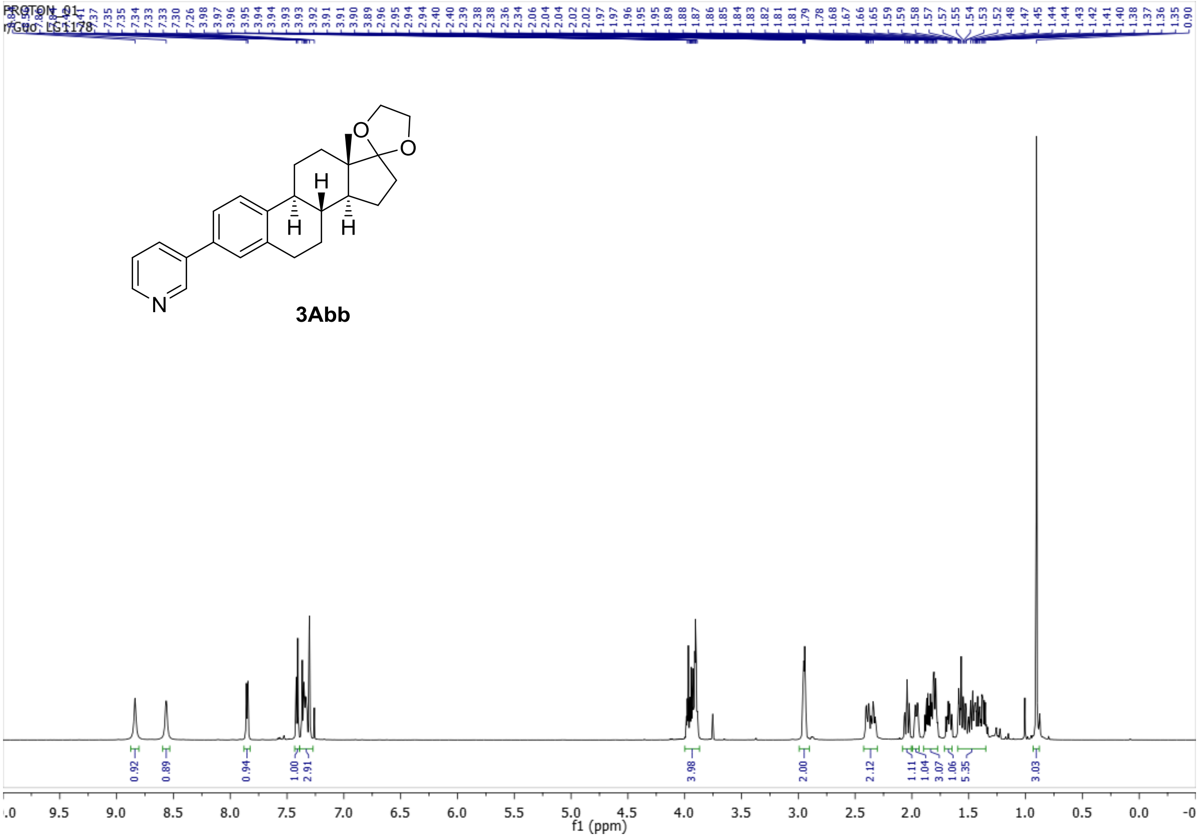

Supplementary Figure 87. ^{1}H NMR spectrum in $CDCl_3$ of compound **3Abb**.

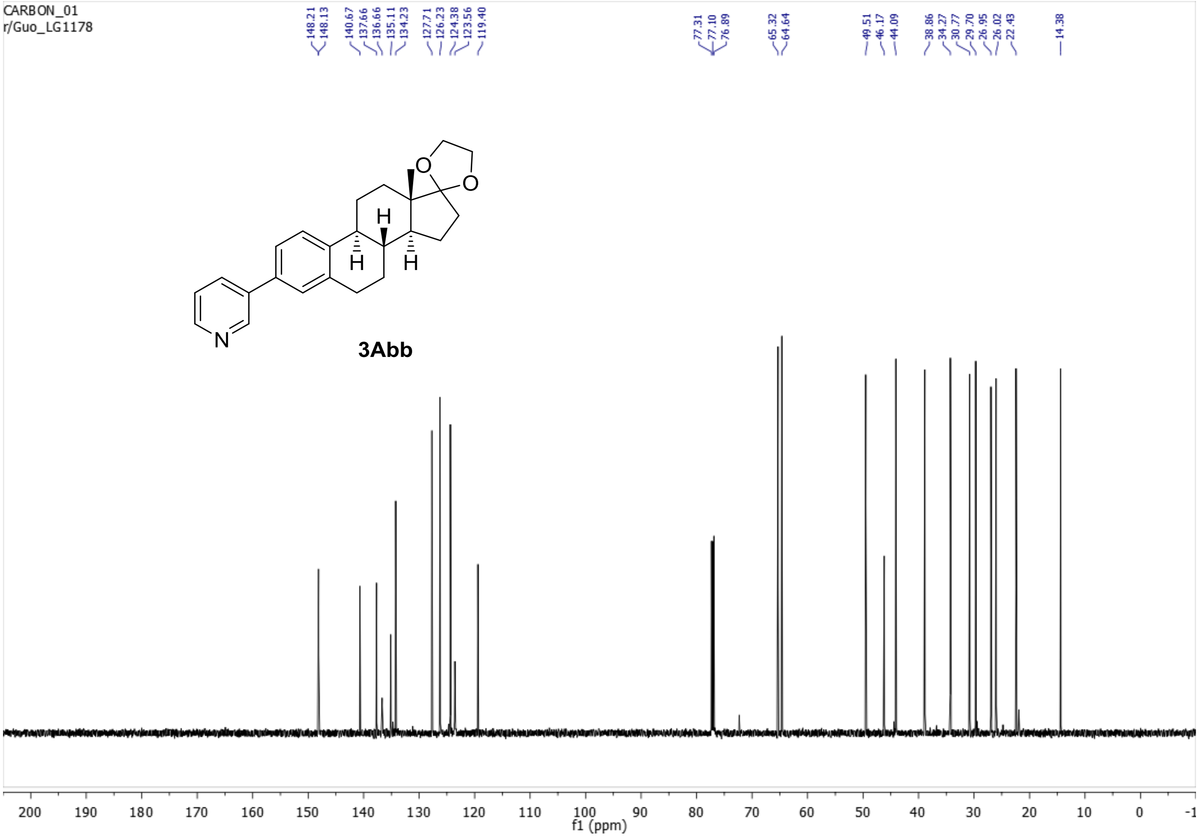

Supplementary Figure 88. ^{13}C NMR spectrum in $CDCl_3$ of compound **3Abb**.

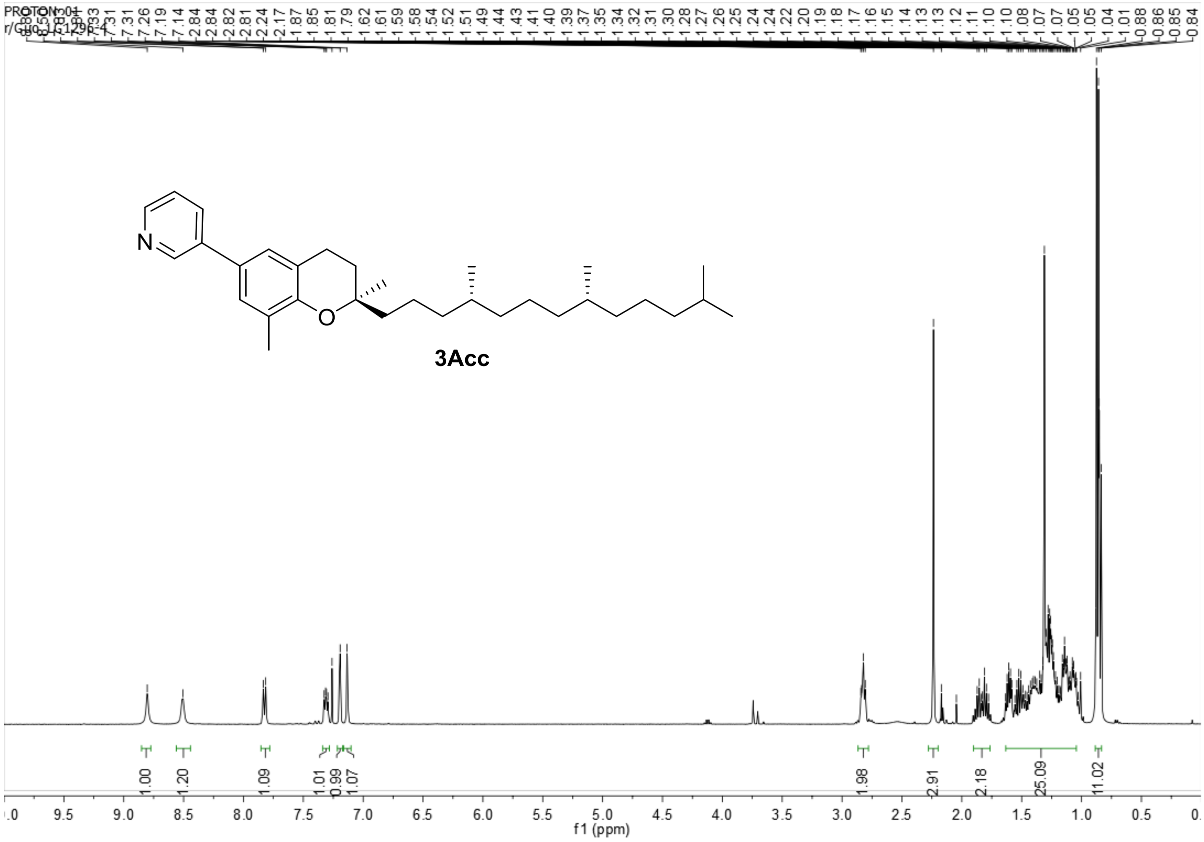

Supplementary Figure 89. ^{1}H NMR spectrum in $CDCl_3$ of compound **3Acc**.

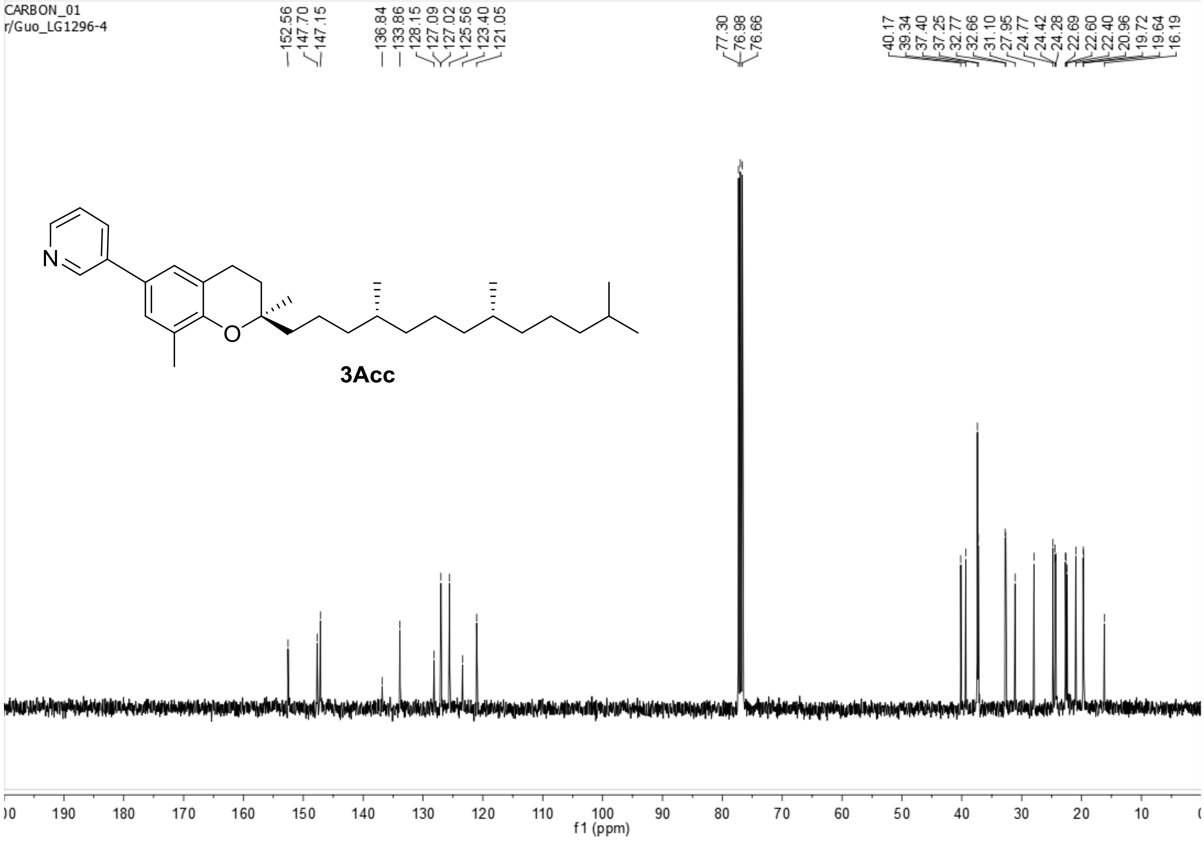

Supplementary Figure 90. ^{13}C NMR spectrum in $CDCl_3$ of compound **3Acc**.

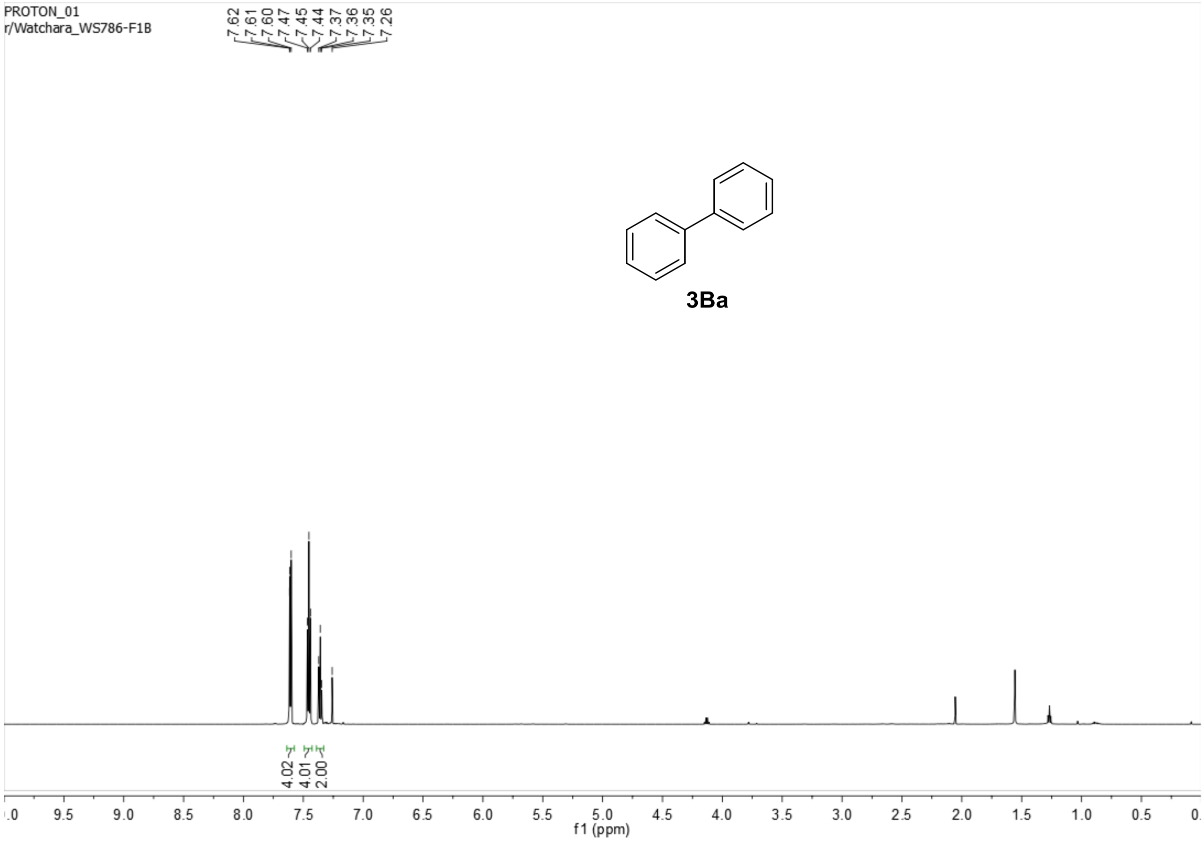

Supplementary Figure 91. ^{1}H NMR spectrum in $CDCl_3$ of compound **3Ba**.

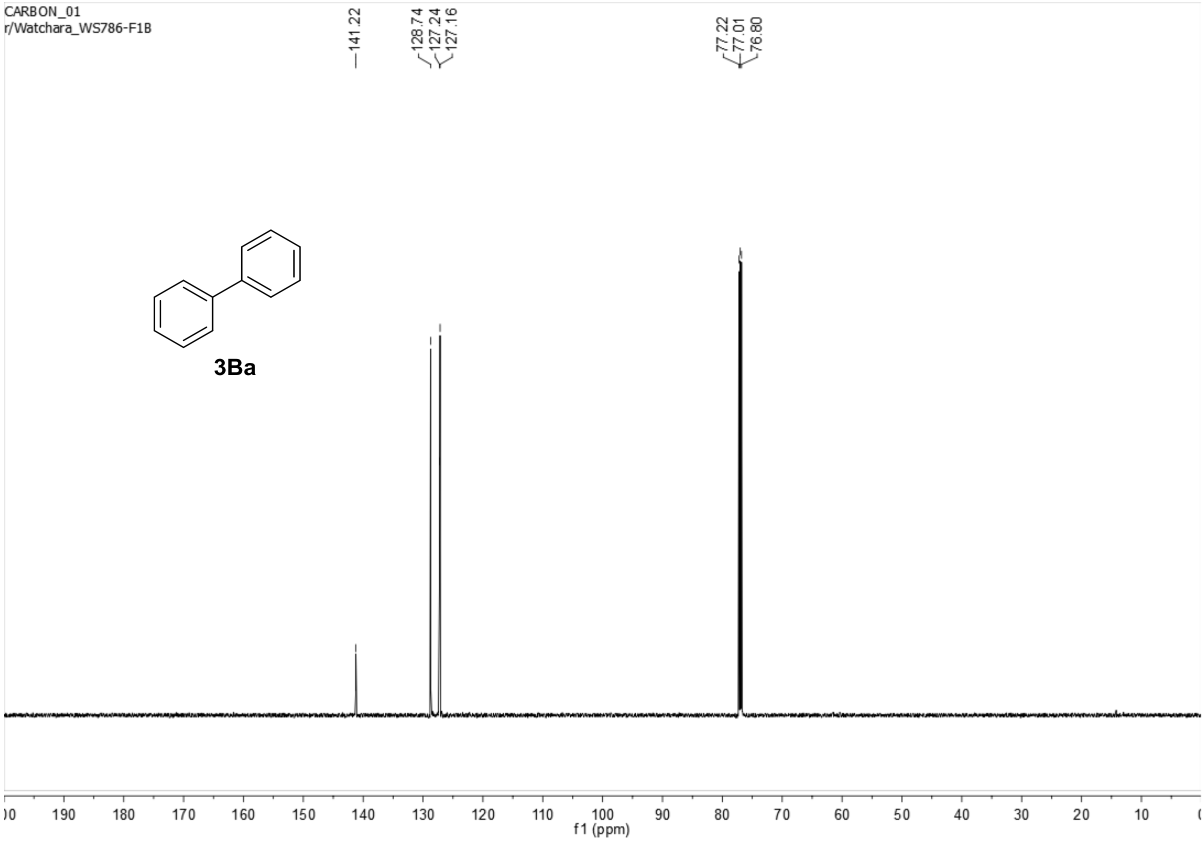

Supplementary Figure 92. ^{13}C NMR spectrum in $CDCl_3$ of compound **3Ba**.

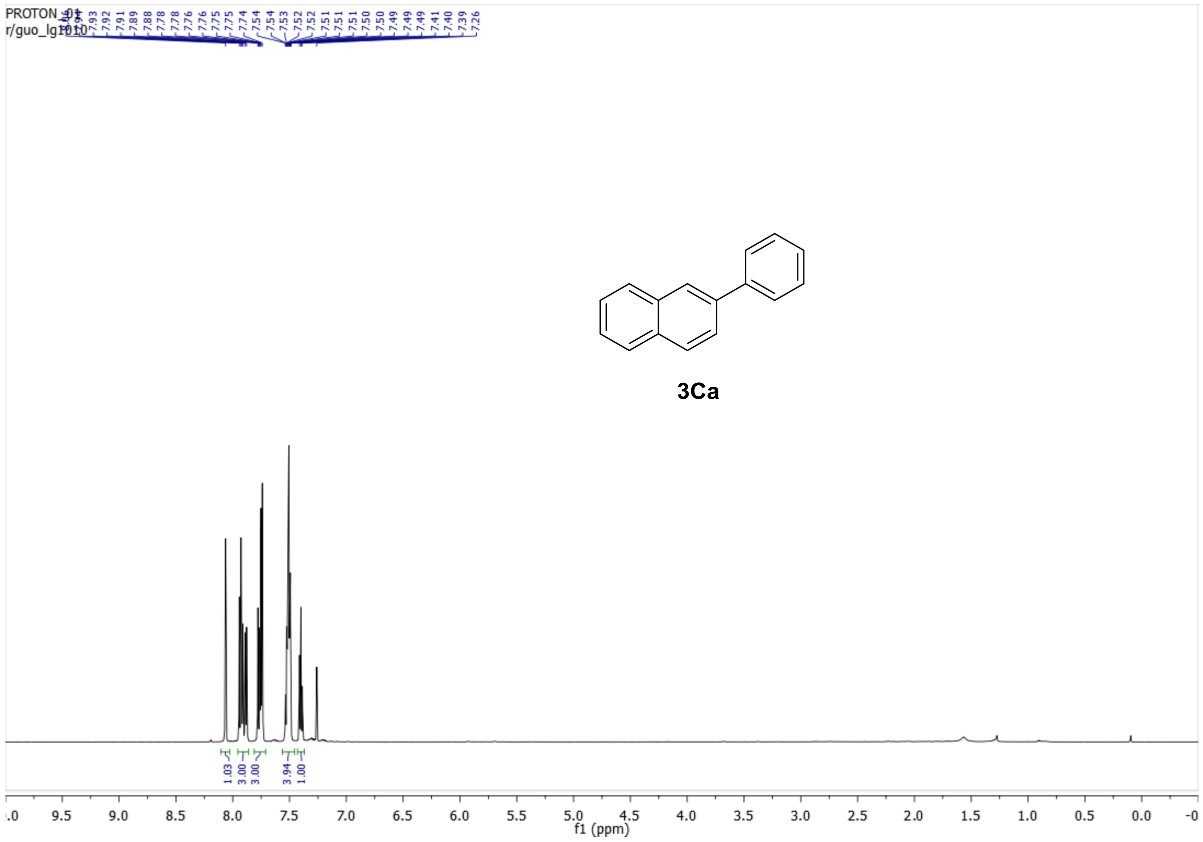

Supplementary Figure 93. ^{1}H NMR spectrum in $CDCl_3$ of compound **3Ca**.

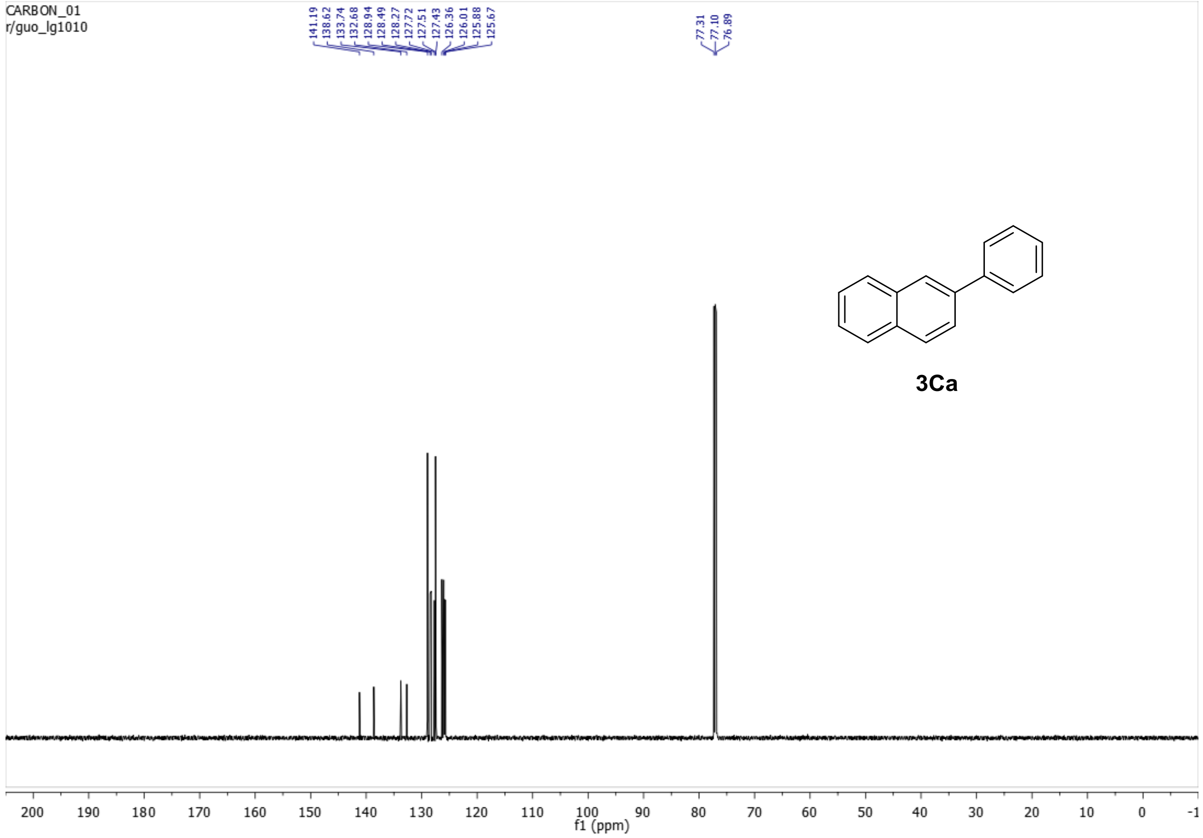

Supplementary Figure 94. ^{13}C NMR spectrum in $CDCl_3$ of compound **3Ca**.

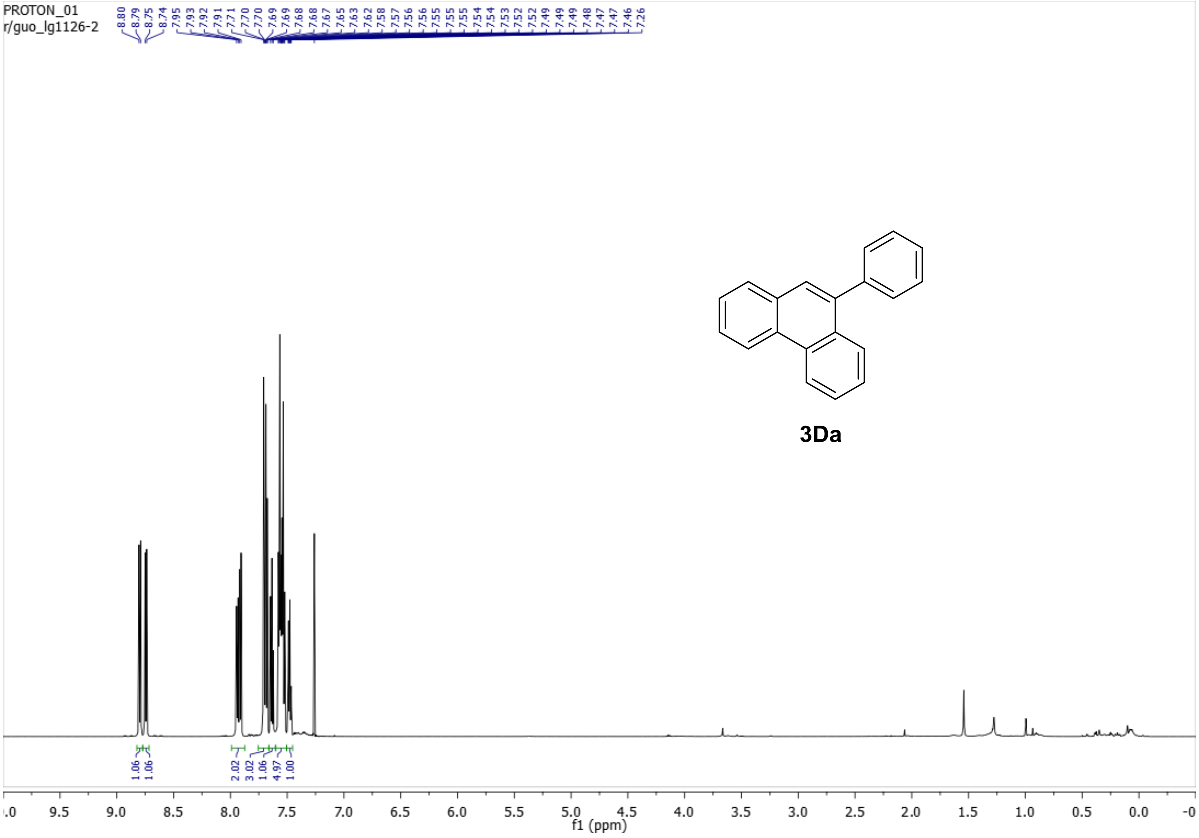

Supplementary Figure 95. ^{1}H NMR spectrum in $CDCl_3$ of compound **3Da**.

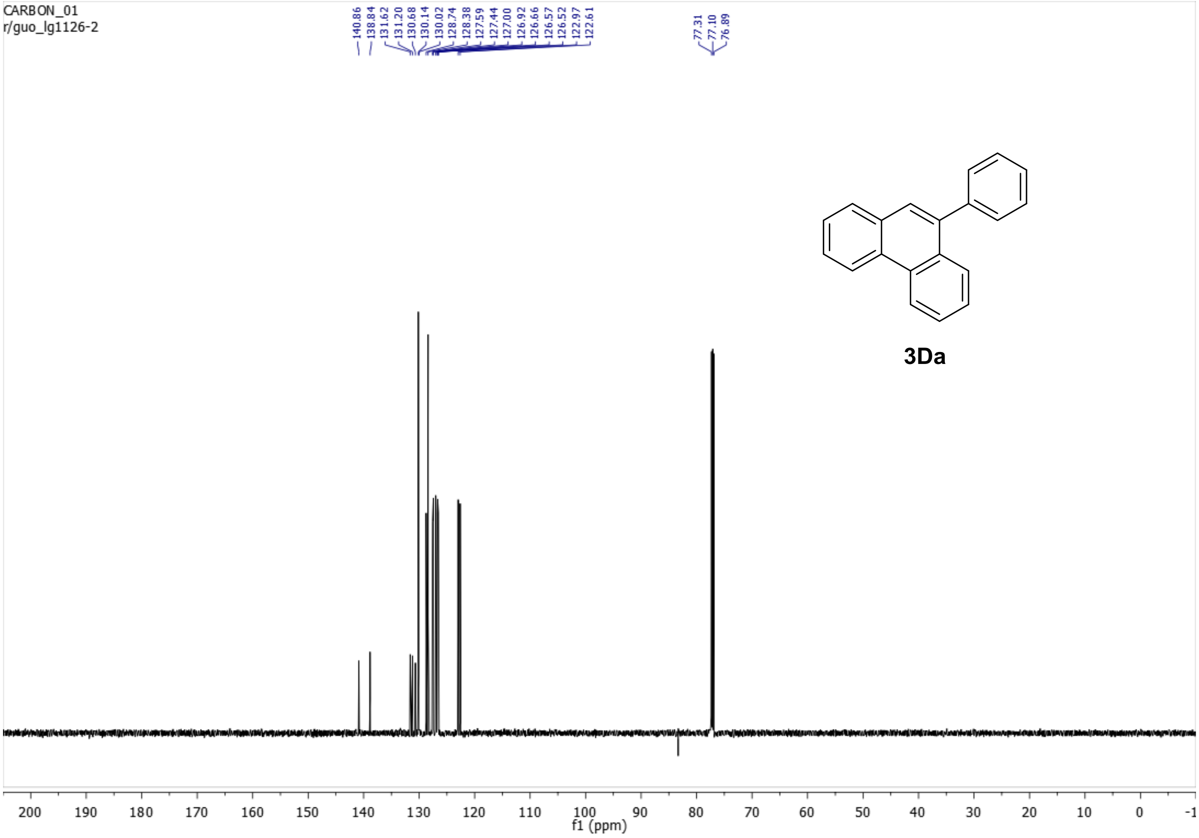

Supplementary Figure 96. ^{13}C NMR spectrum in $CDCl_3$ of compound **3Da**.

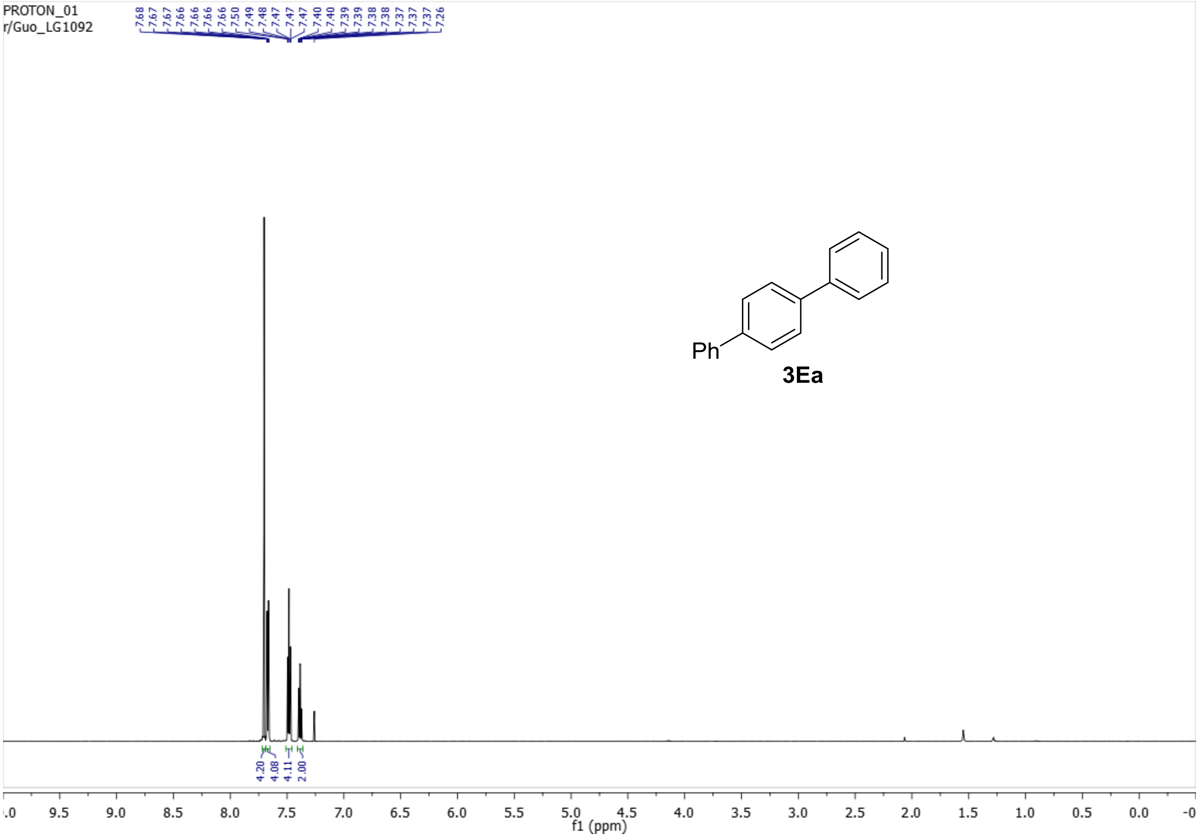

Supplementary Figure 97. ^{1}H NMR spectrum in $CDCl_3$ of compound **3Ea**.

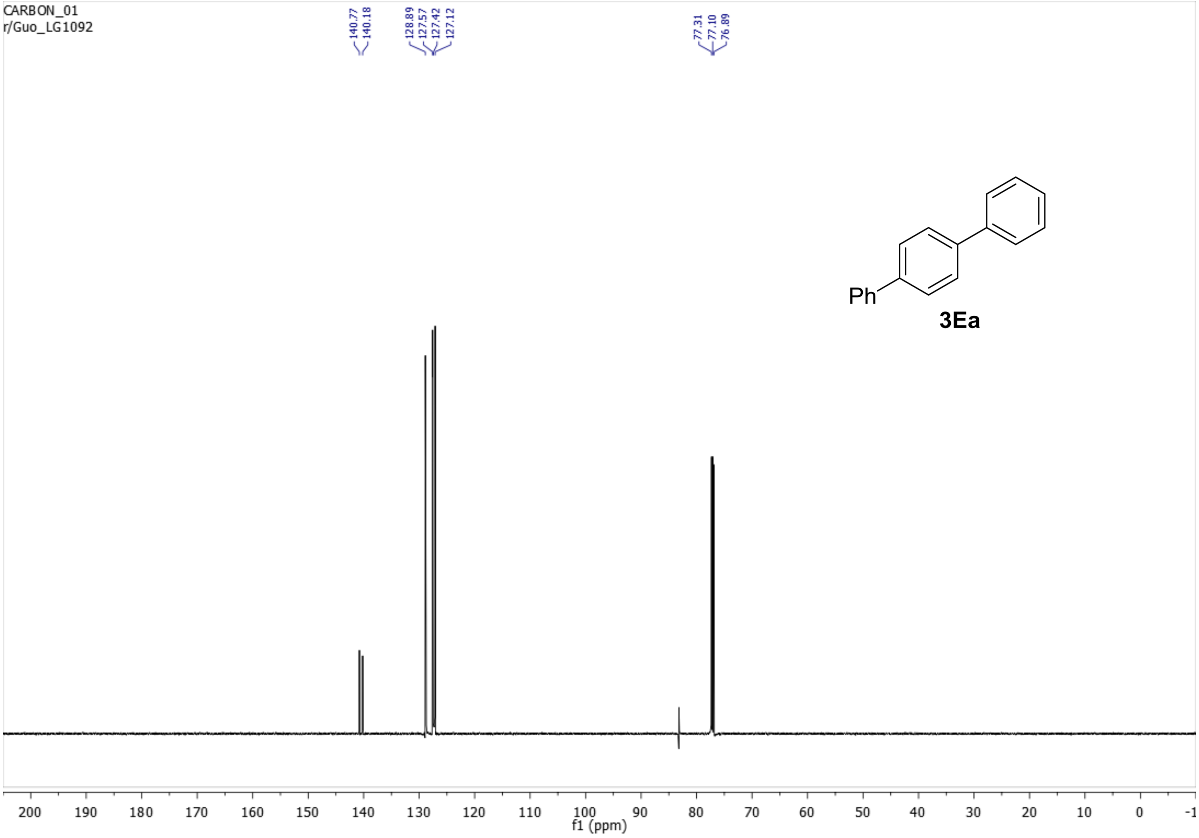

Supplementary Figure 98. ^{13}C NMR spectrum in $CDCl_3$ of compound **3Ea**.

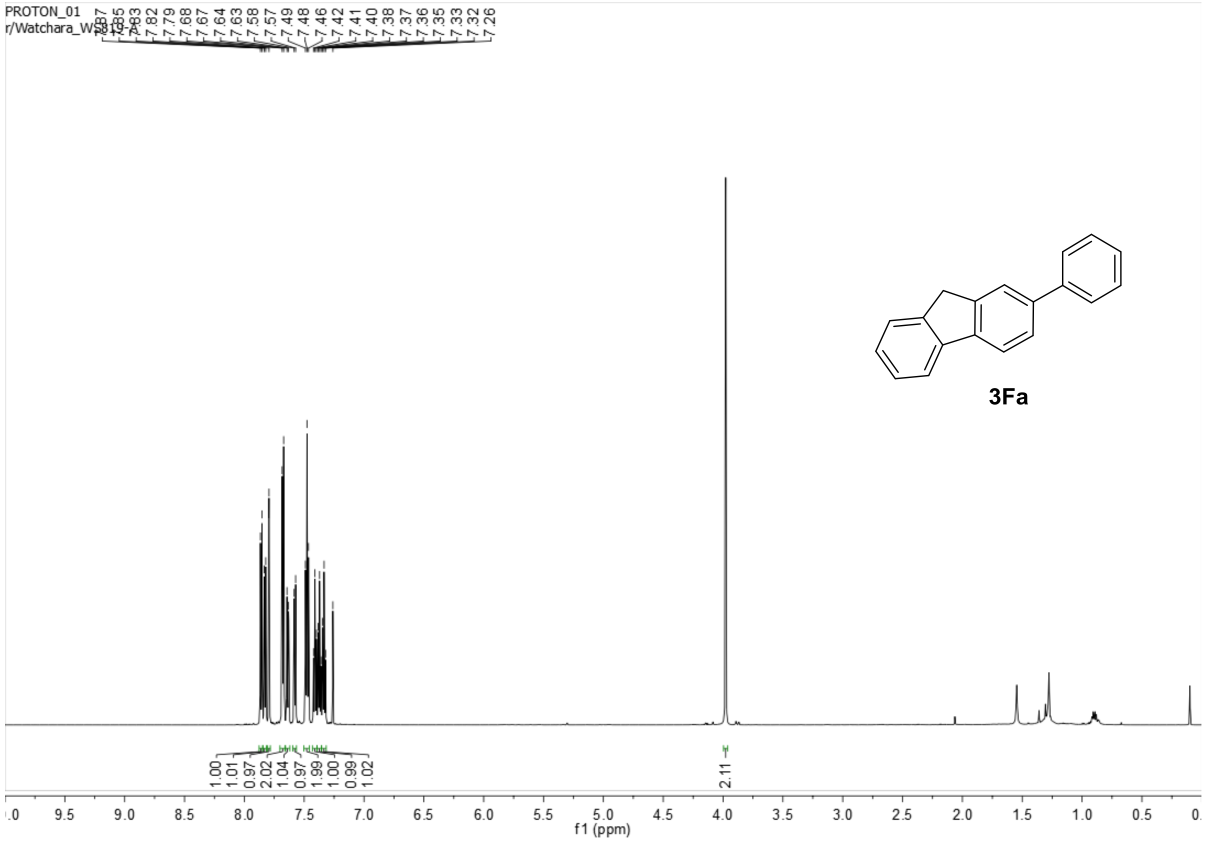

Supplementary Figure 99. ^{1}H NMR spectrum in $CDCl_3$ of compound **3Fa**.

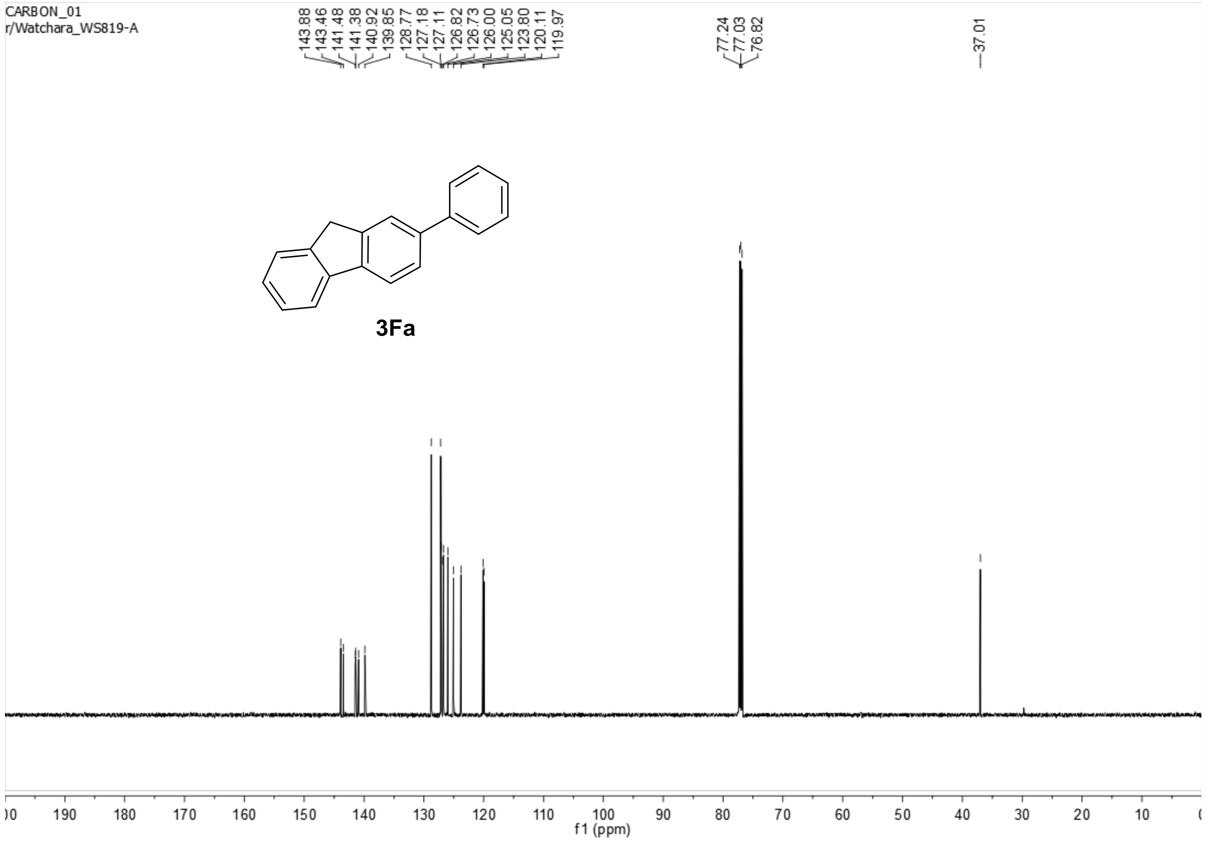

Supplementary Figure 100. ^{13}C NMR spectrum in $CDCl_3$ of compound **3Fa**.

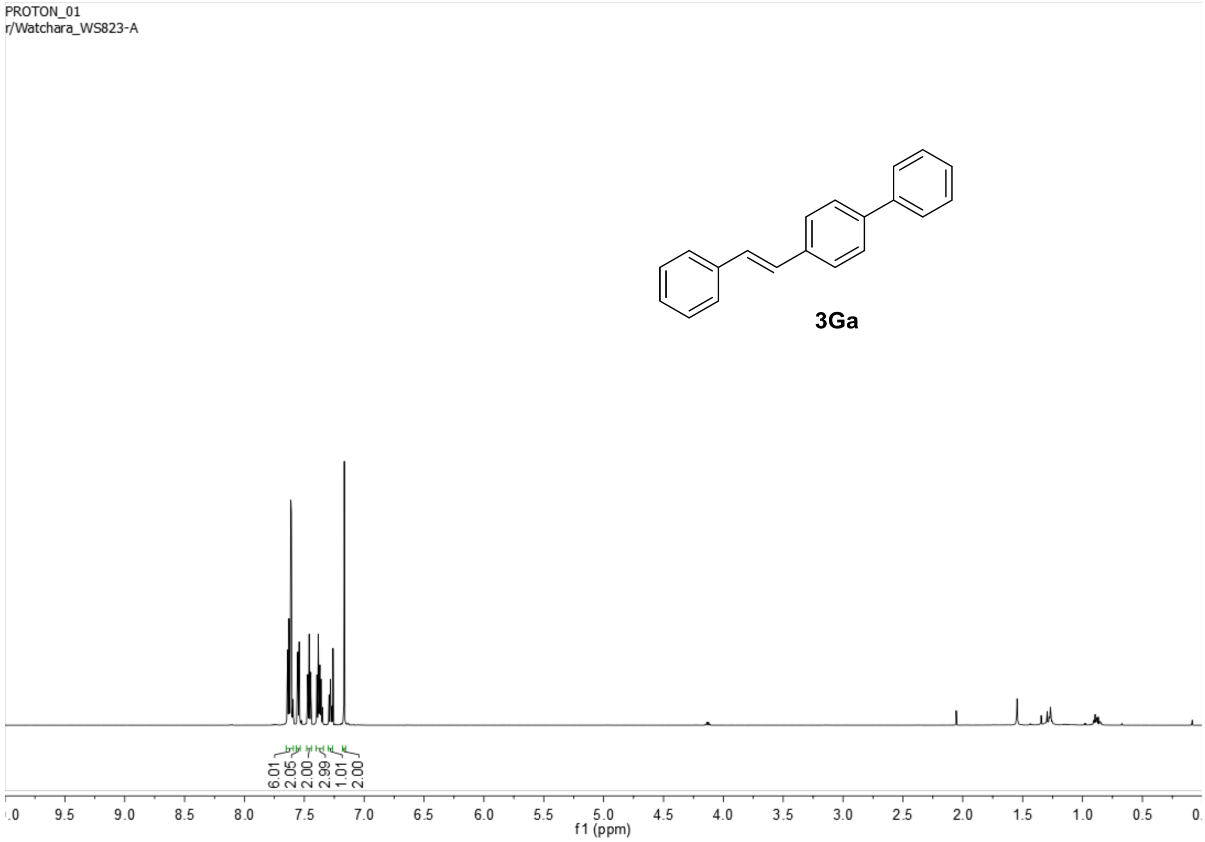

Supplementary Figure 101. ^{1}H NMR spectrum in $CDCl_3$ of compound **3Ga**.

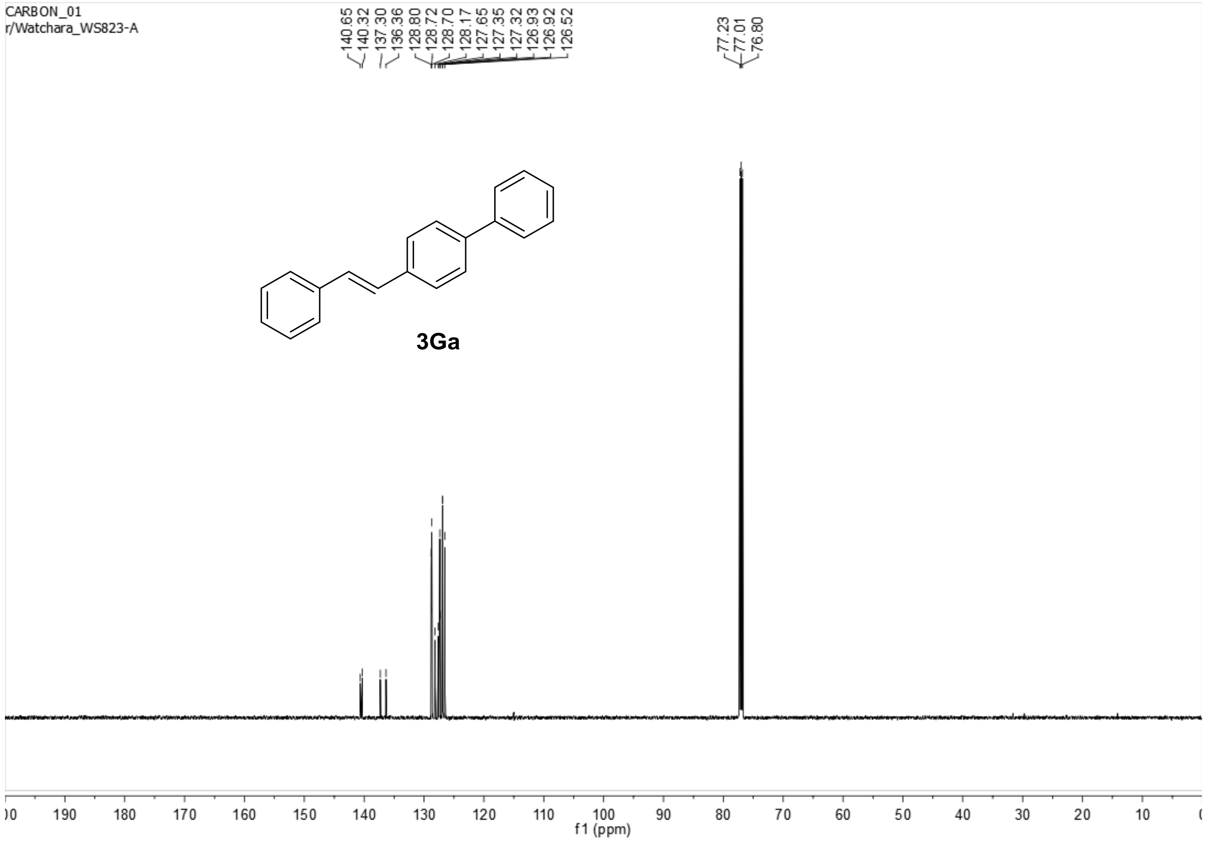

Supplementary Figure 102. ^{13}C NMR spectrum in $CDCl_3$ of compound **3Ga**.

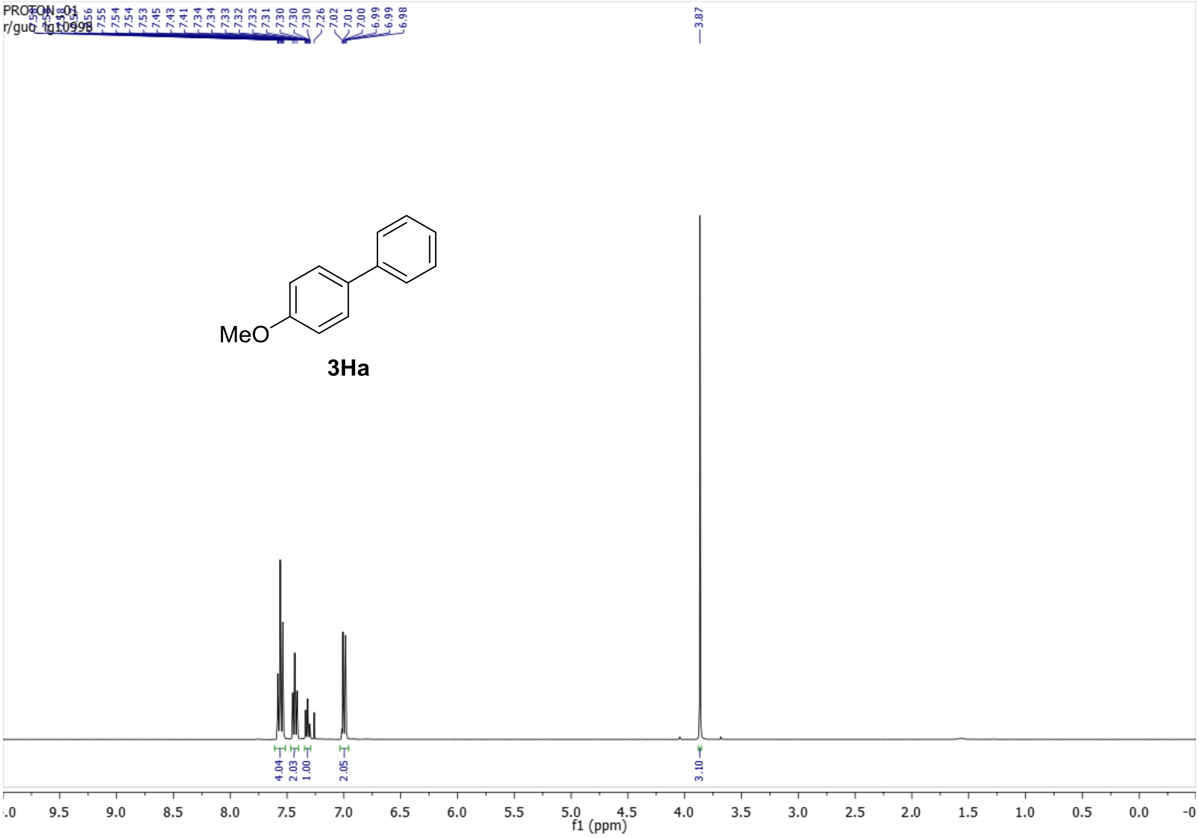

Supplementary Figure 103. ^{1}H NMR spectrum in $CDCl_3$ of compound **3Ha**.

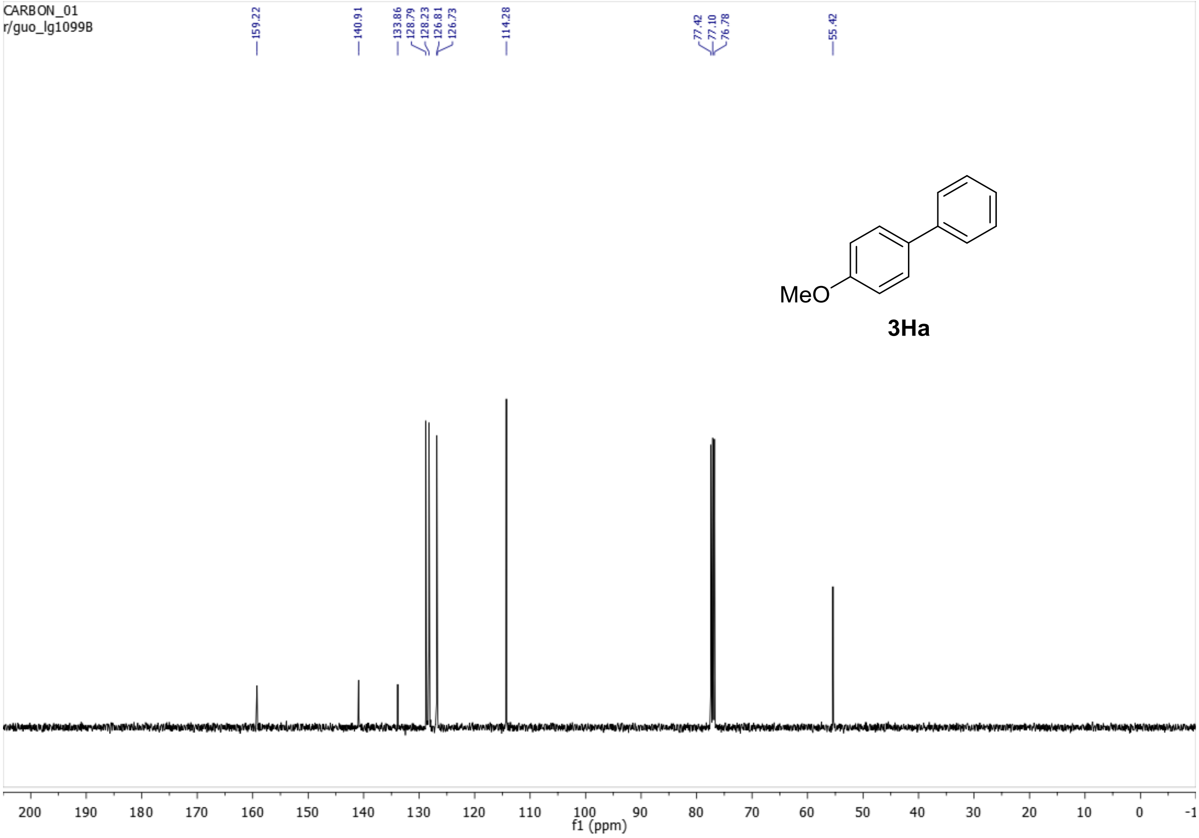

Supplementary Figure 104. ^{13}C NMR spectrum in $CDCl_3$ of compound **3Ha**.

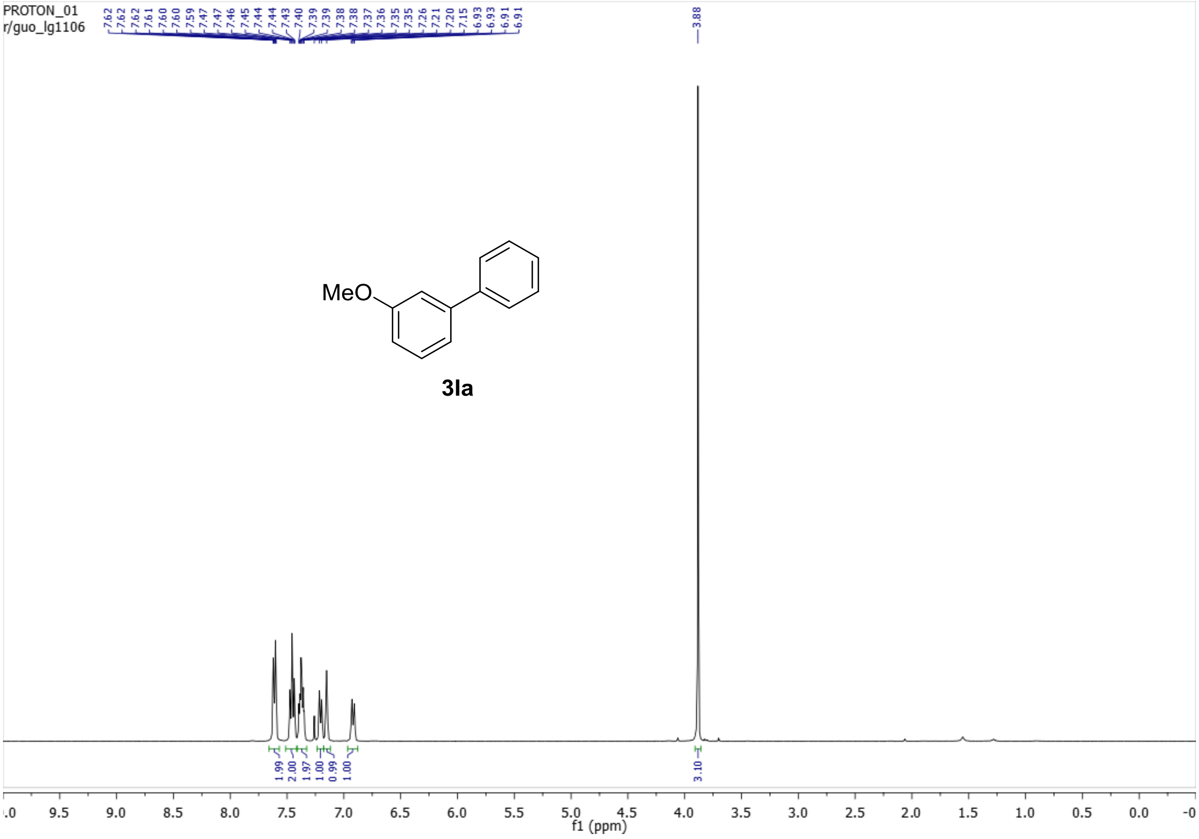

Supplementary Figure 105. ^{1}H NMR spectrum in $CDCl_3$ of compound **3Ia**.

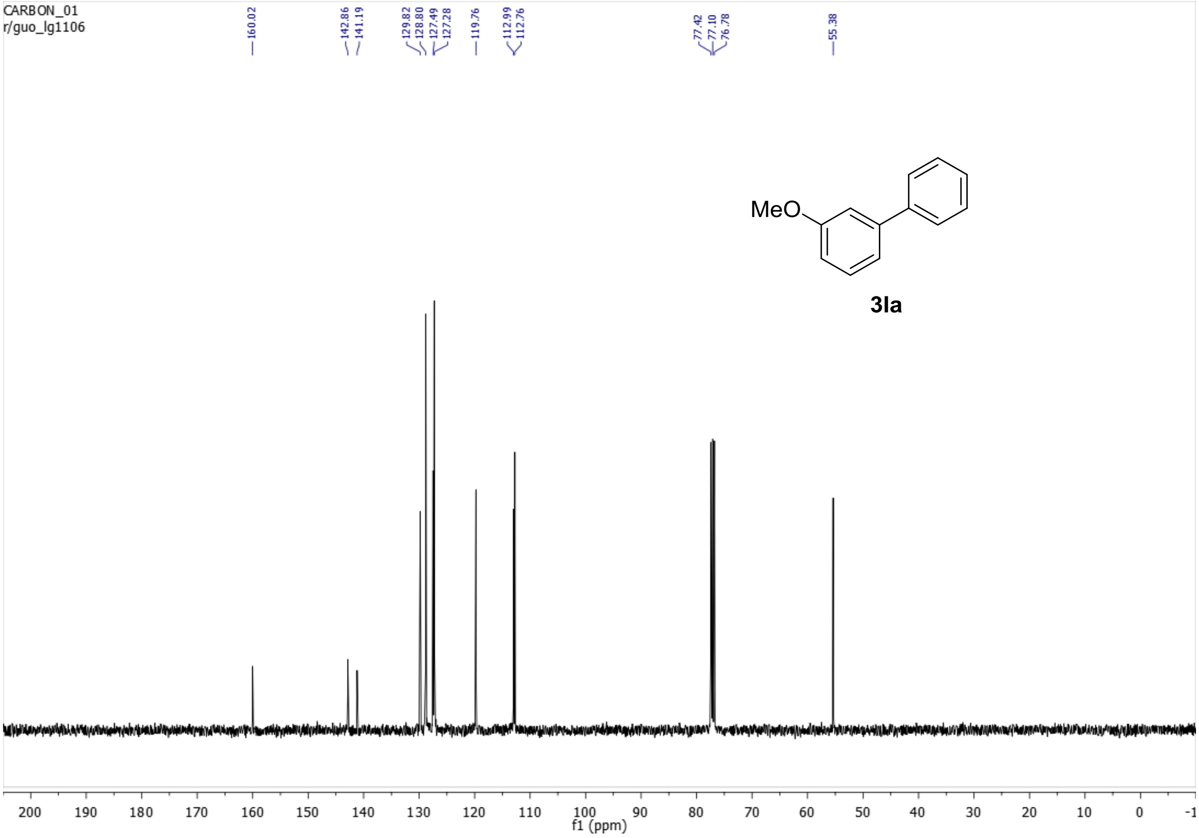

Supplementary Figure 106. ^{13}C NMR spectrum in $CDCl_3$ of compound **3Ia**.

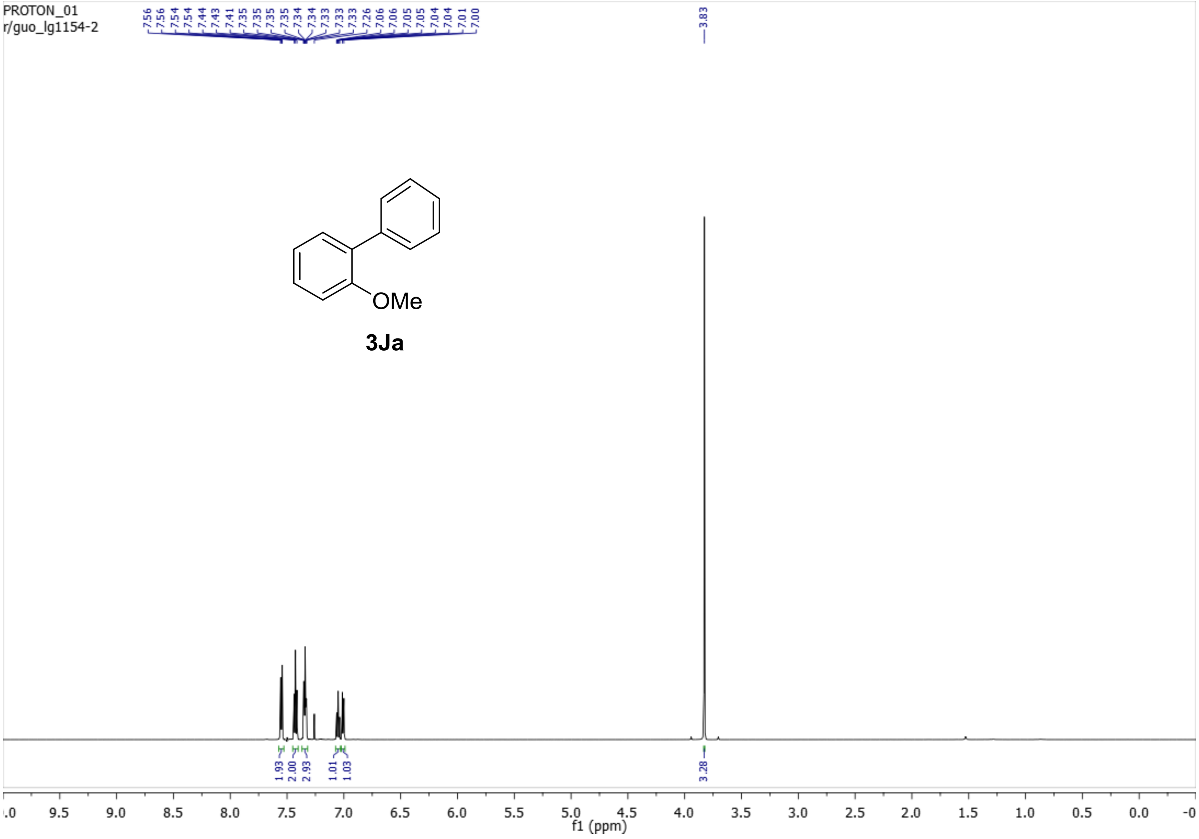

Supplementary Figure 107. ^{1}H NMR spectrum in $CDCl_3$ of compound **3Ja**.

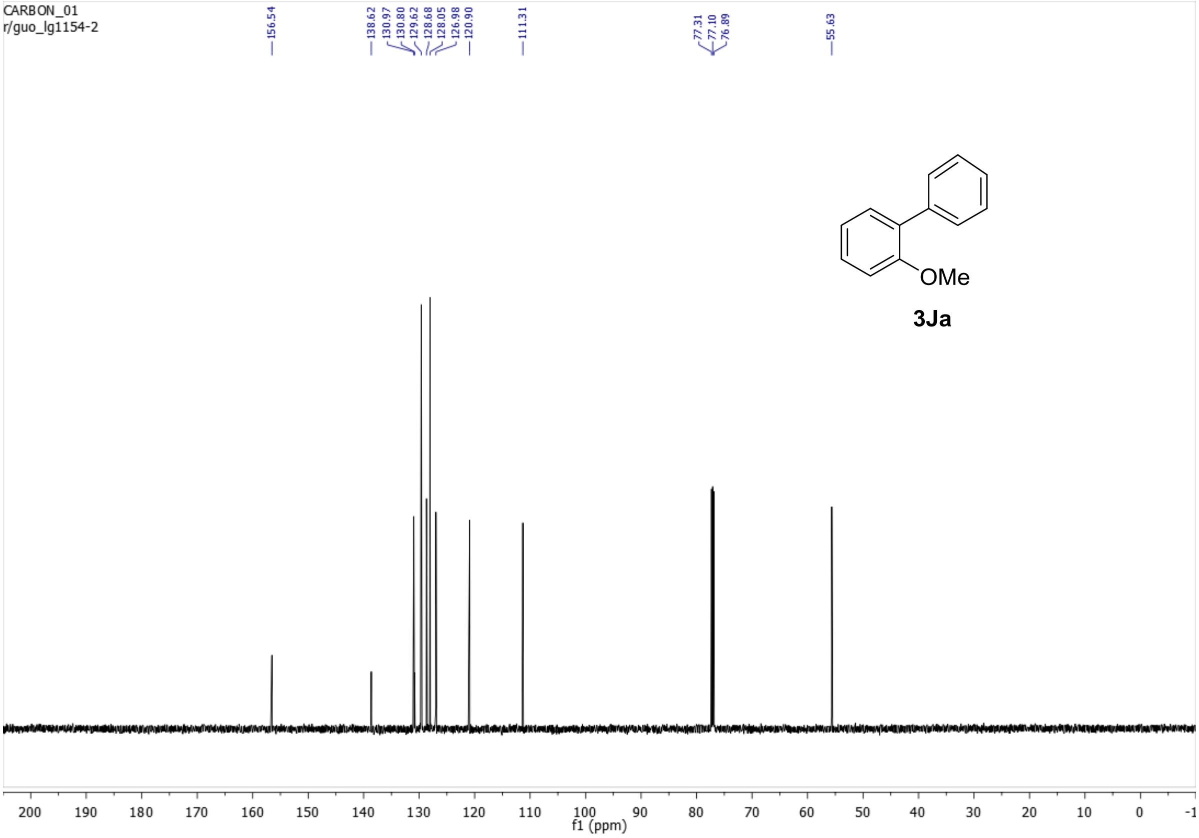

Supplementary Figure 108. ^{13}C NMR spectrum in $CDCl_3$ of compound **3Ja**.

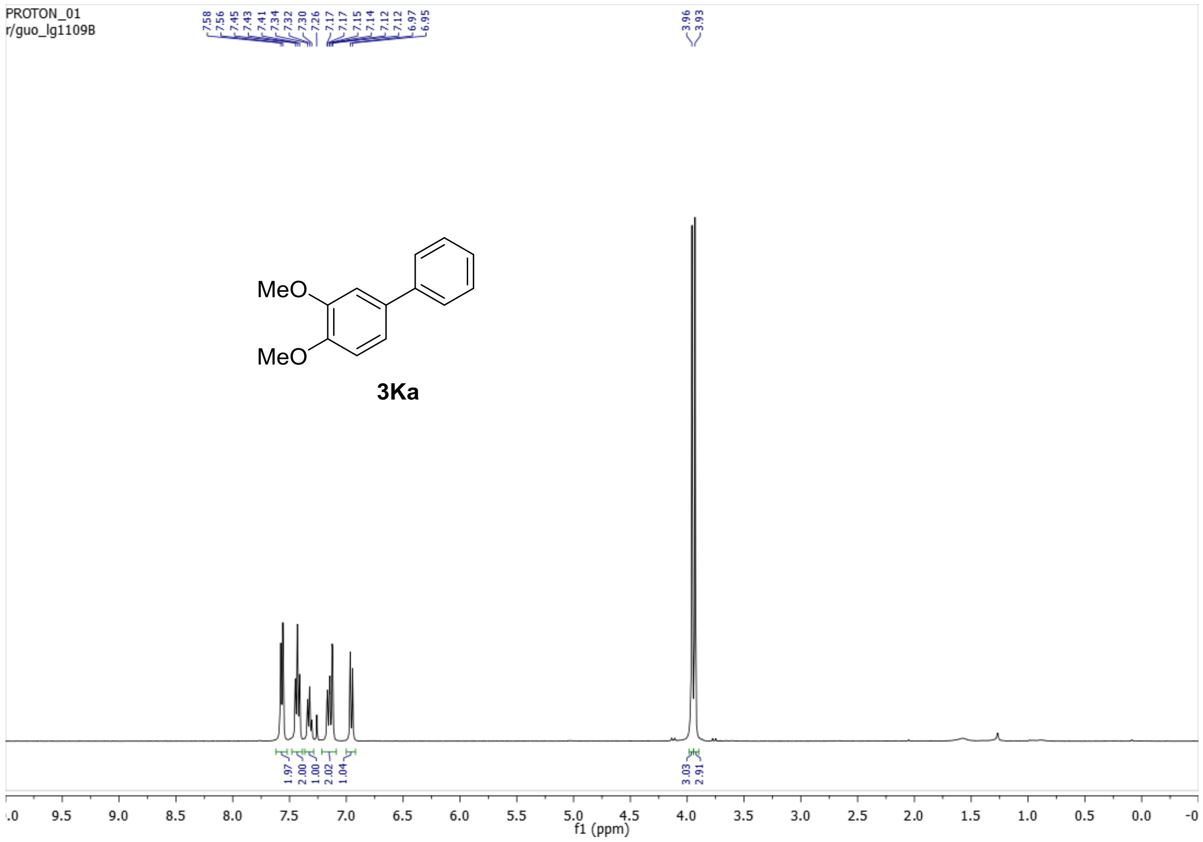

Supplementary Figure 109. ^{1}H NMR spectrum in $CDCl_3$ of compound **3Ka**.

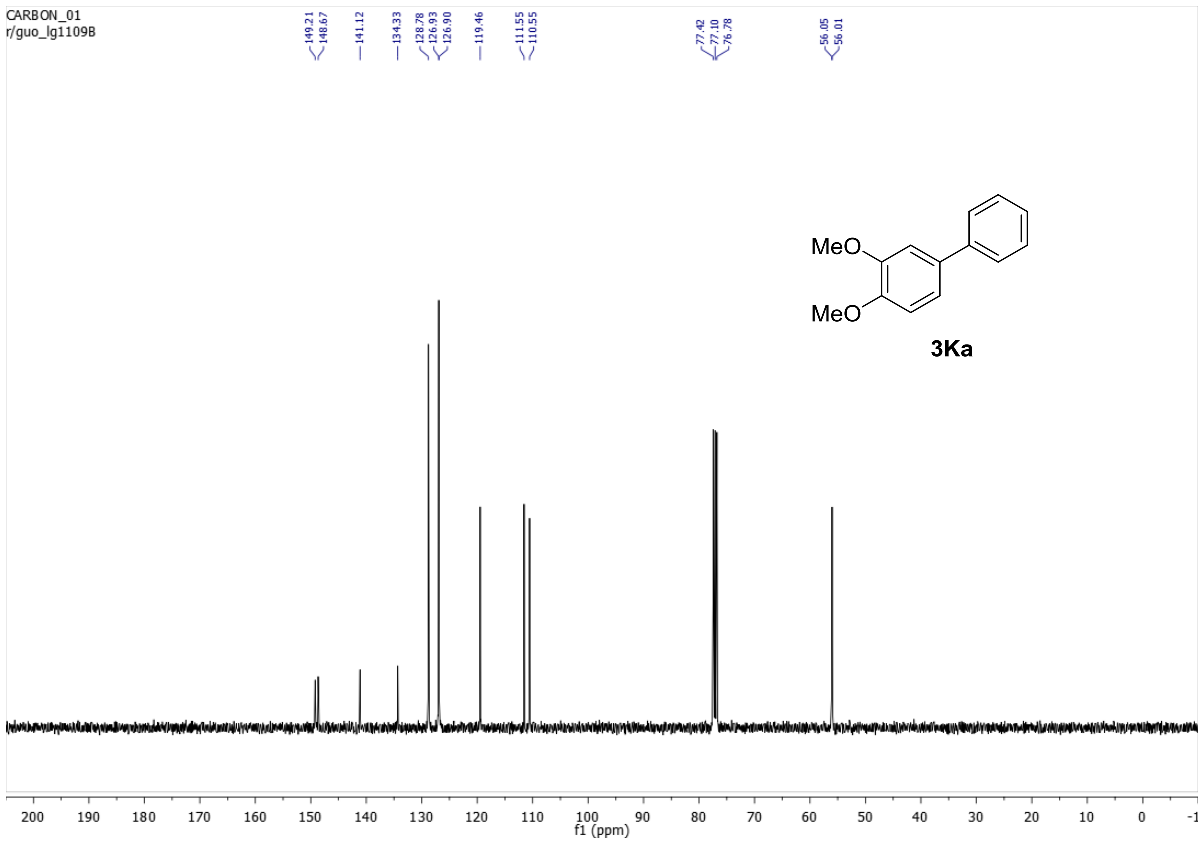

Supplementary Figure 110. ^{13}C NMR spectrum in $CDCl_3$ of compound **3Ka**.

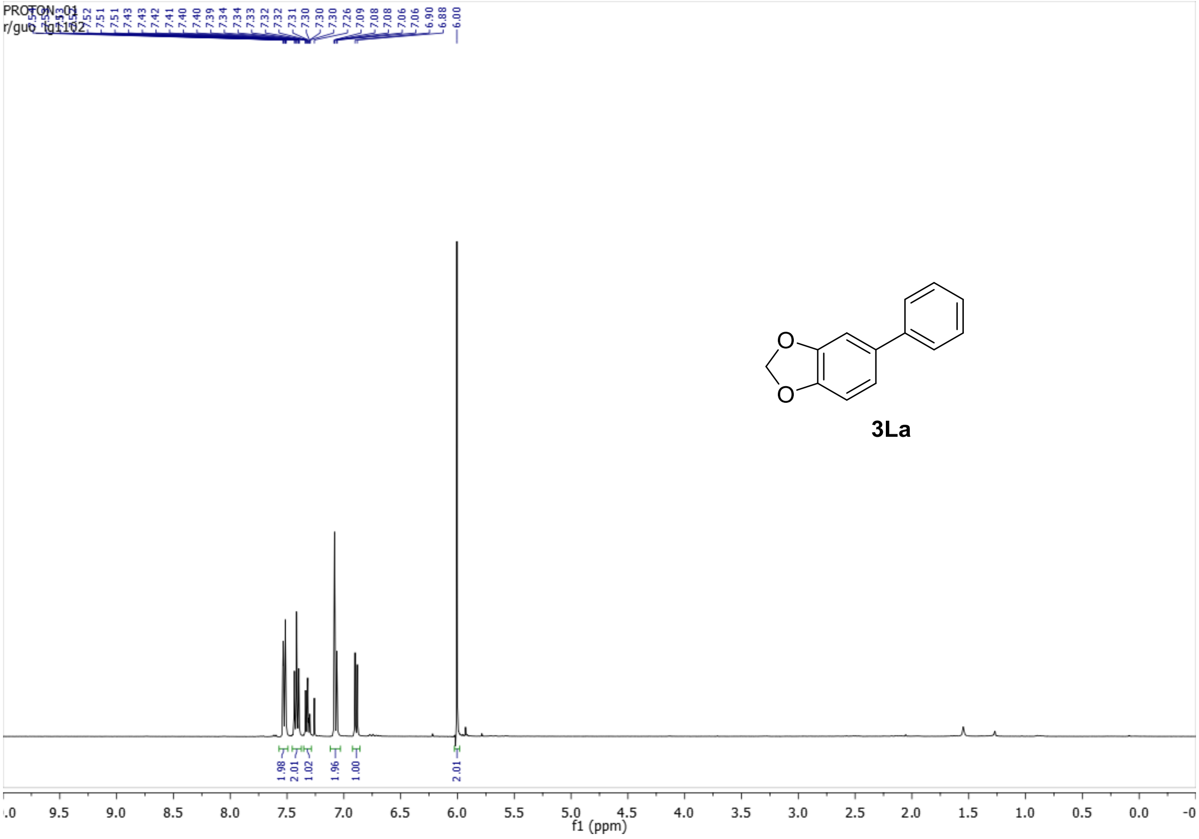

Supplementary Figure 111. ^{1}H NMR spectrum in $CDCl_3$ of compound **3La**.

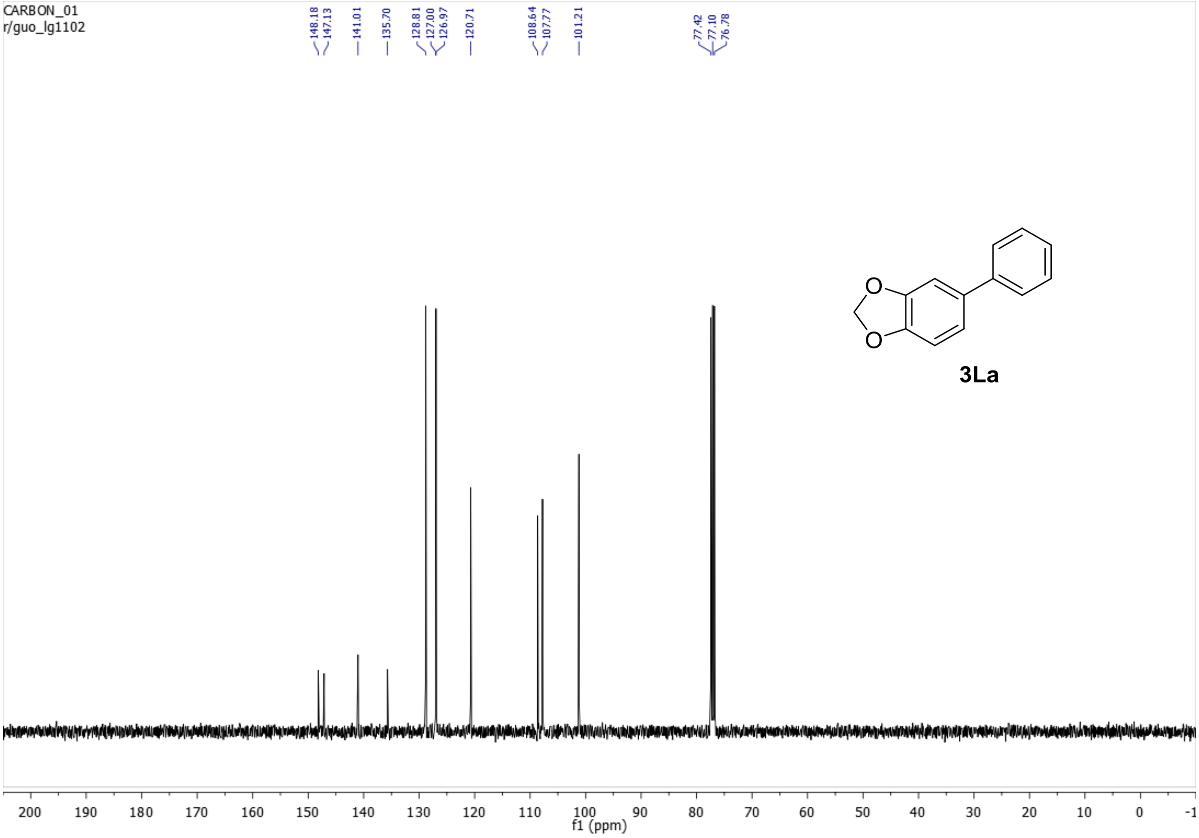

Supplementary Figure 112. ^{13}C NMR spectrum in $CDCl_3$ of compound **3La**.

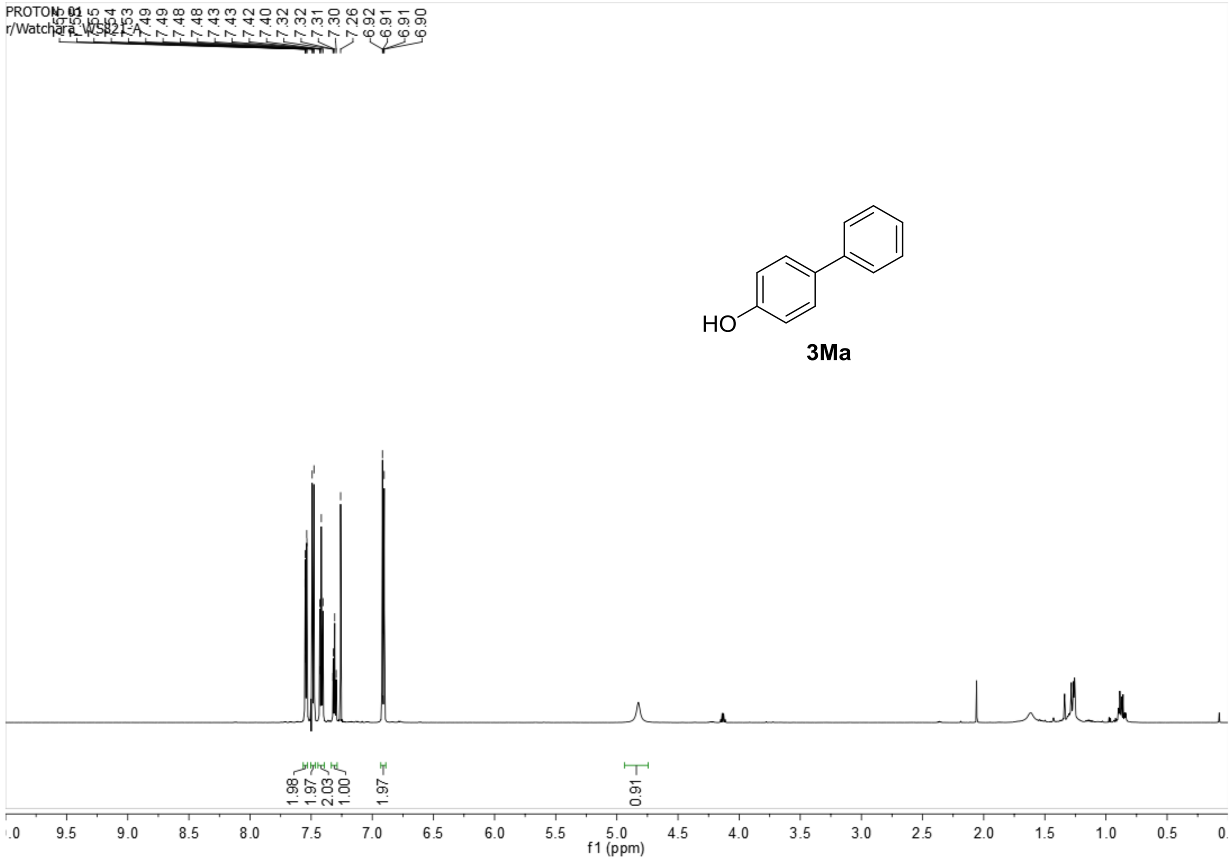

Supplementary Figure 113. ^{1}H NMR spectrum in $CDCl_3$ of compound **3Ma**.

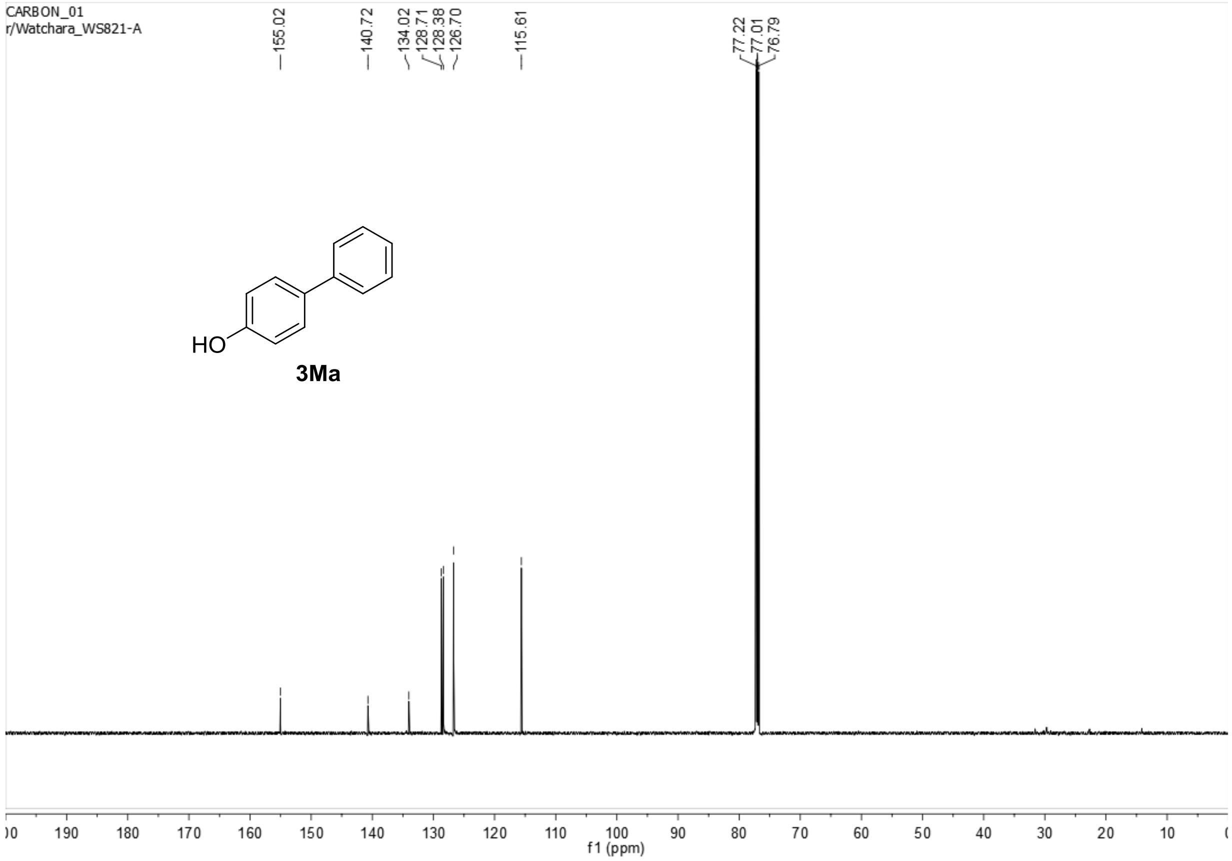

Supplementary Figure 114. ^{13}C NMR spectrum in $CDCl_3$ of compound **3Ma**.

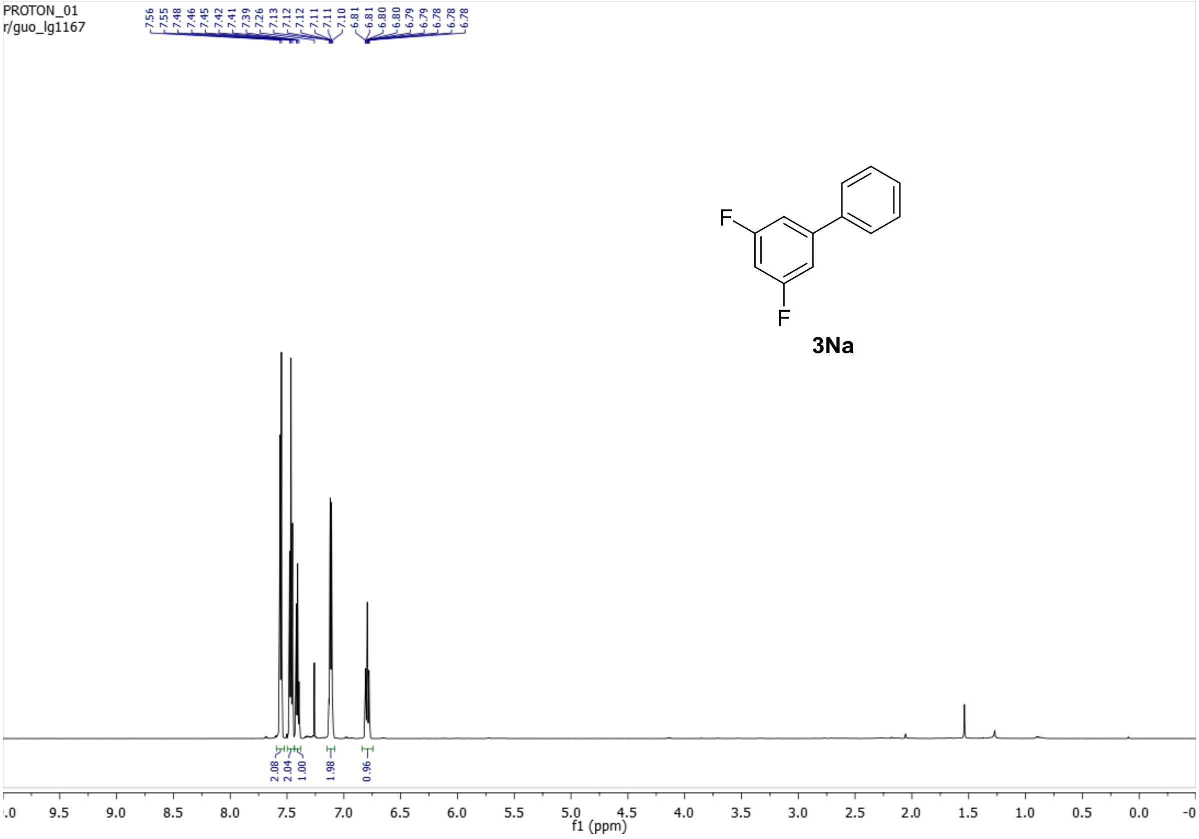

Supplementary Figure 115. ^{1}H NMR spectrum in $CDCl_3$ of compound **3Na**.

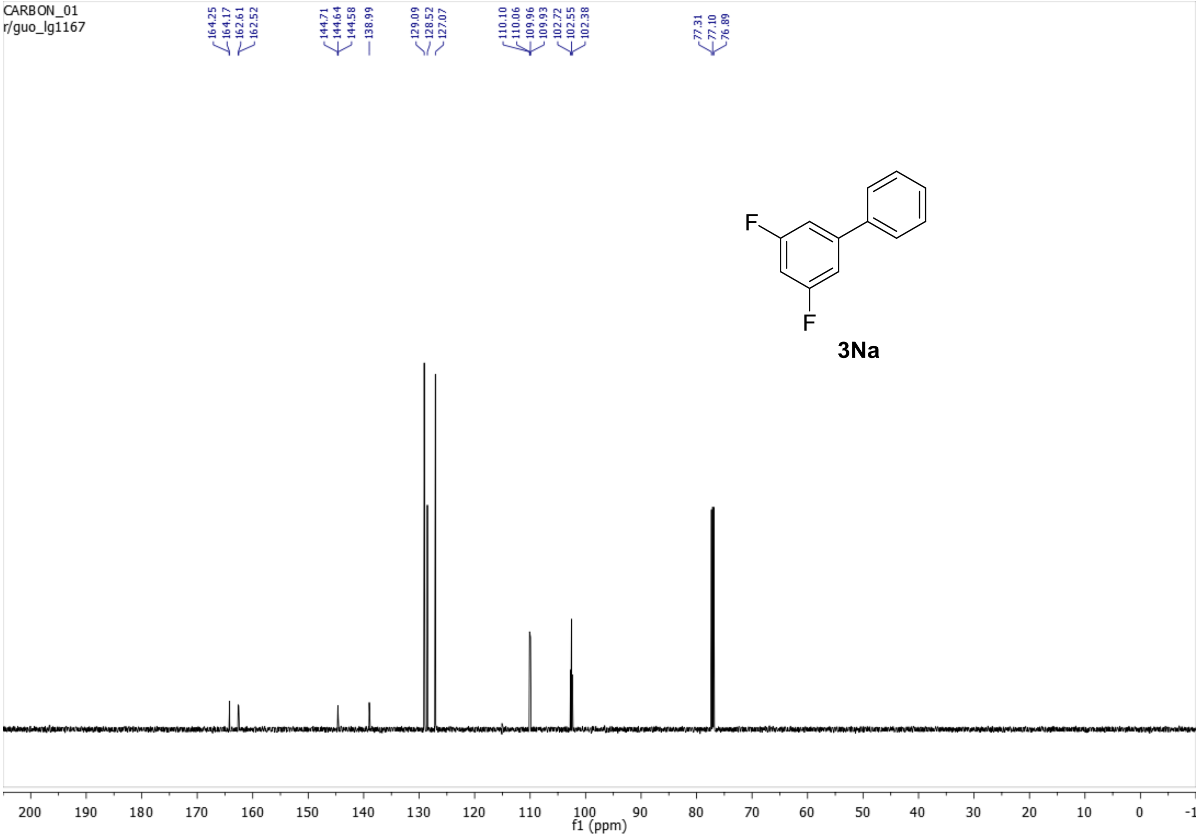

Supplementary Figure 116. ^{13}C NMR spectrum in $CDCl_3$ of compound **3Na**.

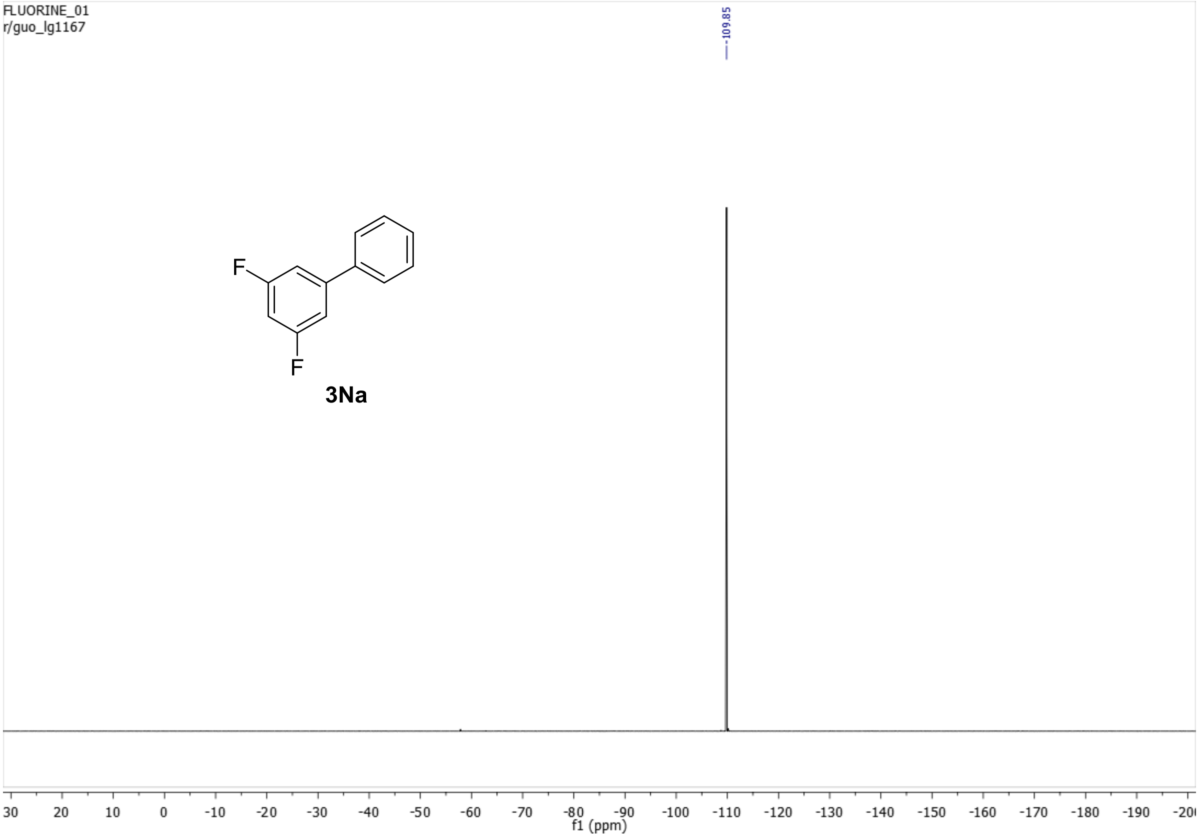

Supplementary Figure 117. ^{19}F NMR spectrum in $CDCl_3$ of compound **3Na**.

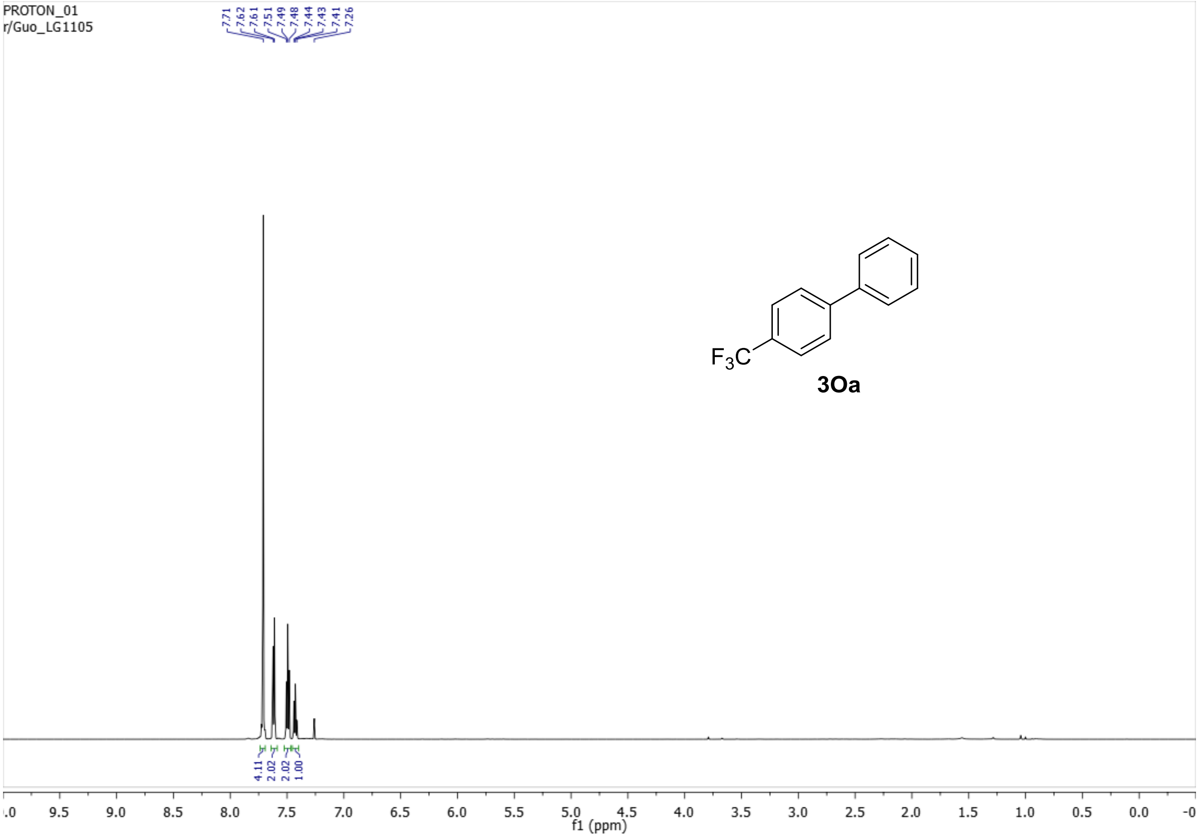

Supplementary Figure 118. ^{1}H NMR spectrum in $CDCl_3$ of compound **3Oa**.

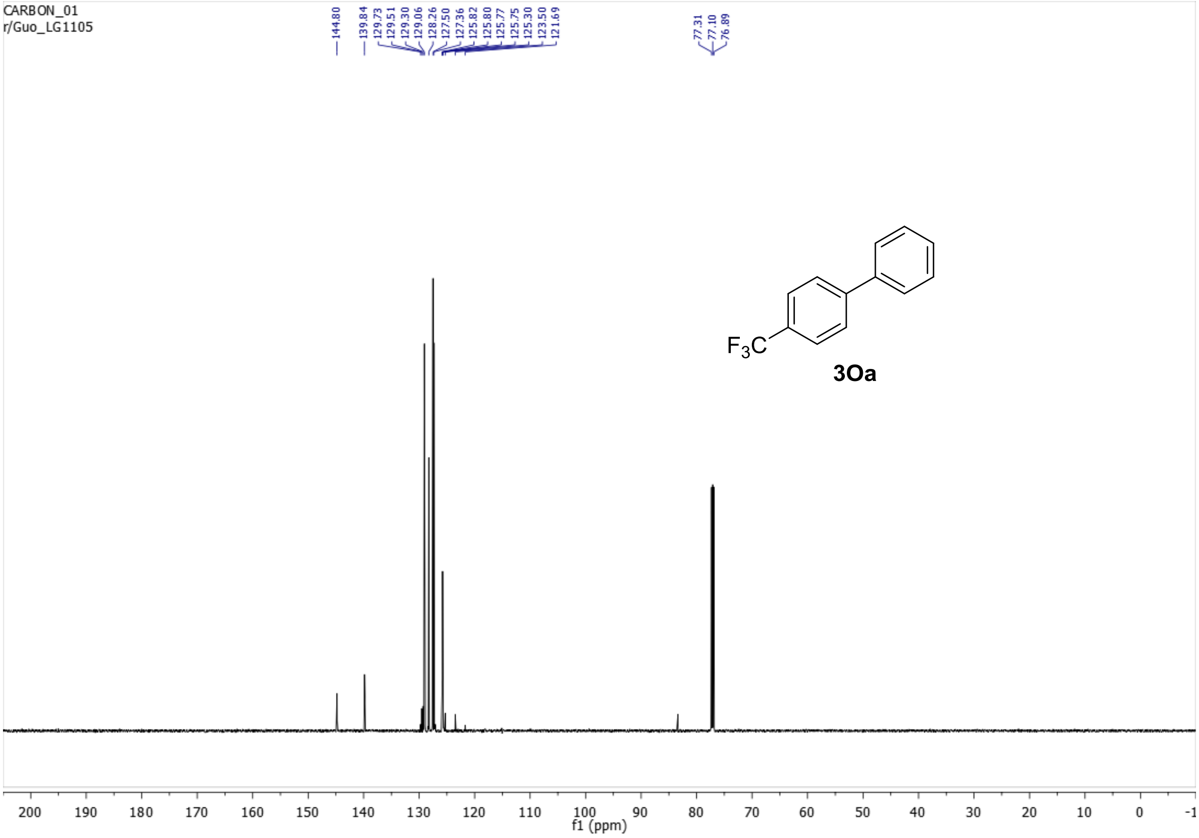

Supplementary Figure 119. ^{13}C NMR spectrum in $CDCl_3$ of compound **3Oa**.

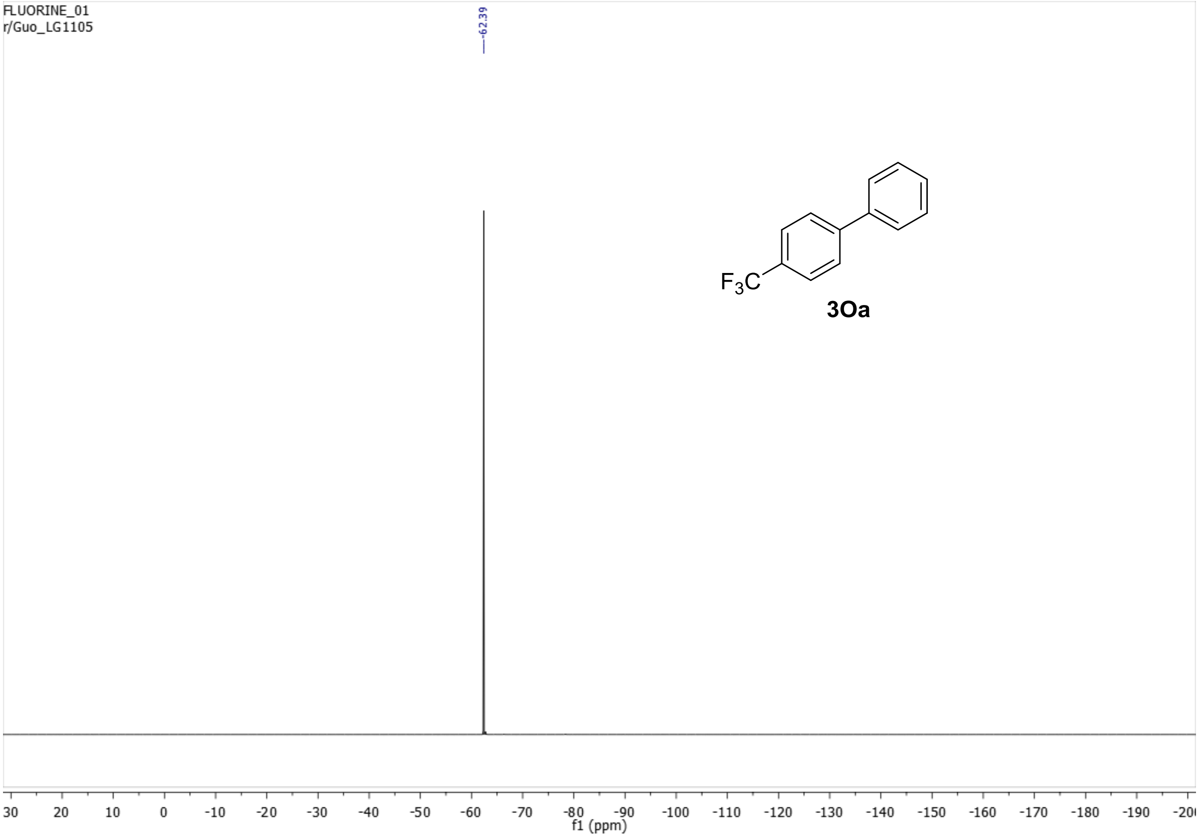

Supplementary Figure 120. ^{19}F NMR spectrum in $CDCl_3$ of compound **3Oa**.

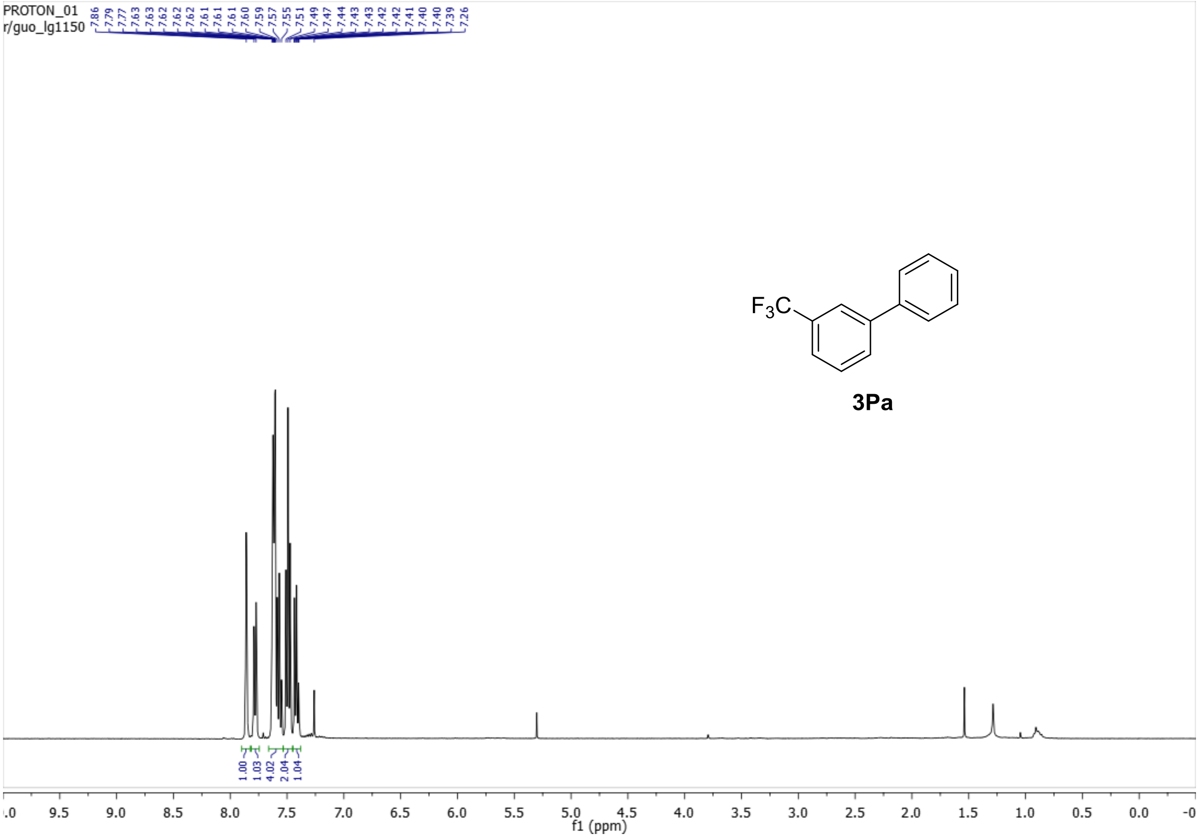

Supplementary Figure 121. ^{1}H NMR spectrum in $CDCl_3$ of compound **3Pa**.

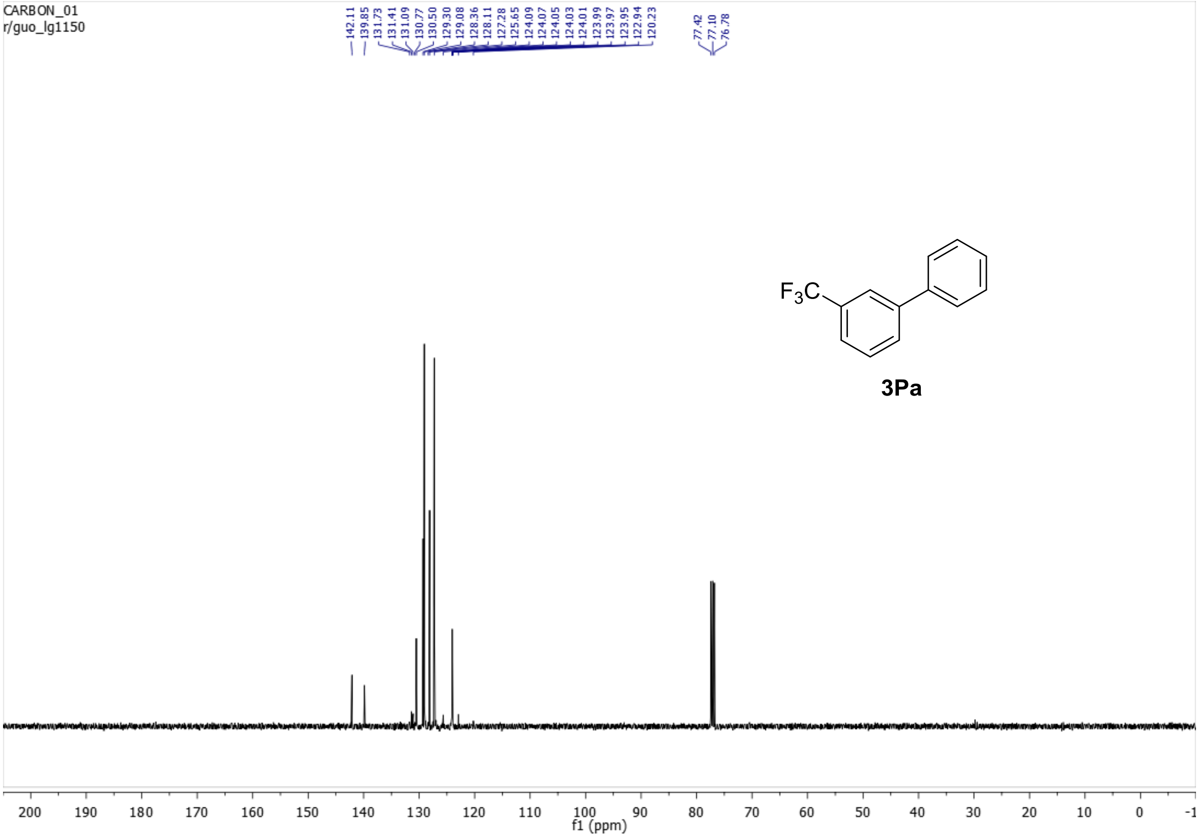

Supplementary Figure 122. ^{13}C NMR spectrum in $CDCl_3$ of compound **3Pa**.

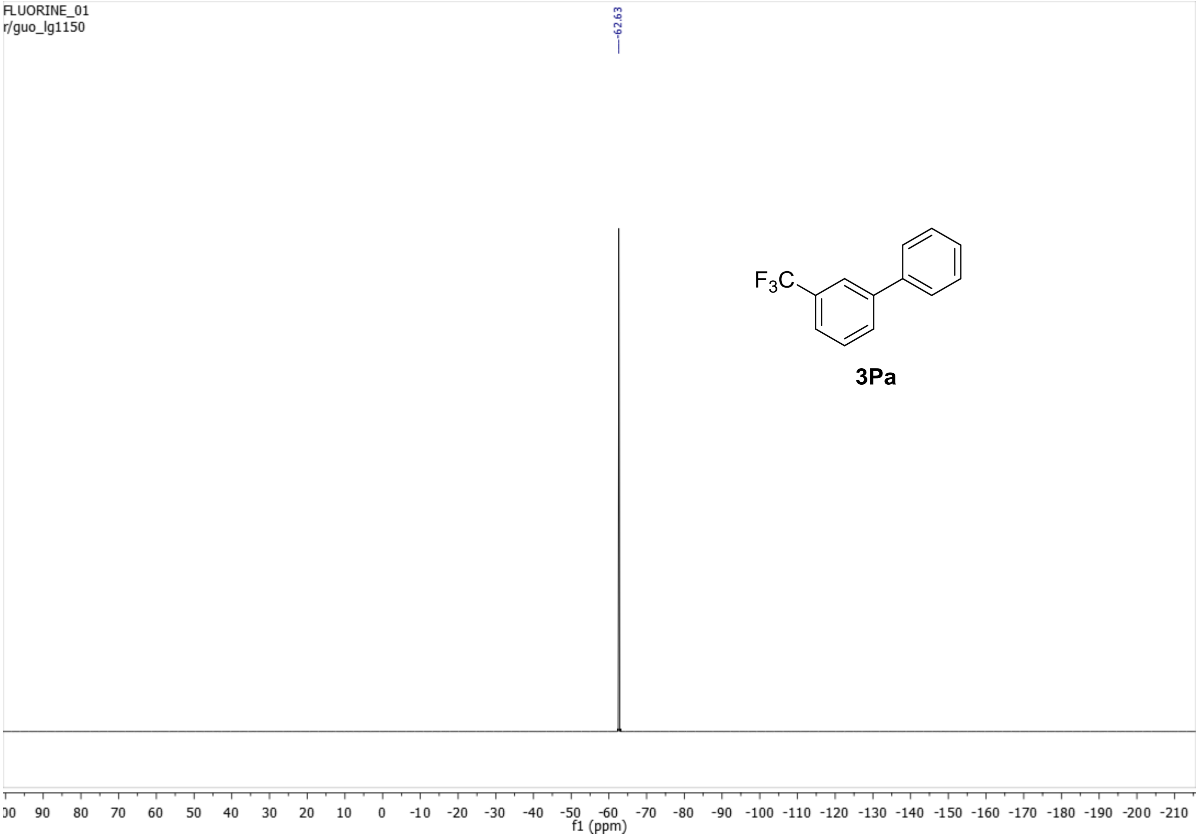

Supplementary Figure 123. ^{19}F NMR spectrum in $CDCl_3$ of compound **3Pa**.

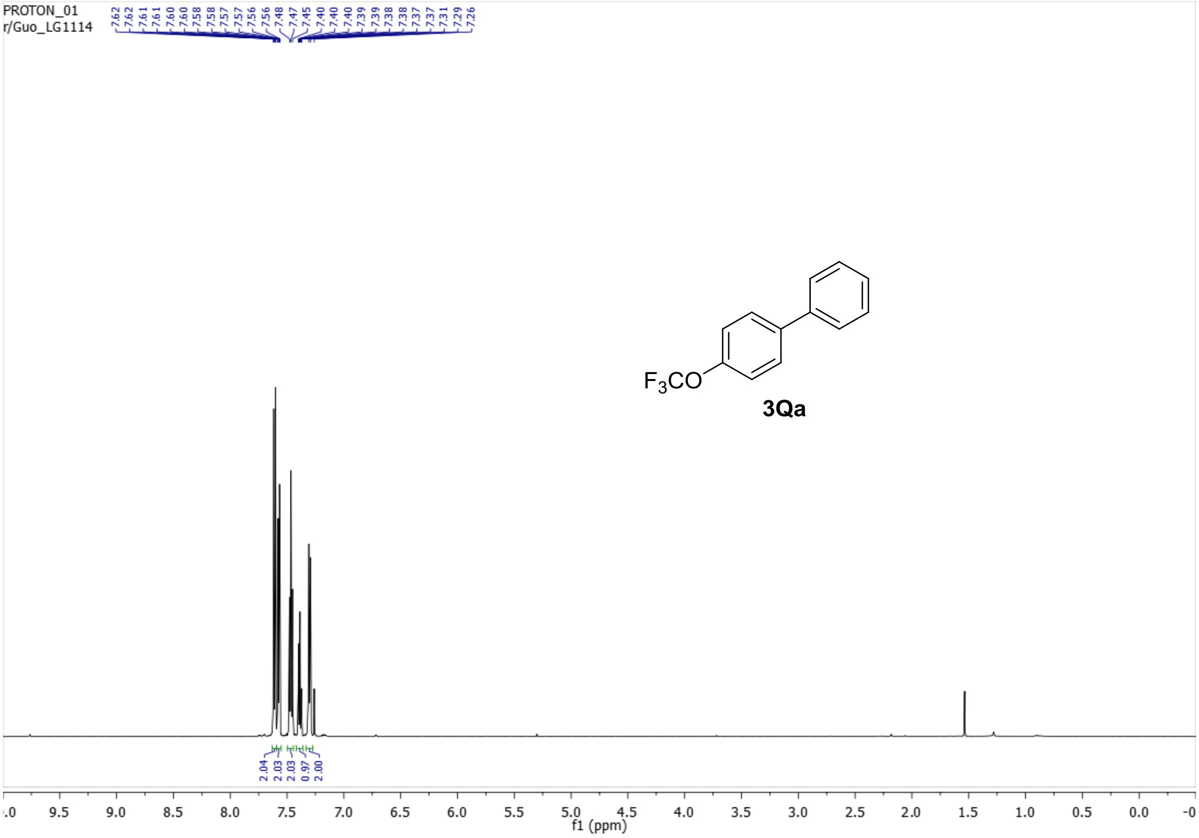

Supplementary Figure 124. ^{1}H NMR spectrum in $CDCl_3$ of compound **3Qa**.

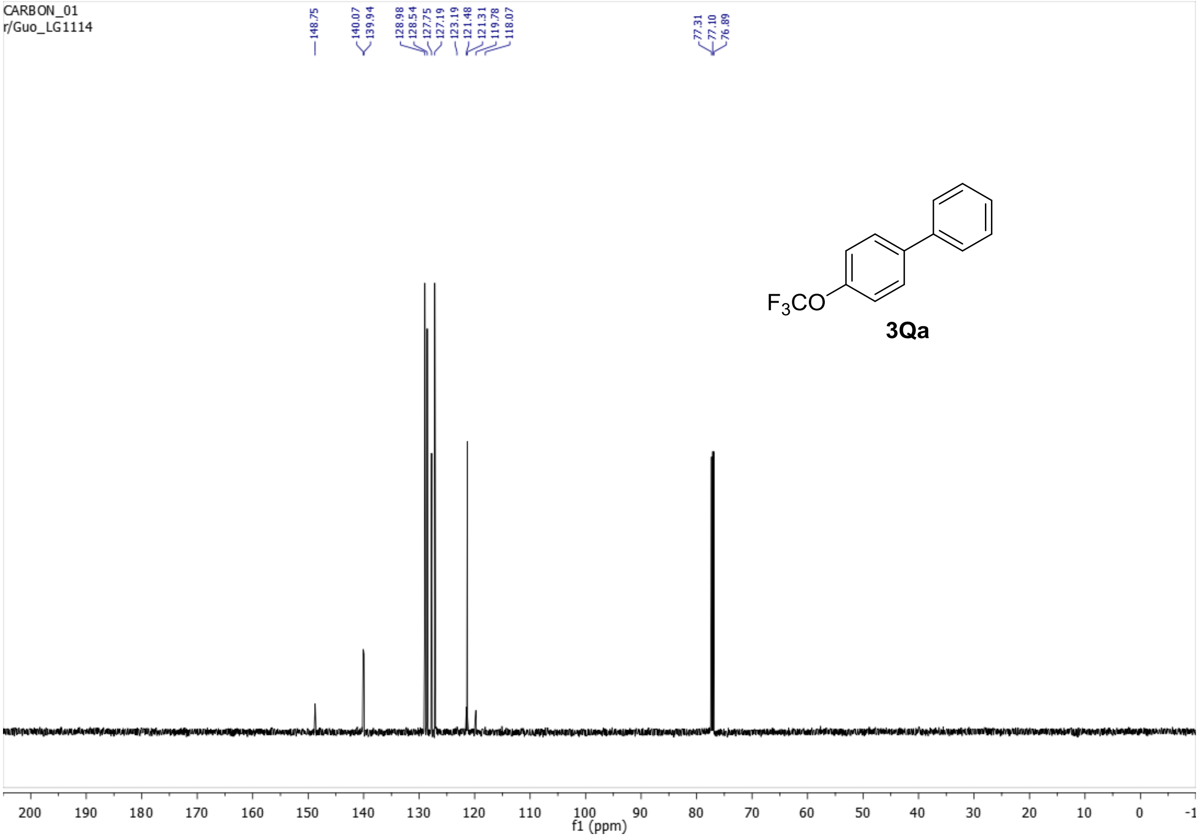

Supplementary Figure 125. ^{13}C NMR spectrum in $CDCl_3$ of compound **3Qa**.

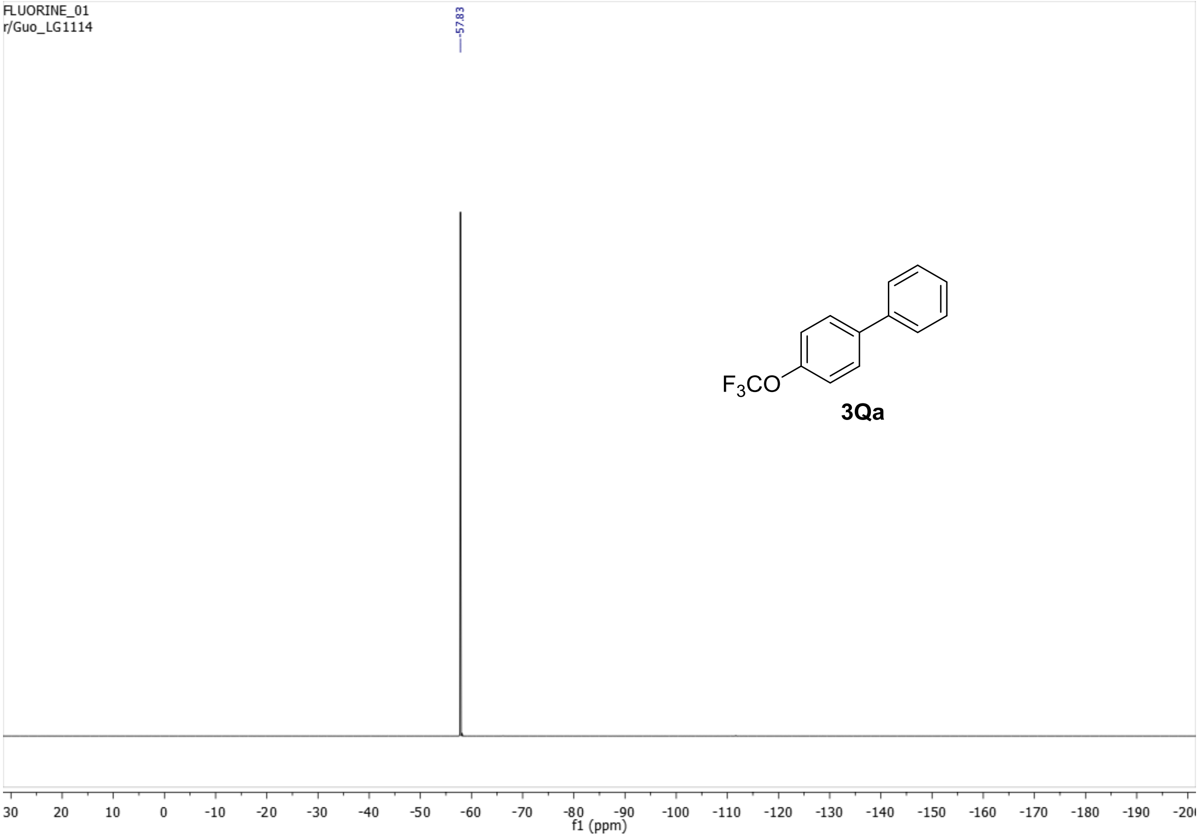

Supplementary Figure 126. ^{19}F NMR spectrum in $CDCl_3$ of compound **3Qr**.

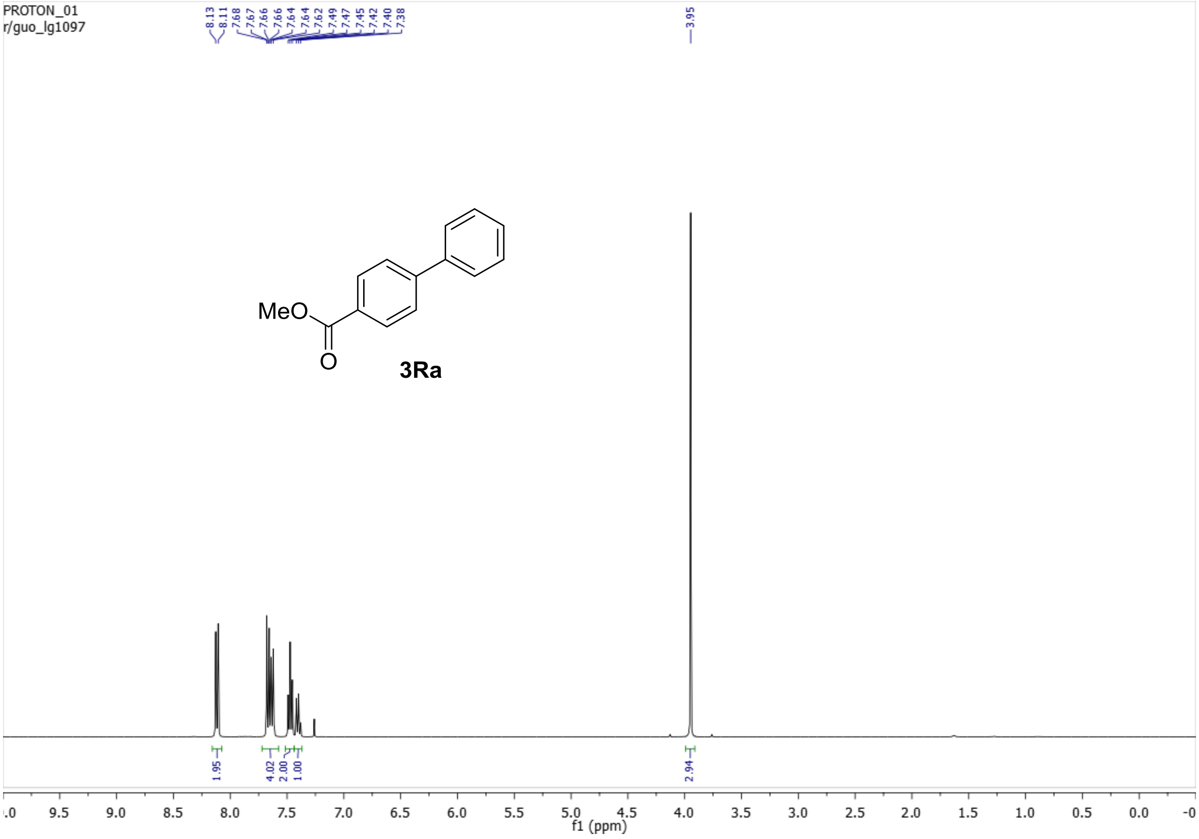

Supplementary Figure 127. ^{1}H NMR spectrum in $CDCl_3$ of compound **3Ra**.

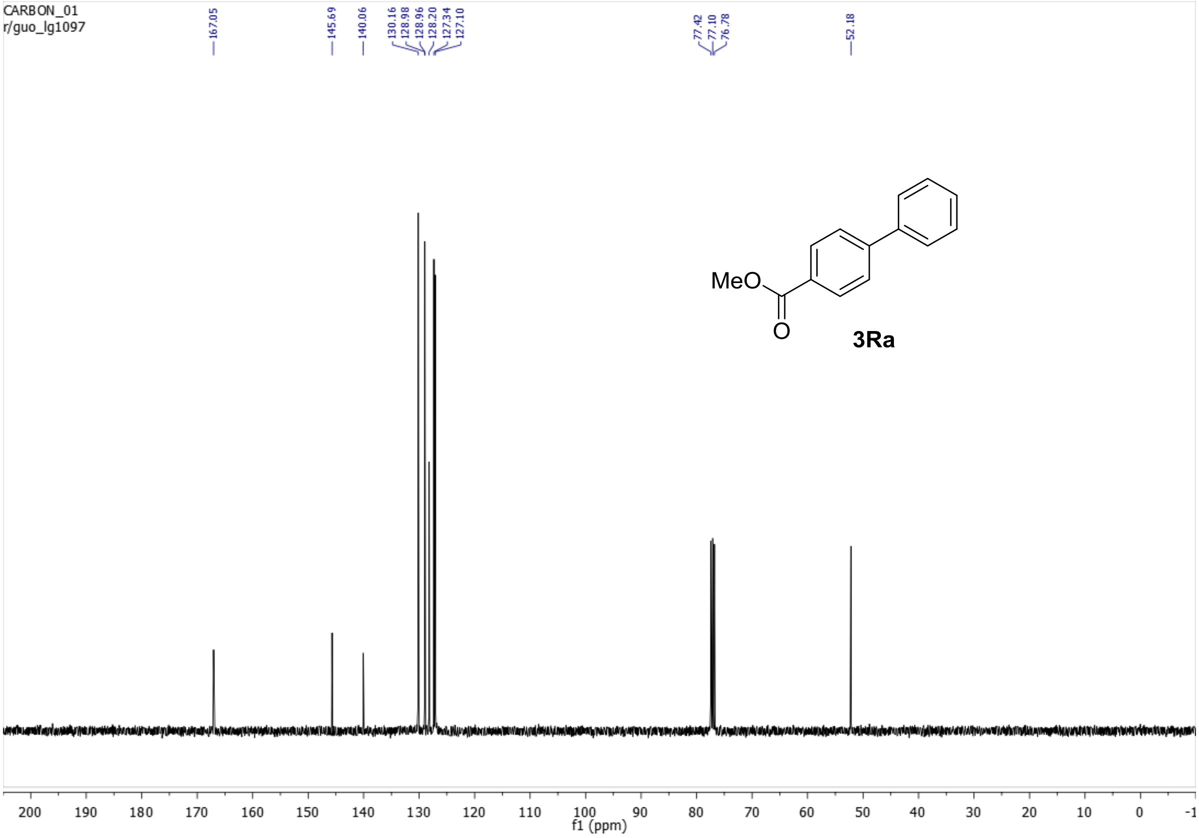

Supplementary Figure 128. ^{13}C NMR spectrum in $CDCl_3$ of compound **3Ra**.

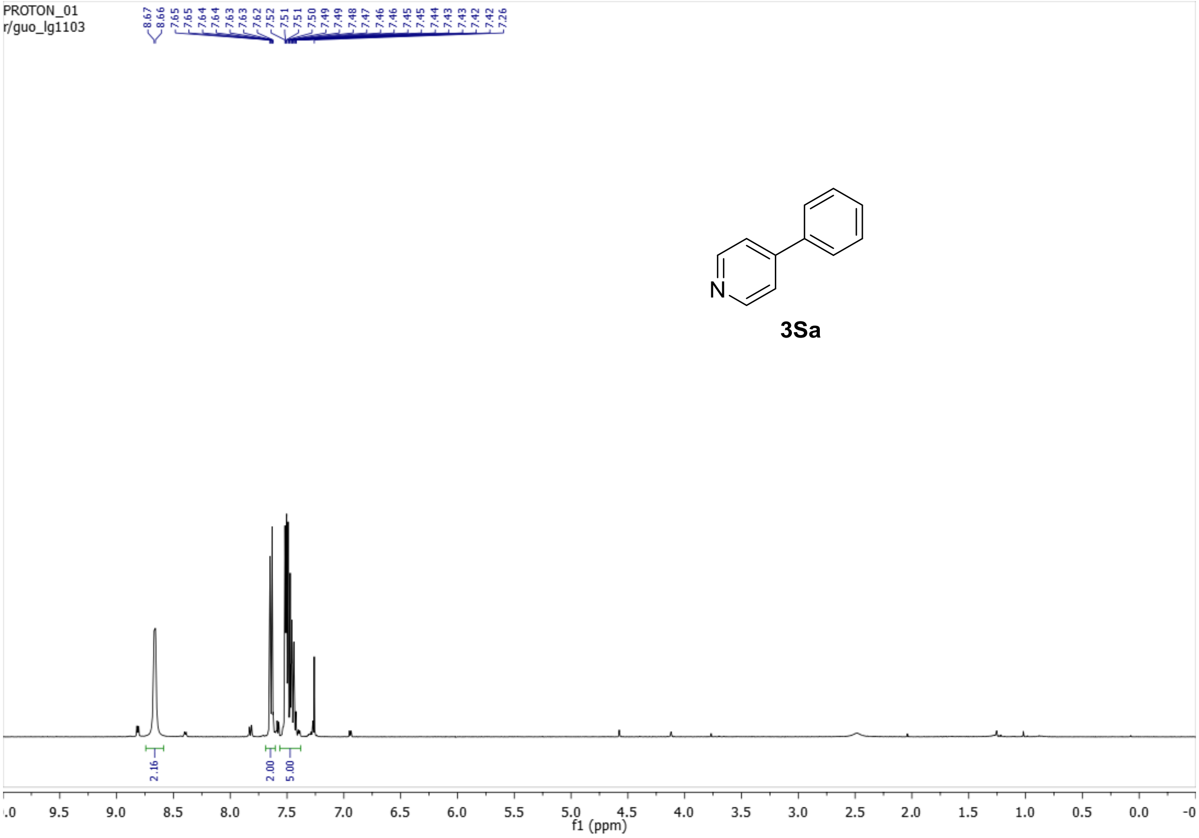

Supplementary Figure 129. ^{1}H NMR spectrum in $CDCl_3$ of compound **3Sa**.

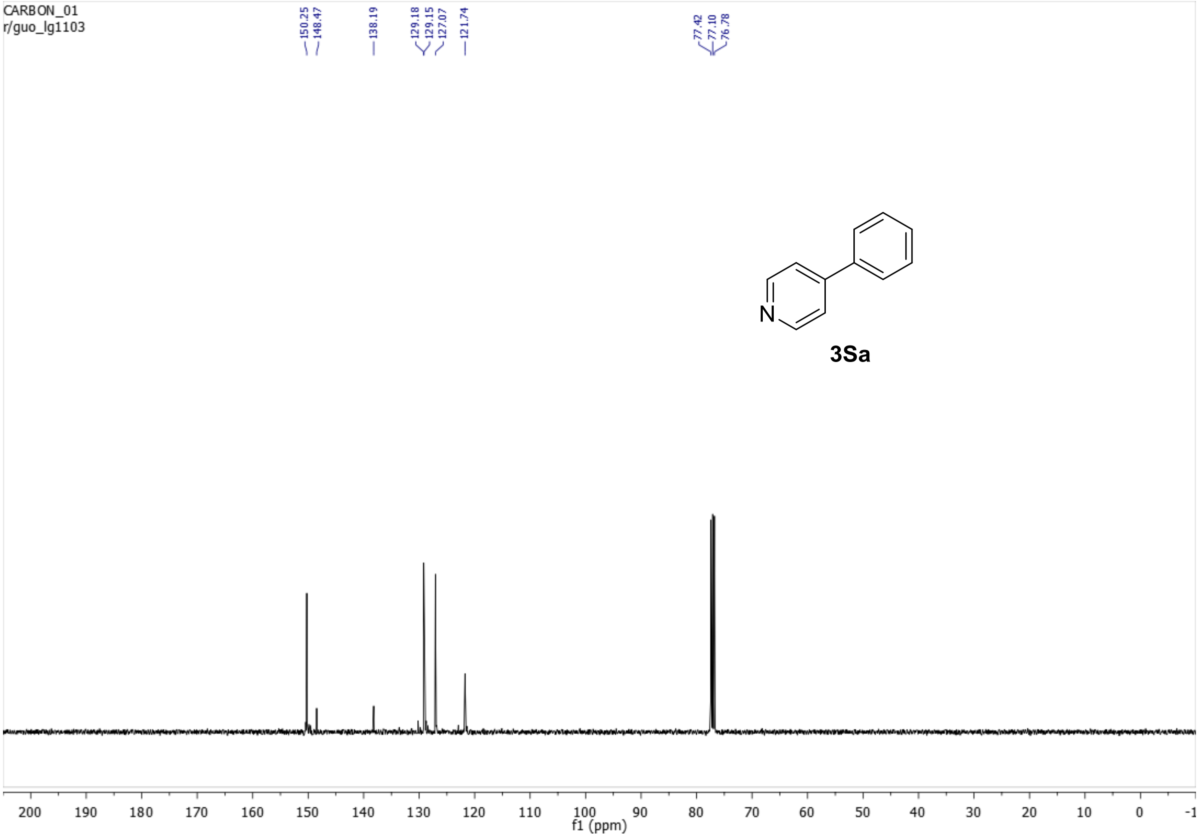

Supplementary Figure 130. ^{13}C NMR spectrum in $CDCl_3$ of compound **3Sa**.

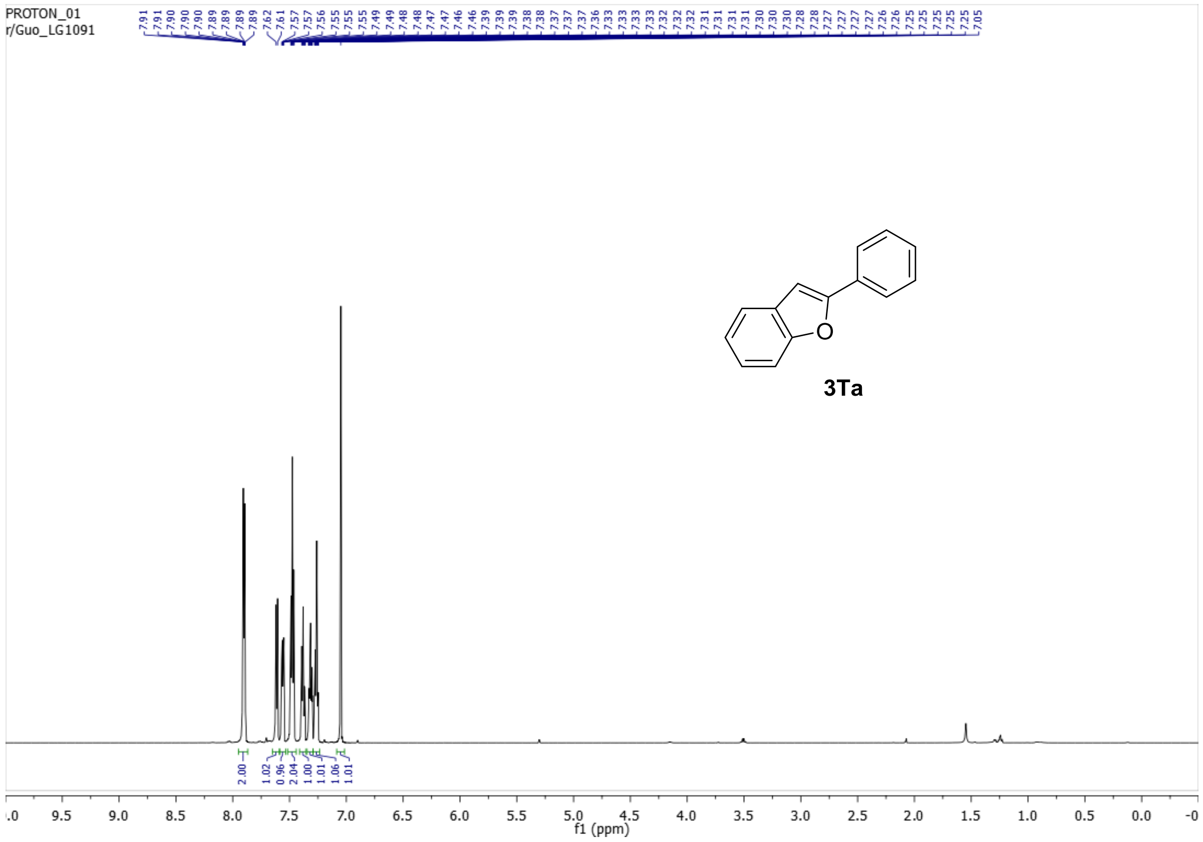

Supplementary Figure 131. ^{1}H NMR spectrum in $CDCl_3$ of compound **3Ta**.

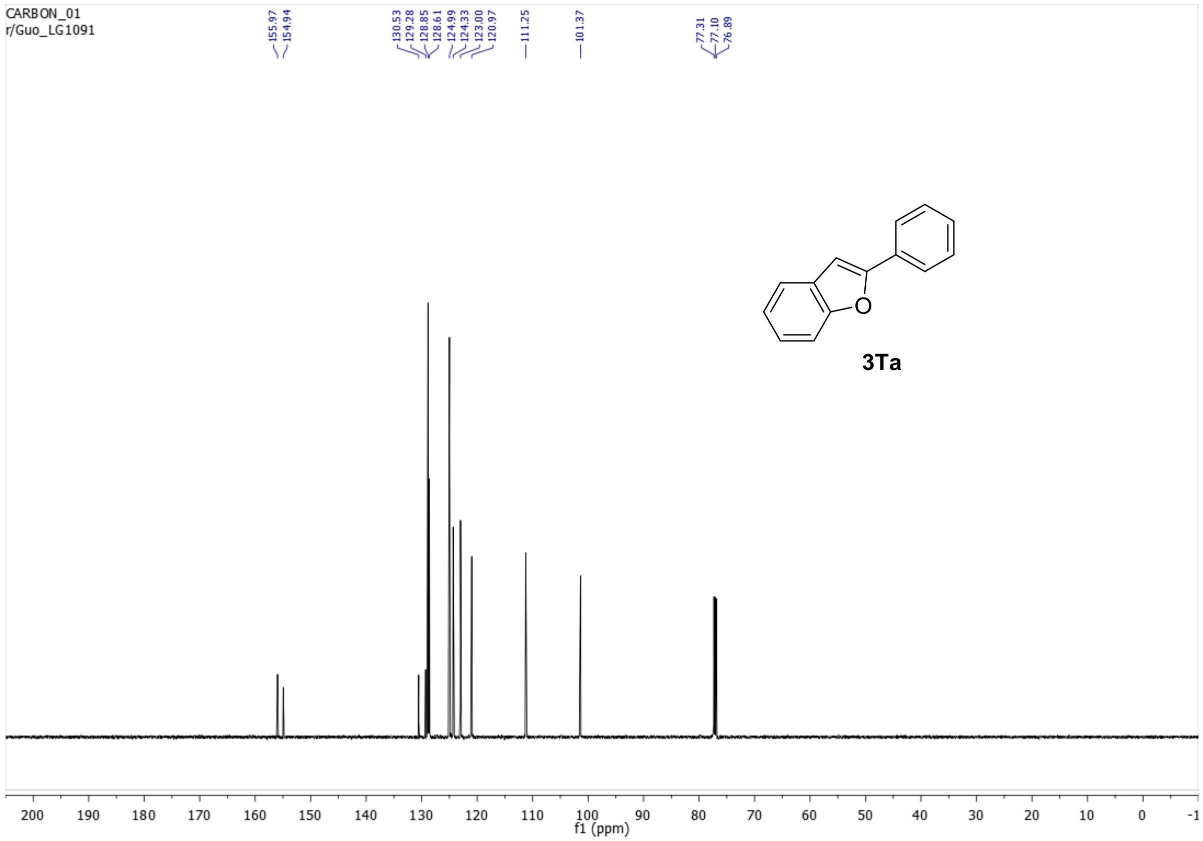

Supplementary Figure 132. ^{13}C NMR spectrum in $CDCl_3$ of compound **3Ta**.

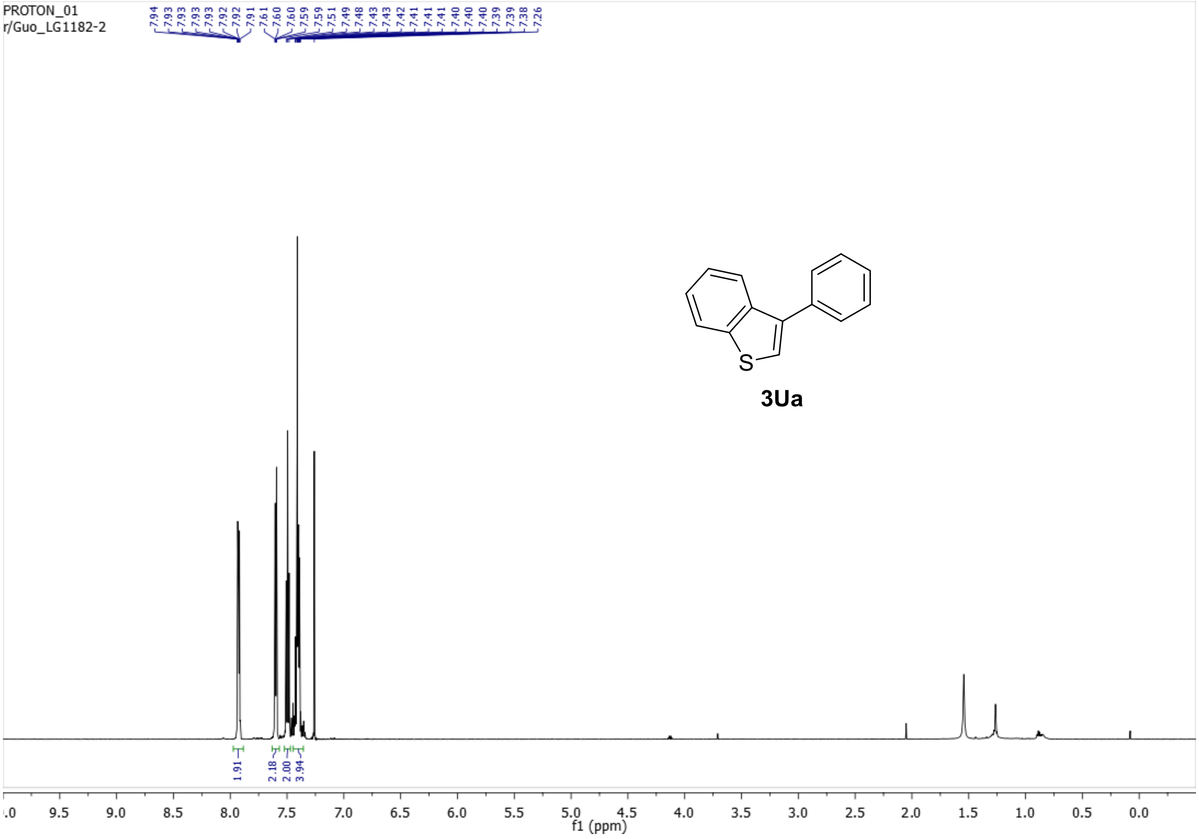

Supplementary Figure 133. ^{1}H NMR spectrum in $CDCl_3$ of compound **3Ua**.

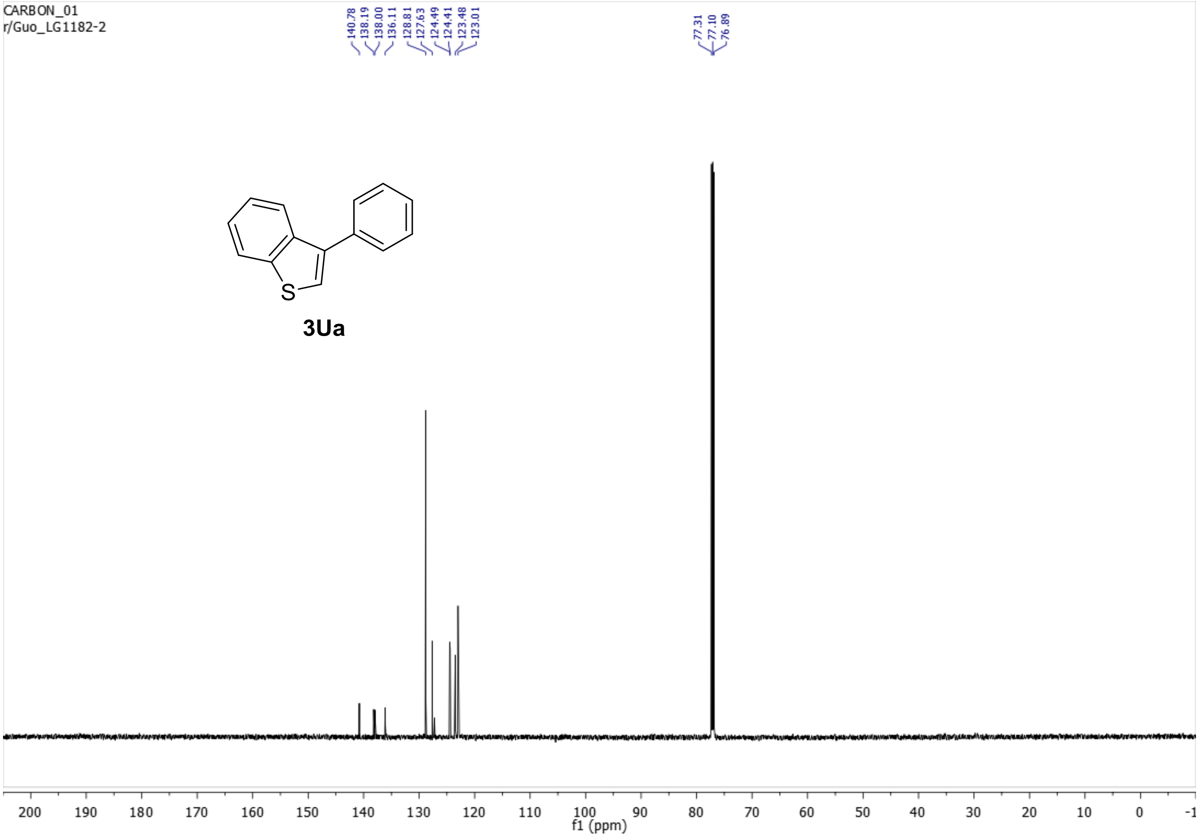

Supplementary Figure 134. ^{13}C NMR spectrum in $CDCl_3$ of compound **3Ua**.

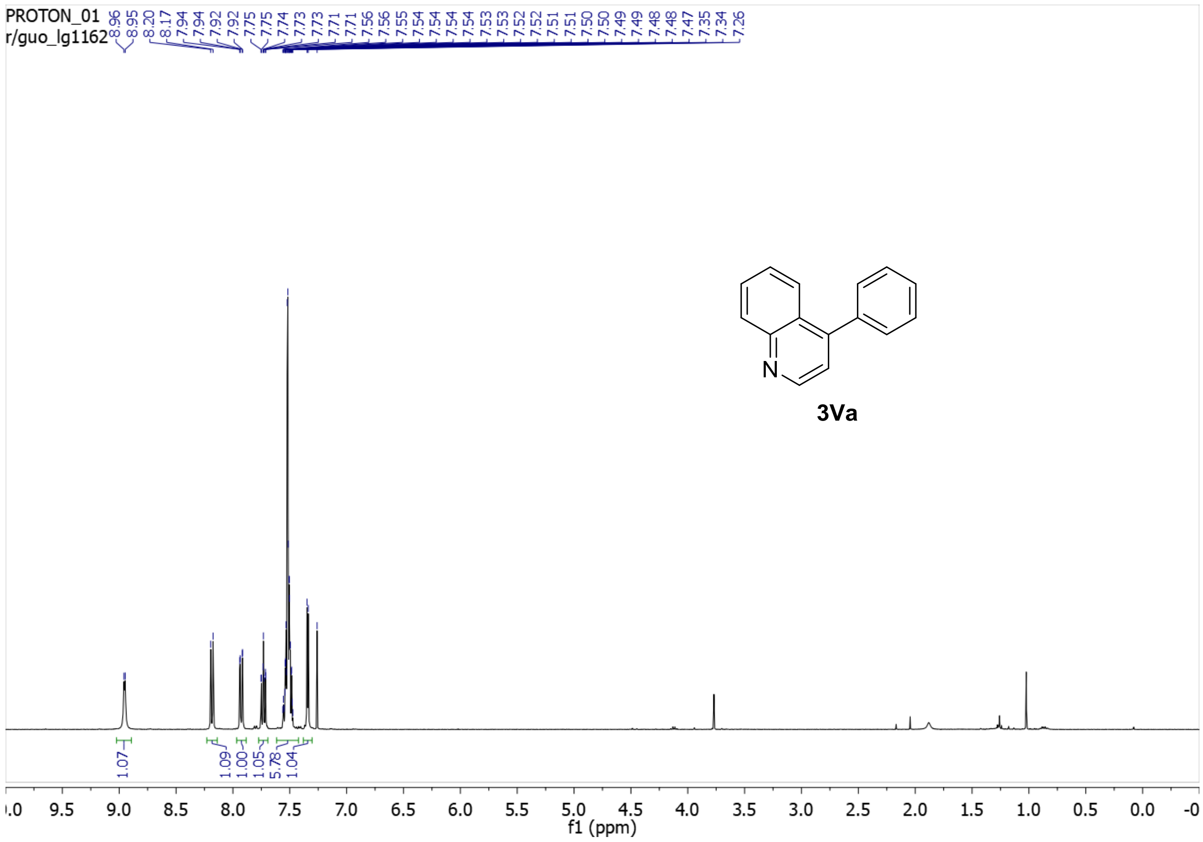

Supplementary Figure 135. ^{1}H NMR spectrum in $CDCl_3$ of compound **3Va**.

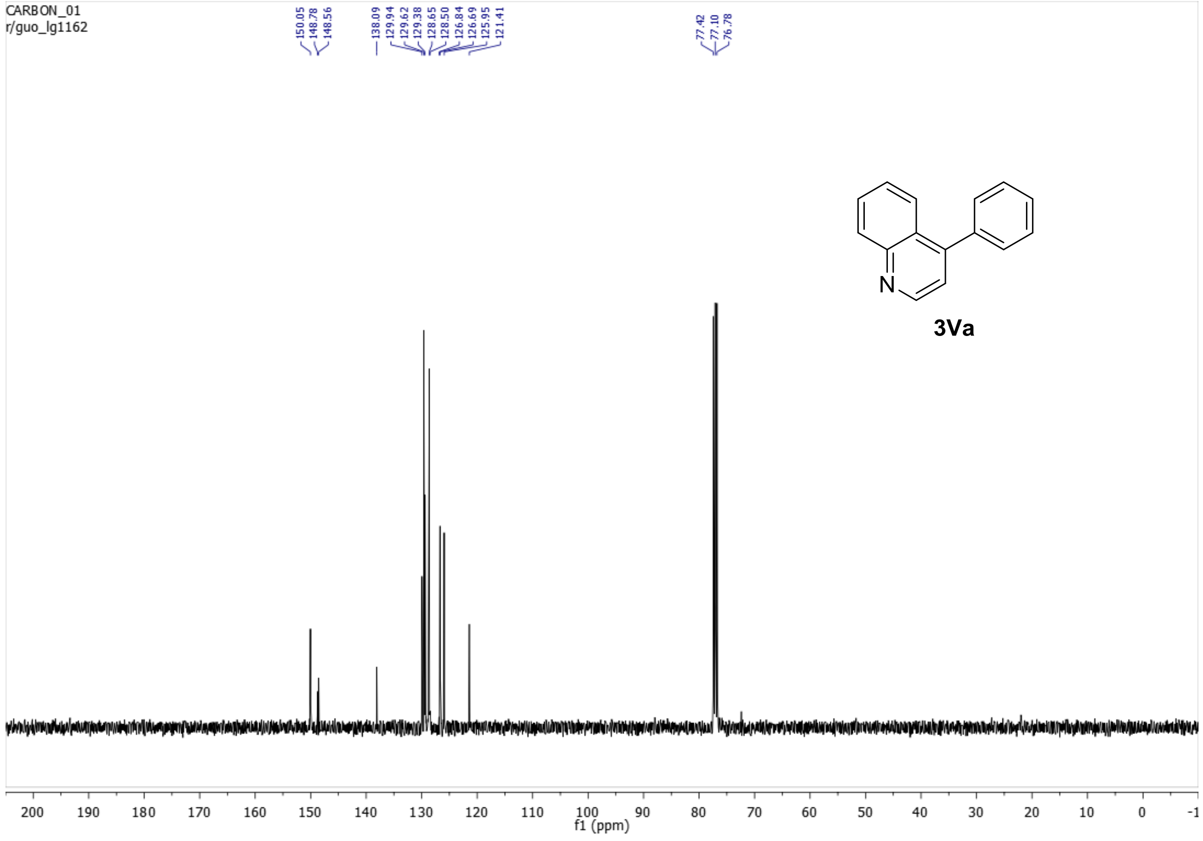

Supplementary Figure 136. ^{13}C NMR spectrum in $CDCl_3$ of compound **3Va**.

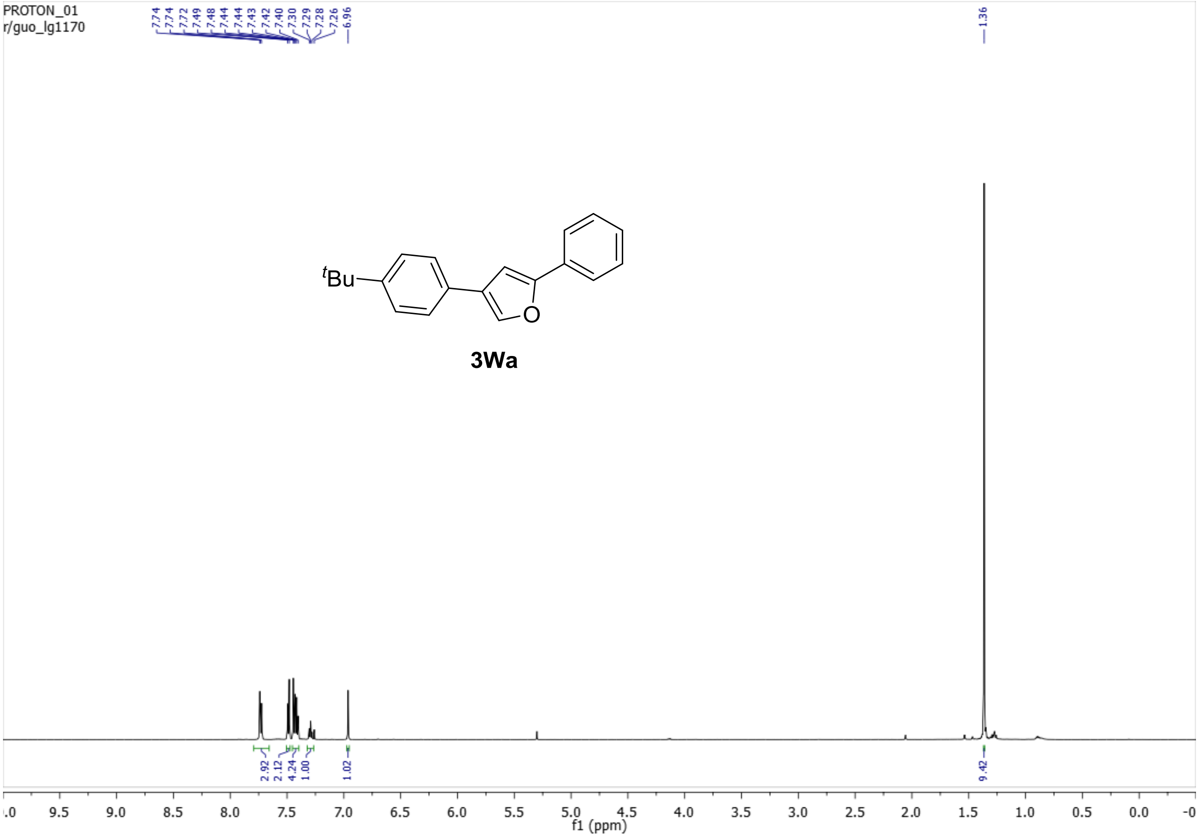

Supplementary Figure 137. ^{1}H NMR spectrum in $CDCl_3$ of compound **3Wa**.

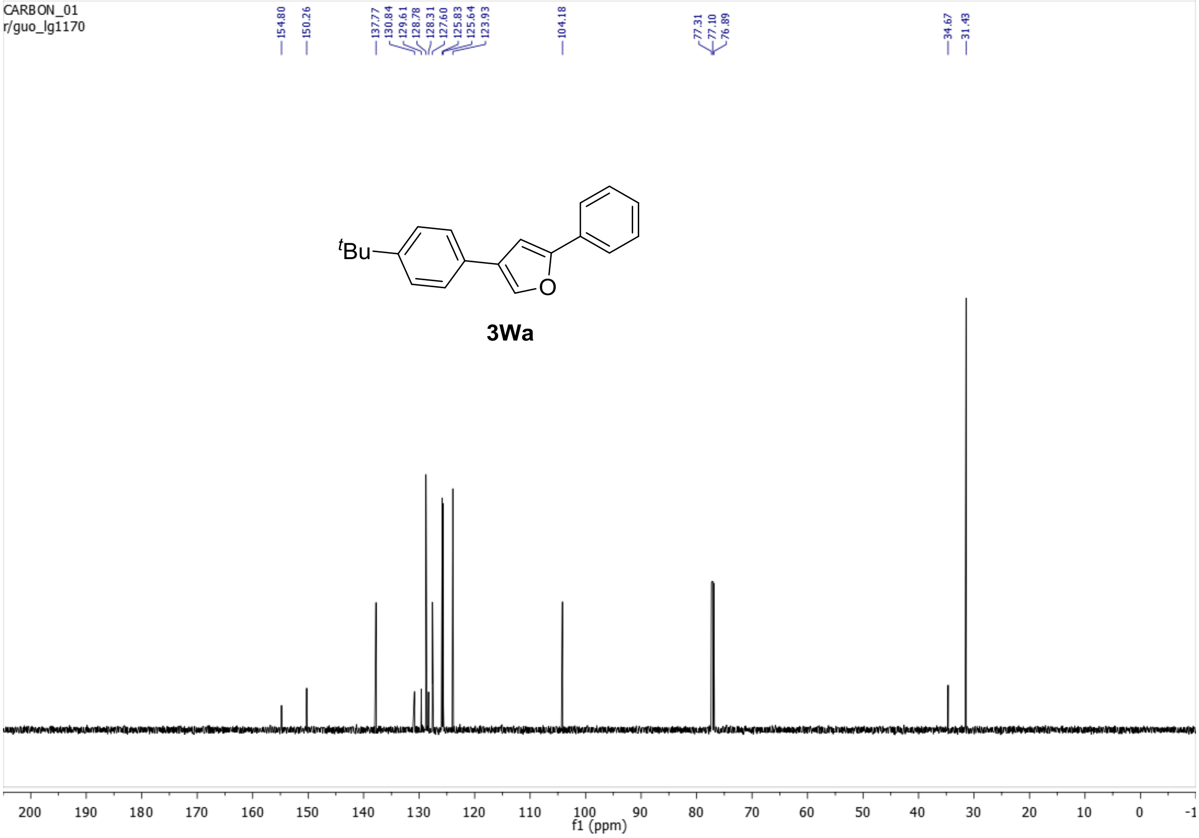

Supplementary Figure 138. ^{13}C NMR spectrum in $CDCl_3$ of compound **3Wa**.

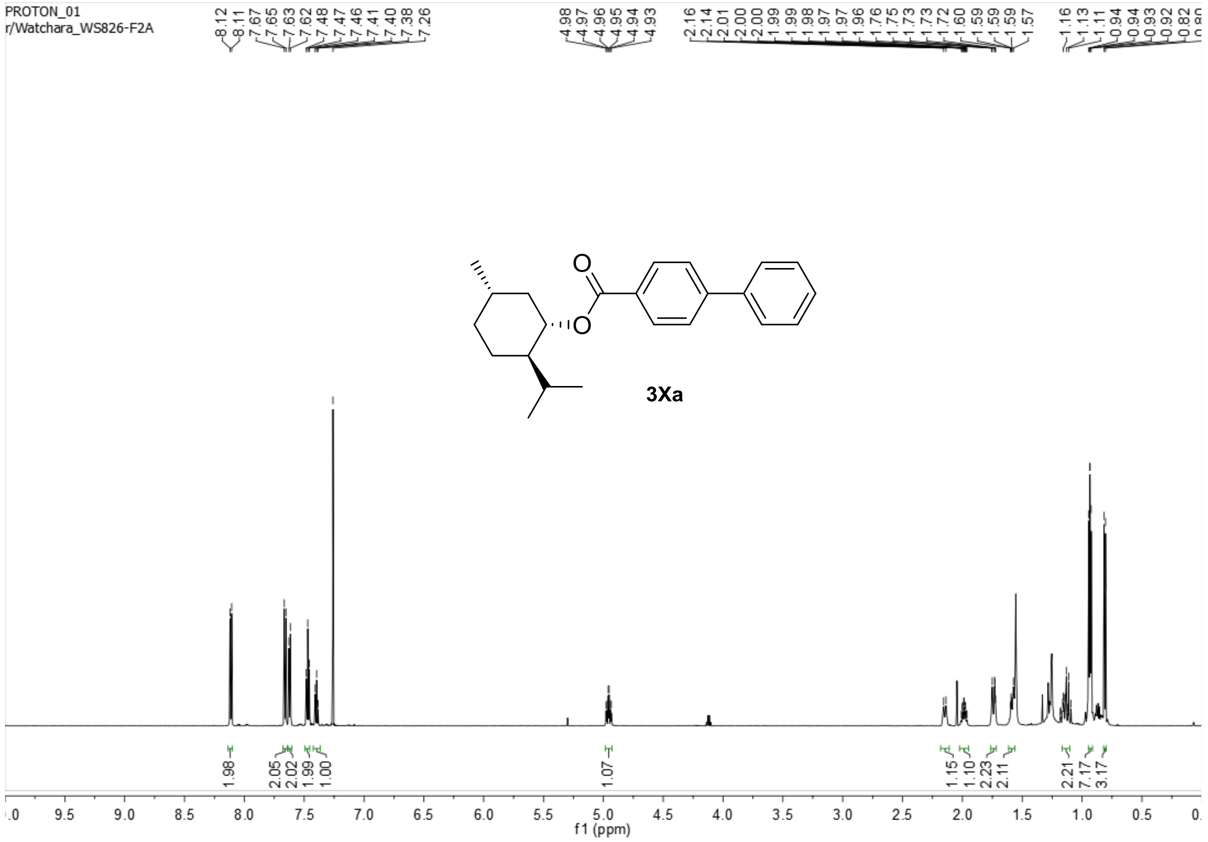

Supplementary Figure 139. ^{1}H NMR spectrum in $CDCl_3$ of compound **3Xa**.

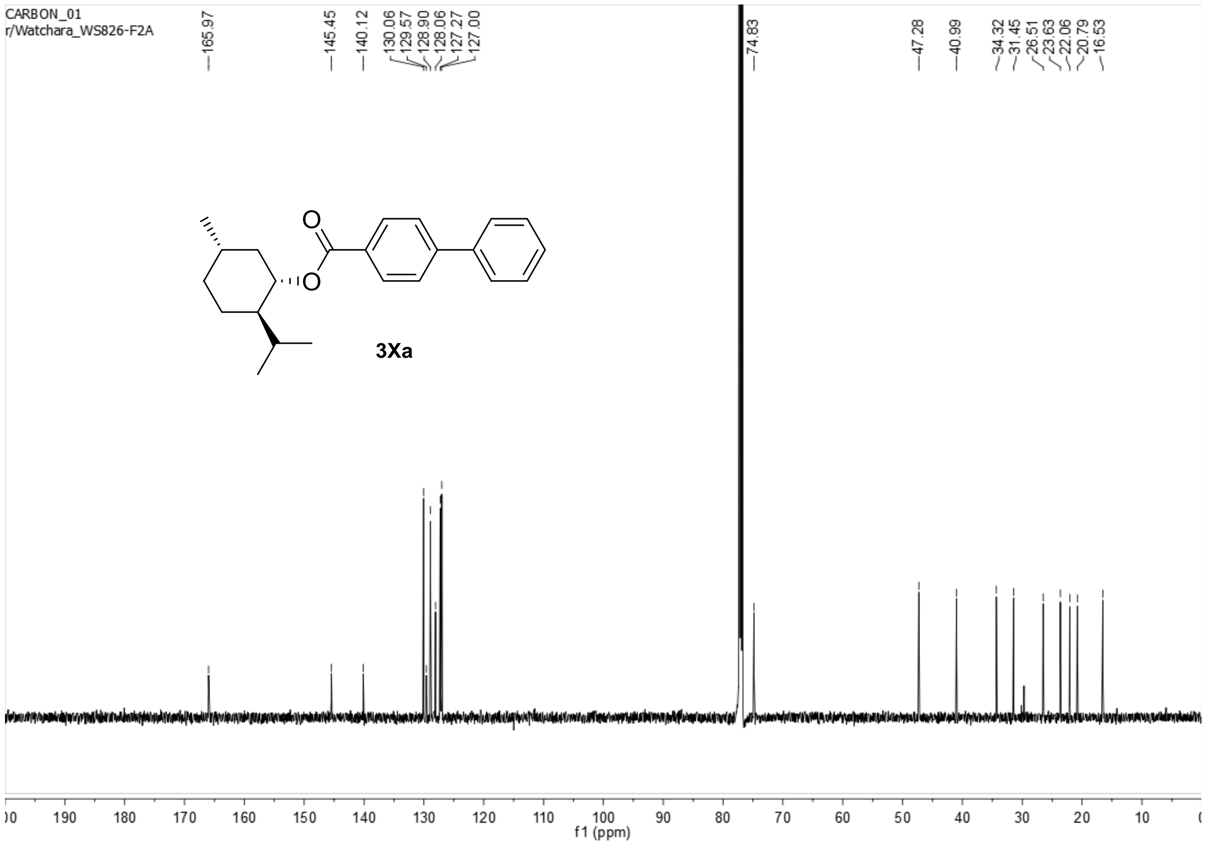

Supplementary Figure 140. ^{13}C NMR spectrum in $CDCl_3$ of compound **3Xa**.

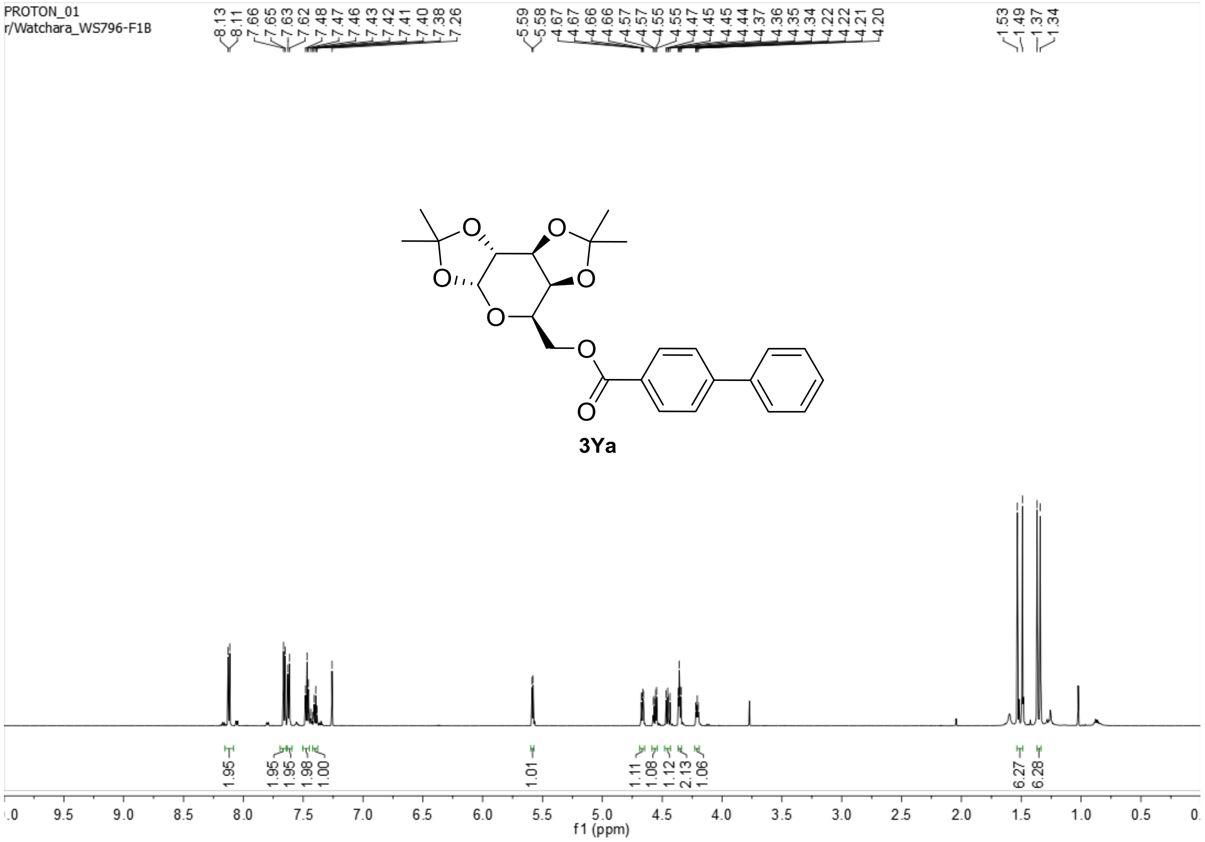

Supplementary Figure 141. ^{1}H NMR spectrum in $CDCl_3$ of compound **3Ya**.

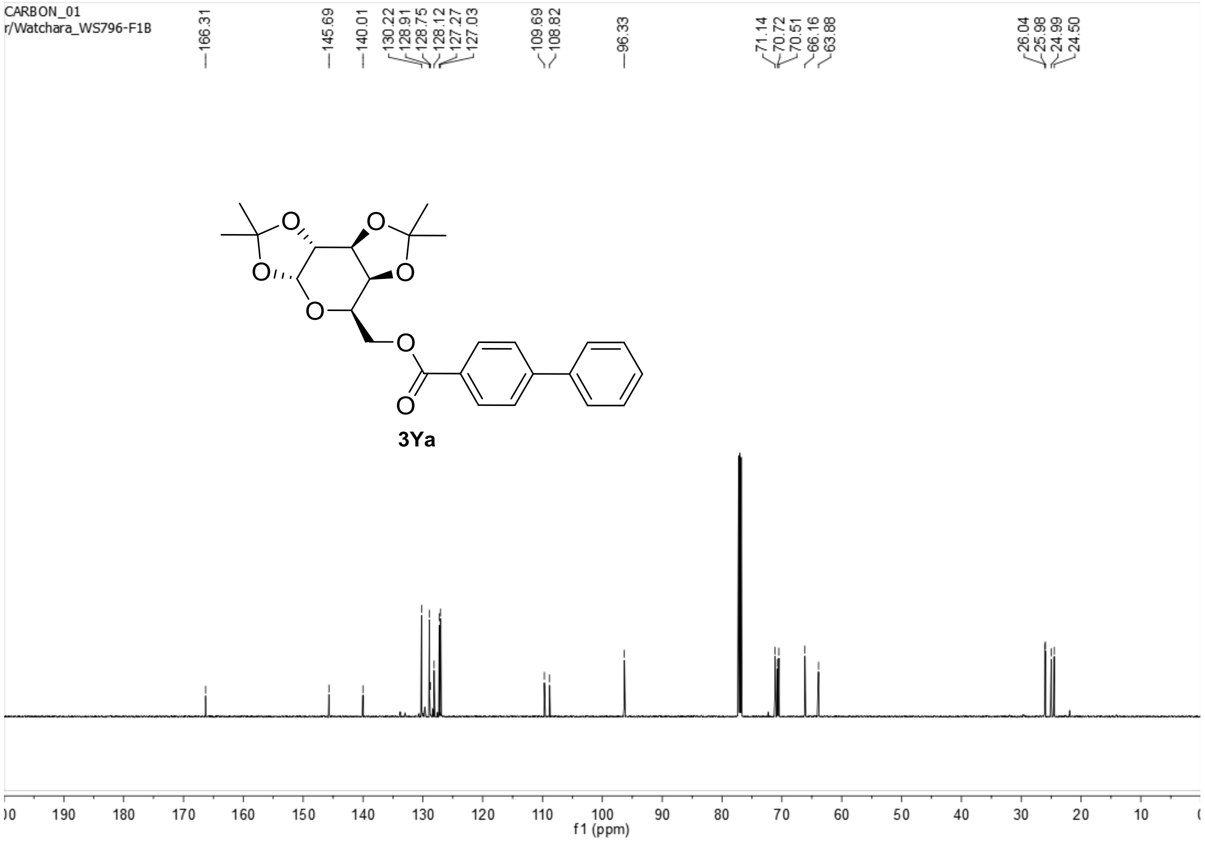

Supplementary Figure 142. ^{13}C NMR spectrum in $CDCl_3$ of compound **3Ya**.

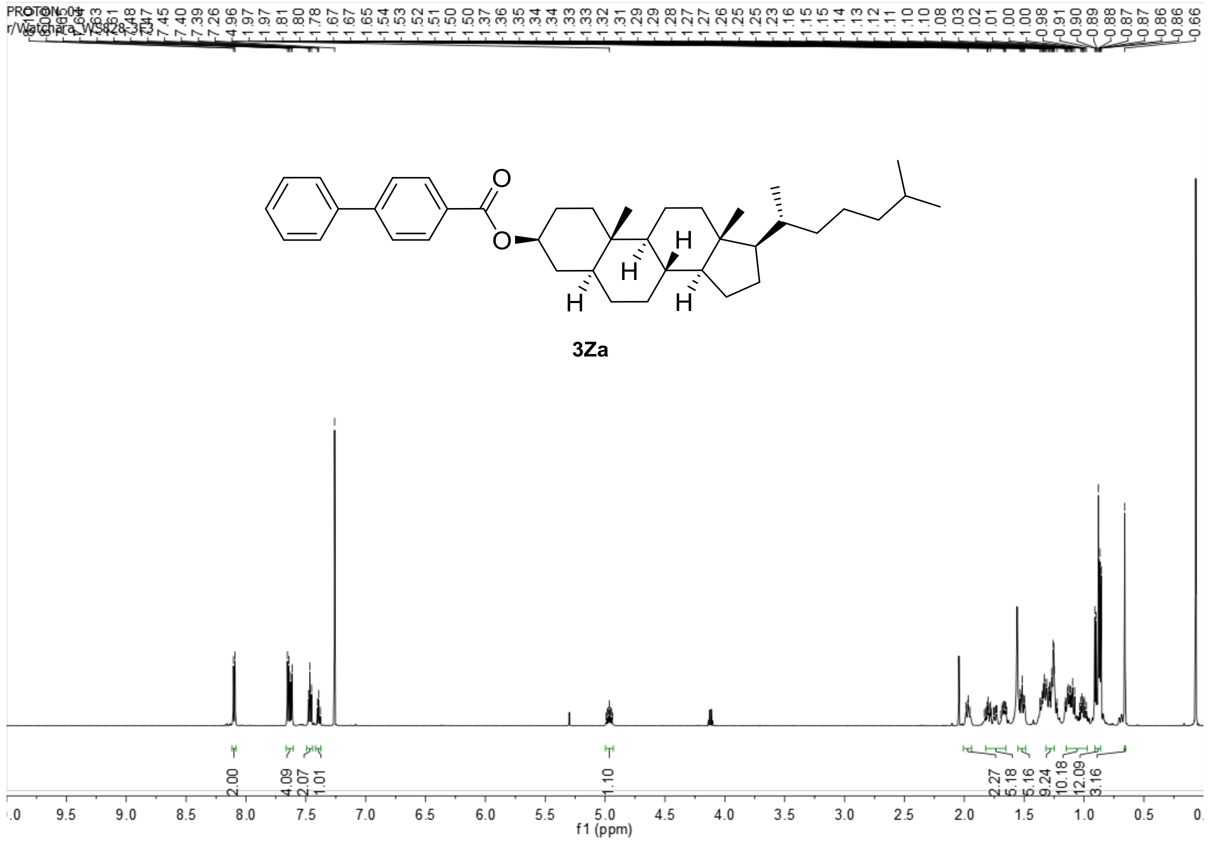

Supplementary Figure 143. ^{1}H NMR spectrum in $CDCl_3$ of compound **3Za**.

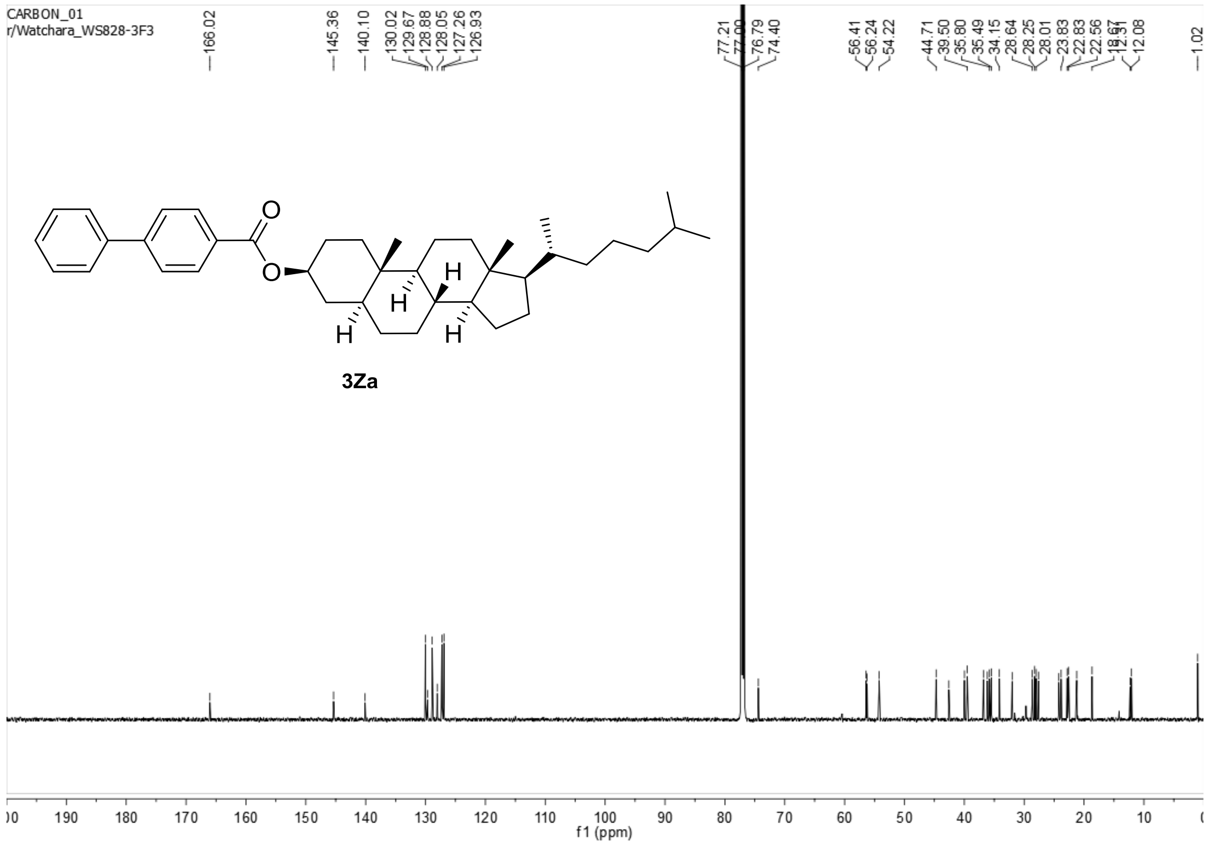

Supplementary Figure 144. ^{13}C NMR spectrum in $CDCl_3$ of compound **3Za**.

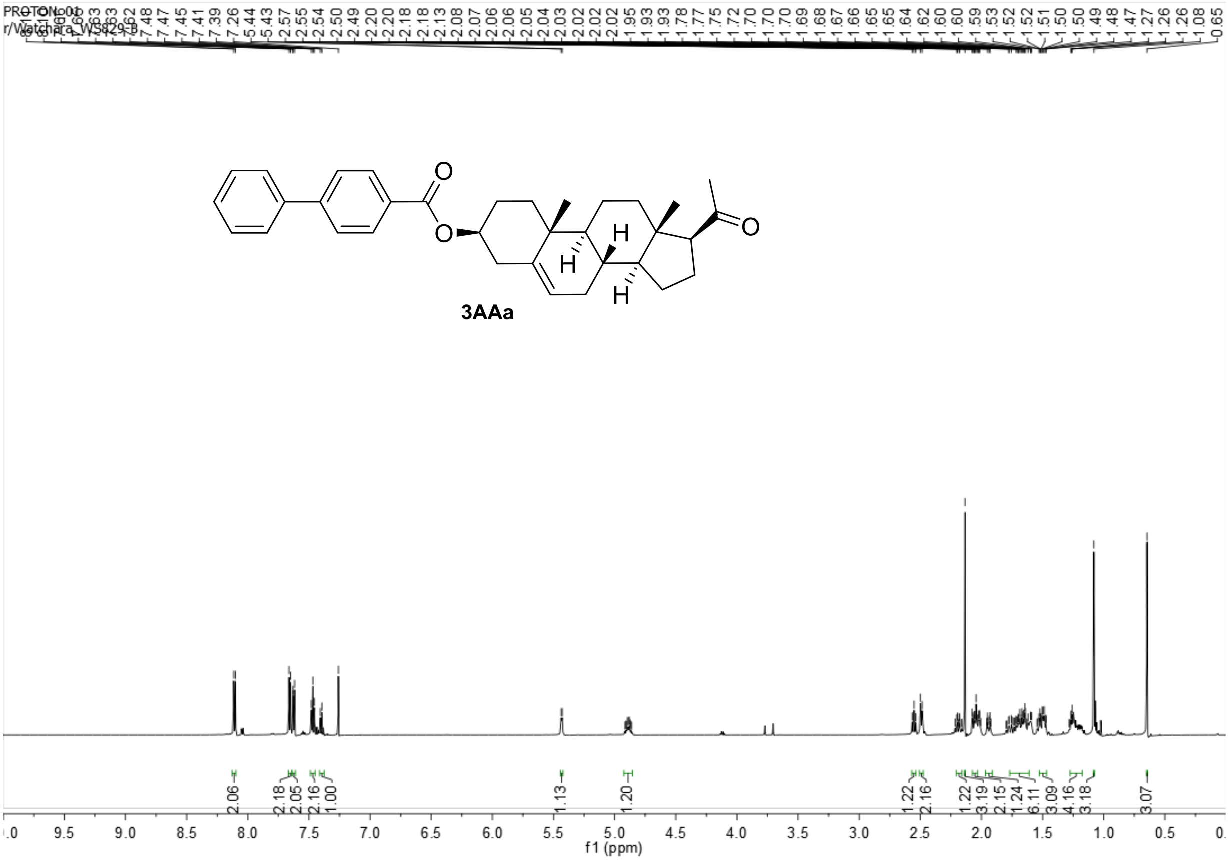

Supplementary Figure 145. ^{1}H NMR spectrum in $CDCl_3$ of compound **3AAa**.

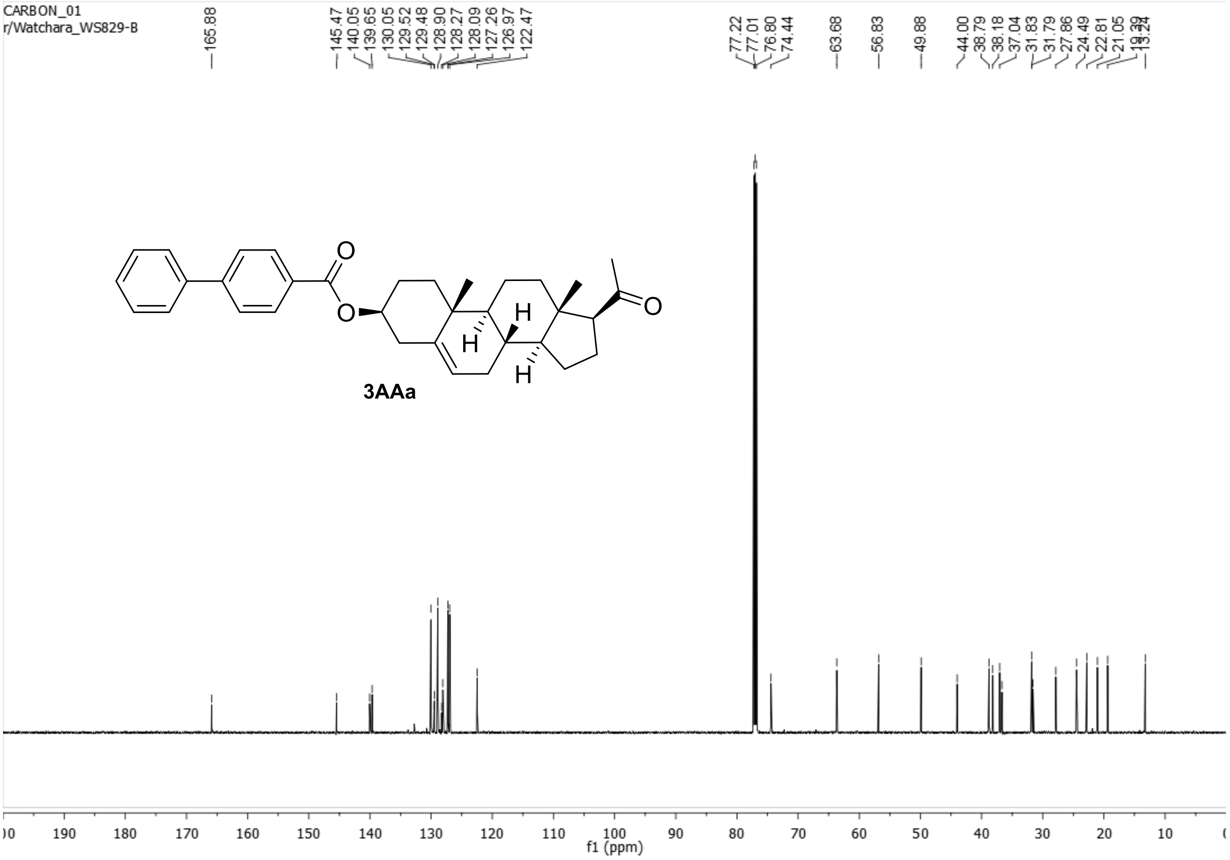

Supplementary Figure 146. ^{13}C NMR spectrum in $CDCl_3$ of compound **3AAa**.

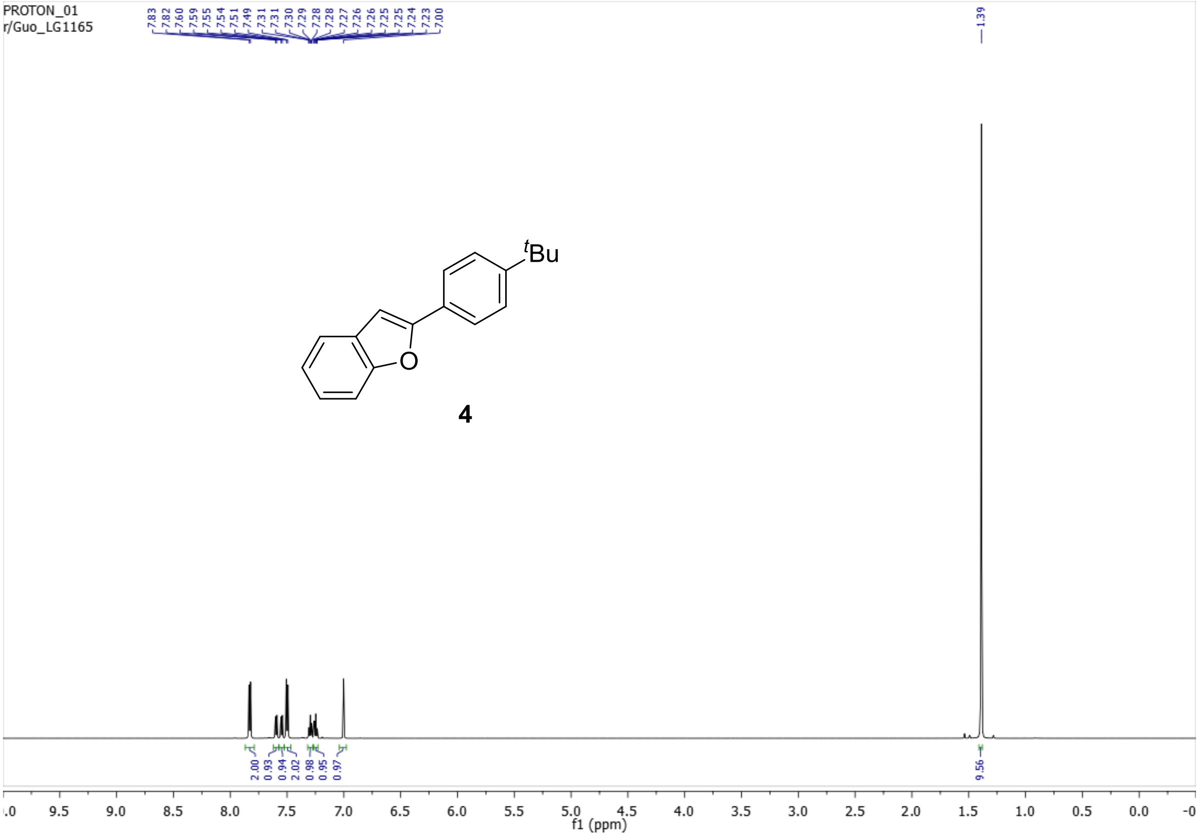

Supplementary Figure 147. ^{1}H NMR spectrum in $CDCl_3$ of compound **4**.

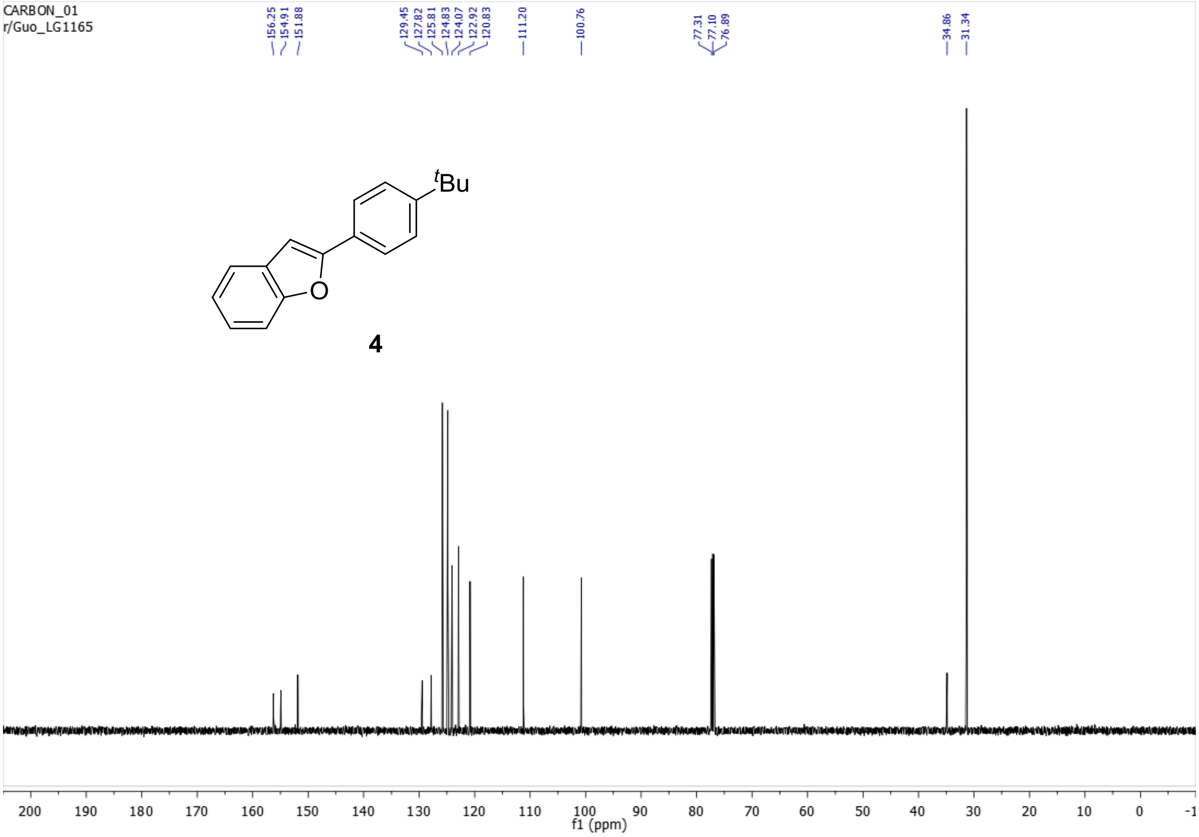

Supplementary Figure 148. ^{13}C NMR spectrum in $CDCl_3$ of compound **4**.

Supplementary References

1. Pearson, M. S. M. & Carbery, D. R. Studies toward the Photochemical Synthesis of Functionalized [5]- and [6]Carbohelicenes. *J. Org. Chem*. **74**, 5320–5325 (2009).
2. Li, G.-L., Kung, K. K.-Y. & Wong, M.-K. Gold-catalyzed amide synthesis from aldehydes and amines in aqueous medium. *Chem. Commun.* **48**, 4112–4114 (2012).
3. Barsamian, A. L., Wu, Z. & Blakemore, P. R. Enantioselective synthesis of α-phenyl- and α-(dimethylphenylsilyl)alkylboronic esters by ligand mediated stereoinductive reagent-controlled homologation using configurationally labile carbenoids. *Org. Biomol. Chem.* **13**, 3781–3786 (2015).
4. Bello, C. S. & Schmidt-Leithoff, J. Borylation of organo halides and triflates using tetrakis(dimethylamino)diboron. *Tetrahedron Lett.* **53**, 6230–6235 (2012).
5. Vinogradova, E. V., Park, N. H., Fors, B. P. & Buchwald, S. L. Palladium-Catalyzed Synthesis of *N*-Aryl Carbamates. *Org. Lett.* **15**, 1394–1397 (2013).
6. Dai, F., et al. Pd-catalyzed C3-selective arylation of pyridines with phenyl tosylates. *Chem. Commun.* **49**, 4634–4636 (2013).
7. LaBerge, N. A. & Love, J. A. Nickel-Catalyzed Decarbonylative Coupling of Aryl Esters and Arylboronic Acids. *Eur. J. Org. Chem.* 5546–5553 (2015).
8. Rao, M. L. N. & Dhanorkar, R. J. Triarylbismuthanes as Threefold Aryl-Transfer Reagents in Regioselective Cross-Coupling Reactions with Bromopyridines and Quinolines. *Eur. J. Org. Chem.* 5214–5228 (2014).
9. Boehner, C. M. et al. Aryl–Aryl Bond Formation by the Fluoride-Free Cross-Coupling of Aryldisiloxanes with Aryl Bromides. *Chem. Eur. J.* **17**, 13230–13239 (2011).
10. Roesner, S. & Buchwald, S. L. Continuous-Flow Synthesis of Biaryls by Negishi Cross-Coupling of Fluoro- and Trifluoromethyl-Substituted (Hetero)arenes. *Angew. Chem. Int. Ed.* **55**, 10463–10467 (2016).
11. Ma, G., Leng, Y., Wu, Y. & Wu, Y. Facile synthesis of 3-arylpyridine derivatives by palladacycle-catalyzed Stille cross-coupling reaction. *Tetrahedron* **69**, 902–909 (2013).
12. Li, Y., Liu, W. & Kuang, C. Direct arylation of pyridines without the use of a transition metal catalyst. *Chem. Commun*. **50**, 7124–7127 (2014).
13. Molander, G. A., Trice, S. L. J. & Kennedy, S. M. Scope of the Two-Step, One-Pot Palladium-Catalyzed Borylation/Suzuki Cross-Coupling Reaction Utilizing Bis-Boronic Acid. *J. Org. Chem.* **77**, 8678–8688 (2012).
14. Ge, S. & Hartwig, J. F. Highly Reactive, Single-Component Nickel Catalyst Precursor for Suzuki–Miyuara Cross-Coupling of Heteroaryl Boronic Acids with Heteroaryl Halides. *Angew. Chem. Int. Ed.* **51**, 12837–12841 (2012).
15. Liu, Z., Dong, N., Xu, M., Sun, Z. & Tu, T. Mild Negishi Cross-Coupling Reactions Catalyzed by Acenaphthoimidazolylidene Palladium Complexes at Low Catalyst Loadings. *J. Org. Chem.* **78**, 7436–7444 (2013).
16. Molander, G. A. & Beaumard, F. Nickel-Catalyzed C−O Activation of Phenol Derivatives with Potassium Heteroaryltrifluoroborates. *Org. Lett.* **12**, 4022–4025 (2010).
17. Cheng, Y., Gu, X. & Li, P. Visible-Light Photoredox in Homolytic Aromatic Substitution: Direct Arylation of Arenes with Aryl Halides. *Org. Lett.* **15**, 2664–2667 (2013).

18. Budén, M. E., Guastavino, J. F. & Rossi, R. A. Room-Temperature Photoinduced Direct C–H-Arylation via Base-Promoted Homolytic Aromatic Substitution. *Org. Lett.* **15**, 1174–1177 (2013).
19. Shi, G., Chen, D., Jiang, H., Zhang, Y. & Zhang, Y. Synthesis of Fluorenes Starting from 2-Iodobiphenyls and CH_2Br_2 through Palladium-Catalyzed Dual C–C Bond Formation. *Org. Lett.* **18**, 2958–2961 (2016).
20. Guastavino, J. F., Budén, M. E. & Rossi, R. A. Room-Temperature and Transition-Metal-Free Mizoroki–Heck-type Reaction. Synthesis of *E*-Stilbenes by Photoinduced C–H Functionalization. *J. Org. Chem.* **79**, 9104–9111 (2014).
21. Liu, W., Cao, H., Xin, J., Jin, L. & Lei, A. Cobalt-Catalyzed Direct Arylation of Unactivated Arenes with Aryl Halides. *Chem. Eur. J.* **17**, 3588–3592 (2011).
22. Gauchot, V. & Lee, A.-L. Dual gold photoredox $C(sp^2)$–$C(sp^2)$ cross couplings – development and mechanistic studies. *Chem. Commun.* **52**, 10163–10166 (2016).
23. Liu, Y., Park, S. K., Xiao, Y. & Chae, J. Copper(II)-catalyzed C–O coupling of aryl bromides with aliphatic diols: synthesis of ethers, phenols, and benzo-fused cyclic ethers. *Org. Biomol. Chem.* **12**, 4747–4753 (2014).
24. Zhou, H.-P., Liu, J.-B., Yuan, J.-J. & Peng, Y.-Y. Palladium-catalyzed Suzuki cross-couplings of *N′*-mesyl arylhydrazines *via* C–N bond cleavage. *RSC Adv.* **4**, 25576–25579 (2014).
25. Tang, Z.-Y. & Hu, Q.-S. Room-Temperature Ni(0)-Catalyzed Cross-Coupling Reactions of Aryl Arenesulfonates with Arylboronic Acids. *J. Am. Chem. Soc.* **126**, 3058–3059 (2004).
26. Kumar, A. & Shah, B. A. *Org. Lett.* **17**, 5232–5235 (2015).
27. Lee, D.-H., Taher, A., Ahn, W.-S. & Jin, M.-J. Room temperature Stille cross-coupling reaction of unreactive aryl chlorides and heteroaryl chlorides. *Chem. Commun.* **46**, 478–480 (2010).
28. Hoshiya, N., Shuto, S. & Arisawa, M. The Actual Active Species of Sulfur-Modified Gold-Supported Palladium as a Highly Effective Palladium Reservoir in the Suzuki–Miyaura Coupling. *Adv. Synth. Catal.* **353**, 743–748 (2011).
29. Jiang, H., An, X., Tong, K., Zheng, T., Zhang, Y. & Yu, S. Visible-light-promoted iminyl-radical formation from acyl oximes: a unified approach to pyridines, quinolines, and phenanthridines. *Angew. Chem. Int. Ed.* **54**, 4055–4059 (2015).
30. Wang, X. et al. Palladium-Catalyzed Addition of Potassium Aryltrifluoroborates to Aliphatic Nitriles: Synthesis of Alkyl Aryl Ketones, Diketone Compounds, and 2-Arylbenzo[*b*]furans. *J. Org. Chem.* **78**, 5273–5281 (2013).
31. Tang, S., Zeng, L., Liu, Y. & Lei, A. Zinc-Catalyzed Dehydrogenative Cross-Coupling of Terminal Alkynes with Aldehydes: Access to Ynones. *Angew. Chem. Int. Ed.* **54**, 15850–15853 (2015).
32. Frisch, M. J. et al. *Gaussian 09* Revision D.01 (Gaussian 2013).
33. Chai J.-D. & Head-Gordon M. Long-range corrected hybrid density functionals with damped atom–atom dispersion corrections. *Phys. Chem. Chem. Phys.* **10**, 6615–6620 (2008).
34. Weigend, F. & Ahlrichs, R. Balanced basis sets of split valence, triple zeta valence and quadruple zeta valence quality for H to Rn: Design and assessment of accuracy. *Phys. Chem. Chem. Phys.* **7**, 3297–3305 (2005).
35. Weigend, F. Accurate Coulomb-fitting basis sets for H to Rn. *Phys. Chem. Chem. Phys.* **8**, 1057–1065 (2006).

36. Zhao, Y. & Truhlar, D. G. The M06 suite of density functionals for main group thermochemistry, thermochemical kinetics, noncovalent interactions, excited states, and transition elements: two new functionals and systematic testing of four M06-class functionals and 12 other functionals. *Theor. Chem. Acc*. **120**, 215–241 (2008).
37. Marenich, A. V., Cramer, C. J. & Truhlar, D. G. Universal Solvation Model Based on Solute Electron Density and on a Continuum Model of the Solvent Defined by the Bulk Dielectric Constant and Atomic Surface Tensions. *J. Phys. Chem. B* **113**, 6378−6396 (2009).
38. Legault, C. Y. CYLView, 1.0b; Université de Sherbrooke: Canada, http://www.cylview.org (2009).
39. Chatupheeraphat, A. et al. *J. Am. Chem. Soc.* **140**, 3724–3735 (2018).
