# Supplementary material for: Nickel-catalyzed Suzuki–Miyaura cross-couplings of aldehydes
Source: Nat Commun. 2019 Apr 29;10:1957. doi: 10.1038/s41467-019-09766-x (PMC6488620; doi:10.1038/s41467-019-09766-x)
Supplement: Supplementary file 2 — Description of Additional Supplementary Files [file 41467_2019_9766_MOESM2_ESM.pdf]

Description of Additional Supplementary Information

File Name: Supplementary Data 1

Description: Cartesian coordinates and energies of calculated structures

PPr.
