# Supplementary material for: Nickel-catalyzed Suzuki–Miyaura cross-couplings of aldehydes
Source: Nat Commun. 2019 Apr 29;10:1957. doi: 10.1038/s41467-019-09766-x (PMC6488620; doi:10.1038/s41467-019-09766-x)
Supplement: Supplementary file 3 — Supplementary Data [file 41467_2019_9766_MOESM3_ESM.pdf]

Cartesian coordinates and energies of calculated structures

PPr_3:

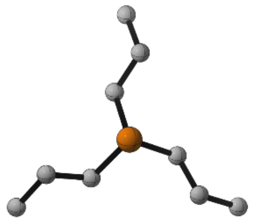

E_{el} = -696.807996

Zero-point correction = 0.286168

Thermal correction to Energy = 0.300677

Thermal correction to Enthalpy = 0.301621

Thermal correction to Gibbs Free Energy = 0.244136

P	-0.00327700	0.00016200	-0.68414300
C	-1.56705500	0.49209600	0.19839400
H	-1.69528800	1.57659300	0.03888200
H	-1.44133700	0.34795900	1.28710900
C	1.20382100	1.10681600	0.20226500
H	2.20602000	0.67027500	0.05010300
H	1.01068500	1.07424000	1.29027500
C	0.35442800	-1.59946700	0.19993000
H	-0.52294000	-2.25216400	0.05154200
H	0.42859000	-1.41649800	1.28760300
C	1.60615200	-2.31736600	-0.30636100
H	2.48582500	-1.66176100	-0.18832400
H	1.50648900	-2.49471400	-1.39026900
C	-2.81548800	-0.23954100	-0.29590000
H	-2.69260900	-1.32644200	-0.15140700
H	-2.91668900	-0.08950600	-1.38385200
C	1.20378400	2.55189600	-0.29718900
H	0.20306900	2.99575600	-0.15817900
H	1.38745200	2.55857300	-1.38469900
C	2.24007200	3.42352500	0.40559300
H	3.25657000	3.02456400	0.26037600
H	2.22612800	4.45406200	0.02011900
H	2.05400400	3.46846100	1.49039000
C	1.86611100	-3.63780100	0.41173400
H	1.02077900	-4.33270400	0.28512400
H	2.76841300	-4.13449300	0.02461900
H	2.00709900	-3.48151600	1.49296700
C	-4.08894100	0.21875800	0.40814300
H	-4.26184300	1.29537000	0.25171300
H	-4.97237200	-0.31993900	0.03389300
H	-4.02618000	0.04795400	1.49451300

Ph-CHO (1b):

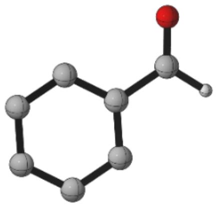

E_{el} = -345.460567

Zero-point correction = 0.111041

Thermal correction to Energy = 0.117301

Thermal correction to Enthalpy = 0.118245

Thermal correction to Gibbs Free Energy = 0.080524

C	1.32339800	-1.32949500	0.00000100
C	-0.04711300	-1.09764400	0.00000000
C	-0.53234100	0.21601500	-0.00000100
C	0.36097600	1.29130000	-0.00000100
C	1.73453300	1.05848800	0.00000000
C	2.21324700	-0.25155400	0.00000100
H	1.70626900	-2.35220900	0.00000100
H	-0.76762400	-1.91841000	0.00000000
H	-0.02540900	2.31484600	-0.00000100
H	2.43402700	1.89681200	0.00000000
H	3.28998900	-0.43570000	0.00000100
C	-1.99663400	0.46612600	-0.00000200
H	-2.28346800	1.54904800	0.00001600
O	-2.83627300	-0.39672600	0.00000000

4a:

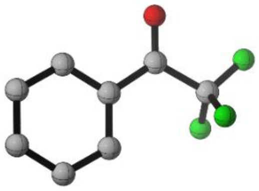

E_{el} = -682.532941

Zero-point correction = 0.117094

Thermal correction to Energy = 0.126710

Thermal correction to Enthalpy = 0.127654

Thermal correction to Gibbs Free Energy = 0.080502

C	1.00711100	-1.05747200	0.01775300
C	0.66107100	0.30152400	0.00813300
C	1.67247300	1.27272900	-0.00759100
C	3.00922400	0.89475100	-0.01500700
C	3.34783300	-0.45953900	-0.00558700

C	2.34834700	-1.43128800	0.01123300
H	0.24084000	-1.83216400	0.03263800
H	1.38306400	2.32513200	-0.01431300
H	3.79117300	1.65624600	-0.02812100
H	4.39825500	-0.75899900	-0.01127500
H	2.61350800	-2.49020400	0.01973100
C	-0.74285300	0.79913200	0.01436200
O	-1.03432100	1.96552800	0.02802800
C	-1.89972600	-0.23811500	-0.00562500
F	-1.82508200	-1.00292400	-1.09943300
F	-3.07210700	0.36456600	0.00117400
F	-1.83316200	-1.04103900	1.06171200

2a:

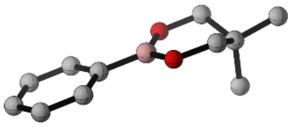

E_{el} = -603.427609

Zero-point correction = 0.248218

Thermal correction to Energy = 0.260973

Thermal correction to Enthalpy = 0.261917

Thermal correction to Gibbs Free Energy = 0.208762

C	-3.65679000	1.20798600	-0.10910800
C	-2.27166800	1.20409900	0.04385100
C	-1.55568600	-0.00016500	0.12222600
C	-2.27196600	-1.20423900	0.04396300
C	-3.65709900	-1.20776700	-0.10907200
C	-4.35094700	0.00019400	-0.18628800
H	-4.19835400	2.15497400	-0.16933000
H	-1.72808000	2.15030600	0.10449200
H	-1.72865600	-2.15059300	0.10483900
H	-4.19891800	-2.15460100	-0.16943900
H	-5.43697100	0.00031000	-0.30788700
C	2.06695200	-1.23018900	0.48590400
C	2.06679100	1.23060300	0.48525500
C	2.74966000	0.00008300	-0.12608300
H	2.43004800	-2.15053900	0.00063700
H	2.32114700	-1.30260800	1.55890300
H	2.42929300	2.15058400	-0.00114300
H	2.32163900	1.30403200	1.55804400
B	0.00276600	-0.00018400	0.28741900
O	0.66447700	1.19197700	0.34750400
O	0.66464200	-1.19226700	0.34725800

C	2.58695100	-0.00040300	-1.65167100
H	3.06170900	0.88960500	-2.09245300
H	3.06065700	-0.89143300	-2.09158300
H	1.53063400	0.00001400	-1.95735200
C	4.23278400	0.00023500	0.24461600
H	4.73773600	-0.88792800	-0.16562600
H	4.73754600	0.88881000	-0.16496100
H	4.37989300	-0.00031500	1.33611000

PhCF$_3$CHOB(nep):

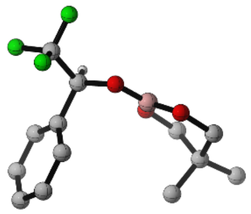

E_{el} = -1055.065704

Zero-point correction = 0.287701

Thermal correction to Energy = 0.305976

Thermal correction to Enthalpy = 0.306921

Thermal correction to Gibbs Free Energy = 0.239501

C	1.22994100	-2.64039900	-1.34169900
C	1.15008500	-1.26646600	-1.12527000
C	1.40650500	-0.73958500	0.14366200
C	1.75100700	-1.59882200	1.18786400
C	1.83669700	-2.97216700	0.96980800
C	1.57434100	-3.49580300	-0.29547000
H	1.02752800	-3.04578700	-2.33543200
H	0.88238400	-0.59151000	-1.94050600
H	1.94791100	-1.18986700	2.18179700
H	2.10462300	-3.63787300	1.79304500
H	1.63905200	-4.57254000	-0.46653100
C	1.31866800	0.75623900	0.37359800
H	1.13582500	0.94632100	1.44462700
C	2.63592300	1.46022100	0.04687700
F	3.61831700	0.96818300	0.80949400
F	2.98913700	1.30410400	-1.22671200
F	2.55072000	2.76730700	0.29108800
O	0.33767000	1.35284100	-0.42242600
C	-2.64346500	0.26526400	1.37600000
C	-3.25913500	1.15575200	-0.83420700
C	-3.44648400	-0.04319800	0.10639100
H	-3.10957300	1.10691800	1.91905200
H	-2.63981700	-0.60469300	2.05186700

H	-3.73654500	0.95786900	-1.80703400
H	-3.74956500	2.04690000	-0.40203500
B	-0.97948000	1.13382600	-0.12462600
O	-1.89941000	1.44259200	-1.07412100
O	-1.30112700	0.58950500	1.08423900
C	-2.92698600	-1.33167700	-0.54668900
H	-3.08844000	-2.19230100	0.12030300
H	-3.45511600	-1.52895000	-1.49213400
H	-1.84999900	-1.28732700	-0.76697000
C	-4.92694200	-0.18686400	0.45820500
H	-5.52243900	-0.40496800	-0.44163800
H	-5.08198700	-1.01355400	1.16858800
H	-5.32799900	0.73142100	0.91534100

3Ba:

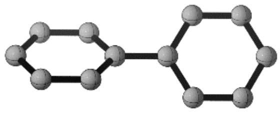

E_{el} = -463.104196

Zero-point correction = 0.183395

Thermal correction to Energy = 0.192204

Thermal correction to Enthalpy = 0.193149

Thermal correction to Gibbs Free Energy = 0.148803

C	-2.85570400	1.12792900	-0.42187900
C	-1.46269200	1.12725400	-0.42206600
C	-0.74367300	0.00000000	0.00000600
C	-1.46268600	-1.12725400	0.42208900
C	-2.85569800	-1.12792900	0.42192400
C	-3.55811900	0.00000000	0.00002800
H	-3.39591000	2.01434700	-0.76179100
H	-0.92250400	2.00823500	-0.77662600
H	-0.92249300	-2.00823600	0.77664000
H	-3.39589900	-2.01434600	0.76184500
H	-4.65027800	0.00000100	0.00003700
C	0.74367300	0.00000000	-0.00000600
C	1.46269200	1.12725500	0.42206400
C	1.46268600	-1.12725500	-0.42208700
C	2.85570400	1.12793000	0.42187700
H	0.92250400	2.00823600	0.77662200
C	2.85569800	-1.12793000	-0.42192200
H	0.92249300	-2.00823700	-0.77663700
C	3.55811900	0.00000000	-0.00002800
H	3.39591000	2.01434800	0.76178800

H	3.39589900	-2.01434800	-0.76184100
H	4.65027800	0.00000100	-0.00003700

CO:

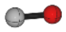

E_{el} = -113.296344

Zero-point correction = 0.005191

Thermal correction to Energy = 0.007552

Thermal correction to Enthalpy = 0.008496

Thermal correction to Gibbs Free Energy = -0.013931

C	0.00000000	0.00000000	-0.64465400
O	0.00000000	0.00000000	0.48349000

Ph:

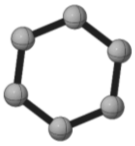

E_{el} = -232.145466

Zero-point correction = 0.101497

Thermal correction to Energy = 0.105856

Thermal correction to Enthalpy = 0.106800

Thermal correction to Gibbs Free Energy = 0.074048

C	-1.28712600	-0.53785700	0.00000200
C	-0.17759000	-1.38342000	0.00011100
C	1.10937500	-0.84572700	-0.00008700
C	1.28708000	0.53796100	0.00001900
C	0.17770600	1.38340200	0.00007600
C	-1.10944400	0.84564200	-0.00008400
H	-2.29476600	-0.95866600	-0.00006200
H	-0.31730300	-2.46650800	0.00004800
H	1.97807300	-1.50730100	-0.00018100
H	2.29481300	0.95852800	0.00000200
H	0.31717000	2.46651400	0.00004200
H	-1.97799400	1.50742500	-0.00006900

phenylboronic acid:

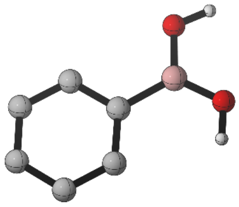

E_{el} = -408.172935

Zero-point correction = 0.126715

Thermal correction to Energy = 0.133636

Thermal correction to Enthalpy = 0.134580

Thermal correction to Gibbs Free Energy = 0.095340

C	0.56624000	1.20492000	-0.00001900
C	-0.17696400	0.01486700	0.00003900
C	0.53249600	-1.19682900	0.00005600
C	1.92546000	-1.21884000	0.00002500
C	2.64178800	-0.02187300	-0.00003000
C	1.95982400	1.19424100	-0.00005100
H	0.05942300	2.17625000	-0.00004100
H	-0.02568600	-2.13598900	0.00010300
H	2.45673400	-2.17336300	0.00004400
H	3.73427300	-0.03650700	-0.00005600
H	2.51486300	2.13501500	-0.00009500
B	-1.75347200	-0.00165900	0.00010800
O	-2.52682800	1.12480000	0.00007700
O	-2.38494400	-1.20621100	-0.00014800
H	-3.34189300	-1.10975400	-0.00019500
H	-2.02923800	1.94501400	0.00014800

PhCH(OH)(CF_3):

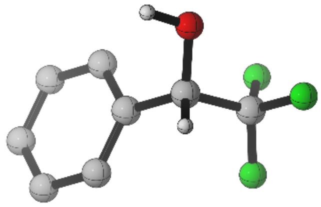

E_{el} = -683.744477

Zero-point correction = 0.140356

Thermal correction to Energy = 0.150492

Thermal correction to Enthalpy = 0.151436

Thermal correction to Gibbs Free Energy = 0.103698

O	1.23572600	1.55520700	1.06155700
C	0.83757400	0.23713300	0.83015500
H	1.03343900	-0.41194300	1.70579200
C	-0.61974000	0.10799600	0.43289400
C	-1.23513600	1.13274100	-0.29143900
C	-1.34924900	-1.03812200	0.75772100
C	-2.56827500	1.01448100	-0.67816700
H	-0.65784900	2.02238800	-0.55055600
C	-2.68086500	-1.15839700	0.36643100
H	-0.87079800	-1.84497700	1.31875100

C	-3.29303800	-0.13088300	-0.35076100
H	-3.04287400	1.82051000	-1.24193400
H	-3.24404200	-2.05742300	0.62560000
H	-4.33813700	-0.22369800	-0.65392600
C	1.76484700	-0.29752800	-0.26404800
F	3.04148500	-0.20092000	0.10134200
F	1.61655700	0.35813900	-1.41248900
F	1.50404900	-1.58941100	-0.49145200
H	0.63893400	1.93869400	1.71049400

A:

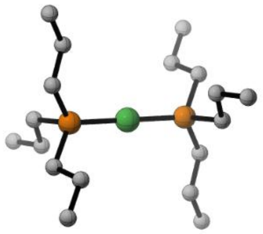

E_{el} = -2901.966670

Zero-point correction = 0.575208

Thermal correction to Energy = 0.606693

Thermal correction to Enthalpy = 0.607637

Thermal correction to Gibbs Free Energy = 0.509999

Ni	0.00000000	0.00000000	0.00000000
P	-2.14028700	0.00184600	0.00936100
P	2.14028600	-0.00184600	-0.00936100
C	2.99183200	0.04476100	1.63781000
H	4.08003900	0.15382600	1.48216700
H	2.83318000	-0.94346200	2.10100600
C	-2.99183200	-0.04476000	-1.63781000
H	-4.08003900	-0.15382600	-1.48216700
H	-2.83318100	0.94346300	-2.10100600
C	-2.97892600	-1.40385900	0.88151500
H	-4.06473400	-1.20684300	0.93164600
H	-2.84610200	-2.29337600	0.24278200
C	-2.97714900	1.45612800	0.79963700
H	-4.06725800	1.38188100	0.63667700
H	-2.80987500	1.36076500	1.88573600
C	2.97892600	1.40385900	-0.88151600
H	4.06473400	1.20684300	-0.93164600
H	2.84610100	2.29337600	-0.24278200
C	2.97714900	-1.45612800	-0.79963700
H	2.80987500	-1.36076600	-1.88573500
H	4.06725800	-1.38188100	-0.63667600
C	2.41277800	1.68779500	-2.27220500

H	1.32885600	1.86938700	-2.18018500
H	2.51275200	0.79027200	-2.90637500
C	2.46514800	1.13542800	2.57007800
H	1.37587400	1.00702600	2.68537900
H	2.60250800	2.12494900	2.10199100
C	-2.46514800	-1.13542700	-2.57007800
H	-1.37587400	-1.00702600	-2.68537900
H	-2.60250900	-2.12494800	-2.10199200
C	-2.41277900	-1.68779500	2.27220400
H	-1.32885600	-1.86938900	2.18018400
H	-2.51275200	-0.79027300	2.90637500
C	-2.44529500	2.80621200	0.32045000
H	-1.35445300	2.83270600	0.48069000
H	-2.59049400	2.89814200	-0.76936200
C	2.44529500	-2.80621200	-0.32044900
H	1.35445400	-2.83270600	-0.48068900
H	2.59049500	-2.89814200	0.76936300
C	3.14229200	1.12695900	3.93680100
H	2.74989600	1.92565800	4.58410500
H	2.98117600	0.16743800	4.45341100
H	4.23016000	1.27499200	3.84460400
C	3.08737300	2.87134100	-2.95839300
H	2.65013300	3.06354800	-3.94969700
H	2.97780100	3.78990900	-2.36005100
H	4.16578500	2.69310200	-3.09617600
C	-3.14229200	-1.12695800	-3.93680200
H	-2.98117600	-0.16743600	-4.45341100
H	-2.74989600	-1.92565700	-4.58410600
H	-4.23016100	-1.27499100	-3.84460400
C	-3.08737300	-2.87134200	2.95839200
H	-2.97780200	-3.78991000	2.36005000
H	-2.65013300	-3.06354900	3.94969600
H	-4.16578500	-2.69310300	3.09617600
C	-3.10718100	3.98838600	1.02100700
H	-2.95084400	3.94153000	2.11049900
H	-2.69670000	4.94564900	0.66614100
H	-4.19426800	4.00307100	0.84264000
C	3.10718300	-3.98838700	-1.02100500
H	2.69670100	-4.94564900	-0.66613900
H	2.95084500	-3.94153100	-2.11049800
H	4.19426900	-4.00307100	-0.84263900

B:

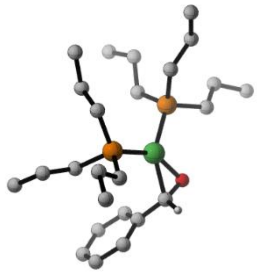

E_{el} = -3247.462974

Zero-point correction =0.691187

Thermal correction to Energy = 0.729576

Thermal correction to Enthalpy = 0.730520

Thermal correction to Gibbs Free Energy = 0.619594

Ni	0.01087500	-0.65033800	-0.82191600
P	-2.11214500	-0.34417900	-0.26551800
P	1.28203200	1.03860500	-0.33963400
C	1.20132100	-1.99108900	-1.53283000
O	-0.03665500	-2.40446200	-1.42497300
C	2.24029100	-2.42578000	-0.56129900
C	3.59792600	-2.18194000	-0.81437000
C	4.20131400	-3.03679600	1.36492500
C	1.88519500	-3.02003300	0.65924700
C	2.85487900	-3.31681600	1.61215600
H	3.89144800	-1.74659000	-1.77460000
H	4.95994400	-3.26846100	2.11574300
H	0.82859800	-3.22763600	0.84174300
H	2.56065300	-3.77255500	2.56099300
C	2.49398400	1.41965300	-1.69188300
C	0.55709900	2.69168700	0.08672800
C	2.31700600	0.64211400	1.13840400
C	-2.39240600	-0.53396400	1.56040400
C	-2.85359900	1.28411000	-0.75583300
C	-3.17220600	-1.64013500	-1.04979800
C	3.14662100	2.79957500	-1.77392100
H	1.96669300	1.20535300	-2.63558600
H	3.26056900	0.63088100	-1.60118700
C	4.20053600	2.87264700	-2.87486500
H	2.37283200	3.56177900	-1.96253900
H	3.60511500	3.06630700	-0.80900200
C	3.39381300	1.62252400	1.59431200
H	2.76904500	-0.33858000	0.92617600
H	1.59721600	0.45365900	1.95357400
C	4.06935900	1.16241400	2.88305600
H	4.15613700	1.72156000	0.80459200

H	2.96512800	2.62955100	1.73825400
C	-0.19323600	2.70656000	1.41860500
H	-0.13032600	2.94970800	-0.73711400
H	1.34113800	3.46536100	0.09787500
C	-0.99885300	3.98303700	1.63496400
H	-0.86234900	1.83349900	1.47766600
H	0.52689900	2.58537300	2.24448000
C	-4.36400000	1.44557900	-0.92491900
H	-2.35475900	1.53214200	-1.70804500
H	-2.47198000	2.02144000	-0.03040000
C	-4.74076000	2.86511300	-1.33943500
H	-4.72597200	0.73415600	-1.68475400
H	-4.88397200	1.18793600	0.00983300
C	-4.51676100	-2.01651800	-0.42771800
H	-2.51327300	-2.52291000	-1.09866100
H	-3.30101600	-1.32443000	-2.09993200
C	-5.21259400	-3.13613300	-1.19683300
H	-4.36086700	-2.34058500	0.61456700
H	-5.18230200	-1.14067300	-0.37766000
C	-3.59819000	0.12944700	2.22352200
H	-1.46818200	-0.17479000	2.04124500
H	-2.39895600	-1.62333100	1.73812400
C	-3.63941600	-0.11903200	3.72826300
H	-3.56667300	1.21603400	2.03554700
H	-4.53014400	-0.23473800	1.76458300
H	3.76169000	2.64412000	-3.85885100
H	5.00966700	2.14664000	-2.69753700
H	-2.73097800	0.26507000	4.21860900
H	-3.70599900	-1.19573100	3.95004300
H	-4.58606100	-4.04100000	-1.23068500
H	-5.41568700	-2.83690300	-2.23719100
H	-4.25539100	3.14311600	-2.28829100
H	-4.42448800	3.59701000	-0.57907600
H	-1.76635700	4.10724500	0.85359000
H	-0.35101600	4.87304400	1.60296000
H	4.52180900	0.16716300	2.75050400
H	3.34076600	1.08750300	3.70585600
H	4.86094200	1.85943100	3.19650200
H	4.65367300	3.87347100	-2.93388900
H	-5.82725500	2.97152300	-1.47493400
H	-1.51024300	3.97453900	2.60915800
H	-4.50638800	0.37329000	4.19338600
H	-6.17211500	-3.40630100	-0.73105000
C	4.56897800	-2.47560800	0.14126100
H	5.62136800	-2.27113100	-0.07117800

H	1.59406100	-1.76651000	-2.54558000

[B-C]‡:

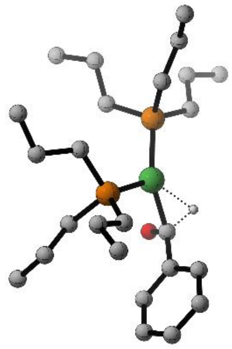

E_{el} = -3247.424003

Zero-point correction = 0.687762

Thermal correction to Energy = 0.726231

Thermal correction to Enthalpy = 0.727175

Thermal correction to Gibbs Free Energy = 0.614750

Ni	-0.20820700	-0.95407700	0.00557800
P	-2.37070100	-0.53276000	-0.06784500
P	1.08758400	0.78556400	-0.05782100
C	1.06457600	-2.28458300	0.46661800
O	0.68222000	-2.39431900	1.64814200
C	2.49962500	-2.36555600	0.06721900
C	2.85879600	-2.55211900	-1.27126000
C	5.19044400	-2.30842500	-0.69430500
C	3.49997000	-2.18698600	1.02935700
C	4.83835500	-2.14136900	0.64728600
H	2.07336100	-2.69600000	-2.01902800
H	6.24111300	-2.27787500	-0.99194500
H	3.20176900	-2.07777600	2.07438300
H	5.61493000	-1.98430400	1.39973200
C	2.50895100	0.78837000	-1.24986800
C	0.22540900	2.40862800	-0.34765900
C	1.82856800	0.95422400	1.63711700
C	-2.88734300	0.43137800	-1.56959300
C	-3.43355100	-2.05642700	-0.05052200
C	-2.94844900	0.46073600	1.39082800
C	3.22492200	2.07840700	-1.64589000
H	2.11156200	0.30016800	-2.15530300
H	3.23538200	0.06972400	-0.83890500
C	4.34122900	1.81308300	-2.65248800
H	2.50449000	2.79186900	-2.07962700
H	3.64752500	2.56873100	-0.75571300
C	3.20962600	1.58236600	1.81356900

H	1.83423000	-0.07083700	2.04357400
H	1.07831200	1.49260800	2.24231100
C	3.68721700	1.51561000	3.26101900
H	3.93411600	1.05519700	1.17134300
H	3.20069100	2.63156600	1.47653400
C	0.81643000	3.70654300	0.20401100
H	-0.78417300	2.27182400	0.07409300
H	0.07647500	2.48267500	-1.43981900
C	-0.06219700	4.91597700	-0.10292800
H	0.94104100	3.61544100	1.29540200
H	1.82437900	3.87350100	-0.20305800
C	-4.89802700	-1.99905700	0.38680200
H	-2.88615000	-2.75383300	0.60532000
H	-3.34526100	-2.48715600	-1.06389700
C	-5.56321700	-3.37205800	0.34741100
H	-4.95749300	-1.59829900	1.41215000
H	-5.46508000	-1.30139500	-0.24773200
C	-4.21642900	1.30957900	1.30080400
H	-2.09632200	1.10824600	1.65615400
H	-3.02092600	-0.26415100	2.22052200
C	-4.51228200	2.04369000	2.60547100
H	-4.10756900	2.04631400	0.48723700
H	-5.07943900	0.68220400	1.03048600
C	-4.32780500	0.38366400	-2.07862400
H	-2.20839700	0.08471200	-2.36628500
H	-2.58278800	1.47377600	-1.36925000
C	-4.52394000	1.23460200	-3.33015200
H	-4.59964300	-0.66070200	-2.30376500
H	-5.02431200	0.71942700	-1.29537600
H	3.94325600	1.35994800	-3.57426300
H	5.08555900	1.11460100	-2.23834000
H	-3.84935200	0.91153400	-4.13873400
H	-4.31160500	2.29619900	-3.12669700
H	-3.67956200	2.70789300	2.88621600
H	-4.66179900	1.33318800	3.43361000
H	-5.03753000	-4.08583900	1.00074200
H	-5.55431100	-3.78911300	-0.67206500
H	-1.06947700	4.79370000	0.32678500
H	-0.18084900	5.05701100	-1.18895100
H	3.74331800	0.47183800	3.60825300
H	2.99810900	2.05085200	3.93337100
H	4.68535800	1.96413300	3.37742700
H	4.86342600	2.74073200	-2.93099700
H	-6.61087900	-3.32000300	0.67961900
H	0.36759000	5.84070700	0.31028400

H	-5.55552600	1.16525600	-3.70634200
H	-5.42031000	2.65952600	2.52341700
C	4.19830600	-2.52880000	-1.65130600
H	4.47122600	-2.67342000	-2.69904800
H	0.31521800	-2.60660900	-0.39764200

C:

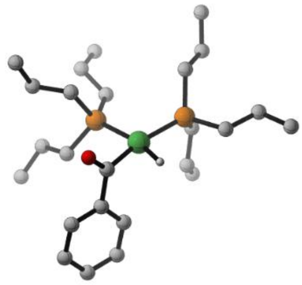

E_{el} = -3247.432694

Zero-point correction = 0.686399

Thermal correction to Energy = 0.725518

Thermal correction to Enthalpy = 0.726462

Thermal correction to Gibbs Free Energy = 0.612160

Ni	-0.09499400	-0.70961300	-0.88162600
P	0.83361600	1.14736800	-0.08456900
P	-2.21681500	-0.55889300	-0.29118400
C	-3.13605100	0.95216700	-0.87141900
C	2.41999400	0.79469100	0.79744500
C	1.25317100	2.20720500	-1.53617500
C	-0.14240900	2.21680100	1.07659400
C	-3.27260600	-1.95662500	-0.87463100
C	-2.46042500	-0.58282800	1.54475600
C	-4.20545500	1.55705500	0.03972300
C	-4.89172600	2.76269500	-0.59469500
C	-4.77175000	-1.92790300	-0.58687800
C	-1.60275000	-1.64919800	2.22762200
C	-1.79088200	-1.68032400	3.74021800
C	2.40554800	3.20762100	-1.44654500
C	2.61942600	3.94196000	-2.76689100
C	0.16783300	3.70775600	1.20773200
C	-0.75782300	4.39302100	2.20865800
C	3.05130500	1.83086900	1.72525500
C	4.35901700	1.32247400	2.32585600
H	-3.57379000	0.68046100	-1.84731600
H	-2.37411800	1.71504600	-1.10038600
H	-4.95870100	0.79681800	0.29986300
H	-3.74585200	1.86345500	0.99382800
H	-5.39994300	2.48488500	-1.53134900

H	-4.16183300	3.55157100	-0.83622400
H	-2.80787600	-2.86414700	-0.45334800
H	-3.08435600	-2.02265300	-1.95902500
C	-5.48112300	-3.19130200	-1.06625900
H	-4.94858500	-1.79977700	0.49419900
H	-5.22564300	-1.05009800	-1.07636600
H	-3.52546600	-0.72136600	1.79189100
H	-2.17907000	0.41120900	1.92628800
H	-1.83346800	-2.64218700	1.80639400
H	-0.54310900	-1.46169400	1.98468600
H	0.31485600	2.71529500	-1.82141300
H	1.46357100	1.47720900	-2.33672100
H	2.22962100	3.93929000	-0.64242900
H	3.33014100	2.67040500	-1.17991500
H	-0.07886100	1.72106000	2.06203900
H	-1.19262100	2.10883100	0.76309100
H	1.21468500	3.86007300	1.50897200
H	0.06010500	4.18880600	0.22181500
H	3.12639100	0.49903800	0.00353800
H	2.23398900	-0.13627400	1.35850200
H	3.23758500	2.77141700	1.18419800
H	2.34849400	2.07760000	2.53860500
H	-2.83594000	-1.90271600	4.00798500
H	-1.53312900	-0.70929500	4.19226800
H	1.72092000	4.51090100	-3.05473100
H	2.83719500	3.23189900	-3.57932800
H	5.10036900	1.11509100	1.53858400
H	4.20107300	0.38465000	2.88098200
H	-0.64347000	3.95901500	3.21467200
H	-1.81409100	4.27711700	1.91678400
H	-5.08179000	-4.08543500	-0.56268400
H	-5.34457900	-3.33474600	-2.14940100
C	1.56619300	-1.23037500	-1.59751600
C	2.42495500	-2.19429500	-0.79415500
O	2.03514500	-0.75265600	-2.61890000
C	1.89373900	-2.90354600	0.28577800
C	4.06956400	-3.82561300	0.77678700
C	3.78587900	-2.31996400	-1.09046800
C	4.60631200	-3.12852800	-0.30802200
H	0.82402500	-2.81063600	0.49554000
H	4.71282200	-4.45896100	1.39256500
H	4.17586800	-1.76304500	-1.94609900
H	5.67011200	-3.22015700	-0.54078800
H	4.79660600	2.05731600	3.01799800
H	3.45822100	4.65102100	-2.70165300

H	-0.54676400	5.47001100	2.28266900
H	-5.64440500	3.19628400	0.08037800
H	-1.15421100	-2.44641300	4.20684900
H	-6.56195700	-3.14587300	-0.86616500
C	2.70980500	-3.71689100	1.07017200
H	2.28588500	-4.26964100	1.91204300
H	-0.34070300	-1.99889800	-1.57652700

D:

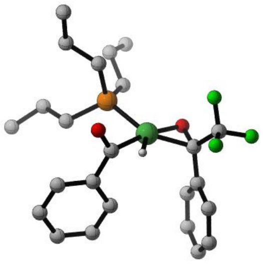

E_{el} = -3233.154430

Zero-point correction = 0.519502

Thermal correction to Energy = 0.553495

Thermal correction to Enthalpy = 0.554439

Thermal correction to Gibbs Free Energy = 0.451476

Ni	0.36838100	-0.56670100	-0.38690000
P	-1.79224800	-0.43441600	0.18829600
C	-2.45543400	1.26206300	0.48389100
C	-2.81968400	-1.19218800	-1.14026000
C	-2.07063400	-1.39590800	1.73593300
C	-4.31529500	-0.88389500	-1.21586500
C	-4.98823500	-1.61510700	-2.37359400
C	-3.44975900	-1.99552500	2.00970200
C	-3.48577300	-2.76076200	3.32934300
C	-3.70752600	1.45575300	1.33826300
C	-4.06896600	2.93150500	1.47972300
H	-2.64433600	-2.27908900	-1.06133700
H	-2.33153000	-0.87661000	-2.07747500
H	-4.81423700	-1.15256100	-0.27212100
H	-4.45591900	0.20223200	-1.34192400
H	-1.76132500	-0.72743100	2.55847400
H	-1.30864400	-2.19098600	1.71081800
H	-4.22028600	-1.20910500	2.01975600
H	-3.71933500	-2.67848000	1.18750400
H	-2.59867800	1.70093700	-0.51813300
H	-1.61838300	1.82773800	0.92673900
H	-4.55767800	0.90785900	0.90408600
H	-3.54155700	1.02295700	2.33846700

H	-4.89959500	-2.70651900	-2.25607000
H	-4.52285700	-1.34671900	-3.33455700
H	-4.26744200	3.38647600	0.49661200
H	-3.24614000	3.49714000	1.94442300
H	-3.26581500	-2.09595700	4.17948000
H	-2.73707600	-3.56784100	3.33827300
C	0.07667000	0.92920000	-1.83882900
C	0.30575600	2.26553600	-1.22016100
O	-0.67541300	0.74431900	-2.77092400
C	1.29952300	2.45693600	-0.25449000
C	0.61116200	4.75632300	-0.00441500
C	-0.52020200	3.33388100	-1.59266000
C	-0.37118100	4.57335100	-0.98236400
H	1.96284200	1.63609400	0.02553900
H	0.72659800	5.73101400	0.47509900
H	-1.27818100	3.16576500	-2.36074900
H	-1.02015700	5.40410000	-1.26749900
H	-4.96636400	3.06755100	2.10101100
H	-6.05876600	-1.36973000	-2.43584600
H	-4.47301800	-3.21387600	3.50287200
C	1.44973200	3.70173400	0.35339200
H	2.22697100	3.84173500	1.10720700
H	0.98147800	0.13909100	-1.67267700
C	4.80859700	1.29585700	1.75569900
C	3.71114300	0.85882400	2.49804200
C	2.82740300	-0.07322400	1.96102900
C	3.02122600	-0.58062300	0.66847600
C	4.13370100	-0.14607400	-0.06675000
C	5.01754600	0.78379300	0.47547500
H	5.50260200	2.02779900	2.17472700
H	3.54365300	1.24563900	3.50596600
H	1.96135200	-0.41999900	2.52703800
H	4.30566600	-0.52305800	-1.07486900
H	5.87659900	1.11493300	-0.11222800
C	1.97360700	-1.49839300	0.10920900
O	0.95408400	-1.81510600	0.85135300
C	2.41435900	-2.55202400	-0.90841900
F	2.87604400	-2.00096700	-2.04771500
F	3.40123200	-3.31941100	-0.42799800
F	1.40859400	-3.35486200	-1.24378900

[D-E]‡:

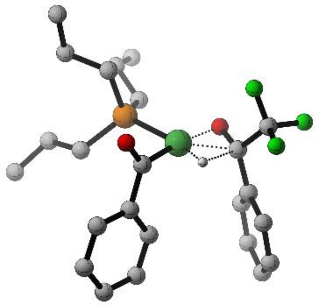

E_{el} = -3233.141942

Zero-point correction = 0.515467

Thermal correction to Energy = 0.549353

Thermal correction to Enthalpy = 0.550297

Thermal correction to Gibbs Free Energy = 0.446698

Ni	0.25409000	-0.49739300	-0.52658400
P	-1.84173700	-0.38087400	0.18565600
C	-2.40359400	1.32030500	0.61432800
C	-2.90850000	-0.98936700	-1.18294000
C	-2.11756300	-1.44291700	1.66366500
C	-4.39494500	-0.63072700	-1.20318100
C	-5.10490500	-1.23971300	-2.40856500
C	-3.51274500	-2.01107500	1.92427500
C	-3.54750400	-2.87655600	3.18037700
C	-3.63883100	1.50309500	1.49538400
C	-3.94921100	2.97780200	1.73436300
H	-2.76498100	-2.08348000	-1.20565100
H	-2.41995300	-0.59976500	-2.09239800
H	-4.88826500	-0.96572600	-0.27763500
H	-4.50382200	0.46565200	-1.23424600
H	-1.76505900	-0.85040700	2.52609600
H	-1.38804800	-2.26134600	1.55680000
H	-4.25189400	-1.19992100	2.01527000
H	-3.82543100	-2.61589200	1.05734000
H	-2.52665100	1.83912300	-0.35189900
H	-1.53430600	1.80473500	1.08941200
H	-4.51219200	1.01206600	1.03987400
H	-3.47583100	1.00370400	2.46463300
H	-5.03996400	-2.33898400	-2.39241400
H	-4.65029500	-0.89417800	-3.34971000
H	-4.15439700	3.49729900	0.78550000
H	-3.09944400	3.48859000	2.21353300
H	-3.28781400	-2.28921500	4.07523700
H	-2.82695800	-3.70589600	3.10816900
C	-0.03748200	1.01098100	-1.61076400

C	0.43317400	2.33788100	-1.06371500
O	-0.67178800	0.96431100	-2.64285300
C	1.21787400	2.41064900	0.09123900
C	1.21644800	4.81941800	-0.04579900
C	0.04427700	3.51797400	-1.70811000
C	0.43306300	4.75386400	-1.20068700
H	1.52944900	1.49274100	0.59606600
H	1.52253400	5.79000800	0.35164100
H	-0.56444200	3.43721800	-2.61146300
H	0.12853600	5.67316400	-1.70611300
H	-4.82819000	3.10148300	2.38385100
H	-6.17023000	-0.96601400	-2.42658200
H	-4.54574900	-3.30981700	3.34068700
C	1.61109000	3.64743300	0.59869800
H	2.22935200	3.69250500	1.49782500
H	1.43155300	-0.24784500	-1.37665100
C	4.59746500	0.79239400	2.25265000
C	3.53138700	0.11862700	2.84640800
C	2.67853200	-0.66171400	2.06883000
C	2.88102300	-0.77387900	0.68965600
C	3.95474500	-0.09749700	0.09822900
C	4.80719100	0.67834800	0.87812800
H	5.26471700	1.40778900	2.86010800
H	3.36229200	0.20068700	3.92230500
H	1.83337400	-1.18809700	2.51487600
H	4.11263100	-0.15606700	-0.97849800
H	5.63765400	1.20674800	0.40537100
C	1.86282200	-1.56482800	-0.09874600
O	0.81935000	-1.99924200	0.51697400
C	2.38808400	-2.50847700	-1.19887800
F	3.13920600	-1.89087500	-2.11802600
F	3.14545700	-3.45579700	-0.63866400
F	1.38772700	-3.10593300	-1.83485100

E:

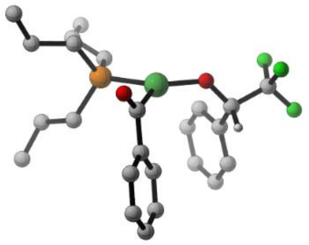

E_{el} = -3233.166619

Zero-point correction = 0.520998

Thermal correction to Energy = 0.554156

Thermal correction to Enthalpy = 0.555100

Thermal correction to Gibbs Free Energy = 0.453744

Ni	0.05336300	-0.24976800	-1.05093900
P	-1.93679800	-0.55863700	-0.24279600
C	-2.46017100	0.73104600	0.96372400
C	-3.19863300	-0.62375100	-1.57896200
C	-1.95897900	-2.17943300	0.64235200
C	-4.66955000	-0.37267100	-1.24492400
C	-5.54948200	-0.43590300	-2.48970700
C	-3.29291600	-2.89296500	0.86722200
C	-3.11167300	-4.22379000	1.59114800
C	-3.54975400	0.41857100	1.98872900
C	-3.87672500	1.63308900	2.85235200
H	-3.06760500	-1.60632000	-2.06465300
H	-2.84764300	0.12082200	-2.31315200
H	-5.02880200	-1.10237000	-0.50230100
H	-4.77350300	0.62025700	-0.77766000
H	-1.43721700	-2.00172900	1.59917300
H	-1.28917400	-2.83612800	0.06128700
H	-3.97825600	-2.25167900	1.44127000
H	-3.77942300	-3.07049900	-0.10560300
H	-2.73498900	1.60800400	0.35146500
H	-1.53763800	1.02758800	1.48811200
H	-4.46453700	0.06754900	1.48776700
H	-3.21466200	-0.40913200	2.63525900
H	-5.49205100	-1.42726100	-2.96568400
H	-5.23150100	0.30866400	-3.23541100
H	-4.26208000	2.46244200	2.23915200
H	-2.98101400	1.99879900	3.37793400
H	-2.65173000	-4.07848800	2.58118800
H	-2.46043400	-4.90182400	1.01797000
C	-0.41312100	1.46801600	-1.49397200
C	0.11260200	2.56877000	-0.61024400
O	-1.05825600	1.70522000	-2.48627800
C	0.76490500	2.28874700	0.59342800
C	1.03272000	4.64969500	1.00718000
C	-0.07754600	3.89987700	-1.00198900
C	0.38208400	4.93597800	-0.19545000
H	0.91810200	1.25016800	0.89739500
H	1.39343200	5.46466200	1.63894600
H	-0.58833500	4.09747500	-1.94661500
H	0.23502500	5.97362300	-0.50295700
H	-4.63768200	1.39146900	3.60858800
H	-6.60394200	-0.24108300	-2.24508700
H	-4.07635300	-4.73032900	1.74018700

C	1.22427600	3.32580600	1.40159000
H	1.73648600	3.09475100	2.33806300
O	1.73112000	-0.68168900	-1.50563200
C	2.88231100	-0.43385200	-0.80972400
H	3.27190200	0.58839600	-1.00226200
C	2.75431700	-0.59747500	0.70357800
C	3.42185000	0.25746300	1.58214700
C	1.92595900	-1.59358300	1.22962900
C	3.24176400	0.14292000	2.96070600
H	4.07641000	1.03547300	1.18091100
C	1.74192600	-1.71120700	2.60544300
H	1.42493200	-2.28002100	0.54342500
C	2.39376100	-0.83639300	3.47600300
H	3.76379800	0.82547000	3.63533100
H	1.09291800	-2.49577500	3.00264800
H	2.24776200	-0.92501400	4.55482100
C	3.97699800	-1.36467700	-1.34821300
F	5.14756400	-1.13678100	-0.73021100
F	4.17494200	-1.17866500	-2.65142200
F	3.67429100	-2.65331200	-1.16373300

F:

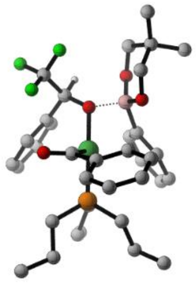

E_{el} = -3836.611060

Zero-point correction = 0.771724

Thermal correction to Energy = 0.819206

Thermal correction to Enthalpy = 0.820150

Thermal correction to Gibbs Free Energy = 0.689885

Ni	0.12588800	-0.07304300	-0.39921800
P	2.26738900	-0.12279900	-0.00328200
C	3.37723100	0.14579500	-1.44455200
C	2.47774700	1.31958900	1.11458900
C	2.84252600	-1.65111800	0.84637400
C	3.84376300	1.98188100	1.27775800
C	3.76196100	3.16876600	2.23286900
C	3.96448000	-1.52749300	1.87607200
C	4.27228800	-2.86532200	2.54029800
C	4.86360000	-0.17493400	-1.28471800

C	5.65819000	0.17963900	-2.53789500
H	2.08037000	0.99619500	2.08809200
H	1.75917000	2.06958100	0.74391600
H	4.58380800	1.25617500	1.65010000
H	4.21019500	2.32710100	0.29660400
H	3.10410500	-2.36649900	0.04767500
H	1.94515300	-2.06674400	1.32861200
H	4.87661700	-1.12793400	1.40550900
H	3.66578700	-0.79752200	2.64600400
H	3.22902300	1.20245400	-1.72278100
H	2.95745600	-0.43399300	-2.28044100
H	5.28426700	0.36197800	-0.42121300
H	4.98515300	-1.24881500	-1.06716500
H	3.42118300	2.84825100	3.22964800
H	3.04285000	3.91669800	1.86376800
H	5.58884900	1.25653500	-2.75746600
H	5.27768300	-0.36285500	-3.41715100
H	4.60147400	-3.61093000	1.79990700
H	3.37998300	-3.27048900	3.04269000
C	0.37866800	-1.08208900	-1.91538600
C	0.47244300	-2.56313600	-1.68123900
O	0.55217100	-0.58558300	-2.99814300
C	1.27065700	-3.32208900	-2.54621700
C	0.83884100	-5.28635800	-1.21166800
C	-0.15977600	-3.17291700	-0.59229600
C	0.01945600	-4.53623800	-0.36753400
H	1.73632000	-2.83067300	-3.40345800
H	0.98763600	-6.35212800	-1.02362600
H	-0.81762400	-2.58291100	0.05618600
H	-0.48429700	-5.01504900	0.47495900
H	6.72252900	-0.07091700	-2.41997100
H	4.73871500	3.66113400	2.34875100
H	5.06674700	-2.76484300	3.29427400
C	1.46269300	-4.67937700	-2.30341000
H	2.09526100	-5.26991500	-2.96984100
O	-1.70962100	0.45519300	-0.39920400
C	-2.04174200	1.72460900	-0.88173600
H	-2.80558100	2.15609600	-0.21395500
C	-0.85200300	2.67379400	-0.92815400
C	0.19283100	2.50782400	-1.85049700
C	-0.76271800	3.70450200	0.01128700
C	1.29479800	3.36125000	-1.82352600
H	0.15159200	1.71126900	-2.59632700
C	0.34787000	4.54760100	0.04785500
H	-1.57594300	3.84945700	0.72631700

C	1.38033400	4.37839200	-0.87269800
H	2.08920600	3.23465700	-2.56298100
H	0.40076400	5.34280100	0.79440100
H	2.24627200	5.04419000	-0.85474300
C	-2.73332000	1.57750700	-2.24009300
F	-3.04181800	2.77961000	-2.73790900
F	-3.87564400	0.88852600	-2.12716400
F	-1.98906000	0.94088900	-3.14372600
C	0.11869200	1.65120100	3.76891200
C	-0.80930800	1.45813600	2.74717400
C	-1.00929800	0.19936300	2.15471100
C	-0.22306700	-0.86150700	2.63607500
C	0.71188800	-0.68221200	3.65859600
C	0.88271700	0.57733700	4.23192400
H	0.23963000	2.63953800	4.21948700
H	-1.43463500	2.29476400	2.42856800
H	-0.38815500	-1.86078000	2.22479500
H	1.29428700	-1.53280500	4.02330300
H	1.60364600	0.72198700	5.04048200
C	-3.61375700	-1.83226400	0.28763400
C	-4.63437400	0.30124300	0.99524300
C	-4.81953700	-1.22504500	1.02183000
H	-3.68227800	-2.93422000	0.29707100
H	-3.62944400	-1.50766600	-0.77132200
H	-5.38918700	0.77721800	1.64533400
H	-4.81940500	0.65844500	-0.03386200
B	-2.22120900	-0.05973000	1.09693000
O	-3.36654100	0.71146200	1.43525400
O	-2.41138900	-1.46060900	0.89974500
C	-4.86234500	-1.72979500	2.46746300
H	-5.72609300	-1.30348800	3.00208900
H	-4.95349500	-2.82762300	2.49735100
H	-3.94954300	-1.44885200	3.01108700
C	-6.10731900	-1.58983000	0.28500800
H	-6.27362900	-2.67873000	0.29957300
H	-6.98107300	-1.11328100	0.75761700
H	-6.07391300	-1.26619100	-0.76753300

[F-G]‡:

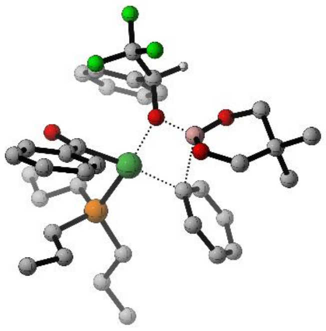

E_{el} = -3836.598457

Zero-point correction = 0.771341

Thermal correction to Energy = 0.818207

Thermal correction to Enthalpy = 0.819151

Thermal correction to Gibbs Free Energy = 0.690141

Ni	-0.23769300	-0.00392400	0.06754000
P	-2.21918500	-0.91111000	0.09483300
C	-3.38275500	-0.31784700	1.39145800
C	-2.94475900	-0.47264000	-1.54060200
C	-2.32547400	-2.73889000	0.23759400
C	-4.45565600	-0.26726800	-1.63875500
C	-4.87572700	0.10473000	-3.05725700
C	-3.69542800	-3.39110500	0.07002800
C	-3.62411200	-4.90257400	0.26932600
C	-2.90346300	-0.67023800	2.80277600
C	-3.63920900	0.11390100	3.88331600
H	-2.59990300	-1.25246400	-2.24118500
H	-2.44219800	0.46238100	-1.83190700
H	-4.99892500	-1.16910500	-1.31297500
H	-4.75181500	0.53950300	-0.94982900
H	-1.89330100	-2.99189600	1.21843900
H	-1.61630200	-3.14490800	-0.49921300
H	-4.41742300	-2.96139600	0.78449800
H	-4.09067400	-3.17441100	-0.93568500
H	-4.38636600	-0.73266400	1.20606700
H	-3.46743800	0.77240500	1.27520500
H	-3.02975800	-1.75277100	2.97287600
H	-1.82292400	-0.46815400	2.88659300
H	-4.61276500	-0.69137700	-3.77183000
H	-4.36639400	1.02442000	-3.38417800
H	-4.72719800	-0.05010700	3.82761700
H	-3.45014000	1.19267300	3.77080000
H	-3.27038100	-5.15029500	1.28235700
H	-2.92180300	-5.36019200	-0.44475200

C	-1.18674400	1.61310000	0.42057200
C	-0.97448700	2.16318000	1.81696400
O	-1.94035700	2.20894500	-0.32789400
C	0.01940900	1.66354400	2.66582800
C	-0.71113400	3.16181900	4.41633700
C	-1.82372100	3.17680200	2.27613900
C	-1.69833600	3.67159700	3.57030400
H	0.71315200	0.89512200	2.31341600
H	-0.60846400	3.54982600	5.43254400
H	-2.58176000	3.56080500	1.58958000
H	-2.37026500	4.45697200	3.92430500
H	-3.30356400	-0.18192500	4.88817000
H	-5.96056200	0.27423600	-3.12584700
H	-4.60768900	-5.37516400	0.12945800
C	0.15025500	2.16427700	3.96035200
H	0.93293200	1.77586500	4.61565000
O	1.45645900	0.92632300	-0.30252400
C	1.73054100	1.76340200	-1.41088900
H	2.70977500	1.46908800	-1.81535300
C	0.70896800	1.60168300	-2.51713400
C	-0.33295200	2.49576800	-2.76775000
C	0.81763900	0.44323000	-3.29727300
C	-1.25770900	2.22497500	-3.77562100
H	-0.45187700	3.39594700	-2.16762600
C	-0.11466800	0.16609400	-4.29184900
H	1.64582800	-0.24434400	-3.10620400
C	-1.15881800	1.06028200	-4.53190500
H	-2.07261300	2.92902700	-3.95516100
H	-0.02167800	-0.74585900	-4.88565600
H	-1.89458900	0.84870400	-5.31101300
C	1.94838100	3.17333400	-0.87005700
F	2.97110000	3.17191900	-0.01063700
F	0.89238500	3.67259400	-0.23054100
F	2.25757600	4.00780200	-1.86662100
C	0.89274500	-3.94750200	-1.20159100
C	0.81347600	-2.55317300	-1.20929500
C	0.77276000	-1.79015300	-0.02629200
C	0.83060800	-2.52465000	1.17591200
C	0.89986800	-3.91758600	1.20634600
C	0.92702700	-4.63676200	0.01110600
H	0.92464800	-4.49959900	-2.14466000
H	0.77551900	-2.04304300	-2.17518100
H	0.81329100	-1.98207100	2.12757100
H	0.93478800	-4.44578700	2.16296800
H	0.98699700	-5.72776400	0.02440600

C	3.75722000	-1.04514900	1.77911200
C	4.54843300	-0.71195700	-0.56425400
C	4.66245100	-1.62541800	0.66095600
H	3.32944500	-1.87443400	2.36934700
H	4.36408800	-0.43182200	2.46474600
H	5.11113200	-1.14254000	-1.40687000
H	4.99010200	0.27771400	-0.33930600
B	2.32278000	-0.17634400	0.01425300
O	3.20920300	-0.55183600	-0.98071900
O	2.71115100	-0.21420900	1.33179700
C	4.24060600	-3.04597100	0.27062500
H	4.93484900	-3.45786100	-0.47906500
H	4.25084900	-3.71521500	1.14469600
H	3.23217000	-3.06527700	-0.15940600
C	6.11342200	-1.63853900	1.15098600
H	6.20955500	-2.25077800	2.06144000
H	6.78316300	-2.06937400	0.39041700
H	6.47261700	-0.62426400	1.38669400

G:

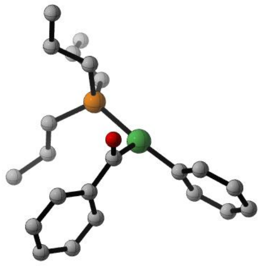

E_{el} = -2781.540868

Zero-point correction = 0.481055

Thermal correction to Energy = 0.509864

Thermal correction to Enthalpy = 0.510808

Thermal correction to Gibbs Free Energy = 0.418974

Ni	-0.60562100	-0.97921400	-0.08794400
C	-2.43723200	-1.44159400	-0.08983200
C	-0.94945400	0.33941400	1.12560800
P	1.60316100	-0.61182500	-0.08381000
C	2.04348000	1.15160300	-0.37152300
C	2.31628900	-1.03716100	1.56514200
C	2.58115100	-1.55501500	-1.33315000
C	3.54614200	-0.26950500	2.05318600
C	4.05898800	-0.80358000	3.38706000
C	4.09444500	-1.34810500	-1.37729100
C	4.75384000	-2.11501000	-2.51977200
C	1.58424900	1.66140800	-1.73827400

O	-0.77080100	0.14295000	2.30566400
C	-3.05774100	-1.60392200	-1.34410800
C	-4.32628700	-2.17802300	-1.46478900
C	-5.01362700	-2.59183500	-0.32537300
C	-3.15895000	-1.85617800	1.04323100
C	-4.42580400	-2.42812000	0.93030700
H	2.52007700	-2.12162000	1.53238500
H	1.48611100	-0.90485500	2.27883500
H	4.35304000	-0.31141400	1.30429300
H	3.28876900	0.79598600	2.16418900
H	2.13333000	-1.30678400	-2.31089300
H	2.34482300	-2.61939500	-1.16268800
H	4.32084600	-0.27389500	-1.47979300
H	4.53813400	-1.66665200	-0.42032700
H	3.12450800	1.31313200	-0.23487900
H	1.53553600	1.71759900	0.42708500
H	2.20904900	1.21028500	-2.52831800
H	0.55252700	1.32021500	-1.92552300
H	4.36827900	-1.85710200	3.30029000
H	3.27710400	-0.75004100	4.16014400
H	4.35445600	-1.79508800	-3.49493300
H	4.57549900	-3.19756500	-2.42622300
H	-2.54793700	-1.28218500	-2.26019800
H	-4.78194900	-2.29705500	-2.45168500
H	-2.71733200	-1.72355100	2.03577400
H	-4.96283700	-2.74598400	1.82819100
C	-1.28858200	1.71586300	0.60867200
C	-1.84786400	1.91672400	-0.65549100
C	-0.99986100	2.82007400	1.42027700
H	-2.09422700	1.04878300	-1.27062000
C	-1.24900900	4.11084300	0.96245200
H	-0.57779500	2.64201100	2.41185400
C	-1.80473700	4.30581000	-0.30357800
H	-1.01477100	4.96977800	1.59538800
H	-2.00359500	5.31817300	-0.66262800
C	-2.10983200	3.20822900	-1.10847500
H	-2.55819800	3.35896200	-2.09272400
H	4.92569300	-0.22694400	3.74242800
H	5.84179500	-1.95404900	-2.53341500
H	-6.00762900	-3.03707400	-0.41462700
C	1.63421400	3.18147900	-1.83889900
H	2.65063700	3.56354000	-1.65322500
H	1.32191300	3.52545300	-2.83599700
H	0.95762500	3.63869000	-1.10018800

[G-H]‡:

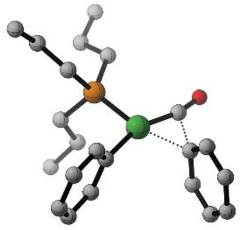

E_{el} = -2781.530024

Zero-point correction = 0.479046

Thermal correction to Energy = 0.507692

Thermal correction to Enthalpy = 0.508636

Thermal correction to Gibbs Free Energy = 0.415508

Ni	0.57188900	-0.50481200	-0.31179400
C	2.36252100	-1.55359500	-0.42737200
P	-1.54462500	-0.14123800	-0.16978700
C	-1.95192100	0.54998300	1.48199500
C	-2.12496500	1.12678700	-1.36784600
C	-2.63774500	-1.61316100	-0.40243700
C	-3.61752800	1.44835000	-1.42296000
C	-3.92054100	2.60448300	-2.37150600
C	-4.00154800	-1.62516600	0.29026400
C	-4.81331900	-2.86790700	-0.06159000
C	-1.43131500	-0.31285700	2.63226100
C	3.03003200	-1.64270900	0.80084900
C	4.40171700	-1.41950700	0.87134100
C	5.11387500	-1.11000800	-0.28839500
C	3.08706100	-1.25389100	-1.58752800
C	4.45965600	-1.02932900	-1.51774700
H	-1.76785300	0.79617500	-2.35779600
H	-1.53563600	2.02668100	-1.12601600
H	-4.17856000	0.55635500	-1.74634400
H	-3.98704500	1.69690300	-0.41430800
H	-2.04568700	-2.48730100	-0.08773200
H	-2.75509300	-1.72469500	-1.49480000
H	-3.85691700	-1.58296400	1.38188300
H	-4.57589100	-0.72331200	0.02712900
H	-3.03808000	0.71250100	1.57300400
H	-1.46317600	1.53739000	1.50683300
H	-1.91807700	-1.30280600	2.61071700
H	-0.35601200	-0.50203200	2.47616400
H	-3.58033200	2.37785800	-3.39406900
H	-3.41089300	3.52543200	-2.04897000
H	-4.27446400	-3.78595100	0.21995200
H	-5.01170900	-2.92015800	-1.14358200

H	2.46834100	-1.88249400	1.70803700
H	4.91832900	-1.48147300	1.83140700
H	2.57115900	-1.18772700	-2.54920000
H	5.02074700	-0.78529500	-2.42230900
H	-4.99974100	2.81267700	-2.41569800
H	-5.78240400	-2.87305700	0.45872600
H	6.18989000	-0.93040500	-0.23244500
C	1.07574000	1.35190000	0.01623700
C	1.19372900	2.29523700	-1.02280200
C	1.39638500	1.81951600	1.30563400
C	1.59771600	3.61361800	-0.79731000
H	0.96497000	1.99875600	-2.05377100
C	1.79808900	3.13499800	1.54900400
H	1.33991900	1.13545100	2.16107600
C	1.89817900	4.04266900	0.49507200
H	1.67742600	4.31230500	-1.63533600
H	2.03682300	3.45335000	2.56803700
H	2.20984700	5.07389900	0.67839200
C	0.79517900	-2.23176900	-0.55751800
O	0.62467600	-3.38973100	-0.68893200
C	-1.64456400	0.33822800	3.99384500
H	-2.71323900	0.52471100	4.18442300
H	-1.26539500	-0.29990800	4.80552500
H	-1.12076500	1.30504400	4.05062100

H:

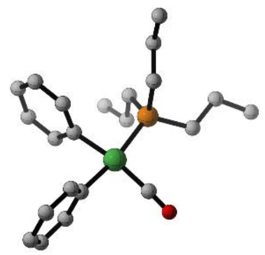

E_{el} = -2781.551455

Zero-point correction = 0.479567

Thermal correction to Energy = 0.508759

Thermal correction to Enthalpy = 0.509703

Thermal correction to Gibbs Free Energy = 0.416736

Ni	-0.70178000	-0.77282600	-0.01378900
C	-2.62381700	-0.84285100	0.07941200
P	1.51151600	-0.37147800	0.05902700
C	2.07557000	1.07842300	-0.91684600
C	2.01384700	-0.04239200	1.81030500
C	2.61482200	-1.74751000	-0.48466300

C	3.08656100	1.01840900	2.06017300
C	3.41522200	1.16199300	3.54249500
C	4.12354300	-1.53831000	-0.36856500
C	4.92054000	-2.73751200	-0.87290700
C	1.86242400	0.89155300	-2.41977800
C	-3.41520200	-0.86953000	-1.07643400
C	-4.80720600	-0.93917600	-0.99745300
C	-5.43895100	-0.99055300	0.24427200
C	-3.27341600	-0.90046100	1.31974700
C	-4.66475700	-0.97130400	1.40390700
H	2.32359900	-1.01575400	2.22824400
H	1.09659400	0.24091200	2.34738200
H	4.00430700	0.77491100	1.50047200
H	2.73311200	1.98548800	1.66863500
H	2.33509200	-1.97161900	-1.52849700
H	2.30606700	-2.63160400	0.09935000
H	4.42010300	-0.63806100	-0.93192600
H	4.38509300	-1.34155300	0.68417300
H	3.13257200	1.29707600	-0.69926200
H	1.48243000	1.93332600	-0.55582200
H	2.57238700	0.13954200	-2.80492900
H	0.85396200	0.48341500	-2.59870300
H	3.80502700	0.21982700	3.95926100
H	2.51964600	1.43702400	4.12125800
H	4.71502000	-2.93144600	-1.93719800
H	4.66278100	-3.65029600	-0.31335700
H	-2.94533600	-0.82367500	-2.06395600
H	-5.40214000	-0.95253700	-1.91474800
H	-2.69053100	-0.87611700	2.24596400
H	-5.14793500	-1.00925000	2.38388400
H	4.17377400	1.94108000	3.70807700
H	6.00238300	-2.57180600	-0.76464100
H	-6.52815900	-1.04510300	0.30882100
C	-0.99407600	1.12295400	0.03627800
C	-0.90358400	1.86843300	1.22185200
C	-1.22102000	1.84035400	-1.15011800
C	-0.98011300	3.26373200	1.22028300
H	-0.78091100	1.35809800	2.18281200
C	-1.29782700	3.23320500	-1.16166100
H	-1.34745300	1.30499500	-2.09621300
C	-1.16414700	3.95575100	0.02478300
H	-0.90178600	3.81170100	2.16325500
H	-1.47176200	3.75833000	-2.10493000
H	-1.22455400	5.04646300	0.01920600
C	-0.70883600	-2.56400600	-0.08626800

O	-0.76924600	-3.69953600	-0.13397600
C	2.02349100	2.19643200	-3.19082700
H	3.02444800	2.63032700	-3.03873700
H	1.88366200	2.04249700	-4.27090600
H	1.28115600	2.93559600	-2.85239300

[H-A]‡:

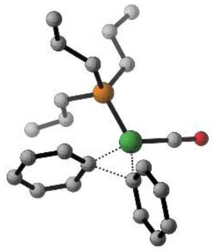

E_{el} = -2781.539661

Zero-point correction = 0.478639

Thermal correction to Energy = 0.507165

Thermal correction to Enthalpy = 0.508109

Thermal correction to Gibbs Free Energy = 0.417874

Ni	-0.72254200	-0.84456100	-0.36022900
C	-2.57558000	-0.44795900	0.03824000
P	1.44484100	-0.41713300	0.09341600
C	2.07176700	1.16244400	-0.61394400
C	1.69225100	-0.25899800	1.92569700
C	2.68853000	-1.68012500	-0.42376400
C	2.70087000	0.76673500	2.44350400
C	2.83001300	0.73052800	3.96289200
C	4.16225700	-1.42230400	-0.11781300
C	5.07015900	-2.53393600	-0.63569600
C	2.10773900	1.15289800	-2.14219200
C	-3.62270600	-0.34734800	-0.89411300
C	-4.93100800	-0.66574400	-0.54447200
C	-5.23676500	-1.09063000	0.74993500
C	-2.90350800	-0.86283000	1.34340900
C	-4.21293800	-1.18886100	1.69097600
H	1.94923400	-1.27085300	2.28412200
H	0.70257900	-0.03204700	2.35148000
H	3.68906700	0.59879600	1.98560200
H	2.38403000	1.77342800	2.12698900
H	2.54012500	-1.82152500	-1.50821000
H	2.35713600	-2.62583300	0.03908200
H	4.47113600	-0.46159900	-0.56223100
H	4.30083100	-1.31459700	0.97053300
H	3.06704400	1.40222300	-0.20762400

H	1.38118800	1.94544700	-0.26009300
H	2.85710200	0.42301400	-2.49389600
H	1.13493500	0.80409600	-2.52660100
H	3.18767500	-0.25181000	4.31015800
H	1.85958800	0.92028400	4.44789600
H	4.96891600	-2.65247700	-1.72586300
H	4.81761600	-3.50081800	-0.17308600
H	-3.41187600	-0.00550500	-1.91005200
H	-5.72309300	-0.58054100	-1.29269800
H	-2.12082100	-0.93436500	2.10536400
H	-4.43456200	-1.51605300	2.71002900
H	3.53898000	1.49113000	4.32184800
H	6.12767900	-2.32379900	-0.41760200
H	-6.26585100	-1.33337900	1.02300000
C	-1.27939700	0.98112500	-0.07153400
C	-1.14175800	1.73433900	1.11238300
C	-1.26576100	1.69679600	-1.28849200
C	-0.93713100	3.11023700	1.07924400
H	-1.21113500	1.23347900	2.08120100
C	-1.06122200	3.07441200	-1.32107100
H	-1.41575200	1.16520700	-2.23293300
C	-0.88402300	3.79292300	-0.13834500
H	-0.82633600	3.65839300	2.01841500
H	-1.04697000	3.59280700	-2.28332900
H	-0.73086100	4.87365300	-0.16281600
C	-0.79619600	-2.50585500	-1.03722900
O	-0.81074300	-3.54463600	-1.51266500
C	2.42237100	2.52695800	-2.72198200
H	3.39854800	2.89516100	-2.36810400
H	2.45115500	2.50184300	-3.82145700
H	1.65944300	3.25918200	-2.41642700

[B-C’]‡:

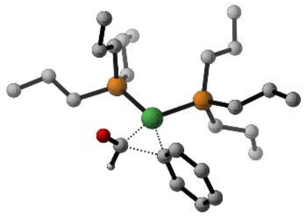

E_{el} = -3247.415143

Zero-point correction = 0.687562

Thermal correction to Energy = 0.726180

Thermal correction to Enthalpy = 0.727124

Thermal correction to Gibbs Free Energy = 0.615437

Ni	-0.15018200	-0.92672900	-0.26932100
P	1.30073800	0.73897100	0.10250000
P	-2.13943000	-0.03905700	-0.24104900
C	-2.34326800	1.61305700	-1.08166900
C	2.81978500	0.18567300	0.99831600
C	1.87455800	1.33349400	-1.55852400
C	0.72587900	2.25484700	1.00957100
C	-3.52901600	-1.04546900	-0.91644200
C	-2.63140400	0.23852500	1.52102500
C	-3.15543500	2.70294100	-0.38173500
C	-3.19461600	3.99972200	-1.18400000
C	-4.95139600	-0.51527600	-0.75560500
C	-2.77502000	-1.08059900	2.28715300
C	-2.85213800	-0.87437000	3.79535800
C	3.25110400	1.97600200	-1.72529900
C	3.53343300	2.35986900	-3.17468500
C	1.38819400	3.60932400	0.75785100
C	0.73205700	4.72801000	1.56147700
C	3.79180700	1.19080700	1.61448200
C	4.96768300	0.49513900	2.29461300
H	-2.77291100	1.40067800	-2.07589000
H	-1.32627400	1.99019800	-1.27633800
H	-4.18260000	2.34953900	-0.19671100
H	-2.72277200	2.90489700	0.61231900
H	-3.65335500	3.84321400	-2.17297900
H	-2.17898400	4.39425800	-1.34856200
H	-3.41615400	-2.03596800	-0.44392500
H	-3.29374000	-1.20323600	-1.98332400
C	-5.99744500	-1.47757500	-1.31128500
H	-5.16006800	-0.32882600	0.31116400
H	-5.04711400	0.46059700	-1.26150400
H	-3.55834800	0.82987400	1.59487900
H	-1.82895500	0.84750600	1.96972600
H	-3.68479100	-1.59949500	1.94244800
H	-1.94545500	-1.76057700	2.03245700
H	1.08298200	2.00960200	-1.92771000
H	1.81837000	0.44009600	-2.20240600
H	3.33938200	2.86727400	-1.08558300
H	4.02277500	1.26907500	-1.38007500
H	0.77595100	1.99440900	2.08207700
H	-0.34971000	2.34278700	0.78625200
H	2.46127700	3.56090700	0.99796200
H	1.32516900	3.85001600	-0.31628300
H	3.33833700	-0.48743400	0.29256800
H	2.44490300	-0.48665800	1.78771000

H	4.17078800	1.88596200	0.85027100
H	3.25987000	1.81144900	2.35495300
H	-3.68999900	-0.21335800	4.07069100
H	-1.92762000	-0.41509100	4.18058900
H	2.79498600	3.08943800	-3.54356100
H	3.48735700	1.47937800	-3.83479400
H	5.54029400	-0.10799300	1.57272600
H	4.62062500	-0.18563900	3.08774600
H	0.80642700	4.53573800	2.64339900
H	-0.33777500	4.82011700	1.31433800
H	-5.94511000	-2.45199400	-0.80155100
H	-5.83840500	-1.65813500	-2.38598800
H	5.65744600	1.22118500	2.75042700
H	4.53153000	2.80956700	-3.28431900
H	1.20737900	5.69969500	1.36118100
H	-3.77490500	4.77650500	-0.66409900
H	-2.99241400	-1.83118100	4.31952600
H	-7.01632900	-1.08234600	-1.18318600
C	1.10495300	-2.44749900	-0.43780500
C	1.69319400	-3.00696500	0.71039200
C	1.84685800	-2.46132300	-1.62975200
C	3.00195300	-3.47318300	0.69117400
H	1.08962500	-3.08534500	1.61908300
C	3.16418200	-2.92484600	-1.65639300
H	1.38604600	-2.09471700	-2.55344500
C	3.74853600	-3.41932900	-0.49143700
H	3.44724000	-3.89374700	1.59638300
H	3.73096600	-2.91163000	-2.59089700
H	4.77546700	-3.79221700	-0.50862500
C	-0.69234800	-2.69434100	-0.59017000
H	-0.86873300	-2.92788800	-1.68061300
O	-1.24371600	-3.38507500	0.26388000

C’:

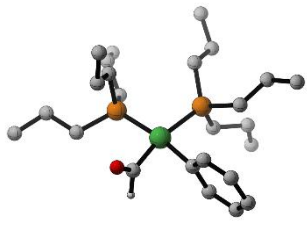

E_{el} = -3247.420807

Zero-point correction = 0.687876

Thermal correction to Energy = 0.727213

Thermal correction to Enthalpy = 0.728157

Thermal correction to Gibbs Free Energy = 0.615052

Ni	0.06516100	-1.05394500	0.31597400
P	-1.21760700	0.76917800	-0.11614900
P	2.08947600	-0.15322800	0.23486800
C	2.32056100	1.53104200	1.00122000
C	-2.78454700	0.39321000	-1.01847100
C	-1.71115800	1.40649700	1.55273900
C	-0.51010400	2.21988900	-1.04075800
C	3.47447700	-1.12642500	0.96654300
C	2.57751900	0.01496700	-1.54056000
C	3.14954400	2.57086600	0.24645200
C	3.20756900	3.90614800	0.98086600
C	4.89172800	-0.57952000	0.81184600
C	2.75102000	-1.34923600	-2.21794500
C	2.80964100	-1.23759600	-3.73687700
C	-3.02355300	2.16688300	1.73644800
C	-3.26051600	2.55342200	3.19315500
C	-0.95794800	3.64000100	-0.69622200
C	-0.19264900	4.69042200	-1.49549600
C	-3.61527300	1.52430100	-1.62247000
C	-4.85239700	0.99050500	-2.33938800
H	2.75782600	1.35200300	1.99834000
H	1.31696200	1.94067600	1.19326100
H	4.17050600	2.19191000	0.08003400
H	2.72160100	2.72912900	-0.75760400
H	3.66160200	3.79373600	1.97787800
H	2.19800900	4.32504200	1.12108700
H	3.39259800	-2.13156600	0.52344700
H	3.22294400	-1.25629200	2.03333100
C	5.94278400	-1.52749500	1.38243300
H	5.10624000	-0.39890900	-0.25486800
H	4.97487200	0.40003000	1.31191200
H	3.49229400	0.61896100	-1.65089500
H	1.76503000	0.57451500	-2.03236300
H	3.67866600	-1.81825500	-1.85014200
H	1.94269900	-2.03395400	-1.91227000
H	-0.85938900	2.00503800	1.92021700
H	-1.73140400	0.50648700	2.18781100
H	-3.03748300	3.07233000	1.11019200
H	-3.85620400	1.53467700	1.38877400
H	-0.68978000	2.00946800	-2.10961000
H	0.58053100	2.16113100	-0.91881400
H	-2.03820000	3.75395800	-0.87408800
H	-0.80469500	3.82142100	0.38056600
H	-3.38857700	-0.21841900	-0.32797300

H	-2.48557400	-0.31198900	-1.81133600
H	-3.92451500	2.23633300	-0.84250900
H	-3.00327000	2.10058700	-2.33620000
H	3.62740600	-0.57403000	-4.06171000
H	1.86983800	-0.82801400	-4.14045300
H	-2.46660500	3.22205500	3.56225200
H	-3.27418700	1.66368300	3.84206400
H	-5.50183900	0.43496400	-1.64526200
H	-4.57215600	0.29899100	-3.14919600
H	-0.34813300	4.55683300	-2.57751600
H	0.89039800	4.62038100	-1.30510100
H	5.90867900	-2.50425600	0.87584300
H	5.77484000	-1.70567400	2.45603500
H	-5.44507200	1.80581700	-2.78027800
H	-4.22163900	3.07378700	3.31774700
H	-0.51388200	5.70934700	-1.23356300
H	3.80175200	4.64441800	0.42238200
H	2.97000400	-2.22159700	-4.20123300
H	6.95751200	-1.11945600	1.26320400
C	-1.52355000	-2.14246800	0.38940500
C	-2.00440600	-2.80849100	-0.75184700
C	-2.31983700	-2.21276400	1.54524500
C	-3.24192700	-3.45022800	-0.76153000
H	-1.39257100	-2.82735900	-1.65982700
C	-3.56381300	-2.85042400	1.54588100
H	-1.96771500	-1.75753100	2.47813600
C	-4.03751700	-3.46155700	0.38617700
H	-3.58931800	-3.95094000	-1.66958600
H	-4.16271200	-2.87558100	2.46085500
H	-5.00876000	-3.96204000	0.38150100
C	0.75458300	-2.73932400	0.74625200
H	0.69198500	-3.03847900	1.83809700
O	1.27812000	-3.51099000	-0.02865400

[C-J]‡:

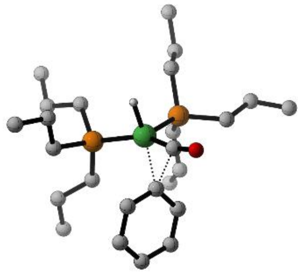

E_{el} = -3247.409769

Zero-point correction = 0.684176

Thermal correction to Energy = 0.723097

Thermal correction to Enthalpy = 0.724041

Thermal correction to Gibbs Free Energy = 0.612091

Ni	0.00802700	-0.41246600	-1.25189200
P	-1.93537700	0.14405200	-0.17761100
C	-2.51259900	1.76196300	-0.88422100
C	-3.35189100	-1.00561300	-0.48565500
C	-2.01077900	0.41588000	1.65601600
C	-4.75329600	-0.59602500	-0.03883400
C	-5.79350600	-1.68002400	-0.30631200
C	-1.64185000	-0.82678000	2.46454300
C	-1.48622600	-0.54124900	3.95399800
C	-3.34257900	2.71421300	-0.02379800
C	-3.75306000	3.96798100	-0.79073600
H	-3.07140300	-1.96606900	-0.01974200
H	-3.33075500	-1.19939200	-1.57140800
H	-4.74849100	-0.35295100	1.03730400
H	-5.04945000	0.32842800	-0.56116600
H	-2.99817900	0.79665600	1.96448100
H	-1.28955300	1.22157800	1.87654400
H	-2.41202200	-1.60360600	2.31801400
H	-0.70538100	-1.25125700	2.07015300
H	-3.05547000	1.49578600	-1.80750800
H	-1.60137300	2.27266800	-1.23228200
H	-4.24161300	2.20647400	0.36005100
H	-2.76114400	3.00962800	0.86565500
H	-5.54391700	-2.61077300	0.22712200
H	-5.84864900	-1.91912800	-1.37973200
H	-4.36952300	3.71275800	-1.66709600
H	-2.86941800	4.51372400	-1.15746900
H	-2.41200500	-0.12724200	4.38392400
H	-0.68256500	0.19157300	4.13299100
C	-0.35004500	-1.78170400	-2.23669000
O	-0.77250100	-2.56231600	-3.00044900
H	-4.33561700	4.65447300	-0.15837700
H	-6.79591600	-1.36445700	0.01973300
H	-1.23416200	-1.45482900	4.51269100
C	0.64332700	-2.36551000	-0.81932000
C	2.01471300	-2.55805100	-1.03710000
C	0.02884000	-3.08737500	0.21188000
C	2.76782900	-3.37947500	-0.19788100
H	2.49776400	-2.04620800	-1.87425700
C	0.77361900	-3.91221700	1.05386700
H	-1.04764300	-2.99007300	0.36926000
C	2.14924600	-4.04773400	0.85923300

H	3.83955500	-3.50369900	-0.37135100
H	0.28046200	-4.44886200	1.86820100
H	2.73630700	-4.68953100	1.52022900
P	1.49266700	0.79657800	-0.12867400
C	0.99772300	2.55036800	0.17824100
H	0.82949800	2.98415300	-0.82206100
H	-0.00067900	2.50255500	0.64393600
C	1.84358200	0.08574800	1.54878300
H	1.65340300	-0.99511500	1.44344800
H	1.04603300	0.46527200	2.21161200
C	3.16266500	0.95006500	-0.90010900
H	3.82465700	1.54405300	-0.24871500
H	3.58159400	-0.06983600	-0.92482500
C	1.91070500	3.45197300	1.00487800
H	2.91581900	3.48485400	0.55284500
H	2.03970900	3.02271400	2.01176100
C	1.36312300	4.87097900	1.12770400
H	2.02582600	5.50805100	1.73203700
H	1.25425100	5.34159400	0.13796000
H	0.37039300	4.87198700	1.60514800
C	3.13216900	1.53623700	-2.31020600
H	2.69865000	2.55058400	-2.27915300
H	2.44696600	0.93782100	-2.93104300
C	4.51514900	1.59799500	-2.94984300
H	4.95861300	0.59334000	-3.03637600
H	4.46699300	2.02935500	-3.96064300
H	5.20644600	2.21543700	-2.35407000
C	3.21335100	0.28541500	2.19596100
H	3.99221900	-0.13003800	1.53699900
H	3.44261300	1.35795400	2.30118600
C	3.29758500	-0.39545600	3.55874900
H	3.11025100	-1.47682500	3.46592900
H	4.28882100	-0.26163800	4.01708400
H	2.54730100	0.01183700	4.25533100
H	0.03991600	0.50473900	-2.43240000

J:

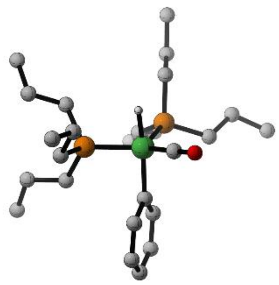

E_{el} = -3247.421804

Zero-point correction = 0.685187

Thermal correction to Energy = 0.724798

Thermal correction to Enthalpy = 0.725742

Thermal correction to Gibbs Free Energy = 0.611266

Ni	-0.16502400	-0.50629300	1.13048500
P	1.85332600	0.17735800	0.28697300
C	2.42653400	1.77244600	1.03661600
C	3.21388900	-1.04004200	0.61457000
C	1.86548600	0.50340100	-1.54226700
C	4.67793500	-0.60035600	0.63911000
C	5.61975300	-1.76557100	0.92928000
C	3.15935000	0.40745800	-2.34959700
C	2.92551400	0.65784000	-3.83658700
C	3.47058000	2.63999100	0.33395700
C	3.78883500	3.90700300	1.12304000
H	3.06823100	-1.85747300	-0.11164100
H	2.95105000	-1.48239100	1.58948500
H	4.95796600	-0.13529200	-0.31805200
H	4.81370000	0.17610100	1.40985000
H	1.41156300	1.50372700	-1.66052600
H	1.13040100	-0.20020400	-1.96346000
H	3.90444700	1.12297900	-1.96943700
H	3.59937000	-0.59466000	-2.21549800
H	2.75097400	1.51586600	2.06050200
H	1.50234600	2.35637000	1.17359000
H	4.39814400	2.07333700	0.16481300
H	3.09779900	2.92167500	-0.66474400
H	5.53973500	-2.54285500	0.15308800
H	5.38240900	-2.23812800	1.89521700
H	4.21533600	3.66486800	2.10910300
H	2.88050400	4.50611200	1.29404000
H	2.51231100	1.66426500	-4.00953200
H	2.21085500	-0.06979200	-4.25269300
C	-0.10419300	-1.31847600	2.70446400
O	-0.04077300	-1.82994100	3.72608000
H	4.51383800	4.54007400	0.59018700
H	6.66829500	-1.43421200	0.96608600
H	3.86112400	0.57590300	-4.40937400
C	-0.50902300	-2.18929500	0.15503500
C	-1.67061400	-2.92753100	0.46342100
C	0.26220200	-2.69648200	-0.90528100
C	-2.05486100	-4.06257000	-0.25264000
H	-2.31074800	-2.60228900	1.29059100
C	-0.10726300	-3.83153800	-1.63165200

H	1.18899600	-2.19804500	-1.19948400
C	-1.27581600	-4.51996600	-1.31511400
H	-2.96949800	-4.59483900	0.02383500
H	0.52702300	-4.17919500	-2.45216400
H	-1.57076500	-5.40707400	-1.88066100
P	-1.75682800	0.55576600	-0.00115000
C	-1.43835900	2.36707300	-0.26010900
H	-0.81461500	2.68478500	0.58975000
H	-0.78478400	2.42466400	-1.14919600
C	-2.07443600	-0.09748600	-1.69822200
H	-2.41034000	-1.13636800	-1.54618600
H	-1.08226100	-0.19135000	-2.16945100
C	-3.42137700	0.47100800	0.78863800
H	-4.14243400	1.07888900	0.21761300
H	-3.73895700	-0.58004200	0.68263700
C	-2.62081500	3.32520000	-0.40780500
H	-3.20672000	3.32542700	0.52578100
H	-3.30736200	2.97767900	-1.19460500
C	-2.17104000	4.74932600	-0.71996900
H	-3.02678700	5.43815200	-0.77874100
H	-1.48737600	5.12876900	0.05589800
H	-1.63725900	4.79530600	-1.68254300
C	-3.42358400	0.87120700	2.26281300
H	-3.00933100	1.88794900	2.37583800
H	-2.73240100	0.21090900	2.80947100
C	-4.81332200	0.81178500	2.88683400
H	-5.24242900	-0.19916800	2.80245000
H	-4.78329100	1.07432500	3.95473100
H	-5.50695100	1.50811600	2.38900600
C	-3.02754800	0.64865300	-2.62804300
H	-4.00433000	0.79274200	-2.13650200
H	-2.63359400	1.65764100	-2.83370100
C	-3.23129900	-0.08761000	-3.94926200
H	-3.66771700	-1.08468800	-3.78321300
H	-3.90228600	0.46898800	-4.62045400
H	-2.27277600	-0.23107800	-4.47235900
H	-0.14119900	0.72959200	1.95370900

K:

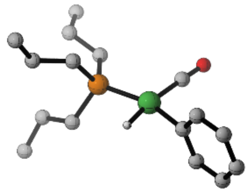

E_{el} = -2550.587346

Zero-point correction = 0.395601

Thermal correction to Energy = 0.419961

Thermal correction to Enthalpy = 0.420905

Thermal correction to Gibbs Free Energy = 0.339081

Ni	1.08576500	-0.27708800	-0.01227900
P	-1.10664800	-0.01924900	-0.01415800
C	-1.66664300	0.79845900	-1.57459700
C	-2.12349300	-1.55880900	0.15146100
C	-1.60070100	1.08539400	1.38220600
C	-3.58954700	-1.57437600	-0.28294600
C	-4.24557400	-2.92985300	-0.03648300
C	-3.04033200	1.08517000	1.89614000
C	-3.23791000	2.08531400	3.03162300
C	-2.95428600	1.62435100	-1.57365900
C	-3.23331800	2.24587800	-2.93886700
H	-2.02750100	-1.85914200	1.21026200
H	-1.57142800	-2.32623900	-0.41626100
H	-4.15701100	-0.79274300	0.24350100
H	-3.65310200	-1.32989300	-1.35598400
H	-1.29141900	2.09650900	1.06694400
H	-0.91542000	0.82509600	2.20577800
H	-3.74111300	1.31687200	1.07963500
H	-3.30360500	0.07518100	2.25110100
H	-1.71841100	-0.00137000	-2.33389600
H	-0.82184400	1.43852600	-1.87466100
H	-3.81087800	1.00276100	-1.27142400
H	-2.87334400	2.42449200	-0.81994700
H	-4.24088100	-3.18308900	1.03535500
H	-3.71335400	-3.73334800	-0.56942000
H	-3.34748400	1.47051300	-3.71269400
H	-2.40986300	2.90711000	-3.25033900
H	-3.02998100	3.11315600	2.69568000
H	-2.56147000	1.86883800	3.87318200
C	1.40294900	-2.04462700	-0.02883300
O	1.64436600	-3.15772200	-0.04271600
H	-4.15691000	2.84315800	-2.92408000
H	-5.29120400	-2.93558700	-0.37784700
H	-4.26898100	2.05839100	3.41375500
H	1.08461100	1.19454100	0.00082700
C	2.94888400	0.18936100	0.00199800
C	3.67334800	0.33589700	-1.19115500
C	3.65343600	0.33705200	1.20685500
C	5.04196300	0.61272700	-1.18272500
H	3.16523500	0.23509000	-2.15514100

C	5.02200400	0.61352800	1.22095300
H	3.12943200	0.23805800	2.16246600
C	5.72474500	0.75271300	0.02479300
H	5.57800000	0.72119600	-2.12972400
H	5.54244000	0.72293700	2.17651000
H	6.79573400	0.96888700	0.03374100

[K-L]‡:

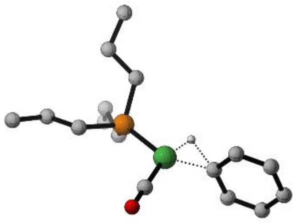

E_{el} = -2550.585094

Zero-point correction = 0.394363

Thermal correction to Energy = 0.418500

Thermal correction to Enthalpy = 0.419445

Thermal correction to Gibbs Free Energy = 0.338133

Ni	1.09374200	0.44508900	0.01837200
P	-1.08685800	0.05080300	0.01395400
C	-1.62638000	-0.72358100	1.60654000
C	-2.18928800	1.51816000	-0.24137000
C	-1.51178700	-1.15103400	-1.32756500
C	-3.65778900	1.47517200	0.18214600
C	-4.38737200	2.77542800	-0.14254100
C	-2.94408200	-1.26168200	-1.84980200
C	-3.07326800	-2.31966700	-2.94182900
C	-2.87131000	-1.61189300	1.63697600
C	-3.12871600	-2.18791300	3.02623800
H	-2.10031700	1.76330000	-1.31493600
H	-1.68515400	2.34652400	0.28370300
H	-4.17470700	0.63462800	-0.30431900
H	-3.71825900	1.28590700	1.26652000
H	-1.14697700	-2.12609100	-0.96070500
H	-0.83778500	-0.89285700	-2.16116000
H	-3.63658800	-1.49937900	-1.02810000
H	-3.26491400	-0.28567300	-2.24973800
H	-1.72289100	0.10606600	2.32873100
H	-0.75488300	-1.30720500	1.94448100
H	-3.75564000	-1.04803300	1.30339700
H	-2.74549500	-2.43905400	0.91952300
H	-4.38462300	2.97164600	-1.22623800
H	-3.90803600	3.63531600	0.35102700

H	-3.29322400	-1.38793500	3.76515800
H	-2.27230400	-2.78751900	3.37212700
H	-2.80361100	-3.31711800	-2.56092600
H	-2.40727500	-2.09798000	-3.79040200
C	1.36946400	2.21844600	0.02397400
O	1.55315600	3.34485200	0.03285900
H	-4.01688100	-2.83696800	3.03131600
H	-5.43558400	2.73891900	0.18900400
H	-4.10199100	-2.37433700	-3.32743500
H	1.34842900	-1.00121500	0.01974100
C	2.89657600	-0.22290200	0.00332200
C	3.62360800	-0.38906800	1.19414800
C	3.58969700	-0.39588500	-1.20673600
C	4.98825500	-0.67972500	1.17669700
H	3.11820500	-0.28594600	2.15871800
C	4.95423600	-0.68608900	-1.22671100
H	3.05730000	-0.29911300	-2.15741400
C	5.66324300	-0.82797600	-0.03438400
H	5.52749800	-0.79466200	2.12091400
H	5.46674900	-0.80619200	-2.18507400
H	6.73128200	-1.05712900	-0.04912900

L:

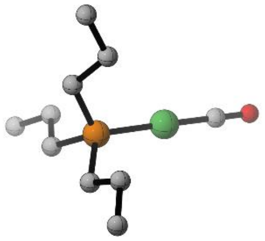

E_{el} = -2318.469049

Zero-point correction = 0.295680

Thermal correction to Energy = 0.314782

Thermal correction to Enthalpy = 0.315726

Thermal correction to Gibbs Free Energy = 0.245231

Ni	-1.10698600	1.26270200	-0.01153600
C	-2.15838800	2.61770100	0.16300400
P	0.21680800	-0.50988800	-0.27697600
O	-2.86248900	3.51867600	0.27850900
C	1.57944100	-0.32059200	-1.50988000
H	2.09667000	-1.29033400	-1.61627500
H	1.08361200	-0.11649500	-2.47410200
C	1.06864300	-1.12143500	1.24772900
H	0.35894700	-1.80045500	1.74941300
H	1.93200900	-1.73983800	0.94444700

C	-0.60853900	-2.03696400	-0.90727800
H	0.11916500	-2.86716300	-0.88533900
H	-0.84236800	-1.84737600	-1.96832900
C	2.58122500	0.79055600	-1.19817800
H	2.03626000	1.73062700	-1.00862500
H	3.11598000	0.55277400	-0.26396500
C	3.59540300	0.99452500	-2.31904400
H	3.09667000	1.28484900	-3.25688400
H	4.31733100	1.78413300	-2.06456900
H	4.16247100	0.07091200	-2.51563600
C	1.49351500	-0.02431400	2.22573900
H	2.16855100	0.68762600	1.72429000
H	0.60377000	0.56191800	2.50972300
C	2.17243400	-0.58239200	3.47164800
H	3.08591800	-1.14168400	3.21375500
H	2.45823900	0.22270200	4.16430900
H	1.50448200	-1.26910900	4.01521500
C	-1.88531600	-2.41136500	-0.15445900
H	-2.58879900	-1.56345800	-0.19789900
H	-1.66059200	-2.56065600	0.91523100
C	-2.54657700	-3.66758600	-0.71201900
H	-3.46824100	-3.91038000	-0.16317000
H	-2.81298600	-3.53630600	-1.77246500
H	-1.87475700	-4.53784000	-0.64311400

[C-K]‡:

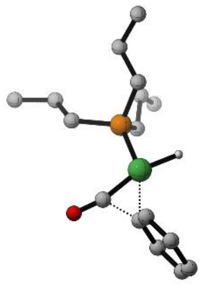

E_{el} = -2550.561624

Zero-point correction = 0.395656

Thermal correction to Energy = 0.419001

Thermal correction to Enthalpy = 0.419945

Thermal correction to Gibbs Free Energy = 0.341359

Ni	1.02784700	-0.25885900	-0.03202600
P	-1.09898700	-0.12216300	-0.03006000
C	-1.74516300	-0.61535800	1.62799900
C	-1.77194000	1.56344900	-0.38083900
C	-1.84366500	-1.26412500	-1.27272900

C	-3.17678400	1.94344100	0.08785700
C	-3.53814600	3.37610900	-0.29326200
C	-3.27377500	-1.02991300	-1.75753700
C	-3.71595300	-2.08866000	-2.76355300
C	-3.17128400	-1.15490900	1.74975900
C	-3.52134400	-1.51505400	3.19054100
H	-1.67405700	1.69679000	-1.47285900
H	-1.03402200	2.25682800	0.05512600
H	-3.92535800	1.25309700	-0.33027200
H	-3.23740700	1.83359500	1.18319600
H	-1.72227200	-2.27109600	-0.83984900
H	-1.15051200	-1.24060400	-2.12944800
H	-3.97216200	-1.02203300	-0.90685900
H	-3.34648400	-0.03321000	-2.22305700
H	-1.60275000	0.26429600	2.27930900
H	-1.03315700	-1.37563700	1.98629600
H	-3.89679000	-0.41970700	1.36927100
H	-3.27681700	-2.05022400	1.11563100
H	-3.53198800	3.50945500	-1.38646700
H	-2.81772000	4.09288800	0.13068300
H	-3.47109100	-0.63064200	3.84504200
H	-2.82203400	-2.26513500	3.59173700
H	-3.69534300	-3.09387500	-2.31452200
H	-3.05123800	-2.10468900	-3.64146400
C	1.70877500	1.37035900	-0.02105600
O	1.83926200	2.54416900	0.01027300
H	-4.53753400	-1.92977100	3.26414000
H	-4.53956000	3.64689700	0.07273300
H	-4.73941600	-1.89993600	-3.11975400
H	0.92696200	-1.79914100	-0.00055800
C	3.04491500	0.32848000	-0.01104300
C	3.68888000	0.04749300	1.20110800
C	3.68935700	0.03057500	-1.21860000
C	4.95684300	-0.52762600	1.20608200
H	3.19149900	0.28296300	2.14573100
C	4.95765500	-0.54498600	-1.21505400
H	3.19304000	0.25343900	-2.16695800
C	5.58883300	-0.82344700	-0.00247700
H	5.45480300	-0.74803900	2.15276100
H	5.45601000	-0.77845000	-2.15837900
H	6.58316800	-1.27588700	0.00107900

K’:

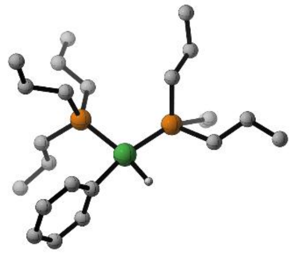

E_{el} = -3134.098494

Zero-point correction = 0.675733

Thermal correction to Energy = 0.713127

Thermal correction to Enthalpy = 0.714072

Thermal correction to Gibbs Free Energy = 0.604582

Ni	-0.17720500	-1.18219600	0.18068600
P	1.96261000	-0.68327700	0.14360500
C	2.44869300	-0.11606700	-1.55553600
C	2.47411500	0.66080500	1.32292500
C	3.08874900	-2.09199400	0.55324300
C	3.64587200	1.57798500	0.97499200
C	3.86316300	2.65853500	2.03010000
C	4.54397600	-1.83862600	0.94673300
C	5.28596100	-3.13573000	1.25731000
C	3.89614500	-0.20530500	-2.03834900
C	4.04967900	0.28805400	-3.47415800
H	2.65265800	0.15564100	2.28799800
H	1.57320300	1.27262800	1.48839000
H	4.56805300	0.98874800	0.85633300
H	3.46147800	2.05604600	-0.00186800
H	3.02331900	-2.77640500	-0.31051900
H	2.56906300	-2.62099200	1.36842500
H	5.07476200	-1.29723300	0.14942700
H	4.57654200	-1.18431700	1.83381400
H	2.07783000	0.91874300	-1.64247800
H	1.80678100	-0.70946900	-2.22848000
H	4.55752200	0.37750900	-1.37923800
H	4.23919700	-1.25054200	-1.97472300
H	4.07825400	2.21405200	3.01444100
H	2.96701400	3.28974200	2.14090000
H	3.75334000	1.34505300	-3.56600300
H	3.41780700	-0.29461000	-4.16272100
H	5.29741400	-3.80551200	0.38305000
H	4.80312700	-3.68036100	2.08370600
H	5.09060500	0.20045000	-3.81917000
H	4.70518300	3.31525900	1.76580500
H	6.32994400	-2.94213900	1.54537200

H	0.19872600	-2.58443000	0.38582900
C	-1.88854300	-2.02297000	0.25242000
C	-2.47685100	-2.60137700	-0.88940200
C	-2.69096100	-1.98848400	1.40946700
C	-3.79576000	-3.05840000	-0.89540100
H	-1.89227200	-2.69359100	-1.81029000
C	-4.01177200	-2.44436100	1.41566800
H	-2.28002900	-1.58799400	2.34262600
C	-4.57934600	-2.97165000	0.25641100
H	-4.21586200	-3.49252400	-1.80767900
H	-4.60080500	-2.38937300	2.33601800
H	-5.61256700	-3.32729200	0.25448000
P	-1.15902500	0.83091200	0.01010700
C	-1.57139700	1.42726000	1.72172400
H	-0.74619300	2.09573600	2.02521800
H	-1.50151500	0.54474200	2.37606900
C	-0.26335100	2.27154700	-0.73952900
H	0.71264000	2.33303600	-0.22928400
H	-0.03470400	1.97905400	-1.77899800
C	-2.75688300	0.83295200	-0.90705600
H	-3.37429900	0.05197600	-0.43679400
H	-3.28408100	1.79075900	-0.77730300
C	-0.94235200	3.63923100	-0.71520600
H	-1.92150100	3.57832600	-1.21788400
H	-1.15127100	3.92718600	0.32839900
C	-0.09772900	4.72265700	-1.37901700
H	-0.59792000	5.70168500	-1.34146400
H	0.87913000	4.82635300	-0.88012500
H	0.09396800	4.48473700	-2.43719300
C	-2.57209500	0.52048700	-2.39221800
H	-1.89107800	-0.34000400	-2.49839300
H	-2.07949900	1.37184000	-2.89386600
C	-3.89243000	0.20843300	-3.08677700
H	-4.60109500	1.04721000	-2.99666400
H	-3.74395700	0.00748600	-4.15832700
H	-4.36062300	-0.67910300	-2.63388100
C	-2.92018000	2.11083200	1.94702200
H	-3.72544800	1.40314000	1.69407300
H	-3.03565800	2.96563000	1.26096200
C	-3.09311100	2.58534600	3.38608900
H	-2.32115700	3.32242900	3.65921300
H	-4.07524500	3.05783800	3.53583100
H	-3.01436200	1.74488800	4.09335200

[K’-A]‡:

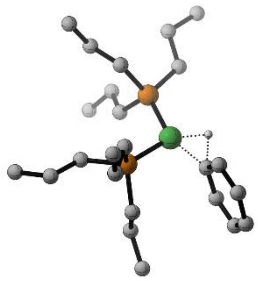

E_{el} = -3134.093869

Zero-point correction = 0.674771

Thermal correction to Energy = 0.711776

Thermal correction to Enthalpy = 0.712720

Thermal correction to Gibbs Free Energy = 0.603948

Ni	0.14227200	-1.08409000	-0.14571700
P	-2.00138300	-0.63463000	-0.12311600
C	-2.49231900	0.07672200	1.52263300
C	-2.49892100	0.63425200	-1.39098900
C	-3.15658100	-2.05748400	-0.41120700
C	-3.68591600	1.56220700	-1.13503800
C	-3.87311400	2.58230600	-2.25437000
C	-4.60407500	-1.82590100	-0.84478300
C	-5.36986600	-3.13625400	-1.00718000
C	-3.94214200	0.04058900	2.00426400
C	-4.10354200	0.67281300	3.38387900
H	-2.64035800	0.07742400	-2.33381400
H	-1.59474600	1.24352600	-1.55418400
H	-4.60999000	0.97666700	-1.01382000
H	-3.53505400	2.09589400	-0.18198200
H	-3.11572200	-2.65348900	0.51794300
H	-2.64120800	-2.67503800	-1.16460300
H	-5.12967800	-1.18478400	-0.12135600
H	-4.61564100	-1.27980600	-1.80260100
H	-2.11381400	1.11322000	1.51796300
H	-1.85680600	-0.45540700	2.25047300
H	-4.59870500	0.55773800	1.28825000
H	-4.28996300	-1.00462400	2.04127900
H	-4.06028900	2.08481700	-3.21898100
H	-2.97407600	3.20760100	-2.37448600
H	-3.80887000	1.73425900	3.37085000
H	-3.47385700	0.16344100	4.13026900
H	-5.41989300	-3.68600500	-0.05408100
H	-4.88026700	-3.79404100	-1.74232700
H	-5.14601900	0.61854600	3.73147100
H	-4.72228600	3.25152200	-2.05083200
H	-6.40135000	-2.96036800	-1.34730100

H	0.20471500	-2.54770000	-0.37417700
C	1.69646800	-2.18123300	-0.24123700
C	2.29202200	-2.70806000	0.92636200
C	2.47595700	-2.23457900	-1.41809000
C	3.60750200	-3.16798000	0.93785200
H	1.71213200	-2.74456600	1.85324300
C	3.79342600	-2.69144700	-1.40906000
H	2.04240300	-1.89564600	-2.36405800
C	4.37645800	-3.15049600	-0.22732000
H	4.03702200	-3.55227800	1.86758100
H	4.36981700	-2.69390600	-2.33867200
H	5.40725700	-3.51179300	-0.21898500
P	1.23294000	0.84506600	-0.01779000
C	1.73075300	1.36408900	-1.73245300
H	0.92417400	2.02290300	-2.10060100
H	1.67947700	0.45176100	-2.34763300
C	0.42934300	2.36380000	0.67937200
H	-0.53029100	2.47021400	0.14388800
H	0.15708800	2.10516100	1.71764600
C	2.82029500	0.73932900	0.91553700
H	3.37147300	-0.10025300	0.46012000
H	3.43214400	1.64500900	0.77850900
C	1.19236100	3.68596200	0.64859200
H	2.17325000	3.56126000	1.13664300
H	1.40382200	3.96187400	-0.39759000
C	0.42831100	4.81973600	1.32602600
H	0.98555100	5.76719300	1.27864900
H	-0.54933400	4.98184600	0.84458600
H	0.24096100	4.59371200	2.38764400
C	2.59990500	0.46160800	2.40233500
H	1.87124800	-0.35890300	2.51027200
H	2.14388700	1.34515700	2.88234800
C	3.89076100	0.09544800	3.12539300
H	4.63780900	0.90101900	3.04284300
H	3.71387900	-0.09000600	4.19550400
H	4.32979300	-0.81515400	2.68918200
C	3.09498400	2.02401100	-1.93600600
H	3.88307200	1.31988800	-1.62548200
H	3.19777600	2.90540700	-1.28281700
C	3.32711300	2.43438900	-3.38668400
H	2.57417800	3.16662100	-3.71953600
H	4.31947800	2.89023000	-3.52084500
H	3.26372000	1.56486300	-4.05970200

E’:

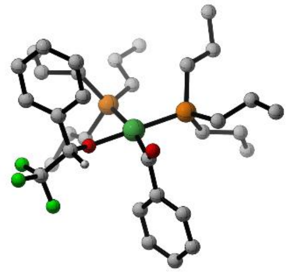

E_{el} = -3930.000712

Zero-point correction = 0.811232

Thermal correction to Energy = 0.860530

Thermal correction to Enthalpy = 0.861474

Thermal correction to Gibbs Free Energy = 0.725601

Ni	0.23162600	-0.39629200	0.17146100
P	2.26937600	0.26451100	0.59879900
C	3.39061100	-0.90022600	1.48620700
C	3.09521700	0.63167700	-1.01429900
C	2.28123800	1.79218100	1.64903800
C	4.61629800	0.74331300	-1.11401700
C	5.06579200	1.04076600	-2.54136900
C	3.36087500	2.84798800	1.41310600
C	3.14711100	4.08879900	2.27366300
C	4.64790200	-0.36046900	2.16861400
C	5.45870800	-1.47774100	2.81920800
H	2.61654500	1.54617000	-1.39975600
H	2.74561900	-0.18107900	-1.67387500
H	4.98985200	1.52954100	-0.44022900
H	5.07680100	-0.19928500	-0.77749600
H	2.31110700	1.43233400	2.69146900
H	1.28614000	2.24864400	1.53622600
H	4.35563500	2.42131600	1.61495400
H	3.36549200	3.14035700	0.34929100
H	3.64744100	-1.68274100	0.75130400
H	2.75040900	-1.40322800	2.22782300
H	5.28250300	0.18311400	1.45293500
H	4.36112300	0.37233900	2.94048600
H	4.64617800	1.99427500	-2.89955300
H	4.73409300	0.25171800	-3.23406100
H	5.80022600	-2.20744400	2.06853200
H	4.85571900	-2.02424100	3.56062500
H	3.16452600	3.83851600	3.34583700
H	2.17261700	4.55606800	2.05921000
C	0.74467600	-2.15802700	0.57952600
C	1.14402600	-2.97110900	-0.63051300

O	0.79404200	-2.66498400	1.68366400
C	2.11367300	-3.97060700	-0.48589500
C	1.99942400	-4.43299400	-2.85232900
C	0.59109600	-2.71937700	-1.89172700
C	1.01217700	-3.45743100	-2.99634200
H	2.51316900	-4.17043300	0.51141000
H	2.33506800	-5.00270200	-3.72210600
H	-0.20372400	-1.97440500	-1.97560200
H	0.56163100	-3.27622000	-3.97479500
H	6.34768900	-1.08075600	3.33109600
H	6.16134200	1.11014600	-2.61050800
H	3.92772200	4.84205200	2.09181700
C	2.55042300	-4.68978400	-1.59548700
H	3.31596900	-5.46100800	-1.48170400
O	-1.51234300	-0.96424200	-0.19679900
C	-2.28928200	-1.82774500	0.53028000
H	-1.75263700	-2.74944000	0.84073900
C	-2.81372600	-1.17330900	1.80604700
C	-4.05448500	-0.53831900	1.91282700
C	-1.92326200	-1.09446800	2.88519100
C	-4.39609100	0.16172600	3.07140100
H	-4.76700800	-0.57674800	1.08839200
C	-2.25955700	-0.38688900	4.03623200
H	-0.94945800	-1.58736300	2.80596600
C	-3.49867300	0.24777400	4.13322000
H	-5.37262900	0.64734300	3.14002100
H	-1.55043500	-0.33581900	4.86597200
H	-3.76651700	0.79960800	5.03730000
C	-3.37926300	-2.35043400	-0.40534600
F	-4.25887900	-3.13520400	0.22892600
F	-2.84372000	-3.07377500	-1.39350100
F	-4.08231700	-1.36324600	-0.99316500
P	-0.67276900	1.58559400	-0.66206300
C	-1.49927000	1.08139500	-2.23009100
H	-0.73164200	0.49067900	-2.75987100
H	-2.24691600	0.34235400	-1.90730400
C	-1.94500700	2.12953000	0.57547700
H	-1.50208300	2.95921100	1.15340200
H	-1.98755000	1.27079800	1.25938500
C	0.33548600	3.07826600	-1.12054500
H	1.15861300	3.13230800	-0.39118100
H	0.81310800	2.82237800	-2.08317600
C	-3.38219600	2.46261900	0.15698800
H	-3.71539900	1.74710400	-0.61195000
H	-4.01672800	2.25072600	1.03188000

C	-3.64670000	3.89595300	-0.28716700
H	-4.71823500	4.04972300	-0.48401700
H	-3.34702300	4.61320800	0.49371800
H	-3.10316400	4.16461000	-1.20353000
C	-2.08860600	2.11756000	-3.18277200
H	-1.30217900	2.81093200	-3.52561900
H	-2.83706600	2.73292800	-2.66222700
C	-2.74030800	1.45350200	-4.39253300
H	-3.16878200	2.19848800	-5.07953200
H	-2.00957100	0.85453900	-4.95864300
H	-3.54804300	0.77495300	-4.07781600
C	-0.31210700	4.45877100	-1.21237500
H	-1.08161000	4.46578400	-1.99799700
H	-0.83811000	4.68178700	-0.27083200
C	0.71429500	5.55277800	-1.49116800
H	0.23948200	6.54336200	-1.54737500
H	1.47895700	5.59123300	-0.69865500
H	1.23475900	5.37665400	-2.44588100

F’:

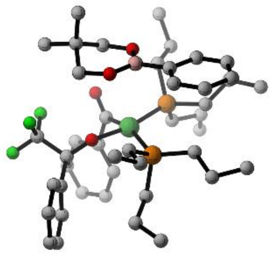

E_{el} = -4533.442749

Zero-point correction = 1.062528

Thermal correction to Energy = 1.125709

Thermal correction to Enthalpy = 1.126653

Thermal correction to Gibbs Free Energy = 0.962535

Ni	-0.10248700	0.73758400	-0.21874100
P	1.75422300	1.87822700	-0.66791400
C	2.47358500	2.54593900	0.88848700
C	3.13064700	0.96549300	-1.48684200
C	1.41270900	3.32225400	-1.77440600
C	4.57022100	1.46689400	-1.37153000
C	5.50608600	0.64738500	-2.25582000
C	2.55526500	4.00622100	-2.52450100
C	2.05400900	5.17232500	-3.37167100
C	3.33021300	3.81096200	0.86176600
C	3.77207400	4.20208100	2.26923900
H	2.84438500	0.87491700	-2.54892600

H	3.08857000	-0.05057700	-1.07176200
H	4.65047700	2.52895800	-1.64340200
H	4.89170300	1.39481300	-0.31949400
H	0.86882800	4.05304000	-1.15245600
H	0.67044700	2.96239000	-2.50441500
H	3.31650400	4.36956100	-1.81868000
H	3.06184700	3.27529700	-3.17605400
H	3.01476300	1.70107900	1.34293100
H	1.60291700	2.72461300	1.53563900
H	4.21750700	3.67690800	0.22491800
H	2.75216700	4.64029600	0.42227200
H	5.27922800	0.80632100	-3.32204100
H	5.39718900	-0.42913000	-2.05387400
H	4.37524100	3.40397600	2.72999500
H	2.90086600	4.37646000	2.91942100
H	1.57361000	5.93903100	-2.74418600
H	1.30930200	4.83665600	-4.11030000
C	-0.77370000	2.42045200	0.34651000
C	-1.81731300	3.03181800	-0.57046800
O	-0.52180500	2.98218300	1.39346300
C	-2.65021800	4.04348000	-0.08213500
C	-3.82682800	4.10729400	-2.19117300
C	-1.99416300	2.57219900	-1.87898000
C	-2.99087500	3.10753100	-2.69047600
H	-2.50120400	4.38170000	0.94525900
H	-4.61757000	4.52216900	-2.82043600
H	-1.34958100	1.77179400	-2.25301000
H	-3.12318300	2.73929200	-3.71031900
H	4.37726000	5.12093800	2.26067100
H	6.55919000	0.92084900	-2.09364700
H	2.87907900	5.65275400	-3.91803400
C	-3.65439300	4.57501600	-0.88694200
H	-4.31378000	5.35317100	-0.49602400
O	-1.65552700	-0.09027700	0.51186800
C	-2.98249800	0.23690300	0.50699400
H	-3.21249800	1.16657600	-0.04815300
C	-3.86052000	-0.84647000	-0.11363000
C	-3.91097200	-2.14098600	0.41766300
C	-4.61734500	-0.55982600	-1.25146700
C	-4.69819600	-3.12289500	-0.17902200
H	-3.33556200	-2.37576500	1.31468900
C	-5.40976000	-1.53970500	-1.85056500
H	-4.58516800	0.44927100	-1.67322600
C	-5.44999600	-2.82555900	-1.31713400
H	-4.73210000	-4.12638600	0.25191100

H	-5.99541300	-1.29669200	-2.74033700
H	-6.06908900	-3.59546600	-1.78300200
C	-3.48445700	0.56732700	1.92063700
F	-4.79794700	0.82759800	1.92206700
F	-2.87206900	1.65079300	2.41331000
F	-3.27927300	-0.42420800	2.80339700
P	0.11278600	-1.37475200	-1.29164700
C	-0.44935900	-2.71525500	-0.17059500
H	0.04668300	-2.51749400	0.78898900
H	-1.50259100	-2.47069400	0.01133800
C	-1.18067700	-1.20921000	-2.62279000
H	-0.66508100	-0.82173800	-3.51983600
H	-1.80311400	-0.38481700	-2.24557800
C	1.65585300	-1.96185700	-2.12632400
H	2.05661400	-1.07026000	-2.62712300
H	2.35994900	-2.18273900	-1.30532000
C	-2.14147500	-2.34885500	-2.98724500
H	-2.51523600	-2.82073000	-2.06474300
H	-3.03035100	-1.87309600	-3.43033200
C	-1.64851000	-3.41045300	-3.96140100
H	-2.47553500	-4.08009100	-4.24171800
H	-1.26140300	-2.95638300	-4.88808700
H	-0.85055800	-4.03669200	-3.54183600
C	-0.28346900	-4.18824300	-0.52801400
H	0.78517000	-4.42516000	-0.65424900
H	-0.76997800	-4.41824700	-1.48786400
C	-0.87359500	-5.07607100	0.56395000
H	-0.73388500	-6.14445900	0.34066200
H	-0.40348100	-4.86587100	1.53795000
H	-1.95358200	-4.89119400	0.67599700
C	1.66618400	-3.10167600	-3.14197500
H	1.25045600	-4.02038400	-2.70605500
H	1.01733000	-2.84093100	-3.99193000
C	3.07960400	-3.37595200	-3.64836600
H	3.09008100	-4.18276000	-4.39625500
H	3.51515900	-2.47829900	-4.11635000
H	3.74232000	-3.66921800	-2.81939100
C	5.03723000	-2.17793500	0.21818200
C	4.10622900	-1.49757600	1.00201900
C	2.96033800	-2.14153500	1.49046200
C	2.78108600	-3.49672100	1.17587300
C	3.70043000	-4.18242300	0.38419200
C	4.83076500	-3.52137800	-0.09818100
H	5.93154500	-1.66404700	-0.14134700
H	4.26876900	-0.44521000	1.24990500

H	1.89934800	-4.01439400	1.56089200
H	3.53946200	-5.23663700	0.14665500
H	5.55763400	-4.05682200	-0.71373800
C	-0.22907000	-1.35155200	3.47207600
C	1.14379800	0.67601300	3.39449200
C	0.34060000	-0.22308500	4.33884700
H	-0.77848300	-2.07876100	4.08930500
H	-0.91787900	-0.94391200	2.71356300
H	1.70065700	1.44071400	3.95943900
H	0.45889000	1.19909600	2.70731100
B	1.89464000	-1.37362400	2.35559500
O	2.09236000	-0.05667900	2.62884600
O	0.80763400	-2.05724600	2.80192400
C	1.23573400	-0.79235600	5.44718400
H	1.65000600	0.02043900	6.06367800
H	0.65632800	-1.45662300	6.10702400
H	2.08008100	-1.37344900	5.04822100
C	-0.80088800	0.58910400	4.95030300
H	-1.43111100	-0.04776900	5.59041200
H	-0.40241700	1.40291800	5.57613100
H	-1.44041700	1.03434100	4.17743900

[F’-G]‡:

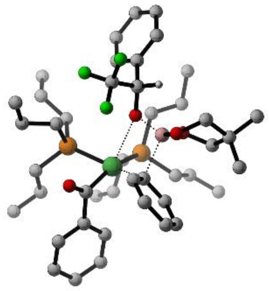

E_{el} = -4533.405627

Zero-point correction = 1.061347

Thermal correction to Energy = 1.123397

Thermal correction to Enthalpy = 1.124341

Thermal correction to Gibbs Free Energy = 0.963890

Ni	0.97113700	-0.26917400	-0.23228000
O	-1.70870300	0.37813800	-0.80248700
B	-1.86573300	-1.05759500	-0.82648100
C	0.01839900	-1.71752100	-1.36106400
P	0.35854700	-0.73856600	2.00459400
C	2.12792700	-0.21516100	-1.73312600
P	1.73601300	1.82904900	-0.01267100
C	-1.16243200	0.12905300	2.60685700

C	3.52468900	2.20179600	-0.28760800
C	0.84884400	2.74505900	-1.34808800
C	1.37088400	2.81508700	1.51482500
C	0.21586200	-2.53397300	2.44174400
C	1.63534500	-0.22968000	3.24766800
C	-2.15161000	-0.57608000	3.53061000
C	-3.46219400	0.19804400	3.63149600
C	0.23805900	-2.96947100	3.90802200
C	3.00256600	-0.86756800	3.01289400
C	1.60759000	3.83078800	-2.10911900
C	0.72900300	4.50636400	-3.15712800
C	1.31757700	4.33707600	1.38730700
C	1.04984500	5.01349100	2.72838500
C	4.48776700	1.48280800	0.65321100
O	2.05325300	0.43754000	-2.75421400
C	0.26923700	-3.06420200	-0.99814300
C	0.38137700	-4.09681800	-1.92455200
C	0.21374000	-3.82401800	-3.28220700
C	-0.12182500	-1.49952800	-2.75464300
C	-0.04784400	-2.51922600	-3.69611800
O	-2.68773500	-1.54466000	-1.87273500
O	-2.16899100	-1.62715900	0.42956800
H	-1.68977700	0.40411600	1.68682300
H	-0.82070700	1.06990500	3.07135200
H	-2.36011400	-1.58407500	3.14038900
H	-1.71998400	-0.70558200	4.53677700
H	-3.92254000	0.31358900	2.63782800
H	-3.29951600	1.21003300	4.03413500
H	1.05155000	-3.02641500	1.91718200
H	-0.69629700	-2.89660200	1.95542400
H	1.21834700	-2.74192500	4.35610100
H	-0.50066600	-2.40212100	4.49077200
H	1.26339800	-0.45926400	4.26022300
H	1.72376400	0.86353900	3.19854000
H	2.92884700	-1.96352200	3.11217100
H	3.30776900	-0.68335100	1.97193300
H	-0.05763300	3.16007300	-0.88811300
H	0.51742500	1.97904800	-2.06108300
H	2.00620500	4.59123600	-1.41721500
H	2.47643000	3.37438100	-2.60768200
H	2.13796400	2.53840900	2.25543200
H	0.41427300	2.44263300	1.91473200
H	2.26521300	4.71479700	0.96967900
H	0.53207100	4.62452000	0.67047700
H	3.66615200	3.29263500	-0.22189300

H	3.74483000	1.92168500	-1.32859000
H	4.25360800	1.73055700	1.70261700
H	4.34709100	0.39704700	0.55007600
H	-0.13147000	5.01205300	-2.69357300
H	0.33005200	3.76636700	-3.86708200
H	1.84701200	4.79203300	3.45526100
H	0.10042300	4.66448200	3.16486300
H	0.39457400	-3.31759400	0.05394400
H	0.59046600	-5.11597900	-1.59025900
H	-0.30577000	-0.48309700	-3.10544100
H	-0.18196100	-2.29518000	-4.75648300
C	4.07513300	-0.34783600	3.96256400
H	3.81495200	-0.55235300	5.01322000
H	4.20167700	0.74180200	3.85976400
C	-0.04444000	-4.46237600	4.05009500
H	0.68847200	-5.06211000	3.48732000
H	-1.04386800	-4.71314400	3.66079000
C	3.38528500	-1.04166100	-1.50321000
C	3.48448900	-2.00961500	-0.49873600
C	4.51232300	-0.75423700	-2.28179100
H	2.60668400	-2.24886900	0.10262400
C	5.72247400	-1.39899400	-2.04280200
H	4.41360500	-0.00666100	-3.07202700
C	5.81551800	-2.35276500	-1.02790000
H	6.59903900	-1.15891600	-2.64919300
H	6.76482800	-2.85880300	-0.83788400
C	4.69169000	-2.66382800	-0.26258700
H	4.75620500	-3.41905900	0.52394300
H	1.29795400	5.25526200	-3.72822100
H	0.98649700	6.10610500	2.61976500
H	-4.18199400	-0.31498600	4.28706500
H	-0.00282100	-4.78172400	5.10205500
H	0.28953400	-4.62935900	-4.01744000
C	-3.91004500	-2.09240900	-1.47139500
H	-4.46800600	-2.38445100	-2.37614400
H	-4.52778600	-1.33677100	-0.94195100
C	-2.62909300	-2.95260000	0.48059300
H	-3.00456100	-3.11921000	1.50428600
H	-1.78130000	-3.64398000	0.33115700
C	-3.73501700	-3.30762000	-0.55408300
C	-3.29397800	-4.51498600	-1.38755700
H	-2.39927800	-4.26655900	-1.97726600
H	-3.05216500	-5.37654800	-0.74395600
H	-4.09127700	-4.82598200	-2.08115500
C	-5.06242500	-3.60046000	0.14970400

H	-5.86907100	-3.77295600	-0.58113800
H	-4.98578500	-4.50210700	0.77788900
H	-5.36336100	-2.76077500	0.79669100
C	-2.85134400	1.10452300	-1.12476100
H	-3.66162500	0.43569500	-1.44569900
C	-2.63505900	1.97695500	-2.37532700
F	-2.03462800	3.14665600	-2.11775300
F	-1.91135900	1.35616800	-3.30080100
F	-3.82564000	2.27294000	-2.91553900
C	-3.36052000	1.94970600	0.02582100
C	-4.72699300	2.02375200	0.29761600
C	-2.47219400	2.68349800	0.81636100
C	-5.20039900	2.81726300	1.34316800
H	-5.42943100	1.45334100	-0.31558400
C	-2.93912300	3.47658300	1.85848100
H	-1.40369500	2.61167500	0.61342100
C	-4.30754800	3.54628300	2.12482600
H	-6.27217700	2.86384500	1.54724100
H	-2.23291700	4.04479100	2.46846600
H	-4.67610600	4.16773400	2.94376900
H	5.04977800	-0.81606900	3.75949500
C	5.94487400	1.82328300	0.36203200
H	6.13972600	2.90081100	0.48277700
H	6.62179900	1.27841800	1.03643300
H	6.20744800	1.54567600	-0.67065600

[E-M]‡:

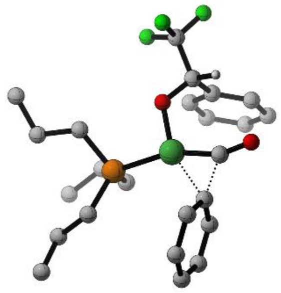

E_{el} = -3233.143312

Zero-point correction = 0.519473

Thermal correction to Energy = 0.553248

Thermal correction to Enthalpy = 0.554192

Thermal correction to Gibbs Free Energy = 0.450351

Ni	0.00025600	-0.95392800	-0.79054100
C	-0.69262200	-2.55317700	-1.00168300
O	-1.53376000	-3.34503200	-1.25030900
O	-1.30233800	0.29215100	-1.04492800

C	-2.61963800	-0.01997800	-0.82105600
H	-2.96451900	-0.90733100	-1.39300500
C	-2.90837000	-0.27912800	0.65166500
C	-2.50009500	0.64838900	1.61806000
C	-3.49678900	-1.47368200	1.06903500
C	-2.68547300	0.38847400	2.97277000
H	-2.03004900	1.57896400	1.29471000
C	-3.67955600	-1.73970000	2.42729500
H	-3.79731000	-2.21221800	0.32165700
C	-3.27500900	-0.80969300	3.38223700
H	-2.37063000	1.12515000	3.71607800
H	-4.13849300	-2.68073700	2.73909600
H	-3.42040100	-1.01467500	4.44536400
C	-3.46596200	1.13064100	-1.37355700
F	-4.77080900	0.92918000	-1.14767900
F	-3.30512500	1.25260500	-2.69307100
F	-3.14623500	2.31387800	-0.82854400
P	1.44509100	0.72335300	-0.14802100
C	0.97234200	2.28056500	-1.00103200
H	0.85667600	1.98435300	-2.05678700
H	-0.06379500	2.46190000	-0.67110400
C	1.12618100	0.91338800	1.67583300
H	1.88403100	0.30192000	2.19558100
H	0.17230800	0.38073500	1.80954400
C	3.25401100	0.45211600	-0.39046500
H	3.40291700	-0.62437800	-0.19902700
H	3.41844700	0.57996700	-1.47517500
C	0.98624900	2.29076700	2.33549300
H	0.40503800	2.95829600	1.67815000
H	0.35789700	2.15410200	3.23100700
C	2.28218200	2.96840500	2.76349000
H	2.07406600	3.91674500	3.28073300
H	2.85237500	2.32889700	3.45597600
H	2.93830100	3.19470500	1.91175700
C	1.83885100	3.53386900	-0.90420300
H	2.83002900	3.33420000	-1.34480800
H	2.01901300	3.79881100	0.14812100
C	1.19680700	4.71909900	-1.61957800
H	1.83178800	5.61573600	-1.56205100
H	1.02398600	4.49516800	-2.68369400
H	0.22112900	4.96509200	-1.17287800
C	4.27887500	1.25688400	0.40528400
H	4.14337100	2.33316900	0.21921600
H	4.10256000	1.10878700	1.48260100
C	5.71238900	0.85730800	0.06856700

H	6.43824400	1.43193700	0.66255300
H	5.88321900	-0.21218400	0.27001200
H	5.93418100	1.03327200	-0.99574400
C	0.83380700	-2.85118400	-0.58901500
C	1.81334400	-3.06944100	-1.56979900
C	1.17082200	-2.95717400	0.76936400
C	3.11161700	-3.40552000	-1.19531000
H	1.54993900	-2.97236900	-2.62589500
C	2.47223000	-3.28871500	1.14127500
H	0.40484500	-2.77803800	1.52902900
C	3.43700700	-3.51470900	0.15854000
H	3.87451200	-3.57758600	-1.95717300
H	2.73455400	-3.37298900	2.19780100
H	4.45733700	-3.77443400	0.45008400

M:

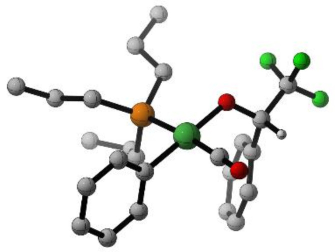

E_{el} = -3233.162869

Zero-point correction = 0.519689

Thermal correction to Energy = 0.553985

Thermal correction to Enthalpy = 0.554930

Thermal correction to Gibbs Free Energy = 0.452348

Ni	0.25009700	-1.20695800	-0.76912900
C	-0.07378100	-2.95845400	-1.06074200
O	-0.23089400	-4.06431400	-1.25451900
O	-1.45562000	-0.57989300	-1.22909500
C	-2.54711900	-1.00221900	-0.52366000
H	-2.73649300	-2.09912800	-0.61533000
C	-2.42459600	-0.71290600	0.97436500
C	-2.72406400	0.53010800	1.54496400
C	-1.86553500	-1.69908400	1.79421700
C	-2.46791500	0.77515900	2.89331500
H	-3.17558600	1.31394200	0.93550600
C	-1.59120000	-1.45343200	3.13870300
H	-1.64042300	-2.68226000	1.37099300
C	-1.89169400	-0.21015700	3.69375200
H	-2.71743100	1.74851600	3.32173200
H	-1.15284400	-2.24022200	3.75678200

H	-1.68813800	-0.01412200	4.74868000
C	-3.79071800	-0.37897000	-1.16425900
F	-4.89867300	-0.64740500	-0.45939300
F	-3.97600600	-0.85947700	-2.39410700
F	-3.70424800	0.95596300	-1.27437300
P	0.86613900	0.91458200	-0.34589800
C	-0.40862300	2.08667100	-0.95093600
H	-0.79589700	1.63517700	-1.87647300
H	-1.24697800	1.98328600	-0.24586100
C	0.96626800	1.09208500	1.49628000
H	1.98429900	0.78734300	1.78968500
H	0.30459800	0.28674600	1.85133900
C	2.47223800	1.39095700	-1.11065100
H	3.08981100	0.48216400	-1.05465800
H	2.24742800	1.53466300	-2.18230700
C	0.54228500	2.38577800	2.20356400
H	-0.38209400	2.77415400	1.74615100
H	0.24834000	2.09382200	3.22458600
C	1.58291900	3.49369200	2.30830700
H	1.21089800	4.30881400	2.94665500
H	2.51705400	3.12038200	2.75689400
H	1.83571900	3.93117500	1.33337500
C	-0.04257400	3.55317000	-1.16168300
H	0.74847200	3.63255500	-1.92576100
H	0.37620500	3.98657200	-0.24007300
C	-1.25377300	4.37126600	-1.59996000
H	-0.98688100	5.42347200	-1.77801500
H	-1.68791200	3.96803600	-2.52773800
H	-2.04288700	4.34807000	-0.83238300
C	3.27513100	2.56914300	-0.56541200
H	2.69958600	3.50306100	-0.64745500
H	3.46370000	2.41340800	0.50792900
C	4.60765000	2.72720000	-1.29173200
H	5.18656200	3.56874200	-0.88378400
H	5.21967800	1.81684500	-1.19550700
H	4.45515000	2.91333800	-2.36644000
C	2.00202500	-1.79583000	-0.24702900
C	3.03767400	-2.01272200	-1.16885300
C	2.27605000	-2.03630100	1.10804800
C	4.30885000	-2.41191200	-0.75099000
H	2.85841900	-1.85786400	-2.23741200
C	3.54603100	-2.43357800	1.53198200
H	1.48642600	-1.90519200	1.85530000
C	4.56970200	-2.61743300	0.60336200
H	5.09919300	-2.56723500	-1.49000300

H	3.73478700	-2.60449800	2.59500800
H	5.56351000	-2.92913500	0.93272900

N:

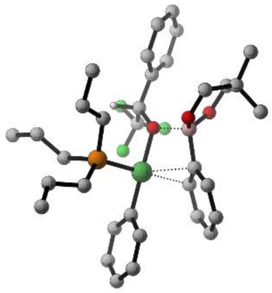

E_{el} = -3723.271158

Zero-point correction = 0.760631

Thermal correction to Energy = 0.806242

Thermal correction to Enthalpy = 0.807186

Thermal correction to Gibbs Free Energy = 0.681875

Ni	1.02453200	-0.63043100	0.28359200
P	1.82900200	1.24298000	-0.50111300
C	3.24239400	1.14187800	-1.67339500
C	0.44698700	2.06867500	-1.40166900
C	2.35614800	2.36080000	0.86953500
C	0.72858800	3.09707200	-2.49471600
C	-0.57127000	3.61905600	-3.10098500
C	2.25080500	3.87174800	0.65888400
C	2.69037200	4.65360700	1.89282100
C	4.06258100	2.40038100	-1.96027200
C	5.16339500	2.13160600	-2.98199500
H	-0.17286600	2.52847200	-0.61681300
H	-0.16154500	1.23846300	-1.79957300
H	1.31046200	3.94174000	-2.09357400
H	1.34496700	2.64200000	-3.28699600
H	3.38863600	2.06106700	1.11843700
H	1.74975700	2.06612900	1.74074800
H	2.85393800	4.18089100	-0.20832200
H	1.20769600	4.13312300	0.41452800
H	2.82297600	0.72178200	-2.60419400
H	3.90468300	0.36253900	-1.27037300
H	3.42027900	3.21655100	-2.32176900
H	4.51849300	2.75751800	-1.02247000
H	-1.19102700	4.11679300	-2.33811000
H	-1.16578500	2.79544800	-3.52636400
H	4.73914500	1.79955400	-3.94248300
H	5.84729900	1.34353100	-2.63069900

H	3.74540100	4.45162000	2.13484100
H	2.08847000	4.37758700	2.77273400
H	5.75923000	3.03625000	-3.17254900
H	-0.37799500	4.34754700	-3.90207500
H	2.58207100	5.73691600	1.73735200
O	-0.83054600	0.01832400	0.45176100
C	-1.35229100	0.94928700	1.33480600
H	-0.76860500	1.88641800	1.23488000
C	-2.80133900	1.33319900	1.07100900
C	-3.85317900	1.08927900	1.95788800
C	-3.07746300	1.99412100	-0.12994900
C	-5.14519300	1.51682900	1.65568400
H	-3.68144500	0.55250800	2.88959100
C	-4.36887900	2.41450700	-0.43719100
H	-2.27188000	2.16339100	-0.84581300
C	-5.40926400	2.18274900	0.46092200
H	-5.95383800	1.31714500	2.36181700
H	-4.56049400	2.92397400	-1.38414100
H	-6.42354900	2.51322600	0.22749900
C	-1.05476000	0.54618400	2.78393900
F	-1.40994000	1.51286500	3.63483300
F	0.27172100	0.36317600	2.94156400
F	-1.64514300	-0.58204800	3.17062500
C	0.97912000	-3.74588900	1.35755600
C	0.09906900	-2.66817200	1.14968800
C	-0.36034900	-2.32876600	-0.14032700
C	0.13142200	-3.09655900	-1.22059300
C	0.99215700	-4.16334700	-1.01675600
C	1.41997600	-4.49205700	0.27914400
H	1.31088200	-3.98876400	2.36909900
H	-0.31864200	-2.15974500	2.02142900
H	-0.20084600	-2.83552700	-2.22869500
H	1.34927600	-4.74925600	-1.86760800
H	2.10839000	-5.32605100	0.43168000
C	-2.83587800	-0.65907100	-2.37524700
C	-3.98260000	-1.46182900	-0.35196400
C	-3.89400800	-1.65051200	-1.87255700
H	-2.67481000	-0.80678400	-3.45919600
H	-3.23744100	0.36659100	-2.24760700
H	-4.65580800	-2.22579900	0.07724000
H	-4.44872700	-0.47869500	-0.15234200
B	-1.53170000	-1.19156900	-0.34791600
O	-2.74657600	-1.55875500	0.28982400
O	-1.60758000	-0.79568000	-1.72794900
C	-3.49817700	-3.09123900	-2.21204800

H	-4.28315200	-3.79341900	-1.88936800
H	-3.35625500	-3.21641500	-3.29769900
H	-2.56574200	-3.38461400	-1.71095000
C	-5.24354700	-1.31168300	-2.50549500
H	-5.20448500	-1.42634100	-3.60072000
H	-6.03290500	-1.98122000	-2.12859300
H	-5.54411000	-0.27543900	-2.28143300
C	2.77059200	-1.29699300	0.37102400
C	3.43777400	-1.13852700	1.59531700
C	3.42029500	-2.01206400	-0.64341600
C	4.69817800	-1.70100700	1.80817500
H	2.96244200	-0.58358800	2.41091100
C	4.68228600	-2.57129400	-0.43665100
H	2.93379300	-2.14107600	-1.61321600
C	5.32553000	-2.42057400	0.79156200
H	5.19256200	-1.57594100	2.77521900
H	5.16655500	-3.13003100	-1.24213100
H	6.31217000	-2.86028600	0.95490800

[N-O]‡:

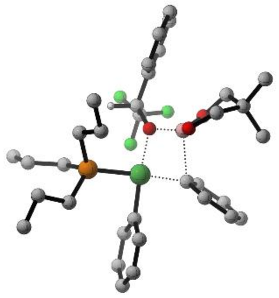

E_{el} = -3723.260259

Zero-point correction = 0.759312

Thermal correction to Energy = 0.804910

Thermal correction to Enthalpy = 0.805854

Thermal correction to Gibbs Free Energy = 0.679134

Ni	0.86621400	0.59017300	-0.09942600
P	2.26730900	-1.09229000	0.33784400
C	1.47859200	-2.37272300	1.40761600
C	2.79840200	-1.98152700	-1.18548100
C	3.81571600	-0.64559500	1.23327100
C	3.82311500	-3.10950100	-1.08031600
C	4.05712500	-3.79716500	-2.42206900
C	4.52696100	-1.71437400	2.06358900
C	5.84070500	-1.19450600	2.64004100
C	0.86506400	-1.75714100	2.66806000
C	0.24259900	-2.80090000	3.58912900

H	3.16879300	-1.19104400	-1.85974400
H	1.87154900	-2.35074700	-1.65535600
H	4.77714000	-2.70296300	-0.70886300
H	3.49231200	-3.85371100	-0.33669500
H	3.55502600	0.21001100	1.87406900
H	4.48565700	-0.21741000	0.46826000
H	3.87139000	-2.03467900	2.89011700
H	4.72091500	-2.61782900	1.46561800
H	2.21000000	-3.15506800	1.66734500
H	0.69261500	-2.86925800	0.81563900
H	1.63964500	-1.19890500	3.22120900
H	0.11141500	-1.00761700	2.37358700
H	4.40856100	-3.07740200	-3.17763000
H	3.12947500	-4.25148500	-2.80403100
H	0.99446000	-3.52475000	3.94134100
H	-0.54483000	-3.37154700	3.07070400
H	5.67328300	-0.30069300	3.26083000
H	6.54073000	-0.91102500	1.83883900
H	-0.21166900	-2.33074600	4.47364400
H	4.81229100	-4.59256800	-2.33863800
H	6.33280200	-1.95376200	3.26576000
O	-0.77288900	-0.49590500	-0.16992900
C	-1.13747200	-1.58262500	-0.96995700
H	-0.34153800	-2.33571400	-0.84281500
C	-2.43984400	-2.20860600	-0.49722200
C	-3.47875100	-2.60130900	-1.34318900
C	-2.58133000	-2.39465400	0.88332200
C	-4.64188800	-3.16024500	-0.81315800
H	-3.40336400	-2.46509200	-2.42119700
C	-3.74334800	-2.94982700	1.41029400
H	-1.78350000	-2.06807600	1.55191500
C	-4.78104700	-3.33473700	0.56136000
H	-5.44842800	-3.45574500	-1.48732500
H	-3.83809400	-3.08134100	2.49029100
H	-5.69546400	-3.76859900	0.97116900
C	-1.04447200	-1.26499800	-2.47176400
F	-1.03170300	-2.41988500	-3.15635100
F	0.11229200	-0.64332100	-2.73394300
F	-2.01804500	-0.51612000	-2.96354800
C	-0.90196200	3.93478000	-1.91675000
C	-0.62672900	2.60110000	-1.61487400
C	-0.32588300	2.17955000	-0.30661600
C	-0.32778100	3.17443900	0.69003900
C	-0.57938700	4.51241800	0.40374100
C	-0.86674400	4.89612400	-0.90775200

H	-1.13411500	4.22735500	-2.94392200
H	-0.65630500	1.86671000	-2.42425500
H	-0.12704400	2.88484700	1.72595900
H	-0.56074300	5.26004900	1.20064400
H	-1.06872000	5.94478900	-1.14025200
C	-3.04668600	1.02248700	2.20803000
C	-4.07294700	0.77364300	-0.03432000
C	-4.10656200	1.60260600	1.25315900
H	-2.84115800	1.74380200	3.01706100
H	-3.45663700	0.10906200	2.67832600
H	-4.74903800	1.21328100	-0.78469400
H	-4.43164600	-0.25327000	0.17156300
B	-1.69949300	0.55534800	0.23168200
O	-2.78599000	0.71352100	-0.59779500
O	-1.82400700	0.67630100	1.60186100
C	-3.83385500	3.07328900	0.92420400
H	-4.64648500	3.48247200	0.30380500
H	-3.76985500	3.67824300	1.84225200
H	-2.89571500	3.19916600	0.37061500
C	-5.48047900	1.45955800	1.91243900
H	-5.51665800	2.01030600	2.86566800
H	-6.26934100	1.86869000	1.26245500
H	-5.72308500	0.40485000	2.11888300
C	2.34485500	1.75105700	-0.17959200
C	3.03757300	1.86647600	-1.39562600
C	2.83480800	2.48560800	0.91071200
C	4.17989000	2.66254700	-1.51220000
H	2.67655500	1.33201800	-2.28109800
C	3.97613600	3.28042200	0.80282400
H	2.31477700	2.43931000	1.87270100
C	4.65982300	3.36881000	-0.41014600
H	4.69528700	2.73514100	-2.47399500
H	4.33402200	3.83839400	1.67261200
H	5.55358800	3.99103700	-0.49781700

O:

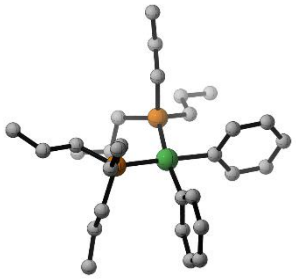

E_{el} = -3365.061987

Zero-point correction = 0.760819

Thermal correction to Energy = 0.802770

Thermal correction to Enthalpy = 0.803714

Thermal correction to Gibbs Free Energy = 0.683740

Ni	0.05360200	-0.57470000	-0.04515300
C	1.24305700	-2.05718800	0.14052800
P	1.83586900	0.78502400	-0.01898700
P	-1.67983000	0.83613600	0.22624700
C	2.19731100	1.27236200	1.72386800
C	-3.15039500	0.50048800	-0.83062600
C	-2.24269800	0.59451500	1.98500500
C	-1.54028200	2.68273100	0.12843000
C	3.39951600	0.03454400	-0.66138700
C	1.73632300	2.39556900	-0.91736400
C	3.44615700	2.09403700	2.03758800
C	3.51713000	2.49795500	3.50731500
C	4.53425500	0.94675800	-1.12535200
C	1.19735600	2.23431900	-2.33898800
C	-3.71653100	0.26902500	2.22339800
C	-4.01421300	0.00704500	3.69577400
C	-2.80055000	3.50155700	0.40101500
C	-2.53106200	5.00336100	0.39514800
C	-2.86127900	0.76236000	-2.30871600
C	1.74994400	-2.80054800	-0.93824000
C	2.70070300	-3.80496800	-0.75855100
C	3.17006600	-4.11158000	0.51956300
C	1.70599900	-2.40823600	1.42076400
C	2.65730100	-3.41360600	1.61196500
H	2.21641200	0.32439900	2.28675400
H	1.29256500	1.80521600	2.06707500
H	4.34248100	1.50817300	1.77883700
H	3.47399900	2.99805500	1.40607900
H	3.51114200	1.61206100	4.16158000
H	2.65652900	3.12474800	3.79027400
H	3.09734000	-0.62860600	-1.48629400
H	3.73925800	-0.65743300	0.12764200
H	4.20509000	1.52907300	-2.00218900
H	4.78902900	1.68661500	-0.35079800
H	2.72112300	2.88876600	-0.93065000
H	1.07293000	3.05084600	-0.33390900
H	1.88349300	1.59925600	-2.92522400
H	0.24317700	1.68120900	-2.30201800
H	-1.95850400	1.50410700	2.54172300
H	-1.62332800	-0.21784400	2.39336600
H	-4.35023900	1.09407000	1.85838600

H	-3.98977000	-0.61951500	1.63257800
H	-1.14191000	2.92902200	-0.86920100
H	-0.75127600	2.96051300	0.84828300
H	-3.56397600	3.26660800	-0.35791300
H	-3.23503800	3.20900600	1.37152500
H	-4.01582300	1.09048300	-0.49206700
H	-3.40093600	-0.56034200	-0.68440200
H	-2.69188600	1.84093000	-2.47472000
H	-1.92155200	0.25674100	-2.58628100
H	-3.75700600	0.87843500	4.31922900
H	-3.43326700	-0.85162700	4.06745200
H	-2.11720300	5.32778000	-0.57286500
H	-1.80466500	5.28095300	1.17513400
H	1.39241900	-2.59324100	-1.95226700
H	3.07748500	-4.35720400	-1.62415500
H	1.31473100	-1.88922300	2.30345900
H	2.99735600	-3.65474900	2.62328200
C	0.99726600	3.56734000	-3.05126000
H	1.94636500	4.11865900	-3.14108800
H	0.29398000	4.20967600	-2.49650100
C	5.78201000	0.14928700	-1.49465600
H	5.56007900	-0.59107700	-2.27880200
H	6.17044300	-0.40174900	-0.62416700
H	-5.07961400	-0.21614300	3.85411700
H	-3.45226600	5.57693900	0.57513100
H	4.43298300	3.06788300	3.72316400
H	6.58336200	0.80598900	-1.86491800
H	3.91596900	-4.89699400	0.66304200
C	-1.25050100	-1.96950000	-0.19829300
C	-1.92745000	-2.58317000	0.86770000
C	-1.61515900	-2.38557200	-1.49273900
C	-2.95095500	-3.51124400	0.65899600
H	-1.64932000	-2.34422100	1.89926900
C	-2.63339600	-3.31298600	-1.71372500
H	-1.09439800	-1.97133300	-2.36297300
C	-3.32071000	-3.87278200	-0.63555900
H	-3.45694100	-3.96216100	1.51743600
H	-2.88985700	-3.60432700	-2.73642800
H	-4.11956500	-4.59907900	-0.80263400
H	0.59334500	3.42510100	-4.06446500
C	-3.98491800	0.27125400	-3.21415800
H	-4.93835700	0.76741000	-2.97299000
H	-3.76326500	0.46657500	-4.27395900
H	-4.13033800	-0.81299900	-3.09021300

[H-A]‡:

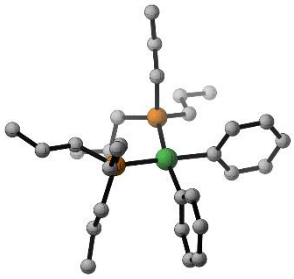

E_{el} = -3365.047616

Zero-point correction = 0.759555

Thermal correction to Energy = 0.800912

Thermal correction to Enthalpy = 0.801856

Thermal correction to Gibbs Free Energy = 0.684967

Ni	-0.07530300	-0.44454600	0.11980600
C	0.55055900	-2.20966200	-0.34596900
P	1.87608000	0.57879800	0.47049700
P	-1.68290500	1.08752200	0.03442400
C	2.03812900	1.20727200	2.20398200
C	-2.84891900	0.84169600	-1.37593700
C	-2.77913500	1.09678400	1.53632400
C	-1.22800000	2.87732500	-0.13951500
C	3.37528400	-0.47404800	0.19330600
C	2.21431900	2.08819800	-0.54492100
C	3.35840700	1.81816000	2.67042600
C	3.26758300	2.39081500	4.08198000
C	4.71257300	0.17384000	-0.16143800
C	1.92509300	1.85788500	-2.02862200
C	-4.01393900	0.19866400	1.44959200
C	-4.67808400	-0.00809900	2.80522200
C	-2.33632700	3.89402600	-0.40229300
C	-1.81736800	5.32909000	-0.41274500
C	-2.13647200	0.88857600	-2.72660900
C	0.77737400	-2.20064300	-1.74337900
C	1.76079600	-2.99315900	-2.33028700
C	2.52756700	-3.86234600	-1.55235700
C	1.31824100	-3.12073400	0.41149000
C	2.28698800	-3.92522700	-0.17713200
H	1.76346100	0.35874600	2.85376700
H	1.21794800	1.93814700	2.31764600
H	4.14765800	1.04982300	2.63924900
H	3.67396200	2.61136400	1.97242800
H	2.98088100	1.61357900	4.80772900
H	2.51126800	3.18966500	4.13785000
H	3.09476400	-1.17934900	-0.60429800

H	3.47189000	-1.09799100	1.09834300
H	4.61323400	0.71811100	-1.11514000
H	4.99954600	0.92754100	0.58851900
H	3.24717600	2.44379800	-0.40130500
H	1.55576100	2.87889400	-0.15169900
H	2.61688900	1.09500600	-2.42491400
H	0.91743800	1.42033800	-2.13455100
H	-3.07984500	2.13018100	1.77612400
H	-2.13640200	0.75357500	2.36402000
H	-4.73837000	0.63750300	0.74396600
H	-3.73645400	-0.78206000	1.03708000
H	-0.48171600	2.92799100	-0.94841400
H	-0.68294500	3.13511300	0.78606100
H	-2.81394100	3.67422700	-1.37112000
H	-3.13093100	3.79588200	0.35606000
H	-3.67392100	1.57178300	-1.33930600
H	-3.29431500	-0.15486200	-1.23017200
H	-1.68296000	1.88336300	-2.88192200
H	-1.29512300	0.17469000	-2.70575800
H	-4.97707700	0.94916000	3.26238500
H	-3.98933400	-0.51289600	3.50084800
H	-1.04195100	5.46327500	-1.18387400
H	-1.36842100	5.59639500	0.55686600
H	0.17394500	-1.54859000	-2.38279000
H	1.92221000	-2.93893500	-3.41025400
H	1.14282400	-3.20127600	1.48719200
H	2.86687100	-4.61150000	0.44573700
C	2.02608800	3.13149600	-2.86024100
H	3.02743100	3.58354700	-2.78163900
H	1.29758400	3.88452000	-2.51735600
C	5.82241000	-0.86525900	-0.29574400
H	5.55868800	-1.62411800	-1.04876500
H	5.98812900	-1.39178100	0.65736400
H	-5.57718200	-0.63574200	2.71495700
H	-2.62293600	6.04923200	-0.61960000
H	4.22879800	2.81567900	4.40762800
H	6.77499700	-0.40305100	-0.59540600
H	3.28674500	-4.49780700	-2.01307600
C	-1.17780300	-2.01896900	0.23886400
C	-1.40786400	-2.29779300	1.61068600
C	-2.14347200	-2.50997700	-0.67088700
C	-2.54040100	-2.98356700	2.04008300
H	-0.67333400	-1.97175000	2.35432000
C	-3.27021800	-3.20110900	-0.24094000
H	-1.98773400	-2.35954700	-1.74276700

C	-3.48355000	-3.44312800	1.11949700
H	-2.68689700	-3.16690800	3.10792800
H	-3.99258300	-3.56235000	-0.97787200
H	-4.36504900	-3.99349500	1.45436400
H	1.82862700	2.93508700	-3.92452800
C	-3.05959700	0.56205900	-3.89529200
H	-3.89086700	1.28198300	-3.95870500
H	-2.52074600	0.58680600	-4.85416600
H	-3.49841400	-0.44150400	-3.78029300

4b:

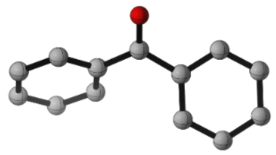

E_{el} = -576.420393

Zero-point correction = 0.194082

Thermal correction to Energy = 0.204676

Thermal correction to Enthalpy = 0.205620

Thermal correction to Gibbs Free Energy = 0.156629

C	1.43974200	-0.90332400	0.64364100
C	1.29963100	0.34584000	0.02616000
C	2.42753100	0.97003800	-0.52184200
C	3.66765700	0.34196100	-0.48301500
C	3.79889800	-0.90655500	0.12750400
C	2.68679400	-1.52335700	0.69847900
H	0.57495800	-1.38473000	1.10477800
H	2.30952200	1.95615600	-0.97500800
H	4.53957800	0.82888600	-0.92475900
H	4.77398300	-1.39727600	0.16472500
H	2.79082600	-2.49172700	1.19225900
C	0.00000100	1.09865800	-0.00001100
O	0.00000000	2.31129200	0.00005400
C	-1.29962800	0.34583700	-0.02619100
C	-2.42753000	0.97005600	0.52178600
C	-1.43974200	-0.90334800	-0.64362800
C	-3.66765800	0.34198100	0.48298100
H	-2.30951300	1.95618800	0.97492000
C	-2.68679500	-1.52338200	-0.69844200
H	-0.57496600	-1.38477400	-1.10475700
C	-3.79889900	-0.90655900	-0.12749000
H	-4.53957800	0.82892200	0.92470800
H	-2.79082800	-2.49176900	-1.19218800
H	-4.77398400	-1.39728100	-0.16469500

4c:

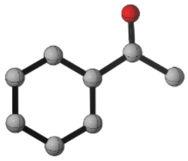

E_{el} = -384.766074

Zero-point correction = 0.139301

Thermal correction to Energy = 0.147086

Thermal correction to Enthalpy = 0.148030

Thermal correction to Gibbs Free Energy = 0.106521

C	-0.43091100	1.19406000	-0.00002000
C	0.20491300	-0.05333300	0.00002900
C	-0.57357500	-1.21783100	0.00009700
C	-1.96172400	-1.13755300	0.00008000
C	-2.58808700	0.11014900	-0.00003300
C	-1.82221300	1.27494000	-0.00007400
H	0.15447700	2.11562500	-0.00001700
H	-0.05864600	-2.18040400	0.00011300
H	-2.56083100	-2.05073600	0.00013900
H	-3.67853100	0.17513800	-0.00009100
H	-2.31055700	2.25155200	-0.00017600
C	1.70167500	-0.20571800	-0.00004600
O	2.20690900	-1.30512100	-0.00016700
C	2.55368600	1.04466700	0.00010000
H	2.34326200	1.65968800	0.88830100
H	3.61046300	0.75318000	-0.00049000
H	2.34250900	1.66064100	-0.88724400

$[D\text{-}E]_b^{‡}$:

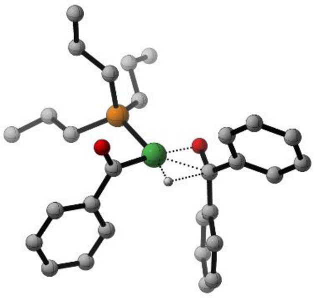

E_{el} = -3127.020008

Zero-point correction = 0.592075

Thermal correction to Energy = 0.627198

Thermal correction to Enthalpy = 0.628143

Thermal correction to Gibbs Free Energy = 0.520793

Ni	0.26074800	-0.26971900	-0.25424700
P	-1.83090800	-0.73339300	0.29677400
C	-2.96703300	0.71832100	0.27122400
C	-2.45734500	-1.93518500	-0.94931600
C	-1.96061800	-1.50604600	1.96350500
C	-3.96222800	-2.11874200	-1.14792300
C	-4.26579700	-3.15293600	-2.22787600
C	-3.13000700	-2.44232500	2.26770700
C	-3.06480500	-2.99336000	3.68888400
C	-4.30343600	0.64141800	1.00808900
C	-5.11323000	1.92484600	0.84898100
H	-1.97221700	-2.89569800	-0.70299200
H	-1.99762100	-1.59495000	-1.89328900
H	-4.44600600	-2.41796800	-0.20512500
H	-4.41138600	-1.15381600	-1.43407200
H	-1.92978400	-0.66897400	2.68299800
H	-1.00382400	-2.03636200	2.09495300
H	-4.09006400	-1.92478400	2.11717200
H	-3.11839700	-3.28028200	1.55167000
H	-3.11972500	0.94835500	-0.79730300
H	-2.37535600	1.56291600	0.66207100
H	-4.89378400	-0.21321800	0.64403900
H	-4.12172700	0.45478300	2.07941800
H	-3.85819800	-4.13966100	-1.95711700
H	-3.81618900	-2.86165900	-3.18967200
H	-5.33636800	2.12154000	-0.21129100
H	-4.55983100	2.79453500	1.23608200
H	-3.12390800	-2.18310700	4.43256400
H	-2.11966900	-3.53148000	3.86052900
C	-0.32558400	0.77240600	-1.69100400
C	-0.40885200	2.26552600	-1.47449900
O	-0.74015900	0.28206400	-2.72345500
C	0.20867300	2.87147700	-0.37603900
C	-0.67095600	5.01567100	-1.05053900
C	-1.15281000	3.04803900	-2.36461700
C	-1.28763200	4.41660000	-2.15171100
H	0.80022600	2.26416100	0.31479900
H	-0.77713000	6.09022700	-0.88397800
H	-1.61903400	2.55481300	-3.22067400
H	-1.87407200	5.02385900	-2.84518800
H	-6.06967500	1.86483500	1.38900500
H	-5.34902600	-3.26762400	-2.38133100
H	-3.89136700	-3.69176600	3.88676000
C	0.08176600	4.24340300	-0.16610000
H	0.57331700	4.70856600	0.69114600

H	1.43760300	0.20354700	-1.01042400
C	3.67407400	2.85475800	2.28031900
C	2.75758100	2.06367100	2.97064200
C	2.27243900	0.88948000	2.39489500
C	2.70319200	0.49750700	1.12529600
C	3.62805700	1.29264500	0.43874800
C	4.10937600	2.46471200	1.01169400
H	4.05159700	3.77626600	2.72903800
H	2.41609800	2.36216700	3.96445300
H	1.54523800	0.26272500	2.91418200
H	3.95198900	0.99945000	-0.56291100
H	4.82138600	3.08431100	0.46245700
C	2.10442000	-0.74032500	0.49552200
O	1.12416500	-1.30708800	1.10611500
C	3.00899700	-1.62648600	-0.33742400
C	2.53930400	-2.27000900	-1.48645400
C	4.30867300	-1.88928900	0.10552700
C	3.35452700	-3.15721400	-2.18249100
H	1.52919100	-2.05353700	-1.84518300
C	5.12525500	-2.78083900	-0.59013700
H	4.68683100	-1.39384100	1.00280100
C	4.65110400	-3.41554900	-1.73592900
H	2.97752300	-3.64344200	-3.08472200
H	6.13854600	-2.97727400	-0.23286300
H	5.29281500	-4.10745100	-2.28583500

[D-E]$_c^{‡}$:

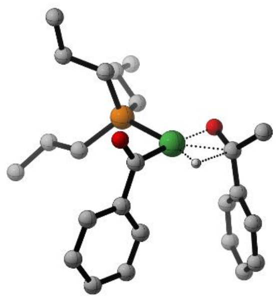

E_{el} = -2935.366391

Zero-point correction = 0.539018

Thermal correction to Energy = 0.570618

Thermal correction to Enthalpy = 0.571562

Thermal correction to Gibbs Free Energy = 0.474854

Ni	0.32492100	-0.63090200	-0.98704700
P	-1.63784300	-0.37489800	0.01216700
C	-1.77787900	1.19131400	0.97463800
C	-2.92628800	-0.32658000	-1.30186600

C	-2.03826600	-1.75722000	1.16139200
C	-4.28275400	0.31924800	-1.01816900
C	-5.20216200	0.26184100	-2.23443300
C	-3.50227000	-2.07824200	1.46212600
C	-3.64520800	-3.24329100	2.43682900
C	-2.86970300	1.34186500	2.03312900
C	-2.82098400	2.71219200	2.70266700
H	-3.04885000	-1.37154800	-1.63585700
H	-2.43123400	0.20345000	-2.13384500
H	-4.77475400	-0.16701900	-0.16124300
H	-4.12791900	1.37193000	-0.73066900
H	-1.48608800	-1.53820700	2.09237900
H	-1.53547900	-2.63529600	0.72536600
H	-4.01456100	-1.19324100	1.86969800
H	-4.02073100	-2.32563200	0.52133700
H	-1.84890000	1.99172800	0.21814200
H	-0.78894200	1.33298100	1.44172500
H	-3.86326800	1.18555000	1.58581600
H	-2.75023000	0.55762600	2.79907500
H	-5.41181000	-0.78031800	-2.52276700
H	-4.74073200	0.75955700	-3.10123200
H	-2.97332100	3.51677500	1.96645400
H	-1.84443700	2.88426500	3.18158800
H	-3.16583400	-3.01541600	3.40186000
H	-3.17066100	-4.15327700	2.03789100
C	0.26800200	1.14815700	-1.55944000
C	1.01768900	2.17814700	-0.74224300
O	-0.43139200	1.50893300	-2.48610100
C	1.90988600	1.79791300	0.26456800
C	2.31397600	4.11642100	0.80053100
C	0.78135000	3.53794400	-0.97337500
C	1.42417300	4.50350100	-0.20446800
H	2.10121000	0.73725000	0.44545900
H	2.82030700	4.87440800	1.40302400
H	0.08302000	3.81187000	-1.76746200
H	1.23586800	5.56403600	-0.38732000
H	-3.59842400	2.80909800	3.47490500
H	-6.16479900	0.75496600	-2.03357500
H	-4.70306300	-3.47261800	2.63304400
C	2.55870200	2.76324800	1.03260200
H	3.25935300	2.45459900	1.81152900
H	1.52000100	-0.38614700	-1.82514100
C	4.76034700	-0.96017100	1.76113900
C	3.56290400	-1.52116300	2.20307600
C	2.59561000	-1.91995200	1.28268200

C	2.81418300	-1.76372000	-0.09012500
C	4.02330700	-1.21010400	-0.52649800
C	4.98770600	-0.80729500	0.39264300
H	5.51674900	-0.64117600	2.48152300
H	3.38066900	-1.64947800	3.27241900
H	1.64788100	-2.35100600	1.60960900
H	4.20109800	-1.05385700	-1.59228500
H	5.91979700	-0.36171600	0.03871900
C	1.70724400	-2.15212200	-1.04575600
O	0.58308900	-2.48876200	-0.53110300
C	2.08985000	-2.76224700	-2.38005700
H	2.85897300	-2.18919200	-2.91152200
H	2.47987600	-3.77556300	-2.19075700
H	1.19902300	-2.84011400	-3.01500000

$[D\text{-}E]_d^{\ddagger}$:

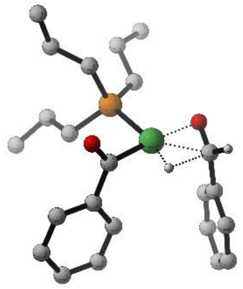

E_{el} = -2896.065217

Zero-point correction = 0.509824

Thermal correction to Energy = 0.540248

Thermal correction to Enthalpy = 0.541192

Thermal correction to Gibbs Free Energy = 0.445756

Ni	0.29701600	-0.56786800	-1.22555000
P	-1.60166100	-0.35315900	-0.10015400
C	-1.53068800	0.96116700	1.19188800
C	-2.93726500	0.10246900	-1.28178900
C	-2.09165000	-1.91486500	0.74590300
C	-4.20452700	0.78577900	-0.76799500
C	-5.19314500	1.07196500	-1.89422100
C	-3.56768300	-2.16884900	1.05248100
C	-3.78490900	-3.51266800	1.74153700
C	-2.55333500	0.95955900	2.32745900
C	-2.32283200	2.11103500	3.30170800
H	-3.18255000	-0.82869700	-1.82155700
H	-2.43160600	0.75381800	-2.01492500
H	-4.69416900	0.16869900	0.00133700
H	-3.93173300	1.73397200	-0.27673900
H	-1.48702600	-1.95436800	1.66925600

H	-1.69110900	-2.71594700	0.10459700
H	-3.97566400	-1.36300400	1.68208200
H	-4.14305900	-2.14517000	0.11249300
H	-1.55054800	1.91827400	0.64271500
H	-0.51308600	0.89790600	1.61230600
H	-3.57544900	1.01780600	1.92331200
H	-2.49257400	0.00366700	2.87338700
H	-5.51486300	0.14050800	-2.38596800
H	-4.73976700	1.71476500	-2.66440900
H	-2.40532200	3.08368100	2.79224100
H	-1.31820800	2.05679000	3.74927400
H	-3.25523100	-3.55458200	2.70625500
H	-3.40934600	-4.34092000	1.12082100
C	0.36051200	1.28327500	-1.50183000
C	1.21845700	2.12676700	-0.58539400
O	-0.34550200	1.82581300	-2.32927000
C	2.12500000	1.54628800	0.30623500
C	2.73365200	3.73162400	1.13157900
C	1.07659800	3.51900000	-0.61183400
C	1.82902200	4.31826300	0.24322900
H	2.24443200	0.46037200	0.32628500
H	3.32725600	4.35946500	1.80035300
H	0.36591300	3.95002300	-1.32072900
H	1.71466300	5.40453800	0.21810800
H	-3.05846500	2.09579000	4.11933500
H	-6.09272200	1.57988900	-1.51612800
H	-4.85220700	-3.69419600	1.93643500
C	2.88295200	2.34527200	1.16072300
H	3.59362700	1.88129100	1.84824300
H	1.49379600	-0.28531100	-2.05317100
C	4.75705500	-1.64877900	1.34506200
C	3.49756300	-2.08303300	1.75927800
C	2.47656200	-2.25390600	0.82692400
C	2.70940600	-1.98855900	-0.52558500
C	3.97698000	-1.56864500	-0.93880700
C	4.99627200	-1.39607600	-0.00716100
H	5.55613300	-1.51076600	2.07684200
H	3.31129600	-2.28910000	2.81564800
H	1.48177300	-2.58973800	1.12669500
H	4.15527200	-1.35209600	-1.99575500
H	5.98148400	-1.05692700	-0.33437300
C	1.58515400	-2.11302100	-1.50948400
O	0.43006000	-2.49201500	-1.11554100
H	1.88616600	-2.29751700	-2.55748100

[F-G]$_b^‡$:

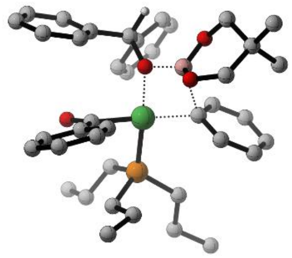

E_{el} = -3730.475781

Zero-point correction = 0.847799

Thermal correction to Energy = 0.895612

Thermal correction to Enthalpy = 0.896556

Thermal correction to Gibbs Free Energy = 0.766225

Ni	0.22619600	-0.16693400	-0.03016500
P	1.73616400	-1.75129600	-0.02364000
C	3.03027100	-1.55333600	-1.31682500
C	2.60958100	-1.74634700	1.60088900
C	1.15375000	-3.48003800	-0.23941700
C	4.09413900	-2.11174300	1.62113500
C	4.65278000	-2.12157800	3.04033800
C	2.17298200	-4.60365100	-0.06899000
C	1.54162400	-5.97472200	-0.29430600
C	2.44938700	-1.60743100	-2.73284400
C	3.39443000	-1.01578000	-3.77212200
H	2.02451400	-2.41561900	2.25489700
H	2.49312000	-0.72683800	1.99491700
H	4.26538100	-3.09288800	1.15014000
H	4.65004500	-1.37776700	1.01658300
H	0.69394000	-3.52787500	-1.23762300
H	0.31745800	-3.60649700	0.46659400
H	3.01291000	-4.46678000	-0.77081500
H	2.60747300	-4.56728400	0.94322300
H	3.81657800	-2.31368200	-1.18764500
H	3.50621400	-0.57654000	-1.14058800
H	2.20852700	-2.65277300	-2.98977800
H	1.49516600	-1.05597900	-2.76086200
H	4.14296400	-2.87373400	3.66324000
H	4.51659100	-1.14118000	3.52287300
H	4.37004300	-1.52734100	-3.76502700
H	3.56796400	0.05307600	-3.57040500
H	1.11806800	-6.05513500	-1.30760300
H	0.72181500	-6.15123300	0.41917600
C	1.72728300	1.02129900	-0.06509700

C	1.97761600	1.69753100	-1.39877200
O	2.48042800	1.27566500	0.86002200
C	1.05140000	1.61741400	-2.44406700
C	2.50962000	2.93475000	-3.84959900
C	3.16725100	2.40973300	-1.58769200
C	3.43697200	3.02052700	-2.80860700
H	0.10197300	1.09385600	-2.29990300
H	2.71755400	3.41596500	-4.80821600
H	3.86705500	2.47300200	-0.75152100
H	4.37146700	3.56779300	-2.95273700
H	2.97515300	-1.10202900	-4.78547800
H	5.72828400	-2.35295900	3.04685800
H	2.27921900	-6.78167900	-0.17093500
C	1.31555300	2.23837600	-3.66382100
H	0.58270400	2.18046900	-4.47154400
O	-1.06297600	1.29765600	0.17144600
C	-1.22969300	2.06426000	1.37256000
H	-2.25337200	2.46647700	1.33250900
C	-1.14066400	1.17012500	2.59417700
C	0.07978600	0.60563500	2.98632900
C	-2.30005500	0.83322000	3.29509000
C	0.12870300	-0.29462800	4.04804000
H	0.99782000	0.88313500	2.46129600
C	-2.24971700	-0.05890300	4.36605800
H	-3.25733700	1.24892200	2.97519500
C	-1.03570200	-0.63164900	4.73969900
H	1.08629400	-0.73259300	4.33923700
H	-3.16645600	-0.31677300	4.90075300
H	-0.99568100	-1.33919000	5.57101100
C	-2.46709800	-3.31424500	0.96126500
C	-1.86663100	-2.05428200	0.98553200
C	-1.38420200	-1.43136900	-0.18268300
C	-1.55449700	-2.14974800	-1.38284100
C	-2.14529000	-3.41239300	-1.42617800
C	-2.60157700	-4.00119600	-0.24686100
H	-2.83273500	-3.76398700	1.88814100
H	-1.77529100	-1.53800400	1.94551400
H	-1.20116200	-1.70223500	-2.31835200
H	-2.25506300	-3.93790900	-2.37864400
H	-3.07132900	-4.98761500	-0.26998100
C	-3.37716100	0.35262700	-2.51868600
C	-4.50005400	0.99424400	-0.38971500
C	-4.67514700	0.19115100	-1.68213100
H	-3.16929200	-0.59268300	-3.05046000
H	-3.52716800	1.13093300	-3.28508200

H	-5.36990700	0.83929900	0.26814500
H	-4.45086900	2.07591200	-0.62376700
B	-2.16908000	0.59786900	-0.41137500
O	-3.34977900	0.60125000	0.31837300
O	-2.23578800	0.73566700	-1.78847300
C	-4.94424200	-1.27442800	-1.32251200
H	-5.91362900	-1.36915700	-0.80782800
H	-4.97711100	-1.90474900	-2.22478200
H	-4.17010900	-1.67457700	-0.65617100
C	-5.85662300	0.75034400	-2.47936700
H	-5.96814300	0.21727400	-3.43682200
H	-6.80012500	0.63233500	-1.92390400
H	-5.72362600	1.82076000	-2.70294700
C	-0.26857000	3.23920000	1.32520700
C	0.32143600	3.76455400	2.47661200
C	-0.01267500	3.85104600	0.09373700
C	1.17299500	4.86553000	2.39398300
H	0.12767500	3.30404400	3.44703200
C	0.84394200	4.94426400	0.00915300
H	-0.47031300	3.44308700	-0.80857600
C	1.44331100	5.45359600	1.16044400
H	1.63559800	5.25926100	3.30167300
H	1.05000500	5.39342900	-0.96483500
H	2.12225800	6.30686800	1.09627200

$[F\text{-}G]_c^{\ddagger}$:

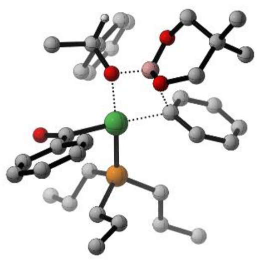

E_{el} = -3538.821461

Zero-point correction = 0.793675

Thermal correction to Energy = 0.838305

Thermal correction to Enthalpy = 0.839249

Thermal correction to Gibbs Free Energy = 0.716992

Ni	0.25872200	0.17811100	0.28277200
P	1.99849500	-0.78328800	-0.63538500
C	3.30274200	0.38675300	-1.19885400
C	2.78225600	-1.88197700	0.62005700
C	1.70924600	-1.85086800	-2.10175000
C	4.29816800	-2.07469300	0.56385400

C	4.78135300	-3.06345700	1.62010000
C	2.86453200	-2.71139700	-2.60648400
C	2.47888900	-3.48035900	-3.86723400
C	2.80058200	1.33576300	-2.29216100
C	3.67219200	2.57851600	-2.43077300
H	2.25349900	-2.84746700	0.54168900
H	2.51671300	-1.44708900	1.59430800
H	4.61617600	-2.41776400	-0.43337800
H	4.78815500	-1.10156800	0.72636500
H	1.35643700	-1.17840600	-2.89822700
H	0.84201900	-2.48207600	-1.85243500
H	3.75045200	-2.08747400	-2.81378900
H	3.16630300	-3.42633900	-1.82402400
H	4.18885000	-0.17049900	-1.54117800
H	3.61701300	0.96082200	-0.31362200
H	2.75316700	0.79612900	-3.25299000
H	1.76773200	1.64826500	-2.06552100
H	4.32811400	-4.05647900	1.47108400
H	4.51147100	-2.72241300	2.63173200
H	4.72014100	2.31373400	-2.64413700
H	3.65287600	3.16693400	-1.49996500
H	2.21358900	-2.79149800	-4.68435600
H	1.60401800	-4.12278400	-3.68215800
C	1.54800700	1.17943000	1.27270900
C	1.69126000	2.63127400	0.86630800
O	2.24219900	0.76859100	2.19131900
C	0.79278700	3.23483900	-0.02088200
C	2.06470100	5.28808000	0.07734500
C	2.76924000	3.37366400	1.36496200
C	2.96033800	4.69383200	0.96952200
H	-0.06981500	2.67801100	-0.39713700
H	2.21265700	6.32459500	-0.23486700
H	3.45060300	2.88407000	2.06459000
H	3.80838000	5.26432000	1.35527100
H	3.31559100	3.22754500	-3.24414300
H	5.87420800	-3.18487300	1.58593400
H	3.30266200	-4.11984300	-4.21752300
C	0.98005800	4.56025500	-0.41081400
H	0.27208000	5.02700400	-1.09914600
O	-1.24540600	0.92801300	1.29180700
C	-1.58264300	0.57407300	2.64664800
H	-2.65718900	0.77828100	2.75056400
C	-1.37407600	-0.91017900	2.88894200
C	-0.08775200	-1.45467800	2.98901900
C	-2.47617700	-1.76588100	2.96563200

C	0.08751800	-2.82891600	3.13993200
H	0.78773500	-0.80208000	2.94399400
C	-2.30255500	-3.13930500	3.13101600
H	-3.48002600	-1.35110100	2.85764400
C	-1.01870300	-3.67593200	3.21104000
H	1.09777800	-3.23942300	3.20925200
H	-3.17489600	-3.79459800	3.18358200
H	-0.87946000	-4.75248800	3.33283300
C	-1.88830800	-3.02376300	-1.51867800
C	-1.50813800	-2.08605300	-0.55730200
C	-1.12476400	-0.77413600	-0.90037700
C	-1.15345200	-0.45983900	-2.27362100
C	-1.52411800	-1.38511000	-3.24892300
C	-1.89186300	-2.67653600	-2.87127500
H	-2.18240600	-4.03133200	-1.21295400
H	-1.51803400	-2.38326800	0.49502000
H	-0.86405700	0.54842200	-2.58897500
H	-1.52871400	-1.10247100	-4.30515800
H	-2.18909700	-3.40814000	-3.62661600
C	-3.30671700	1.92218000	-1.58535400
C	-4.56481800	0.76838000	0.22546700
C	-4.58208100	1.09390700	-1.27071600
H	-2.93738600	1.65164300	-2.59045800
H	-3.56225300	2.99440600	-1.61205200
H	-5.41681600	0.11665900	0.47715000
H	-4.67735500	1.70142500	0.81230100
B	-2.20014300	0.72969200	0.23860300
O	-3.38605200	0.09853900	0.59764700
O	-2.26059200	1.79150000	-0.65264300
C	-4.62458400	-0.21817600	-2.06234300
H	-5.58524400	-0.73150600	-1.89683700
H	-4.52124500	-0.03371400	-3.14287100
H	-3.82263500	-0.90097400	-1.75583900
C	-5.81832400	1.93328500	-1.60462500
H	-5.82869900	2.20173800	-2.67298800
H	-6.74414000	1.37453200	-1.39573600
H	-5.84496400	2.86770800	-1.02167900
C	-0.82057600	1.48093200	3.59604000
H	-1.15631300	1.29391000	4.62639000
H	0.26225800	1.31211400	3.55295000
H	-1.01738500	2.53257400	3.34462700

[F-G]$_d$‡:

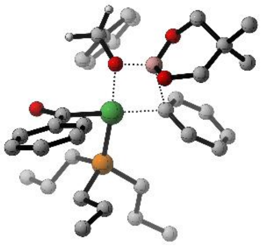

E_{el} = -3499.521767

Zero-point correction = 0.766402

Thermal correction to Energy = 0.809514

Thermal correction to Enthalpy = 0.810458

Thermal correction to Gibbs Free Energy = 0.691405

Ni	0.24896800	0.17325900	0.38029200
P	1.96140100	-0.73490500	-0.63153500
C	3.25316500	0.47313200	-1.13699500
C	2.78522500	-1.92202100	0.51405000
C	1.61556600	-1.68796500	-2.16259800
C	4.29602400	-2.11915500	0.38032500
C	4.81731100	-3.17568000	1.34901000
C	2.75038000	-2.49493200	-2.78732900
C	2.31189300	-3.16593200	-4.08603200
C	2.72758300	1.50524800	-2.13900400
C	3.61666100	2.74054900	-2.22070600
H	2.24762500	-2.87796200	0.39152300
H	2.56053900	-1.55711900	1.52727900
H	4.56490700	-2.39803200	-0.65064800
H	4.80201200	-1.16142500	0.58121300
H	1.22029200	-0.95799400	-2.88538500
H	0.76210800	-2.34243200	-1.92447100
H	3.62100600	-1.84676500	-2.98504500
H	3.09392700	-3.26584800	-2.07849400
H	4.13073500	-0.05429700	-1.54275500
H	3.58752400	0.97655500	-0.21636100
H	2.63456700	1.03619000	-3.13300100
H	1.70958300	1.81499000	-1.85008700
H	4.34981400	-4.15475000	1.15799600
H	4.59549200	-2.89934000	2.39156000
H	4.65070300	2.47462400	-2.49267400
H	3.64326400	3.25980600	-1.24988400
H	1.99738100	-2.41838100	-4.83082400
H	1.45606000	-3.83643600	-3.91196600
C	1.53141400	1.04548000	1.48728000
C	1.75800300	2.51352600	1.19006800
O	2.13980300	0.54359900	2.41902500

C	0.89504800	3.23676300	0.35832500
C	2.28366100	5.19941400	0.61378100
C	2.87728000	3.15053200	1.74058600
C	3.14500600	4.48445300	1.44887900
H	-0.00000900	2.76219700	-0.05399300
H	2.48948500	6.24786700	0.38559100
H	3.52934700	2.57061000	2.39768700
H	4.02530000	4.97209600	1.87397500
H	3.24295300	3.45285000	-2.97108400
H	5.90641200	-3.30076900	1.25755600
H	3.12575100	-3.76067400	-4.52672300
C	1.15762900	4.57669500	0.07585700
H	0.47650400	5.13895800	-0.56655400
O	-1.19639900	0.86029500	1.48231700
C	-1.42394900	0.33972900	2.79213000
H	-2.40928000	0.69032500	3.12935300
C	-1.34460800	-1.16828400	2.86200100
C	-0.09329400	-1.79623800	2.88113900
C	-2.49930600	-1.95562300	2.86561200
C	-0.00022500	-3.18658200	2.87286300
H	0.81165400	-1.18231200	2.90315300
C	-2.40724300	-3.34678300	2.87279400
H	-3.47575800	-1.47079500	2.82870500
C	-1.15760800	-3.96545400	2.86638000
H	0.98282600	-3.66335800	2.88213600
H	-3.31753700	-3.95058800	2.87149200
H	-1.08529600	-5.05556300	2.86310900
C	-2.00647100	-2.73416900	-1.76388300
C	-1.60532100	-1.93091100	-0.69464900
C	-1.19607500	-0.59477700	-0.87406600
C	-1.22318600	-0.11112300	-2.19731100
C	-1.61267400	-0.90047400	-3.27894100
C	-2.00440900	-2.22167600	-3.06262700
H	-2.32121500	-3.76554000	-1.58447000
H	-1.61946300	-2.35698800	0.31238300
H	-0.92380900	0.92580100	-2.38375400
H	-1.61549100	-0.48681700	-4.29099800
H	-2.31704200	-2.84824400	-3.90176300
C	-3.34680400	2.18848900	-1.18127700
C	-4.56326000	0.86453100	0.53703300
C	-4.61597100	1.33502200	-0.91948600
H	-3.02362800	2.04520100	-2.22804700
H	-3.59217000	3.25666700	-1.06024100
H	-5.41620300	0.19942500	0.74747400
H	-4.64857300	1.73635200	1.21519300

B	-2.19782200	0.78330900	0.45504700
O	-3.38331700	0.15073500	0.81220900
O	-2.26474700	1.93325900	-0.31870700
C	-4.68353700	0.10843500	-1.83668900
H	-5.63635200	-0.42435200	-1.68858700
H	-4.62059400	0.40138800	-2.89614300
H	-3.86969700	-0.59719100	-1.62975900
C	-5.85582600	2.20640700	-1.13733000
H	-5.88201600	2.59644700	-2.16722500
H	-6.77918900	1.62733700	-0.97974900
H	-5.87256000	3.06770100	-0.45057300
H	-0.65010800	0.78951700	3.42959700

[F-P]‡:

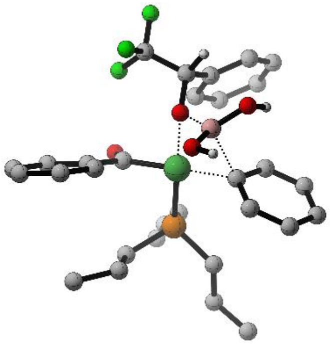

E_{el} = -3641.350420

Zero-point correction = 0.648987

Thermal correction to Energy = 0.691064

Thermal correction to Enthalpy = 0.692008

Thermal correction to Gibbs Free Energy = 0.573815

Ni	-0.00219900	0.04458100	-0.32321000
P	2.03417900	0.09513600	0.46184200
C	2.78273900	1.76876400	0.59683000
C	1.97139800	-0.61280000	2.16305200
C	3.31040800	-0.88509800	-0.42615800
C	2.94323700	-0.06121900	3.20672300
C	2.78913800	-0.76687700	4.55018700
C	4.69979800	-0.97948200	0.19990000
C	5.65254000	-1.79332200	-0.67129600
C	2.96163100	2.43102500	-0.77350500
C	3.12331800	3.94323800	-0.67291900
H	2.09451800	-1.70220700	2.03690400
H	0.94203100	-0.43860600	2.50953300
H	3.98504500	-0.15075400	2.85951700
H	2.75228600	1.01541100	3.33906400
H	3.37128000	-0.46391500	-1.44236000

H	2.88386100	-1.89192200	-0.55185000
H	5.12063600	0.02665800	0.36397200
H	4.62558900	-1.45020000	1.19352800
H	3.74275000	1.70623600	1.13235400
H	2.10990000	2.37002600	1.22637400
H	3.83452900	1.98805100	-1.28213100
H	2.08837500	2.20639700	-1.40853100
H	2.98785600	-1.84639400	4.45709500
H	1.76707500	-0.64757800	4.94220900
H	3.97669600	4.21419900	-0.03095400
H	2.21654500	4.39901500	-0.24510000
H	5.78894300	-1.32104100	-1.65670000
H	5.25772800	-2.80682900	-0.84268000
C	-0.29268600	1.51992900	0.85260100
C	-0.44921900	2.88198600	0.21939200
O	-0.36211500	1.42830100	2.06771600
C	-0.53718400	3.03848800	-1.16775800
C	-0.65460000	5.43692400	-0.89489600
C	-0.47802900	4.01359200	1.04471700
C	-0.57452700	5.28600400	0.49165800
H	-0.53994300	2.16465700	-1.82496800
H	-0.73363500	6.43501300	-1.33226700
H	-0.42027100	3.86423400	2.12516000
H	-0.59034500	6.16549700	1.13962000
H	3.28871600	4.39321300	-1.66283100
H	3.48553800	-0.36057800	5.29858900
H	6.64323100	-1.88925200	-0.20286000
C	-0.64142000	4.31394000	-1.72117300
H	-0.71873600	4.43022400	-2.80421300
O	-1.89937100	-0.04658000	-0.91915800
C	-3.03781400	-0.56190400	-0.26535600
H	-3.70707500	-0.97198500	-1.03602300
C	-2.71291300	-1.66770600	0.71533600
C	-1.91322700	-1.44173300	1.84251600
C	-3.15047100	-2.96576100	0.43748800
C	-1.54755700	-2.50817100	2.66101900
H	-1.56048800	-0.43715500	2.08389800
C	-2.78608200	-4.03100200	1.25987200
H	-3.77646400	-3.14771300	-0.44042200
C	-1.97727200	-3.80347000	2.37151000
H	-0.91912300	-2.32133400	3.53460800
H	-3.13193300	-5.04054300	1.02841400
H	-1.68456100	-4.63531900	3.01594400
C	-3.82559600	0.60796300	0.32460500
F	-4.12917500	1.48759800	-0.62707100

F	-3.18001700	1.26037100	1.28763800
F	-4.97012600	0.14851400	0.84155000
C	0.98879600	-3.94123600	-1.25051900
C	0.30684900	-2.80091200	-0.82245200
C	0.37097500	-1.57644100	-1.52422700
C	1.16822400	-1.57280400	-2.68352700
C	1.84921000	-2.70509000	-3.12974800
C	1.76268100	-3.89752500	-2.41146100
H	0.91615800	-4.86867400	-0.67621000
H	-0.28448100	-2.87113300	0.09639700
H	1.25569200	-0.64483700	-3.25772800
H	2.45346300	-2.65949400	-4.03972600
H	2.29242700	-4.78905400	-2.75554800
B	-1.47708700	-0.65213700	-2.16924600
O	-2.12174400	-1.79867700	-2.61581100
O	-1.12725200	0.31042100	-3.09807200
H	-1.90258100	-2.57505700	-2.09379600
H	-0.99326000	-0.09374000	-3.95873600
